# Transcriptomic profiles of muscle, heart, and spleen in reaction to circadian heat stress in Ethiopian highland and lowland male chicken

**DOI:** 10.1007/s12192-018-0954-6

**Published:** 2018-12-18

**Authors:** Marinus F. W. te Pas, Woncheoul Park, Krishnamoorthy Srikanth, Steve Kemp, Jun-Mo Kim, Dajeong Lim, Jong-Eun Park

**Affiliations:** 1 Wageningen UR Livestock Research, Animal Breeding and Genomics, Wageningen, The Netherlands; 2 Department of Animal Biotechnology and Environment, Rural Development Administration, National Institute of Animal Science, Wanju, 55365 Republic of Korea; 3 Animal Biosciences, International Livestock Research Institute (ILRI), Nairobi, Kenya; 4 Department of Animal Science and Technology, Chung-Ang University, Anseong, Gyeonggi-do 17546 Republic of Korea

**Keywords:** Highland and lowland male chicken, Heat stress, Transcriptome profiles, Heart, Breast muscle—meat, Spleen

## Abstract

Temperature stress impacts both welfare and productivity of livestock. Global warming is expected to increase the impact, especially in tropical areas. We investigated the biological mechanisms regulated by temperature stress due to the circadian temperature cycle in temperature adapted and non-adapted chicken under tropical conditions. We studied transcriptome profiles of heart, breast muscle, and spleen tissues of Ethiopian lowland chicken adapted to high circadian temperatures and non-adapted Ethiopian highland chicken under lowland conditions at three points during the day: morning, noon, and evening. Functional annotations and network analyses of genes differentially expressed among the time points of the day indicate major differences in the reactions of the tissues to increasing and decreasing temperatures, and also the two chickens lines differ. However, epigenetic changes of chromatin methylation and histone (de)acetylation seemed to be central regulatory mechanisms in all tissues in both chicken lines. Finally, all tissues showed differentially expressed genes between morning and evening times indicating biological mechanisms that need to change during the night to reach morning levels again the next day.

**Electronic supplementary material:**

The online version of this article (10.1007/s12192-018-0954-6) contains supplementary material, which is available to authorized users.

## Introduction

Environmental changes, especially temperature changes, influence animal health, welfare, and livestock productivity. Temperatures above normal are felt as heat stress (HS) by all living organisms, and heat stress can be defined as the sum of all external forces acting on an animal that causes an increase in body temperature and evokes a physiological response (Fuquay 1981; Phillips and Piggins 1992, Dikmen and Hansen 2009). For example, it has been shown that the level of productivity and health of dairy cattle, goat, and sheep and layer and broiler chicken are reduced by heat stress, and chicken mortality is increased (Fuquay 1981; Bottje and Harrison 1985; Lu 1989; Young 1990; Yahav et al. 1995; Silanikove 1992; West 2003; Marai et al. 2007). Physiologically, this may relate to reduced feed intake, suppressed immune response, and changed gene expression profiles and animal metabolism (Harrison and Biellier 1969; Emery et al. 1984; Altan et al. 2003; Mashaly et al. 2004; Lin et al. 2006a; Srikanth et al. 2017).

In tropical environments, the daily temperature rise may induce heath stress to chicken. Local environmental conditions, such as altitude and natural conditions, e.g., forests, affect the level and the impact of the temperature rise during the day. Global warming may increase the local temperatures further increasing heat stress effects. This may especially affect future livestock productivity in sub-Saharan countries (Thornton et al. 2009).

The impact of heat on broiler and layer strains of chicken has been studied before (Zahoor et al. 2017). The impact of heat stress in both genotypes of poultry is very similar (Zahoor et al. 2016, 2017). Apart from decreased productivity levels, physiological and behavioral changes were noted affecting not only animal welfare but potentially also consumer health (Lara and Rostagno, 2013). Furthermore, acute heat stress-affected gene expression profiles in the testis of broiler chickens increased the number of apoptotic cells, suggestive for reduced fertility (Wang et al. 2015). Changed gene expression in muscle tissue of broiler chickens has also been reported, suggestive for reduced meat productivity (Li et al. 2011).

As with most environmental challenges, animals living for generations in hot climates adapt to the environmental conditions (Lin et al. 2006b; Renaudeau et al. 2012; Lara and Rostagno, 2013; Kim et al. 2017). Chickens can adapt to circadian heath cycles (Lin et al. 2006b; Renaudeau et al. 2012; Lara and Rostagno, 2013). Furthermore, increasing the temperature to 1–1.5 °C during days 8–21 of egg incubation induced an adaptive response to hot temperatures in later life (Moraes et al. 2003; Janke and Tzschentke, 2010; Loyau et al. 2014a, b, 2015, 2016), including changed genome activity, suggesting epigenomic mechanisms (Loh et al. 2004; Yossifoff et al. 2008).

Together, these data suggest that the transcriptomic changes mediating the response of the chicken to heat stress differ between adapted and non-adapted chickens. We here report on the differences in the transcriptomic profiles and the underlying regulated biological mechanisms in three vital tissues: heart, muscle (meat productivity), and spleen (immune) using a model system of chicken adapted to hot environments and non-adapted chickens. The objectives of these studies were to determine (1) the transcriptome profile differences between highland and lowland Ethiopian chicken before, during, and after hot daytime temperatures and (2) the transcriptome profile changes in highland and lowland Ethiopian chicken separately during the day.

## Materials and methods

### Animals and experimental design

The care and experimental use of Ethiopia chicken were reviewed and approved by the Institutional Animal Care and Use Committee at the International Livestock Research Institute (ILRI, Ethiopia) (IACUC No. 2016-216).

Ethiopian highland male chickens (*N* = 9) from Addis Ababa (altitude 2400 m above sea level, average high temperature 16 °C) were collected randomly and transferred to the Ethiopian lowland Awash village (altitude 950 m above sea level, average high temperature 37 °C), where the experiments were performed after a 20-h free-range period to relieve the animals from the transportation stress. Similarly, Ethiopian male lowland chicken from Awash village (Yangji hatchery, *N* = 9) were collected randomly. The Ethiopian endogenous chicken lines originated from the same route to Ethiopia but separated to different environmental conditions many generations ago. The lowland chickens were adapted to the circadian high temperature cycle, while the highland chickens were not.

All chickens were subjected to the lowland circadian temperature cycle at the same day. The chickens were slaughtered at the experimental location at three different time points: early in the morning at 6.00 AM (i.e., before the onset of increasing temperature; measured conditions 25.2 °C, 64% humidity), at noon, 12.00 PM (i.e., the hottest temperature; measured conditions 34.6 °C, 6% humidity), and in the evening at 6.00 PM (i.e., after the temperature decline; measured conditions 26.3 °C, 9% humidity). Three chickens of each group were sacrificed per time point. Heart, breast muscle, and spleen tissue were snap-frozen and stored at − 80 °C until experimentation. The tissues were transferred to the Republic of Korea for RNA-Seq analysis.

### RNA-Seq analysis

RNA was isolated using TRIzol (Invitrogen, USA). Hundred milligrams of total RNA of each tissue were used to construct libraries for sequencing. The mRNA-Seq library was constructed with TruSeq mRNA library (Illumina Inc.) and sequenced with Illumina HiSeq2000 100PE (Illumina, Inc., San Diego, CA, USA) sequencing in accordance with the manufacturer’s instructions. To remove rRNA, bead-based depletion was used. Libraries were sequenced independently. Obtained sequence reads were aligned to the *Gallus gallus* reference genome (NCBI GenBank, ftp://ftp.ensembl.org/pub/release-89/fasta/gallus_gallus/dna/). Preprocessing and mapping were performed using the pipelines employing FastQC, trimmomatic, and Tophat2. The read mapping was conducted using the RNA-Seq analysis package default settings (mismatch cost 2, insertion cost 3, length fraction 0.8, similarity fraction 0.8, and maximum number of read 1) within the CLC software (Qiagen, Redwood city, CA, USA; https://www.qiagenbioinformatics.com/products/clc-genomics-workbench/). The number of mapped reads was normalized and measured in terms of reads per kilobase per million (FPKM) to determine transcript abundance using Cufflinks.

### Bioinformatic analysis

This experiment investigates the reaction to circadian environmental temperatures in a hot climate zone using two groups of chicken differing for adaptation to high environmental temperatures. Therefore, the first analysis is the direct comparison between the two groups of chicken at each time point.

Each chicken type was investigated at three time points: morning, noon, and evening (see above). Thus, for each chicken type, three analyses were done comparing morning–noon (i.e., the reaction to increasing temperatures), noon–evening (i.e., the reaction to decreasing temperature), and morning–evening (i.e., to investigate whether recovery from hot temperature is complete or that further recovery is needed during night time).

For each analysis, functional annotation analysis and network analyses were done. Because the analysis of the results using the chicken data was unsatisfying, we converted the ENSEMBLE gene numbers to official gene symbols and used the human annotation data. We compared the results obtained with the human annotation always with the results obtained with the chicken annotation. However, here we presented the results obtained with the human annotation. First differentially expressed genes were selected. For each gene, the expression levels were compared between the two groups. These lists of differentially expressed genes were used for functional annotation enrichment analysis using the DAVID software (Huang et al. 2009a, b; https://david.ncifcrf.gov/). Network analyses were done using the online software tools STRING (Szklarczyk et al. 2015, 2017; https://string-db.org/) and STITCH (Kuhn et al. 2014; Szklarczyk et al. 2016; http://stitch.embl.de/). The protein network was investigated with STRING, while the importance of specific metabolites was investigated with STITCH. The higher order pathway organization was investigated using the ClueGo app of Cytoscape (Shannon et al. 2003; Bindea et al. 2009). Groups of pathways were studied using KEGG and BioCarta pathway databases (Kanehisa and Goto, 2000; Kanehisa et al. 2014; http://www.genome.jp/kegg/; Nishimura, 2004; https://cgap.nci.nih.gov/Pathways/BioCarta_Pathways). The cutoff value for a group was arbitrarily set to 3. Several datasets were too large to analyze for the software due to large numbers of differentially expressed genes. To solve this, the software was set to maximum specificity.

## Results

### Differentially expressed genes and related functional annotation enrichment

Supplementary files 1, 2, and 3 provide all RNA-Seq data of all genes after normalization for respectively heart, muscle, and spleen tissues. These were the data used for all analyses. Table 1 showed that the numbers of differentially expressed genes differ among chicken groups and tissues. The difference between highland and lowland chickens seemed largest in muscle tissue. Lowland chickens showed the largest number of differentially expressed genes in heart tissue while highland chicken showed largest number of differentially expressed genes in spleen and muscle tissue. This may indicate differential biological regulatory mechanisms operating in the two chicken groups. Furthermore, all tissues showed reaction to increasing temperatures (i.e., morning–noon) and to decreasing temperatures (i.e., noon–evening). The comparison between gene expression in the morning and the evening indicates that at the end of the day no (full) reversal of gene expression has taken place, suggesting further changes in gene expression patterns during the night. Especially in lowland chicken heart tissue, major differences between morning and evening gene expression profiles were observed. In lowland chicken heart tissue, the reaction during increasing temperatures seemed to be much smaller than during decreasing temperatures. The highland versus lowland chicken comparison suggested a similar mechanism in muscle tissue, which already starts at noon.

**Table 1:** The numbers of differentially expressed genes in three tissues of highland and lowland chicken analyzed at three-time point during the day related to normal lowland circadian environmental temperatures

Comparison	Heart		Muscle		Spleen	
	GG	HS	GG	HS	GG	HS
Highland vs. lowland—all time points	707	570	1900	1561	512	396
Morning	376	319	304	250	364	290
Noon	459	356	914	731	453	356
Evening	287	222	945	766	273	194
Highland—all time points	946	715	1380	1102	2023	1575
Morning–noon	366	274	551	426	965	788
Noon–evening	369	272	467	375	575	415
Morning–evening	372	290	653	536	990	780
Lowland—all time points	2248	1808	1265	1033	1143	869
Morning–noon	297	210	548	455	409	318
Noon–evening	805	659	474	379	316	232
Morning–evening	1612	1319	463	375	571	433

Number of differentially expressed genes as recognized by DAVID for analysis. The chicken (*Gallus gallus*, GG) EMSEMBLE numbers were converted by DAVID into human (*Homo sapiens*, HS) official gene symbols

Table 2 showed the number of significantly enriched functional annotations reported by DAVID. The number of enriched functional annotations using the chicken genome annotation is poor as compared to the human genome annotation data. Therefore, we used the functional annotation of the human genome, although we are aware of potential differences between mammals and birds.

**Table 2:** The numbers of functional annotations enriched using the differentially expressed genes (Table 1) in three tissues of highland and lowland chicken analyzed at three-time point during the day related to normal lowland circadian environmental temperatures

Comparison	Heart		Muscle		Spleen	
	GG	HS	GG	HS	GG	HS
Highland vs. lowland—all time points	0	246	61	897	5	153
Morning	0	162	0	51	0	68
Noon	0	87	14	335	1	143
Evening	0	55	17	340	2	138
Highland—all time points	14	427	23	391	60	1342
Morning–noon	21	359	2	223	67	1229
Noon–evening	1	76	3	119	0	215
Morning–evening	0	38	21	215	11	878
Lowland—all time points	37	823	19	339	6	336
Morning–noon	0	15	22	235	1	133
Noon–evening	12	293	0	148	1	89
Morning–evening	40	834	0	181	7	254

*GG Gallus gallus* (chicken), *HS Homos sapiens* (human)

Heart tissue of lowland chicken showed more functional annotation enrichments in lowland chicken than in highland chicken. Furthermore, lowland chicken showed the highest number of functional annotation enrichments during decreasing temperatures, which was not observed in highland chicken. Spleen tissue showed the opposite pattern with highest number of functional annotations enriched in highland chicken and largest number of enriched functional annotations during the period of increasing daytime temperatures. No differences between the chicken groups were found in muscle tissue. Again, all data indicate a major difference between morning and evening.

### Functional annotations

In this paragraph, we focus on the biological processes underlying the data of Table 2. A summary of the functional annotations can be found in the Supplementary file 4. The data in the file focus on biological and cellular molecular processes and exclude the long lists of protein domains and interactions reported by DAVID but include the cluster analysis of DAVID. Functional annotations describing the same biological functionality were taken together, thus reducing the number of annotations without losing biological information. We describe the data per tissue and focus mainly on general mechanisms.

### Heart tissue

Functional analysis of the differentially expressed genes between highland and lowland chicken indicates a role for mitochondrial processes, mainly related to oxidative energy metabolism, specifically at noon. The annotation was found also in lowland chicken in the afternoon period. In highland chicken during the morning period and in lowland chicken during the afternoon period and the morning–evening comparison, the functional annotation relating to cellular communication pathways (collagen/extracellular matrix/cell-cell adhesion and communication) is found. RNA metabolism, especially splicing, is found in both chicken groups. The chicken groups have differential expression at noon and in the evening. While highland chicken show differential expression during the day, lowland chicken indicate especially a difference between the morning and evening analyses. This indicates that in highland chicken, the expression is regulated during the day and returns to morning levels, but in lowland chicken, the expression between morning and evening remains different. Only in highland chicken during the morning to noon period that differential expression of ribosomal proteins is found. Finally, at several places, heart/muscle annotations are indicated suggesting rebuilding or restructuring of the heart tissue, which may relate to functional adaptations of the heart. Histone (de)acetylation and chromatin methylation were found in both chicken lines.

### Muscle tissue

Analysis of the differentially expressed genes between highland and lowland chicken indicate splicing as an important mechanism in both chicken groups, and in both groups, the expression in the evening did not return to the expression levels in the morning. The highland and lowland chicken groups differ in expression of the proteasome complex, especially during the afternoon period. This difference related mainly to the lowland chickens, which also indicate that expression levels also differ between morning and evening samples. The highland and lowland chicken groups differ also in expression for the cellular communication pathways (collagen/extracellular matrix/cell-cell adhesion and communication), especially during the afternoon period. These effects were mainly found in the highland chickens and not in the lowland chickens. The two chicken group also differed for the expression of mitochondrial genes, which seemed to be regulated only in the highland chicken, which also indicated that evening expression levels differ from morning expression levels. The two chicken groups also differed for acetylation, especially during the afternoon period. Differential expression was observed throughout the day in both chicken groups, indicating that expression remained different between evening and morning expression levels. Finally, at several places, muscle annotations were indicated suggesting rebuilding or restructuring of the muscle tissue, which may relate to functional adaptations of the muscle tissue.

### Spleen tissue

Analysis of the differentially expressed genes between highland and lowland chicken indicates functional annotation enrichment of mitochondrial genes at noon. The expression profiles of the two chicken groups differ: in highland chicken, differentially expressed mitochondrial genes were found during the morning period, while in lowland chicken, functional annotation enrichment of mitochondrial genes was found during the afternoon period. Only in highland chicken the comparison of the expression levels of genes for the evening to morning showed differentially expressed genes. It should be noted that here especially mitochondrial organization was involved, while no oxidative phosphorylation mechanism annotations were reported. Functional annotation enrichment of cellular communication-related pathways (collagen/extracellular matrix/cell-cell adhesion and communication) was found between highland and lowland chickens, especially in the evening. Some indications for differential regulation of these processes were found in highland chicken but not in lowland chicken. These data are supported by regulation of post-translational modification of proteins (Golgi/endoplasmic reticulum/protein transport) data. The two chicken groups differed especially in the morning, while specific regulation was found throughout the day in both chicken groups. Especially in lowland chicken, regulation of alternative splicing was found throughout the day. The two chicken groups differed in the expression of acetylation-related genes at noon. However, both groups showed differential expression of acetylation-related genes throughout the day, with some indication of histone-specific acetylation/deacetylation. Finally, enrichment of cell division was observed in both chicken groups in the morning but not in the afternoon.

#### Network analyses

Three different network analyses were done: a protein network analysis, a protein network analysis including metabolites, and an integration of pathway analyses. Supplementary file 5 provides an overview of the results in the three tissues. All network analysis figures are provided in Supplementary file 6. For the protein networks, we focused on the hub proteins defined as highly connected proteins as compared with other proteins in the network. Hub proteins were analyzed for functional annotation, and the functional annotations of the hub proteins are displayed in Table 3. Furthermore, we observed in several networks specific nuclei, defined as groups of proteins connected with each other but with less connections to other proteins. The proteins of the nuclei were analyzed for functional annotation—it proved to be that the proteins within a nucleus had similar functional annotation. Several of the network analyses were hampered by the creation of very dense networks, too-large-to-analyze. It should be noted that from these networks, data may be missing or incomplete.

**Table 3:** Functional annotations of network hub genes and nuclei created with STRING and STITCH software tools comparing Ethiopian highland and lowland chicken at three different time points during the day: in the morning, at noon, and in the evening measured under lowland conditions

Analysis group	Functional annotation protein hubs and nuclei in the networks, relevant metabolites		
	Heart	Muscle	Spleen
Highland vs. lowland—all time points	*Mitochondrion/Energy metabolism*	Network too dense to analyze	*Mitochondrion/energy metabolism*
	*Mitosis*		*Mitosis/apoptosis*
	*Histone methylation*		*Histone methylation*
			*Circadian rhythm*
			Ribosome
	*Circadian Rhythm*		General metabolism
	Collagen		Immune mechanisms
	Histone deacetylation		Cell adhesion
			Cellular components
	*Calcium*	Saturated fatty acids	*Calcium*
			Dopamine
Morning	*Mitosis/apoptosis*	*Mitosis/apoptosis*	*Mitosis/apoptosis*
	*Circadian rhythm*	*Circadian rhythm*	*Histone deacetylation/methylation*
	*Histone deacetylation*	Immune	
	Transport	Angiogenesis	Protein transport
	Insulin receptor mechanism	Ribosome/tRNA synthesis	Ribosome
			Transcription factor expression
	Immune		
			Protein metabolism
			Signal transduction
Noon	*Angiogenesis*	*Angiogenesis*	*Angiogenesis*
	*Mitosis/apoptosis*	*Mitosis/apoptosis*	*Mitosis/apoptosis*
	*Ribosome*	*Ribosome*	*Ribosome*
	*Immune-related*	*Immune-related*	*Immune*
		*Stress response*	*Mitochondrion/energy metabolism*
	*Mitochondrion/energy*	*Splicing*	
		*Protein transport*	*Stress response*
	*metabolism*	Collagen/ECM	*Chaperone/protein folding*
			*Splicing*
	*Molecular chaperone*		*Protein transport/metabolism*
		Calcium	
Evening	*Mitosis*	*Mitosis*	*Mitosis/stress/apoptosis*
	Angiogenesis	*Mitochondrion*	*Mitochondrion*
	Splicing	Deacetylation	Cellular components/ECM
	Ribosome	Immune	
	Stress fibers	Signaling	
		Iron/calcium	
Highland—all time points	Mitosis/apoptosis	Network too dense to analyze	Network too dense to analyze
	Ribosome		
	Splicing		
	Angiogenesis		
	Heart muscle		
	components		
	Molecular chaperone		
	Stress/immune circadian rhythm		
Morning–noon	*Circadian rhythm*	*Circadian rhythm*	*Molecular chaperone*
	*Ribosome*	*Ribosome*	Protein metabolism
	*Immune*	*Immune*	*Mitosis/apoptosis*
	*Histone deacetylase*	*Histone deacetylase*	
	Transcriptional silencing	*Molecular chaperone* (cell and mitochondrion)	
	*Mitosis/apoptosis*		
	*Molecular chaperone*	Splicing (stress response?)	
		Cell adhesion/communication/ECM	
			Calcium
Noon–evening	*Mitosis*	*Mitosis/apoptosis*	*Mitosis/apoptosis*
	Histone	*(De)acetylation/methylation*	*(De)acetylation*
	*Mitochondrion*		*Energy metabolism*
	Heart functioning	*Splicing*	*Splicing/mRNA metabolism*
		*Molecular chaperone*	
		*Immune*	*Molecular chaperone*
		Ribosome	*Immune processes*
			Protein metabolism/transport
			Transcription factor expression
			Angiogenesis
			Cell adhesion
			Signal transduction
	Dopamine		
Morning–evening	*Splicing/RNA processing*	*Splicing*	*Mitochondrion*
		*Mitochondrion/energy*	*Ribosome*
	*Mitosis*	*Metabolism*	Angiogenesis/lymphatic vasculature development
	*Ribosome*	*Mitosis*	
	Chaperone	(De)acetylation/methylation	Chromatin remodeling
	Angiogenesis		
	Gap junction (synchronized contraction of the heart)	Adherence/ECM	
	Dopamine	Iron	
		Organosulfur	
Lowland—all time points	Deacetylase	Ribosome	Network too dense to analyze
	Saturated fatty acids		
	Cardiovascular disease associated		
Morning–noon	*Mitosis*	*Mitosis/apoptosis*	*Mitosis/apoptosis*
	*Angiogenesis*	*Splicing*	*Angiogenesis*
	Mitochondrion	Ribosome	*Splicing*
		Acetylation/methylation/(telomerase?)	Integration of energy metabolism and metabolism
		Expression regulation	Cholesterol metabolism
		Adipose tissue development	Stress-activated
			Circadian rhythm/clock
			Immune
			Chaperone (heat shock induction)
			Calcium
Noon–evening	*Mitosis/apoptosis*	*Mitosis/apoptosis*	*Down regulation of apoptosis*
	*Mitochondrion*	*Ribosome (synthesis)*	
	*Fatty acid biosynthesis*	Angiogenesis	*Energy metabolism*
	*Circadian rhythm*	Protein transport	*Fatty acid biosynthesis*
	Immune		*Circadian rhythm*
			*Ribosome*
			Splicing
			Transcription factors
Morning–evening	*Mitochondrion*	*Energy metabolism*	*Mitochondrion/energy metabolism/cholesterol metabolism*
	*Fatty acid beta-oxidation*	*Mitosis/apoptosis*	
		*Stress response*	
	*Mitosis/apoptosis*	*Splicing*	*Mitosis/apoptosis (less)*
	*Stress response (high blood pressure)*	*Ribosome*	*Splicing*
		Immune	*Ribosome*
		Transport of proteins	Angiogenesis
		Circadian rhythm	
		General metabolism	
		Cell adhesion	

Heart, (breast) muscle, and spleen tissues were analyzed. Functional annotations shared among two or three tissues are shown in italics. Metabolites appearing as central regulators in the networks are shown apart. Please note that several networks were too large to analyze due to large numbers of differentially expressed genes. These data are either missing or incomplete

Table 3 shows a summary of the functional annotation enrichments of the protein hubs and nuclei of the networks of the three tissues at three different times of the day. The table shows an extraction of the data that can be found in detail in the Supplementary files 2 and 3. The table shows that several general functional mechanisms are shared by the tissues: energy metabolism, (de)acetylation, mitosis/apoptosis, immune functions, circadian rhythm, (molecular) chaperone, stress, and (alternative) splicing. Double annotations suggest that stress and splicing and stress (molecular), chaperone, and mitosis/apoptosis are related to each other. Furthermore, several metabolites were found to be related to functional mechanisms of differentially expressed genes. Among these, calcium and dopamine are shared among the tissues.

### Groups of pathways related to heat stress changes throughout the day

Table 4 shows the results of the Cytoscape-ClueGo analysis of groups of (physiological) pathways harboring differently expressed genes in three tissues: heart (A), (breast) muscle (B), and spleen (C). In heart tissue, the main remarkable result is that lowland chicken seems to respond more than the highland chicken. The lowland chicken shows differential expression in positive regulation of several metabolic pathways. Despite this, there is a long list of groups of pathways differing between morning and evening, indicating that the heat-related changes were not reversed yet at the end of the day during the afternoon. Among them, negative regulation of histone H3-K4 methylation indicates potential increased gene expression levels. In the afternoon, a relation with the gut is suggested via the vagus nerve. The circadian rhythm is changed in highland chicken during the day and still changed at the end of the day. Major differences between the highland chicken and the lowland chicken also include cardiac and skeletal muscle developmental effects, fat cell differentiation and lipid storage, and cell division. There seems to be a relation with immune-related processes and auditory signal responses (especially in the highland chicken).

**Table 4.** Analysis of groups of physiological pathways comparing Ethiopian highland and lowland chicken at three different time points during the day under the lowland environmental conditions: in the morning, at noon, and in the evening of heart (A), (breast) muscle (B), and spleen (C) tissues

A. Heart	
Animal groups	Major pathways–groups of pathways
Highland vs. lowland—all time points	ATP synthesis coupled transport; coenzyme biosynthetic process; cellular carbohydrate catabolic process–positive regulation of B cell activation; positive regulation of proteasomal protein catabolic process–cGMP-PKG signaling pathway–negative regulation of muscle contraction; negative regulation of muscle hypertrophy; myoblast differentiation–regulation of phospholipase C activity; cognition–Rap1 signaling; B cell receptor signaling pathway; chronic myeloid leukemia; cytokine production involved in immune response/regulation of fat cell differentiation; positive regulation of fat cell differentiation; asymmetric cell division/inner ear receptor cell development; establishment of planar polarity/DNA strand elongation/neutral lipid process; glycerolipid metabolism/microtubule-based movement; cilium movement/sulfur amino acid metabolic process/glycosphigolipid metabolic process/collagen fibril organization/mitochondrial translation initiation/lipid storage/negative regulation of transmembrane transport/**Rho GTPase cycle/regulation of RNA stability/negative regulation of G protein-coupled receptor protein signaling pathway**
Morning	Associative learning/sister chromatid cohesion/epithelium cilium movement involved in determination left-right asymmetry/cardiac muscle cell development
Noon	Toll-like receptor TLR1-TLR2 signaling pathway; lymphocyte homeostasis; positive regulation of lipid metabolic process/regulation of membrane repolarization/positive regulation of cAMP biosynthetic process/heart looping/cell cycle DNA replication/**trans-Golgi network vesicle budding/protein targeting to vacuole/GPI anchor biosynthetic process**
Evening	Processing of capped intronless pre-mRNA/**activation of matrix metalloproteinases**/assembly of collagen fibrils and other multimeric structures/**epithelial cell morphogenesis**
Highland*—all time points	mRNA catabolic process
Morning– noon	mRNA processing/nuclear transcribed mRNA catabolic process exonucleolytic; cell-cell junction organization; **regulation of systemic arterial blood pressure**; **oxygen transport**
Noon–evening	Response to auditory stimulus/regulation of adaptive immune response based on somatic recombination of immune receptors built from immunoglobulin superfamily domains/RHO GTPases activate formins/regulation of potassium transport/**regulation of transcription from RNA polymerase II promoter/lens morphogenesis in camera-type eye/adherens junction/cellular response to glucose starvation**
Morning–evening	Unfolded protein response (UPR); RNA surveillance/cell-cell junction assembly/negative regulation of circadian rhythm
Lowland*—all time points	Positive regulation of metabolic process; positive regulation of molecular function; protein modification process/organelle organization/**cell cycle**
Morning–noon	Coenzyme biosynthetic process/arachidonic acid metabolism/**mesonephric tubule morphogenesis/regulation of phospholipase C activity/cellular component disassembly involved in execution phase of apoptosis**
Noon–evening*	Regulation of early endosome to recycling endosome transport/3′-UTR-mediated mRNA destabilization/coenzyme A catabolic process/optic placode formation involved in camera-type eye formation/amniotic stem cell differentiation/vagus nerve morphogenesis/l-ascorbic acid biosynthetic process/**negative regulation of cellular amino acid metabolic processes**/**signal transduction involved in intra-S DNA damage checkpoint/nuclear mRNA surveillance of mRNA 3′-end processing/negative regulation of interleukin-6-mediated signaling pathway**
Morning–evening*	Regulation of interleukin-4 biosynthetic process; interleukin-4 biosynthetic process; primitive erythrocyte differentiation–negative regulation of histone H3-K4 methylation; negative regulation of centrosome duplication–skeletal muscle thin filament assembly/negative regulation of filopodium assembly/activation of meiosis involved in egg activation/regulation of DNA-templated transcription termination/3′-UTR-mediated mRNA destabilization/lateral mesoderm cell fate commitment/geranyl diphosphate metabolic process/coenzyme A catabolic process/positive regulation of sequestering of calcium ion/amniotic stem cell differentiation/regulation of protein tetramerization/coenzyme A transport/heme A biosynthetic process/**negative regulation of uterine smooth muscle contraction/protein processing in phagocytic vesicle/positive regulation of protein deubiquitination/histone H3-K27 acetylation/membrane depolarization during AV node cell action potential/diadenosine polyphosphate metabolic process/transcription initiation from RNA polymerase I promoter large rRNA transcript**
B. Muscle	
Animal groups	Major pathways–groups of pathways
Highland vs. lowland*—all time points	Beta-alanine biosynthetic process; negative regulation of vascular endothelial growth factor signaling pathway/cell migration involved in metanephros development; medium chain fatty acid transport; phosphatidylserine biosynthetic process/regulation of centromeric sister chromatid cohesion/VEGF-activated neuropilin signaling pathway/regulation of membrane depolarization during cardiac muscle cell action potential/acetate metabolic process/regulation of sulfur amino acid metabolic process/regulation of telomere maintenance via semi-conservative replication/dipeptide membrane transport/activation of meiosis involved in egg activation/positive regulation of centrosome duplication/cervix development/glycosyl ceramide biosynthetic process/negative regulation of skeletal muscle cell differentiation/negative regulation of eosinophil activation/N-terminal protein palmitoylation/lysine import/coenzyme A transport/positive regulation of TRAIL production/**Wnt signaling pathway involved in digestive tract morphogenesis/UDP-*N*-acetyl glucose transport/regulation of intrinsic apoptotic signaling pathway in response to osmotic stress by p53 class mediator/negative regulation of glucocorticoid-mediated signaling pathway/cytoplasmic translational elongation/elastin biosynthetic process/transcription initiation from RNA polymerase I promoter for nuclear large rRNA transcript**
Morning*	Negative regulation of proteasome ubiquitin-dependent protein catabolic process/glycerophospholipid biosynthesis/cellular carbohydrate catabolic process/protein O-linked glycosylation
Noon*	Negative regulation of filopodium assembly/dipeptide transmembrane transport/cervix development/induction by symbiont of host defense response/endosome to lysosome transport via multivesicular body sorting pathway/regulation of microglial cell activation/regulation of protein import into mitochondrial outer membrane/coronary vein morphogenesis/negative regulation of nucleobase-containing compound transport/regulation of neutrophil apoptotic process/regulation of natural killer cell cytokine production/succinate transport
Evening*	Mitochondrial RNA 3′-end processing/limb joint morphogenesis/N-terminal amino acid methylation/reversal of alkylation damage by DNA dioxygenases/lysine import/coronary morphogenesis/positive regulation of TRAIL production/positive regulation of Schwann cell differentiation/**atrial ventricular junction remodeling/TIRAP-dependent toll-like receptor signaling pathway/negative regulation of phosphatidylinositol biosynthetic process/regulation of intrinsic apoptotic signaling pathway response to osmotic stress by p53 class mediator/CVT pathway/negative regulation of glucocorticoid-mediated signaling pathway/elastin biosynthetic process/acetate biosynthetic process/transcription initiation from RNA polymerase I promoter for nuclear large rRNA transcript**
Highland*—all time points	Canonical Wnt signaling pathway involved in positive regulation of epithelial to mesenchymal transition/induction by symbiont of host defense response/regulation of DNA-templated transcription termination/mitochondrial RNA surveillance/.../vagus nerve morphogenesis
Morning–noon	Blood vessel endothelial cell migration; positive regulation of cellular carbohydrate metabolic process–signaling by NOTCH1; primary neural tube formation; hormone biosynthetic process; retinol metabolic process–protein deacetylation; alpha linolenic acid (ALA) metabolism/regulation of interleukin 12 biosynthetic process; interleukin 12 biosynthetic process/GABA synthesis, release, reuptake, and degradation/dolichol-linked oligosaccharide biosynthetic process/protein homotetramerization/DNA-templated transcription termination/histone H4 acetylation/**IRE1-mediated unfolded protein response/termination of G protein-coupled receptor signaling pathway**
Noon–evening	Positive regulation of viral transcription/proteasome maturation/regulation of stress fiber assembly/negative regulation of G protein-coupled receptor protein signaling pathway/vitamin transport/organophosphate ester transport/rRNA processing
Morning–evening	Retrograde neurotrophin signaling; DNA-templated transcription elongation; transcription elongation from RNA polymerase II promoter; Ca^2+^ pathway–regulation of epithelial cell differentiation–morphogenesis of embryonic epithelium–regulation of erythrocyte differentiation; positive regulation of peptide hormone secretion; unsaturated fatty acid biosynthetic process; PPAR signaling pathway–mitotic spindle organization; positive regulation of proteasomal ubiquitin-dependent protein catabolic process; negative regulation of proteasomal protein catabolic process
Lowland*—all time points	Positive regulation of branching involved in lung morphogenesis/negative regulation of sequestering of triglyceride/histone H3-K36 trimethylation/positive regulation of centrosome duplication/regulation of blood vessel endothelial cell proliferation involved in sprouting angiogenesis/induction by symbiont of host defense response/regulation of DNA-templated transcription termination/marginal zone B cell differentiation/coronary vein morphogenesis/negative regulation of nucleobase-containing compound transport/regulation of neutrophil apoptotic process/cadmium ion homeostasis/Golgi to plasma membrane CFTR protein transport/l-ascorbic acid biosynthetic process/**xanthine metabolic process/CVT pathway/negative regulation of phospholipase C activity/succinate transport/negative regulation of intestinal phytosterol absorption/transcription initiation from RNA polymerase I promoter for nuclear large rRNA transcript**
Morning–noon	Photoperiodism; PPARA activates gene expression; glycerolipid metabolism/ribonucleoprotein complex biogenesis/regulation of double-strand break repair via homologous recombination/signal transduction involved in cell cycle checkpoint/eye morphogenesis/cellular response to mechanical stimulus/response to vitamin/transcription elongation from RNA polymerase I promoter/histone methylation/positive regulation of stress-activated MAPK cascade/recruitment of mitotic centrosome proteins and complexes/regulation of mRNA stability/positive regulation of leukocyte chemotaxis/**pre-NOTCH expression and processing/cellular monovalent inorganic cation homeostasis**
Noon–evening	Male gonad development; regulation of response to extracellular stimulus–blood vessel endothelial cell proliferation involved in sprouting angiogenesis–negative regulation of DNA biosynthetic process; positive regulation of stem cell proliferation; negative regulation of nuclear division (with muscle pathways!); neural tube patterning/positive regulation of axon extension/**glycolysis + gluconeogenesis/SUMOylation of DNA damage response and repair proteins/response to ionizing radiation**
Morning–evening	Glutamate receptor signaling pathway; oxytocin signaling pathway–adipocytokine signaling pathway–negative regulation of mononuclear cell proliferation; regulation of mesenchymal cell proliferation; mesenchymal cell proliferation–TGF-beta signaling pathway; embryonic digit morphogenesis; osteoblast proliferation–shigellosis/DNA-templated transcription termination; mRNA3′-end processing/response to ammonium ion/NRAGE signals death through JNK/glycerolipid metabolism/centrosome cycle/**mitotic G1-G1S phases/regulation of plasma membrane organization**
C. Spleen	
Animal groups	Major pathways–groups of pathways
Highland vs. lowland—all time points	Detection of mechanical stimulus involved in sensory perception of sound; response to mechanical stimulus; neuroactive ligand-receptor interaction; associative learning–monovalent inorganic cation homeostasis; hormone biosynthetic process/lymphocyte homeostasis/response to acid chemical/entry into host cell/positive regulation of Rho protein signal transduction
Morning	Maintenance of protein location in cell; regulation of chromosomal segregation; anaphase-promoting complex-dependent proteasomal ubiquitin-dependent protein catabolic process; E2F-mediated regulation of DNA replication/protein processing in endoplasmic reticulum/**regulation of fibroblast proliferation**
Noon	Organelle biosynthesis and maintenance; chromosome segregation–anchoring of the basal body to the plasma membrane/negative regulation of protein localization to nucleus/**trans-Golgi network vesicle budding/mitotic spindle organization/regulation of mRNA splicing, via spliceosome**
Evening	...Too small to mention any...
Highland*—all time points	Regulation of glycolytic process by regulation of transcription from RNA polymerase II; regulation of platelet-derived growth factor receptor-alpha signaling pathway/interleukin 4 biosynthetic process/regulation of phosphatidylcholine biosynthetic process/maintenance of mitotic sister chromatid cohesion/positive regulation of ER to Golgi vesicle-mediated transport/protein localization to nonmotile primary cilium/tongue muscle cell differentiation/negative regulation of eosinophil activation/positive regulation of oocyte development/dosage compensation by inactivation of X chromosome/spermidiane catabolic process/positive regulation of aldosterone metabolism/mitochondrial mRNA catabolic process/regulation of protein import into mitochondrial outer membrane/positive regulation of chromatin silencing/cerebellar cortex structural organization/**proline catabolism/protein localization to vacuolar membrane/synaptic vesicle uncoating/response to biotin/receptor-mediated virion attachment to host cell/dolichol metabolic process**
Morning–noon	PI metabolism; phosphatidylinositol dephosphorylation–steroid hormone secretion; C21 steroid hormone biosynthetic process–regulation of receptor biosynthetic process–pre-NOTCH expression and processing; signaling by NOTCH–ARMS-mediated activation; Golgi cisternae pericentriolar stack reorganization; condensation of prometaphase chromosomes; nuclear envelope breakdown; M phase; mRNA transport; transcription organization from RNA polymerase III promoter; cytosolic sensors of pathogen-associated DNA; TRAF3-depedent IRF activation pathway; BBsome-mediated cargo-targeting to cilium; folding of actin by CCT/TriC; mitotic prometaphase (close to M phase); RHO GTPases activate formin; mitotic sister chromatic segregation; cell cycle; cell cycle checkpoints; regulation of spindle organization; histone monoubiquitination; histone H3 acetylation; peptidyl-lysine methylation; regulation of PLK1 activity at G2/M transition; regulation of protein phosphatase type 2A activity/anterograde axon cargo transport/thromboxane signaling through TP receptor/cochlea morphogenesis/methionine metabolic process/mitochondrial translation termination/cytoplasmic mRNA processing body assembly/COPII-coated vesicle budding/RNA destabilization/**negative regulation of DNA recombination/protein K63-linked deubiquitination/C-protein coupled purinergic nucleotide receptor signaling pathway**
Noon–evening	Interleukin 4 signaling; prolactin signaling pathway/NOTCH intracellular domain regulates transcription/protein localization to microtubule cytoskeleton/**postreplication repair/trans-Golgi network vesicle budding/NLS-bearing protein import into nucleus/positive regulation of chromatin modification/cytoskeleton-dependent intracellular transport**
Morning–evening*	SHC-related events triggered by IGF-1R; regulation of anoikis/nucleotide excision repair/regulation of water loss via skin; conversion from APC/C:Cdc20 to APC/C:Cdh1 in late anaphase; APC-Cdc20-mediated degradation of NEK2A/cerebral cortex neuron differentiation; regulation of phosphatidylcholine biosynthetic process/mitotic chromosome condensation/nucleotide-sugar metabolic process/protein export/transport of the SLBP-independent mature mRNA/terpenoid backbone biosynthesis/negative regulation of protein dephosphorylation/phospholipase C-activating dopamine receptor signaling pathway/positive regulation of RNA splicing/*S*-adenosylmethionine cycle/Huntington’s disease/CDP-diacylglycerol biosynthetic process/ER to Golgi vesicle-mediated transport/mitochondrial ATP synthesis coupled proton transport/CBP1(S) activates chaperone genes/SNARE complex assembly/**mitotic prometaphase/regulation of hypoxia-inducible factor (HIF) by oxygen/aorta morphogenesis/centriole replication**
Lowland*—all time points	Negative regulation of osteoclast differentiation; copper ion homeostasis; microtubule cytoskeleton organization involved in mitosis; regulation of centriole elongation; heart formation; spermatic nucleus differentiation; autophagic vacuole assembly; CVT pathway–subpallium development; anterograde axon cargo transport; cochlea morphogenesis; DNA hypermethylation; positive regulation of transcription from RNA polymerase II promoter in response to stress; sphingolipid catabolic process–positive regulation of peptidyl-threonine phosphorylation; mitochondrial biosynthesis; pre-NOTCH expression and processing; retrograde transport, endosome to Golgi–FOXO signaling pathway; oocyte maturation/circadian regulation of translation/transcription from RNA polymerase I promoter/protein refolding/vitamin digestion and absorption/cellular response to heat stress; mitochondrial translation initiation/regulation of T helper 1 type immune response/regulation of fatty acid beta-oxidation/regulation of intracellular steroid hormone receptor signaling pathway/cholesterol biosynthesis/glycosylphosphatidylinositol (GPI)-anchor biosynthesis/energy coupled proton transmembrane transport, against electrochemical gradient/positive regulation of signal transduction by P53 class mediator/**metabolism of porphyrins/SUMOyl DNA damage response and repair process/phosphatidylethanol metabolic process/negative regulation of insulin secretion/embryonic placenta morphogenesis/G protein-coupled purinergic receptor signaling pathway/positive regulation of receptor internalization/synaptic vesicle fusion to presynaptic membrane**
Morning–noon	Interleukin-17 production; regulation of interleukin-17 production/mitotic metaphase plate congression/cholesterol biosynthesis/fructose and mannose metabolism/**neutral lipid biosynthetic process**
Noon–evening	Mitochondrial translation/**nucleoside triphosphate biosynthetic process**
Morning–evening*	Regulation of centrosome cycle; cytoplasmic microtubule organization; regulation of proteasomal ubiquitin-dependent protein catabolic process; PI3K events in ERBB3 signaling; PI3K events in ERBB4 signaling; antigen activates B cell receptor (BCR) leading to generation of second messengers; prostate cancer; negative regulation of cellular response to oxidative stress; Synthesis of PE–AKT phosphorylates targets in the nucleus; negative regulation of smooth muscle differentiation–organelle transport along microtubule/ER to Golgi vesicle-mediated transport/protein localization to kinetochore/regulation of intracellular steroid hormone receptor signaling pathway; GPI anchor metabolic process/mismatch repair/neural tube closure

All groups larger than two pathways are shown, with groups containing three pathways shown in bold. Please note that several datasets were too large to analyze due to large numbers of differentially expressed genes. To solve this, the software was set to maximum specificity, leading to less results. These analyses are marked with an asterisk

Muscle tissue shows several main themes in both chicken lines, including (1) histone modifications, especially during the morning; (2) blood vessel development, in highland chicken mainly during the morning and in lowland chicken mainly during the afternoon; (3) cell division, at the end of the day the situation still differs from the beginning of the day; (4) oxidative stress, in highland chicken especially during the morning; (5) apoptosis throughout the day; (6) response to stress, throughout the day, and in both chicken lines the evening situation still differs from the morning situation; (7) nerve activity, in the lowland chicken mainly during the morning, but the situation at the end of the day still differs from the beginning of the day; and (8) immune response changes. The two chicken lines also differed for muscle differentiation processes and muscle activity. Especially, lowland chicken had a difference in photoperiodism in the morning.

Spleen tissue showed very few differences between the two chicken lines, and in the evening, and they seemed to be similar. Some differences may include regulation of cell division, learning, protein localization at noon, and ear development and functioning. Despite of this, the analysis of both chicken lines individually indicated more differences. Highland chickens showed a wide array of biological processes during the day, with the main focus of regulated processes during the morning. This includes the biological processes leading to apoptosis, histone H3 acetylation, inner ear development, and cell-cell contacts and cell cycle checkpoints. At the end of the day, the processes of apoptosis and cell division were still different from the morning expression levels. Other enriched biological mechanisms were energy metabolism and angiogenesis—which may be related to water loss, blood pressure, cardiovascular disease, and IL4 biosynthesis groups of pathways. Contrarily, the lowland chicken line showed regulation of the proinflammatory IL17 production specifically during the morning. Lowland chickens also showed regulation of energy metabolism more widely via fatty acid, cholesterol, and energy metabolism. In general, they showed regulation of the groups of pathways of circadian rhythm and cellular response to heat and stress in general and oxidative stress specifically—which is still regulated in the evening. These all may be related to DNA hypermethylation.

### Heat stress proteins related to heat stress—differences between chickens and tissues during the day

We investigated the differential expression of the heat shock proteins (HSPs) because these are especially related to the physiological reaction to these specific environmental conditions. We observed differences among the tissues over the day. In heart tissue, we found that the two chickens differed in expression of HSPs especially during the afternoon. Functionally, these HSPs are involved in mitochondrial electron transport and others function as molecular chaperones in the nucleus, regulating cell cycle control and signal transduction. The lowland and highland chickens differ in that the mitochondrial HSP is regulated in lowland but not in highland chicken. On the contrary, in highland chicken, a HSP transcription factor and cell cycle control and signal transduction regulating HSP were regulated, which were not regulated in lowland chicken. However, the chickens share regulation of HSP functioning as a molecular chaperone for correct folding of proteins and controlling protein transport in the ER. Calcium availability is important for this.

The regulated HSP gene profiles in the muscle tissue were different. First of all, the two chickens showed different HSP regulation throughout the day, especially for HSPs functioning as transcription regulators, cell cycle control, and signal transduction and especially for HSPs recruited to the nucleus under heat stress conditions. Furthermore, in the afternoon, the chickens differ for regulation of glucocorticoid and mitochondrial electron transport signaling. At the end of the day, a muscle-specific HSP is differently expressed involved in regulation of actin polymerization. In both chickens, no HSPs regulated during the afternoon. Finally, it is remarkable that there were many more HSPs regulated in highland chickens than in lowland chickens. The focus in lowland chicken is on regulation of heat shock transcriptions factors during the morning and at the end of the day. In highland chickens, this was also found during the morning, but throughout the day, environmental stress-regulated HSPs were found functioning as chaperones and regulating the cellular response to heat and protecting from apoptosis. The muscle-specific HSP was found regulated at the end of the day.

Spleen tissue showed only differences between the two chickens in the afternoon, where the two chickens differed for HSPs regulating mitochondrial electron transport and cellular molecular chaperones described especially in the cellular response to heat stress. However, when investigating the two chickens individually, they showed different profiles. While lowland chickens showed regulated HSPs throughout the day, highland chickens did not have regulated HSPs in the afternoon. Lowland chickens regulated HSPs functioning for glucocorticoid signaling and molecular chaperones especially protecting against aggregated proteins. Highland chickens especially regulated HSPs during the morning. These HSPs especially regulated functions related to signaling to the innate immune system and controlling correct toll-like receptor folding in the endoplasmic reticulum. A relation with the melanosome was also suggested.

### Visualization of circadian expression profiles in three tissues of highland and lowland chicken

#### Muscle cell integrity and functioning

**Fig. 1:**
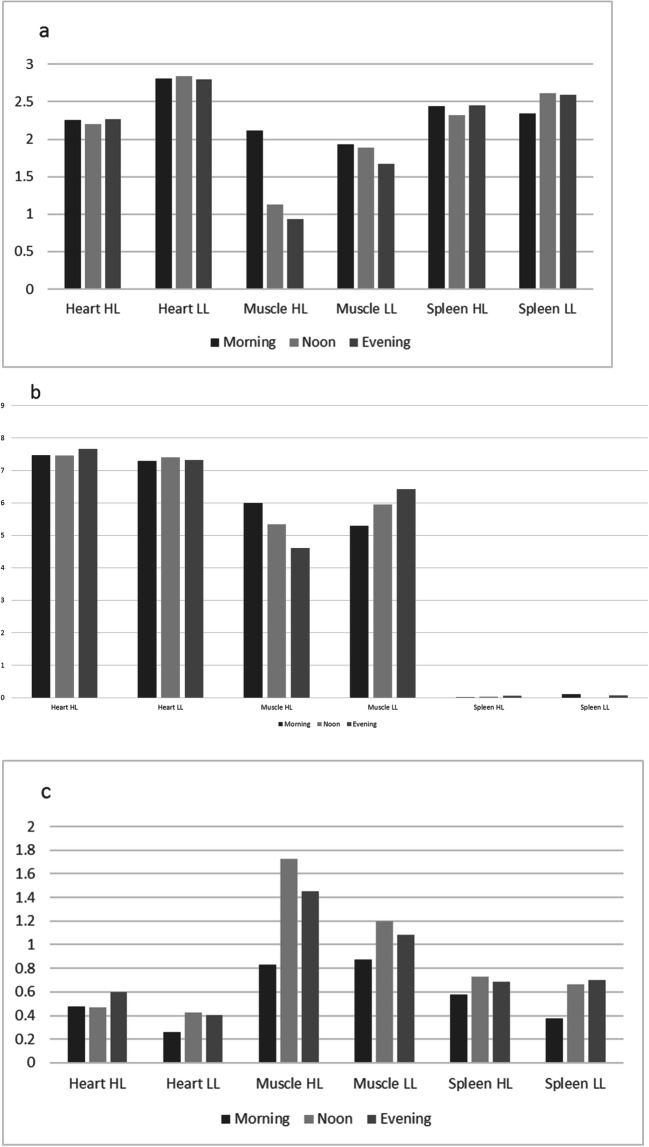

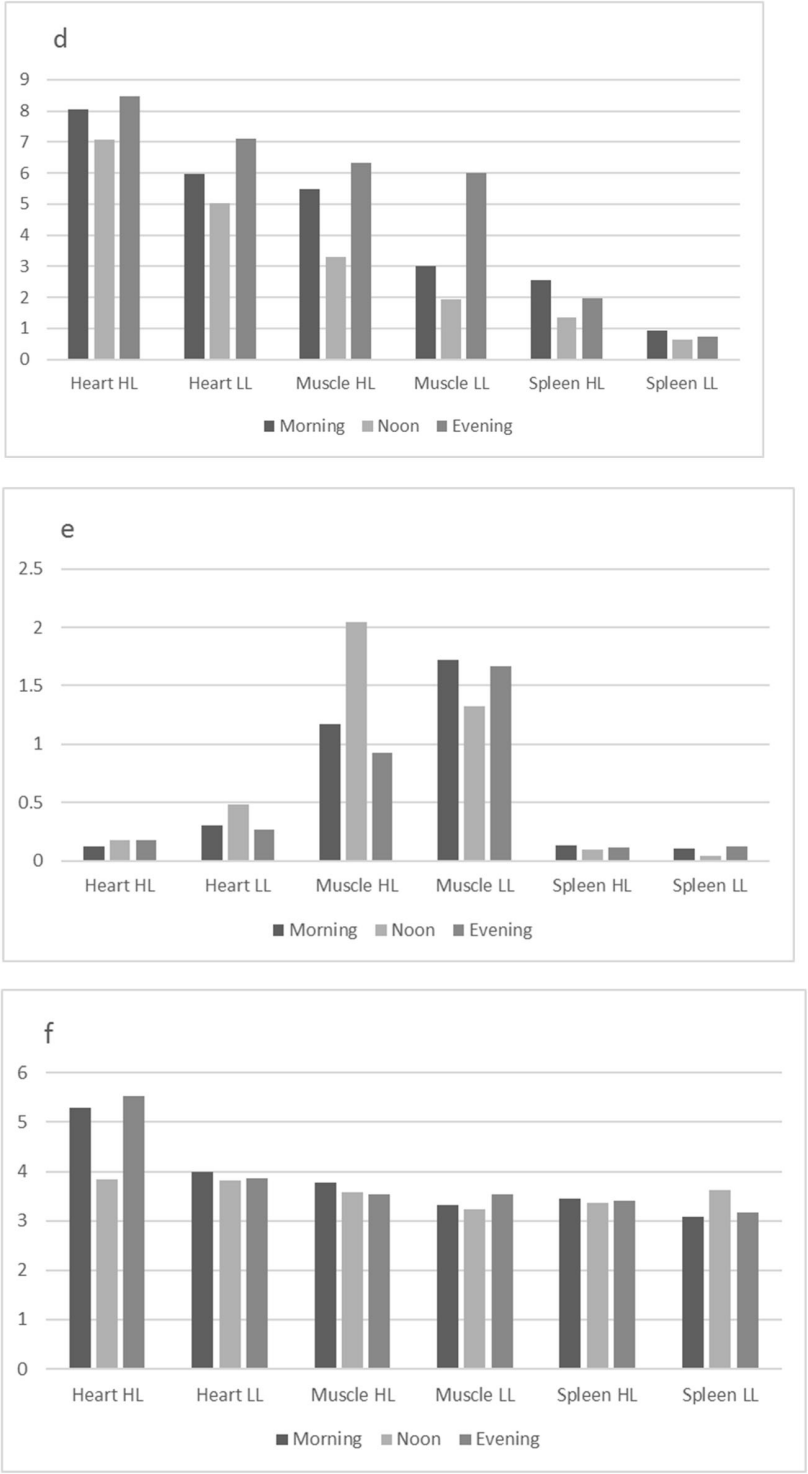

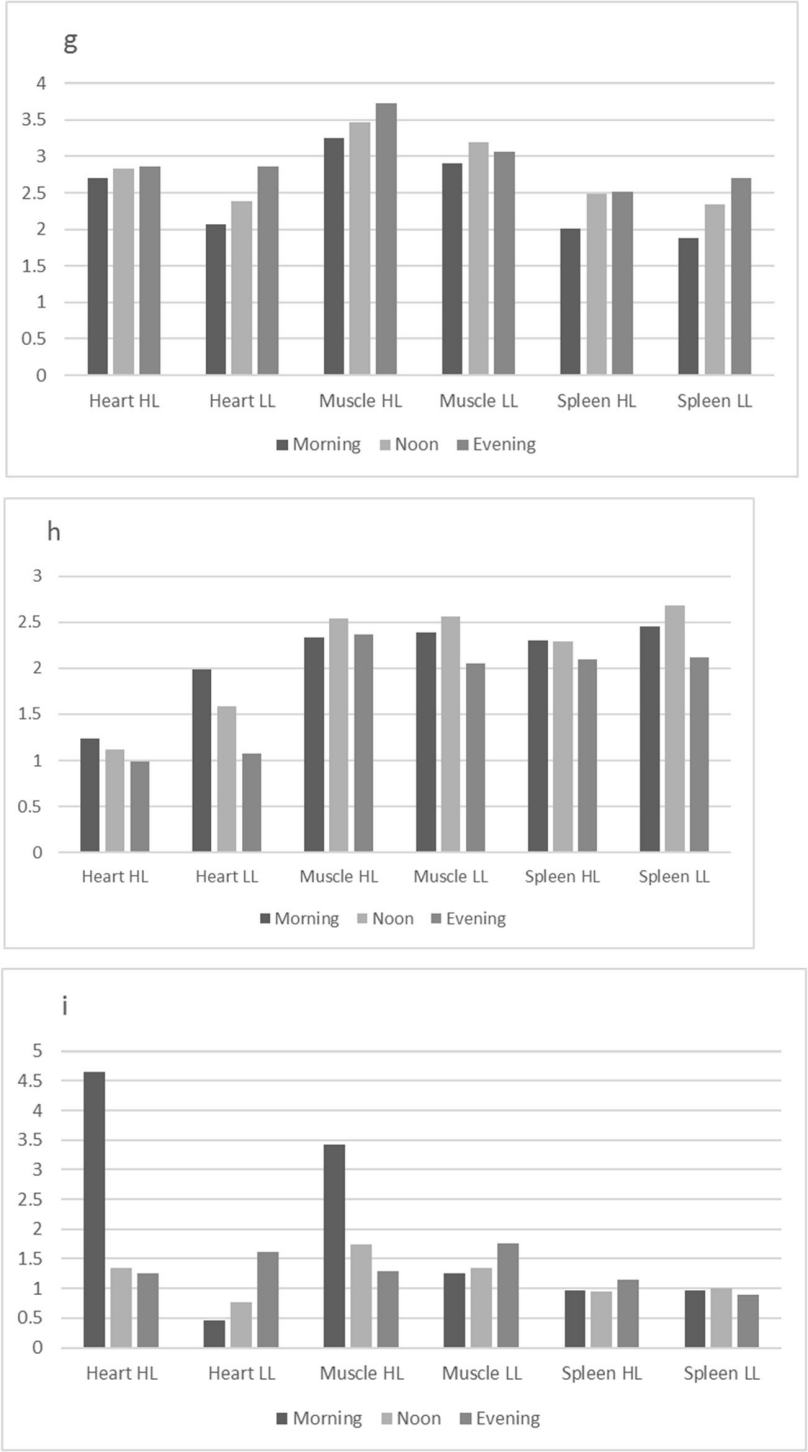

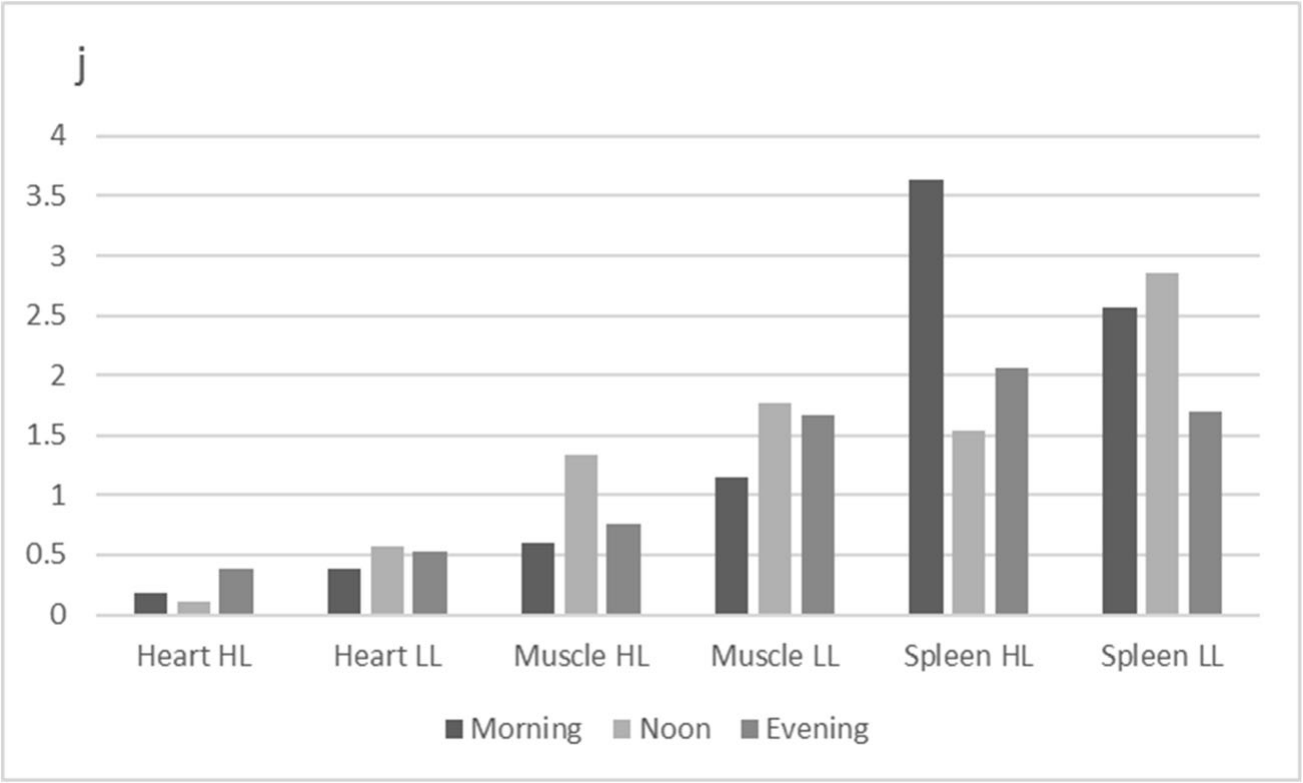
Visualization of circadian expression profiles in three tissues of highland and lowland chicken. **a** Collagen type V alpha 2 chain (Col5A2). **b** Heat shock protein B3 (HSPB3). **c** RNA binding motif protein 15 (RBM15). **d** Pyruvate dehydrogenase kinase, isoenzyme 4 (PDK4). **e** Calcium binding protein 1 (CABP1). **f** DnaJ heat shock protein family (Hsp40) member C12 (DNAJC12). **g** ETS2 repressor factor (ERF). **h** Inhibitor of growth family member 2 (ING2). **i** Nuclear receptor subfamily 4 group A member 3 (NR4A3). **j** Biliverdin reductase A (BLVRA)

The expression profiles of several proteins suggest that the integrity of the muscle tissue may be affected during circadian heat. The expression profiles of the serpin peptidase inhibitor (Serpine, Clade E) gene involved in cellular adhesion and extracellular remodeling, the collagen type V alpha 2 chain and collagen type VI alpha 3 chain (Col5A2 and Col6A3, respectively) gene involved in signaling and focal adhesion, and the dermatopontin (DPT) gene involved in cell-matrix interactions and matrix assembly all follow the same expression pattern showing that the expression in the muscle tissue is decreased during the day in highland chickens but not in lowland chickens. The consequence may be that in highland chickens the extracellular matrix of muscle cells is less well organized, and as a consequence the muscle functioning may be less. We have shown similar effects on the muscle tissue during overtraining of horses (te Pas et al. 2013). Overtraining may also related to body heat. Figure 1a shows the expression pattern of the Col5A2 as an example of this expression profile. Please note that the extracellular matrix protein 2 (ECM2) gene shows the same expression protein but also in the heart tissue of highland chickens.

This effect may be strengthened via heat shock protein expression. Figure 1b shows the expression pattern of the muscle-specific heat shock protein HSPB3. In highland chickens, the expression profile is the same as described above, while the opposite expression profile is observed in lowland chicken, suggesting protection from heat stress in lowland chickens.

The RNA minding motif protein 15 (RBM15) gene expression profile shows a sharp increase at noon in highland chickens. Since the gene is involved in repressing several signaling pathways, this further strengthens the hypothesis of loss of muscle tissue function (Fig. 1c).

#### Energy expenditure

Heat stress may affect the energy metabolism because animals may move less and eat less during heat stress. The mitochondrial pyruvate dehydrogenase kinase, isoenzyme 4 (PDK4) gene expression profile is shown in Fig. 1d. The profile has shown that the expression in all tissues is lower at noon. The expression levels in the morning and the evening are approximately the same suggesting that full recovery of the effect during increasing temperature is taking place in the afternoon when temperature is reduced. The protein is also important for maintaining normal blood pH and in preventing the accumulation of ketone bodies under starvation, which may relate to less feeding at noon.

#### Metabolism

The calcium binding protein 1 (CABP1) gene is involved in calcium-mediated cellular signal transduction regulating numerous processes. Its expression profile (Fig. 1e) shows a high peak at noon in highland chicken while lowland chickens show no regulation in muscle tissue. The DnaJ heat shock protein family (Hsp40) member C12 (DNAJC12) gene shows downregulation in the heart tissue of highland chicken but not in lowland chicken (Fig. 1f). This heat shock protein is involved in complex assembly, protein folding, and export. The profile suggests that the protein metabolism of the heart of highland chicken is affected. Apoptosis may be involved in all tissues and in both chickens. Figure 1g shows the expression profile of the ETS2 repressor factor (ERF) gene suggesting increased expression in both chickens and all three tissues.

The inhibitor of growth family member 2 (ING2) gene is involved in modulation of the activity of histone acetyltransferase and histone deacetylase complexes regulating gene expression and function in DNA repair and apoptosis (Fig. 1h). The expression profile shows a major downregulation of the expression especially in the heart tissue of lowland chickens. Regulation of the expression via this mechanism may be an adaptation to heat stress in lowland chickens. The nuclear receptor subfamily 4 group A member 3 (NR4A3) gene (Fig. 1i) has a role in the regulation of proliferation, survival, and differentiation of many different cell types and also in metabolism and inflammation. The expression profile shows a sharp decreased expression in heart and muscle tissues of highland chickens, while the lowland chickens show the opposite expression profiles in both tissues.

The biliverdin reductase A (BLVRA) gene is an example of a gene regulated in spleen tissue (Fig. 1j). The expression is downregulated during the day, in highland chicken already at noon and in lowland chicken during the afternoon. The gene is involved in heme catabolism and may therefore relate to blood oxygen transport capacity.

## Discussion

We investigated the biological mechanisms underlying the reaction to daytime heat stress in chicken. Regular diurnal changes in physical activity will also affect gene expression profile changes throughout the day. Therefore, we used two chicken lines differing for heat adaptation. We used a lowland chicken line used to high noon temperatures and a highland chicken line not used to this. Both chicken lines were exposed to lowland temperature conditions at the same day. The highland chickens were transported to the lowland location and rested for 20 h before the onset of the experiment. This recovery time is vital because if the recovery time is too short the effect of transportation is part of the measured response, and if the recovery time is too long the first signs of adaptation may be measured. But what is the best recovery time? Part of the answer may be found in the lairage time after transportation before slaughtering an animal. This is usually 3 h. In broilers, it is called “long-term recovery,” and it is effective in lowering plasma corticosterone levels (Zhang et al. 2009). Therefore, it may be assumed that this time effectively reduces transportation stress. However, this work is not about slaughtering the chickens but performing an experiment measuring circadian stress in relation to the (absence of) adaptation to high daytime temperatures. In our choice for 20 h resting time, we are sure that transportation stress is effectively reduced while the animals did not have to time to build up an adaptation response.

We expected differences between the chicken lines in their reactions to lowland daytime temperature variations. Therefore, we analyzed both chicken lines early in the morning, at noon, and late in the evening, i.e., before the daily rise in temperature, at the hottest moment, and after the decline in temperature. We measured the gene expression levels of three tissues: heart, muscle, and spleen. The heart is an organ that has been shown to react to heat stress and is involved in adaptation to environmental heat (Lin et al. 2006a, b). The breast muscle in chicken is the most valuable piece of meat and therefore important for productivity, and the spleen is important for immune functionality. Our results also indicated interactions among these organs showing the importance of investigating several tissues similarly to highlight the biological mechanisms regulated body wide. We compared the gene expression levels among day time points within each chicken line and per time point between the two chicken lines.

We showed differently expressed genes at each time point in each tissue of both chicken lines. The number of differently expressed genes and the (number of) functional annotation enrichments vary among chickens lines and time points. Not surprisingly, there is a high similarity in the patterns of the number of differently expressed genes and the functional annotation enrichments of the biological mechanisms at a higher regulatory level. In highland chicken, the number of differently expressed genes and functional annotations is spleen > muscle > heart, while lowland chicken showed heart > muscle > spleen. When comparing the differently expressed genes between the two chicken lines, we found muscle > heart > spleen. This shows directly that the two chicken lines differ in reaction to heat stress and that this difference is tissue-specific.

Next, we investigated the effect of time of the day related to circadian temperature variation. Here, we found major differences between the highland and the lowland chicken. In the heart tissue of highland chicken, the largest number of regulated biological mechanisms is in the morning, less in the afternoon, and only a low number of biological mechanisms remaining in the evening. In lowland chicken, this is the reverse. This suggests that the heart tissue of highland chicken reacts early to increased temperatures, while the heart tissue in lowland chicken reacts later, especially during declining temperatures in the afternoon. It cannot be excluded, however, that this was still a reaction to increasing temperatures in the morning but late. This correlates well with the observation that in the evening heart tissue was very different from the morning. Because we suggest that the next day the situation in the morning should have been restored, these data indicate that during the night, lowland chicken will need to regulate many biological processes. Since lowland chicken are used to the high noon temperatures, this may be an adaptation mechanism in heart tissue.

In muscle tissue, highland chicken showed similar levels of regulation throughout the day, while lowland chicken showed more regulation in the morning, suggesting that here lowland chicken reacts early while highland chicken react both early and late. However, since in the morning highland chicken and lowland chicken were highly similar, it is supposed that both chicken lines have similar biological-regulated mechanisms. During the afternoon, both chicken lines started to be different and a relatively high number of biological mechanisms differ between evening and morning situation in both chicken lines, suggesting that both chicken lines need to have repair mechanisms in muscle tissue during the night. Muscle tissue has a high volume, especially in broiler-type chicken, and this may require more time to respond to environmental heat and reverse the reaction in the afternoon.

Spleen tissue is remarkably different from the other two tissues, especially in highland chicken. This indicated that regulation of immune processes differed between the two chicken lines. The number of differentially regulated functional annotation enrichments is much higher in highland chicken than in lowland chicken in the morning. Thus, major immune process changes were taking place during rising temperatures in the chicken that were not used to high noon temperatures, while this was not the case in adapted chicken. In highland chickens, the number of genes showing differential expression between morning and evening time points was low indicating that most processes take place during daytime.

This is in agreement with the high number of functional annotation enrichments differing between evening and morning in highland chicken. Surprisingly, the number of functional annotation enrichments differing between highland and lowland chicken was much lower. This suggests that many of the changed functional annotations in highland chicken belong to the same biological processes.

In conclusion, we showed major differences in the regulation of biological functional annotations related to increasing and decreasing temperatures during the day. These differences related to the tissue under investigation and the adaptation of chicken.

### Biological mechanisms regulated in relation with circadian temperature changes

To investigate the biological processes underlying all these changes, we used functional annotation enrichment software and network analyses. For the latter, we used software to make protein networks and we focused on the hub genes and metabolites, and we studied groups of pathways. The added value of studying groups of pathways rather than single pathways is that it brings together more differently expressed genes and pathways. However, because of the sizes of several datasets, some networks were difficult to produce leading to loss of data for groups of pathways. However, data for the most important larger groups will remain. Data from these analyses were both confirming each other and were additive at other places.

Before discussing the regulated biological mechanisms per tissue throughout the day, we discuss a general theme found in our tissues in both chicken lines: epigenetics. Chromatin remodeling through both DNA methylation and histone (de)acetylation was reported. Especially transcriptional silencing was noticed, generally related to methylation and deacetylation (Malecová and Morris 2010; Weinberg and Morris 2016), although negative regulation of methylation leading to increased gene expression was also found. This can be a general biological process regulating all other biological mechanisms though transcription regulation. Epigenetic processes are of utmost importance for regulating genome-wide transcription activity (Orozco et al. 2015; Karsli-Ceppioglu et al. 2017). This suggest that the impact of temperature increase is such that genome-wide activity changes were induced, explaining the size of several of the datasets. Indeed, DNA methylation profile changes have been linked to response to stress in general (Unternaehrer et al. 2012) and to constant heat stress in particular (Hao et al. 2016), here we link it to circadian heat stress. Epigenomics is also a short-time regulator and much faster than mutation and selection (Berger 2007). Epigenetic regulation of the circadian clock has been reported (Sahar and Sassone-Corsi 2013), which makes it likely that also circadian-regulated processes are regulated via epigenetic mechanisms. Our results also indicate that the lowland chickens did not fully genetically adapt to the temperature conditions, although this epigenetic mechanism was found more in highland chickens than in lowland chickens, and epigenetic modifications may be inherited (Feeney et al. 2014; Orozco et al. 2015).

Finally, proteins especially regulated during environmental heat are the heat shock proteins. They perform functions dedicated to cellular survival. Indeed, we found HSPs involved in regulation of the energy metabolism, molecular chaperones ensuring correct protein folding for proper protein functioning, and muscle- and immune-specific functions. The tissues differ in these functions according to their different functions, i.e., immune functions were regulated in the spleen and the muscle tissue expressed the muscle-specific HSP required for proper actin polymerization. Especially the muscle tissue showed more HSP regulation in highland chicken as compared with lowland chicken suggesting that muscle tissue is especially vulnerable to heat stress, more than the other tissues under investigation. This is in agreement with our other muscle-specific data (see the Discussion below).

#### Heart tissue

Highland chicken heart tissue showed major regulation of protein synthesis in the morning that remains different until the evening. This may have affected the other reported processes including cell-cell contacts, circadian rhythm, apoptosis and mitosis, and cardiac and muscle developmental processes. This showed that heart tissue is under major remodeling during the day. The molecular chaperones and angiogenesis may add to this. Contrarily, lowland chicken mainly showed regulation of metabolic pathways: fatty acid synthesis and beta-oxidation and energy metabolism (mitochondrion). Cell-cell contacts and other cellular annotations were found only in the evening. This again shows that the heart tissue in lowland chicken differs between morning and evening. In both chicken lines, angiogenesis, circadian rhythm, and stress response mechanisms were observed indicating that daytime temperature is stressful for chicken hearts, regardless whether the conditions of the animals were adapted to changes in temperatures during the circadian cycle.

#### Muscle tissue

The data indicate both high similarity in biological processes between the two chicken lines and major differences of numbers of functional annotations between the two lines. This can be explained by the observation that similar processes were taking place in both chicken lines at different moments during the day. For this, we refer to the discussion above. Major general mechanisms include RNA (alternative) splicing protein synthesis and proteolysis, angiogenesis, nerve activity, apoptosis, and response to stress. This clearly showed that muscle tissue heavily reacts to environmental temperature. This has been shown for muscle tissue in several chicken lines before (Lara and Rostagno 2013; Zahoor et al. 2016, 2017). Especially in broiler chickens, muscle tissue is a heavily selected tissue. Moreover, the size and high metabolic activity of muscle tissue may lead to higher vulnerability to heat. More blood vessels in muscle tissue can support the loss of metabolically produced heat in the muscle tissue. This will also support nerve activity and tissue apoptosis. Together, it may improve the ability of the muscle tissue to react to heat stress properly. Consequently, there is a relation with immune processes.

#### Spleen tissue

As we have seen, both heart and muscle tissues have regulated immune-related biological mechanisms. Therefore, regulation of expression profiles of immune-related organs like the spleen was expected. Similarly, spleen functional annotations indicate cardiac-, muscle-, brain-, and ear-related regulated biological function. This indicated that the immune system has a widespread function, and the organs in an animal are connected as a functional unity (Martinez et al. 2015; Meng et al. 2016; Tidball 2017). This has been shown before, e.g., Veldman et al. (1984) reported a relation between spleen and ear disorders, and also in the traditional Chinese medicine spleen is seen as a regulator of muscle tissue and heart health (http://www.itmonline.org/5organs/spleen.htm).

Especially, the highland chicken showed a large array of different biological pathways in the morning. This was expected since the functional annotation indicated such a high number of regulated biological functions. However, when they were grouped, it appears that only a limited number of biological functions accounted for these annotations. A major portion relates to cellular division suggesting that the immune system is affected by the environmental temperature. Other major themes were protein metabolism and transport and molecular chaperones to maintain cellular integrity throughout the day. Several of these were also reported in cattle blood transcriptome analysis (Srikanth et al. 2017). Thus, cellular activity is regulated, and therefore immune capacity may be affected. A major difference between highland and lowland chickens is that highland chickens regulate IL4 biosynthesis, which regulates T helper 1 and 2, and B cell-cell activity in the immune response (Brown and Hural 1997), while lowland chickens regulate IL17, a T helper 2 cell activity and proinflammatory process regulator (Jin and Dong 2013). Although the consequence for immune capacity of the chickens is unknown at present, it may be expected that the animals have different capacity towards different pathogenic groups under heat stress conditions. In lowland chicken, energy metabolism and related metabolic pathways (fatty acid, cholesterol) is more regulated than in highland chicken, and the integration between energy metabolism and general metabolism is regulated. This suggests that the function of the immune cells may be affected. Angiogenesis and apoptosis appear general functions regulated in both chicken lines. These may be opposing biological functions. Angiogenesis will improve the functionality of the spleen, while apoptosis may negatively affect its functionality.

### Acclimation to hot environment

In a world affected by global warming, environmental heat stress is a real threat to future livestock health and productivity. In this study, we showed that organ health, remodeling, and (metabolic) function may be affected but that the effects differ for each organ and each chicken line. Adaptation of animals to environmental heat may be a slow process and specific for animals with a different adaptation background.

## Electronic supplementary material

ESM 1 Normalized heart tissue gene expression data (in FPKM). (XLSX 4098 kb)

ESM 2 Normalized muscle tissue gene expression data (in FPKM). (XLSX 3884 kb)

ESM 3 Normalized spleen tissue gene expression data (in FPKM). (XLSX 4208 kb)

ESM 4 Functional annotation analysis of the differentially expressed genes in the experimental groups at different times of the day using the DAVID software. (XLSX 30 kb)

ESM 5 Overview of network analysis. The data were obtained from protein network analysis using the STRING software, Protein and metabolite analysis using the STITCH software, and grouped pathways analysis using the Cytoscape software with the ClueGo app. (XLSX 141 kb)

ESM 6 Network analysis figures. All figures were converted to pdf files. (ZIP 47344 kb)
