# Supplementary material for: Transcriptomic profiles of muscle, heart, and spleen in reaction to circadian heat stress in Ethiopian highland and lowland male chicken
Source: Cell Stress Chaperones. 2018 Dec 18;24(1):175–94. doi: 10.1007/s12192-018-0954-6 (PMC6363629; doi:10.1007/s12192-018-0954-6)
Supplement: Supplementary file 6 — Network analysis figures. All figures were converted to pdf files. (ZIP 47344 kb) [file 12192_2018_954_MOESM6_ESM.zip › Reference.pdf]

Transcriptomic profiles of muscle, heart and spleen in reaction to circadian heat stress in Ethiopian highland and lowland male chicken

Marinus F.W. te Pas[1], Woncheoul Park[2], Krishnamoorthy Srikanth[2], Steve Kemp[3], Jun-Mo Kim[4], Dajeong Lim[2], Jong-Eun Park[2]

1: Wageningen UR Livestock Research, Animal Breeding and Genomics, Wageningen, The Netherlands; 2: Department of Animal Biotechnology and Environment, National Institute of Animal Science, Rural Development Administration, Wanju 55365, Republic of Korea, 3: Animal Biosciences, International Livestock Research Institute (ILRI), Nairobi, Kenya, 4: Department of Animal Science and Technology, Chung-Ang University, Anseong, Gyeonggi-do, 17546, Republic of Korea

Corresponding Authors:

Marinus F.W. te Pas (for the editorial office)

Marinus.tepas@wur.nl

Tel.: +31-317-480522

ORCID ID: 0000-0001-8849-3011

Jong-Eun Park (for future Korean readers)

jepark0105@korea.kr

Tel: +82-63-238-7309

Acknowledgement

This study was carried out with the support of "Cooperative Research Program for Agriculture Science & Technology Development (Project title: Biomarker investigation for livestock animal productivity through combinatory analysis with big-data related to genetic elements affecting economic traits, Project No. PJ011876; Project title: Investigation of expression profiles and genetic networks related to heat stress for chicken, Project No. PJ011221)" Rural Development Administration, Republic of Korea. And this study was supported by 2017 the RDA Fellowship Program of National Institute of Animal Science, Rural Development Administration, Republic of Korea.

Abstract

Temperature stress impact both welfare and productivity of livestock. Global warming is expected to increase the impact, especially in tropical areas. We investigated the biological mechanisms regulated by temperature stress due to the circadian temperature cycle in temperature adapted and non-adapted chicken under tropical conditions. We studied transcriptome profiles of heart, breast muscle, and spleen tissues of Ethiopian lowland chicken adapted to high circadian temperatures and non-adapted Ethiopian highland chicken under lowland conditions at three points during the day: morning, noon, and evening. Functional annotations and network analyses of genes differentially expressed among the time points of the day indicate major differences in the reactions of the tissues to increasing and decreasing temperatures, and also the two chickens lines differ. However, epigenetic changes of chromatin methylation and histone (de)acetylation seemed to be central regulatory mechanisms in all tissues in both chicken lines. Finally, all tissues showed differentially expressed genes between morning and evening times indicating biological mechanisms that need to change during the night to reach morning levels again the next day.

Keywords: Highland and Lowland male chicken; heat stress; transcriptome profiles; heart; breast muscle - meat; spleen

Introduction

Environmental changes, especially temperature changes, influence animal health, welfare and livestock productivity. Temperatures above normal are felt as heat stress (HS) by all living organisms, and heat stress can be defined as the sum of all external forces acting on an animal that causes an increase in body temperature and evokes a physiological response (Fuquay 1981; Phillips and Piggins 1992, Dikmen and Hansen, 2009). For example, it has been shown that the level of productivity and health of dairy cattle, goat, and sheep, and layer and broiler chicken is reduced by heat stress, and chicken mortality is increased (Fuquay 1981; Bottje and Harrison 1985; Lu 1989; Young 1990; Yahav et al. 1995; Silanikove 2000; West 2003; Marai et al. 2007). Physiologically, this may relate to reduced feed intake, suppressed immune response, and changed gene expression profiles and animal metabolism (Harrison and Biellier 1969; Emery et al. 1984; Altan et al. 2003; Mashaly et al. 2004; Lin et al. 2006A; Srikanth et al. 2017).

In tropical environments the daily temperature rise may induce heath stress to chicken. Local environmental conditions, such as altitude and natural conditions e.g. forests, affect the level and the impact of the temperature rise during the day. Global warming may increase the local temperatures further increasing heat stress effects. This may especially affect future livestock productivity in sub-Saharan countries (Thorton et al. 2009).

The impact of heat on broiler and layer strains of chicken has been studied before (Zahoor et al. 2017). The impact of heat stress in both genotypes of poultry is very similar (Zahoor et al. 2016, 2017). Apart from decreased productivity levels, physiological and behavioral changes were noted affecting not only animal welfare, but potentially also consumer health (Lara and Rostagno, 2013). Furthermore, acute heat stress affected gene expression profiles in the testis of broiler chickens increased the number of apoptotic cells, suggestive for reduced fertility (Wang et al. 2015). Changed gene expression in muscle tissue of broiler chickens has also been reported, suggestive for reduced meat productivity (Li et al. 2011).

As with most environmental challenges animals living for generations in hot climates adapts the animal to the environmental conditions (Lin et al. 2006B; Renaudeau et al. 2012; Lara and Rostagno, 2013; Kim et al. 2017). Chickens can adapt to circadian heath cycles (Lin et al. 2006B; Renaudeau et al. 2012; Lara and Rostagno, 2013). Furthermore, increasing the temperature with 1 – 1.5^{0}C during days 8 – 21 of egg incubation induced an adaptive response to hot temperatures in later life (Moraes et al. 2003; Janke and Tzschentke, 2010; Loyau et al. 2014A, B, 2015, 2016), including changed genome activity, suggesting epigenomic mechanisms (Loh et la., 2004; Yossifoff et al. 2008).

Together these data suggest that the transcriptomic changes mediating the response of the chicken to heat stress differs between adapted and non-adapted chickens. We here report on the differences in the transcriptomic profiles and the underlying regulated biological mechanisms in three vital tissues: heart, muscle (meat productivity) and spleen (immune) using a model system of chicken adapted to hot environments and non-adapted chickens. The objectives of these studies were: (1) the transcriptome profile differences between highland and lowland Ethiopian chicken before, during, and after hot daytime temperatures, and (2) the transcriptome profile changes in highland and lowland Ethiopian chicken separately during the day

Materials and Methods

Animals and experimental design

The care and experimental use of Ethiopia chicken was reviewed and approved by the Institutional Animal Care and Use Committee at the International Livestock Research Institute (ILRI, Ethiopia) (IACUC No.: 2016-216).

Ethiopian highland male chicken (N=9) from Addis Ababa (altitude 2400 meters above sea level, average high temperature 16^0C) were collected randomly and transferred to the Ethiopian lowland Awash village (altitude 950 meters above sea level, average high temperature 37^0C), where the experiments were performed after a 20 hour free-range period to relieve the animals from the transportation stress. Similarly, Ethiopian male lowland chicken from Awash village (Yangji hatchery, N=9) were collected randomly. The Ethiopian endogenous chicken lines originated from the same route to Ethiopia, but separated to different environmental conditions many generations ago. The lowland chickens were adapted to the circadian high temperature cycle, while the highland chicken were not.

All chicken were subjected to the lowland circadian temperature cycle at the same day. The chickens were slaughtered at the experimental location at three different time points: early in the morning at 6.00 AM (i.e. before the onset of increasing temperature, measured conditions 25.2 ^{0}C, 64% humidity; at noon, 12.00 AM (i.e. the hottest temperature, measured conditions 34.6^0C, 6 % humidity), and in the evening at 6.00 PM (i.e. after the temperature decline, measured conditions 26.3^0C, 9% humidity). Three chicken of each group were sacrificed per time point. Heart, breast muscle, and spleen tissue were snap frozen and stored at -80^0C until experimentation. The tissues were transferred to the Republic of Korea for RNAseq analysis.

RNAseq analysis

RNA was isolated using TRIzol (Invitrogen, USA). Hundred mg total RNA of each tissue was used to construct libraries for sequencing. The mRNA-Seq library was constructed with TruSeq mRNA library (Illumina Inc.) construction and sequenced with Illumina HiSeq2000 100PE (Illumina, Inc., San Diego, CA, USA) sequencing in accordance with the manufacturer's instructions. To remove rRNA, bead-based depletion was used. Libraries were sequenced independently. Obtained sequence reads were aligned to the Gallus gallus reference genome (NCBI GenBank (ftp://ftp.ensembl.org/pub/release-89/fasta/gallus_gallus/dna/). Preprocessing and mapping was performed using the pipelines with employing the Fastqc, trimmomatic and Tophat2. The read mapping was conducted using the RNA-Seq analysis package default settings (mismatch cost 2, insertion cost 3, length fraction 0.8, similarity fraction 0.8, and maximum number of reads 1) within the CLC software (Qiagen, Redwood city, CA, USA; https://www.qiagenbioinformatics.com/products/clc-genomics-workbench/). The number of mapped reads was normalized and measured in terms of reads per kilobase per million (FPKM) to determine transcript abundance using Cufflinks.

Bioinformatic analysis

This experiment investigates the reaction to circadian environmental temperatures in a hot climate zone using two groups of chicken differing for adaptation to high environmental temperatures. Therefore, the first analysis is the direct comparison between the two groups of chicken at each time point.

Each chicken type was investigated at three time points: morning, noon, evening (see above). Thus, for each chicken type three analyses were done comparing: morning-noon (i.e. the reaction to increasing temperatures), noon-evening (i.e. the reaction to decreasing temperature), and morning-evening (i.e. to investigate whether recovery from hot temperature is complete, or that further recovery is needed during night time.

For each analysis functional annotation analysis and network analyses were done. Because the analysis of the results using the chicken data was unsatisfying, we converted the ENSEMBLE gene numbers to official gene symbols and used the human annotation data. We compared the results obtained with the human annotation always with the results obtained with the chicken annotation. However, here we presented the results obtained with the human annotation. First differentially expressed genes were selected. For each gene the expression levels were compared between the two groups. These lists of differentially expressed genes were used for functional annotation enrichment analysis using the DAVID software (Huang et al. 2009A, B; https://david.ncifcrf.gov/). Network analyses were done using the online software tools STRING (Szklarczyk et

al. 2015, 2017; https://string-db.org/ and STITCH (Kuhn et al. 2014; Szklarczyk et al. 2016; http://stitch.embl.de/). The protein network was investigated with STRING, while the importance of specific metabolites was investigated with STITCH. The higher order pathway organization was investigated using the ClueGo app of Cytoscape (Shannon et al. 2003; Bindea et al. 2009). Groups of pathways were studied using KEGG and BioCarta pathways databases (Kanehisa and Goto, 2000; Kanehisa et al. 2014; http://www.genome.jp/kegg/; Nishimura, 2004; https://cgap.nci.nih.gov/Pathways/BioCarta_Pathways). The cut off value for a group was arbitrarily set to 3. Several datasets were too large to analyze for the software due to large numbers of differentially expressed genes. To solve this, the software was set to maximum specificity.

Results

Differentially expressed genes and related functional annotation enrichment

Supplementary files 1, 2 and 3 provide all RNAseq data of all genes after normalization for respectively heart, muscle, and spleen tissues. These were the data used for all analyses. Table 1 showed that the numbers of differentially expressed genes differs among chicken groups and tissues. The difference between highland and lowland chickens seemed largest in muscle tissue. Lowland chickens showed the largest number of differentially expressed genes in heart tissue while highland chicken showed largest number of differentially expressed genes in spleen and muscle tissue. This may indicated differential biological regulatory mechanisms operating in the two chicken groups. Furthermore, all tissues showed reaction to increasing temperatures (i.e. morning – noon) and to decreasing temperatures (i.e. noon – evening). The comparison between gene expression in the morning and the evening indicates that at the end of the day no (full) reversal of gene expression has taken place, suggesting further changes in gene expression patterns during the night. Especially in lowland chicken heart tissue major differences between morning and evening gene expression profiles were observed. In lowland chicken heart tissue the reaction during increasing temperatures seemed to be much smaller than during decreasing temperatures. The highland versus lowland chicken comparison suggested a similar mechanism in muscle tissue, which already start at noon.

Table 2 showed the number of significantly enriched functional annotations reported by DAVID. The number of enriched functional annotations using the chicken genome annotation is poor as compared to the human genome annotation data. Therefore, we used the functional annotation of the human genome, although we are aware of potential differences between mammals and birds.

Heart tissue of lowland chicken showed more functional annotation enrichments in lowland chicken than in highland chicken. Furthermore, lowland chicken showed the highest number of functional annotation enrichments during decreasing temperatures, which was not observed in highland chicken. Spleen tissue showed the opposite pattern with highest number of functional annotations enriched in highland chicken and largest number of enriched functional annotations during the period of increasing daytime temperatures. No differences between the chicken groups were found in muscle tissue. Again, all data indicate a major difference between morning and evening.

Functional annotations

In this paragraph we focus on the biological processes underlying the data of Table 2. A summary of the functional annotations can be found in the supplementary file 4. The data in the file focus on biological and cellular molecular processes and excludes the long lists of protein domains and interactions reported by DAVID, but includes the cluster analysis of DAVID. Functional annotations describing the same biological functionality were taken together, thus reducing the number of annotations without losing biological information. We describe the data per tissue and focus mainly on general mechanisms.

Heart tissue

Functional analysis of the differentially expressed genes between highland and lowland chicken indicate a role for mitochondrial processes, mainly related to oxidative energy metabolism, specifically at noon. The annotation was found also in lowland chicken in the afternoon period. In highland chicken during the morning period and in lowland chicken during the afternoon period and the morning – evening comparison the functional annotation relating to cellular communication pathways (collagen / extra cellular matrix / cell-cell adhesion and communication) is found. RNA metabolism, especially splicing is found in both chicken groups. The chicken groups have differential expression at noon and in the evening. While highland chicken show differential expression during the day, lowland chicken indicate especially a difference between the morning and evening analyses. This indicates that in highland chicken the expression is regulated during the day and returns to morning levels, but in lowland chicken the expression between morning and evening remains different. Only in highland chicken during the morning to noon period differential expression of ribosomal proteins is found. Finally, at several places heart / muscle annotations are indicated suggesting rebuilding or restructuring of the

heart tissue, which may relate to functional adaptations of the heart. Histone (de)acetylation and chromatin methylation were found in both chicken lines.

Muscle tissue

Analysis of the differentially expressed genes between highland and lowland chicken indicate splicing as an important mechanism in both chicken groups, and in both groups the expression in the evening did not return to the expression levels in the morning. The highland and lowland chicken groups differ in expression of the proteasome complex, especially during the afternoon period. This difference related mainly to the lowland chickens, which also indicate that expression levels also differ between morning and evening samples. The highland and lowland chicken groups differ also in expression for the cellular communication pathways (collagen / extra cellular matrix / cell-cell adhesion and communication), especially during the afternoon period. These effects were mainly found in the highland chickens, and not in the lowland chickens. The two chicken group also differed for the expression of mitochondrial genes, which seemed to be regulated only in the highland chicken, which also indicated that evening expression levels differ from morning expression levels. The two chicken groups also differed for acetylation, especially during the afternoon period. Differential expression was observed throughout the day in both chicken groups, indicating that expression remained different between evening and morning expression levels. Finally, at several places muscle annotations indicated suggesting rebuilding or restructuring of the muscle tissue, which may relate to functional adaptations of the muscle tissue.

Spleen tissue

Analysis of the differentially expressed genes between highland and lowland chicken indicate functional annotation enrichment of mitochondrial genes at noon. The expression profiles of the two chicken groups differ: in highland chicken differentially expressed mitochondrial genes were found during the morning period, while in lowland chicken functional annotation enrichment of mitochondrial genes was found during the afternoon period. Only in highland chicken the comparison of the expression levels of genes for the evening to morning showed differentially expressed genes. It should be noted that here especially mitochondrial organization was involved, while no oxidative phosphorylation mechanism annotations were reported. Functional annotation enrichment of cellular communication related pathways (collagen / extra cellular matrix / cell-cell adhesion and communication) were found between highland and lowland chickens, especially in the evening. Some indications for differential regulation of these processes were found in highland chicken, but not in low land

chicken. These data are supported by regulation of post-translational modification of proteins (Golgi / endoplasmic reticulum / protein transport) data. The two chicken groups differed especially in the morning, while specific regulation was found throughout the day in both chicken groups. Especially in lowland chicken regulation of alternative splicing was found throughout the day. The two chicken groups differed for expression of acetylation related genes at noon. However, both groups showed differential expression of acetylation related genes throughout the day, with some indication of histone-specific acetylation / -de-acetylation. Finally, enrichment of cell division was observed in both chicken groups in the morning, but not in the afternoon.

Network analyses

Three different network analyses were done: a protein network analysis, a protein network analysis including metabolites, and an integration of pathways analyses. Supplementary file 5 provides an overview of the results in the three tissues. All network analysis figures are provided in Supplementary file 6. For the protein networks we focused on the hub proteins defined as highly connected proteins as compared with other proteins in the network. Hub proteins were analyzed for functional annotation, and the functional annotations of the hub proteins are displayed in the Table 3. Furthermore, we observed in several networks specific nuclei, defined as groups of proteins connected with each other, but with less connections to other proteins. The proteins of the nuclei were analyzed for functional annotation – it proved to be that the proteins within a nucleus had similar functional annotation. Several of the network analyses were hampered by the creation of very dense networks, too-large-to-analyze. It should be noted that from these networks data may be missing or incomplete.

Table 3 shows a summary of the functional annotation enrichments of the protein hubs and nuclei of the networks of the three tissues at three different times of the day. The Table shows an extraction of the data that can be found in detail in the supplementary files 2 and 3. The Table shows that several general functional mechanisms are shared by the tissues: energy metabolism, (de)acetylation, mitosis / apoptosis, immune functions, circadian rhythm, (molecular) chaperone, stress, and (alternative) splicing. Double annotations suggest that stress and splicing, and stress, (molecular) chaperone, and mitosis / apoptosis are related to each other. Furthermore, several metabolites were found to be related to functional mechanisms of differentially expressed genes. Among these calcium and dopamine are shared among the tissues.

Groups of pathways related to heat stress changes throughout the day

Table 4 shows the results of the Cytoscape-ClueGo analysis of groups of (physiological) pathways harboring differently expressed genes in three tissues: heart (A), (breast) muscle (B), and spleen (C). In heart tissue the main remarkable result is that lowland chicken seems to respond more than the highland chicken. The lowland chicken shows differential expression in positive regulation of several metabolic pathways. Despite this, there is a long list of groups of pathways differing between morning and evening, indicating that the heat-related changes were not reversed yet at the end of the day during the afternoon. Among them, negative regulation of histone H3-K4 methylation indicates potential increased gene expression levels. In the afternoon a relation with the gut is suggested via the vagus nerve. The circadian rhythm is changed in highland chicken during the day and still changed at the end of the day. Major differences between the highland chicken and the lowland chicken also include cardiac and skeletal muscle developmental effects, fat cell differentiation and lipid storage, and cell division. There seems to be a relation with immune-related processes, and auditory signal responses (especially in the highland chicken).

Muscle tissue shows several main themes in both chicken lines, including (1) histone modifications, especially during the morning; (2) blood vessels development, in highland chicken mainly during the morning, and in lowland chicken mainly during the afternoon; (3) cell division, at the end of the day the situation still differs from the beginning of the day; (4) oxidative stress, in highland chicken especially during the morning; (5) apoptosis throughout the day; (6) response to stress, throughout the day, and in both chicken lines the evening situation still differs from the morning situation; (7) nerve activity, in the lowland chicken mainly during the morning, but the situation at the end of the day still differs from the beginning of the day; (8) immune response changes. The two chicken lines also differed for muscle differentiation processes and muscle activity. Especially, lowland chicken had a difference in photoperiodism in the morning.

Spleen tissue showed very few differences between the two chicken lines and in the evening, and they seemed to be similar. Some differences may include regulation of cell division, learning, protein localization at noon, and ear development and functioning. Despite of this the analysis of both chicken lines individually indicated more differences. Highland chickens showed a wide array of biological processes during the day, with the main focus of regulated processes during the morning. This includes the biological processes leading to apoptosis, histone H3 acetylation, inner ear development, and cell-cell contacts and cell cycle checkpoints. At the end of the day, the processes of apoptosis and cell division were still different from the morning expression levels. Other enriched biological mechanisms were energy metabolism and angiogenesis – which may be related to water loss, blood pressure, Cardiovascular disease, and IL4 biosynthesis groups of pathways. Contrary, the

lowland chickens line showed regulation of the pro-inflammatory IL17 production specifically during the morning. Lowland chickens also showed regulation of energy metabolism more widely via fatty acid, cholesterol, and energy metabolism. In general they showed regulation of the groups of pathways of circadian rhythm and cellular response to heat and stress in general, and oxidative stress specifically – which is still regulated in the evening. These all may be related to DNA hyper methylation.

Heat stress proteins related to heat stress – differences between chickens and tissues during the day

We investigated the differential expression of the heat shock proteins (HSPs) because these are especially related to the physiological reaction to these specific environmental conditions. We observed differences among the tissues over the day. In heart tissue we found that the two chickens differed in expression of HSPs especially during the afternoon. Functionally these HSPs are involved in mitochondrial electron transport and others function as molecular chaperones in the nucleus, regulating cell cycle control and signal transduction. The lowland and highland chicken differ in that the mitochondrial HSP is regulated in lowland but not in highland chicken. Contrary, in highland chicken a HSP transcription factor and cell cycle control and signal transduction regulating HSP were regulated, which were not regulated in lowland chicken. However, the chickens share regulation of HSP functioning as a molecular chaperone for correct folding of proteins and controlling protein transport in the ER. Calcium availability is important for this.

The regulated HSP gene profiles in the muscle tissue were different. First of all, the two chickens showed different HSP regulation throughout the day, especially for HSPs functioning as transcription regulators, cell cycle control and signal transduction, and especially for HSPs recruited to the nucleus under heat stress conditions. Furthermore, in the afternoon the chickens differ for regulation of glucocorticoid and mitochondrial electron transport signaling. At the end of the day a muscle-specific HSP is differently expressed involved in regulation of actin polymerization. Both chickens no HSPs regulated during the afternoon. Finally, it is remarkable that there were many more HSPs regulated in highland chickens than in lowland chickens. The focus in lowland chicken is on regulation of heat shock transcriptions factors during the morning and at the end of the day. In highland chickens this was also found during the morning, but throughout the day environmental stress regulated HSPs were found functioning as chaperones, and regulating the cellular response to heat and protecting from apoptosis. The muscle-specific HSP was found regulated at the end of the day.

Spleen tissue showed only differences between the two chickens in the afternoon, where the two chickens differed for HSPs regulating mitochondrial electron transport and cellular molecular chaperones

described especially in the cellular response to heat stress. However, when investigating the two chickens individually they showed different profiles. While lowland chickens showed regulated HSPs throughout the day, highland chickens did not have regulated HSPs in the afternoon. Lowland chickens regulated HSPs functioning for glucocorticoid signaling and molecular chaperones especially protecting against aggregated proteins. Highland chickens especially regulated HSPs during the morning. These HSPs especially regulated functions related to signaling to the innate immune system and controlling correct toll-like receptor folding in the endoplasmic reticulum. A relation with the melanosome was also suggested.

Visualization of circadian expression profiles in three tissues of highland and lowland chicken

Muscle cell integrity and functioning

The expression profiles of several proteins suggest that the integrity of the muscle tissue may be affected during circadian heat. The expression profiles of the Serpine (Serpin Peptidase Inhibitor, Clade E) gene involved in cellular adhesion and extracellular remodelling, the Col5A2 and Col6A3 (Collagen Type V Alpha 2 Chain, and Collagen Type VI Alpha 3 Chain, respectively) gene involved in signaling and focal adhesion, and the DPT (Dermatopontin) gene involved in cell-matrix interactions and matrix assembly all follow the same expression pattern showing that the expression in the muscle tissue is decreased during the day in highland chickens, but not in lowland chickens. The consequence may be that in highland chickens the extra cellular matrix of muscle cells is less well organized, and as a consequence the muscle functioning may be less. We have shown similar effects on the muscle tissue during overtraining of horses (te Pas et al., 2013) Overtraining may also related to body heat. Fig 1a shows the expression pattern of the Col5A2 as an example of this expression profile. Please note that the ECM2 (Extracellular Matrix Protein 2) gene shows the same expression protein, but also in the heart tissue of highland chickens.

This effect may be strengthened via heat shock protein expression. Fig 1b shows the expression pattern of the muscle-specific heat shock protein HSPB3. In highland chickens the expression profile is the same as described above, while the opposite expression profile is observed in lowland chicken, suggesting protection from heat stress in lowland chickens.

The RBM15 (RNA Binding Motif Protein 15) gene expression profile shows a sharp increase at noon in highland chickens. Since the gene is involved in repressing several signaling pathways this further strengthen the hypothesis of loss of muscle tissue function (Fig 1c).

Energy expenditure

Heat stress may affect the energy metabolism because animals may move less and eat less during heat stress. The mitochondrial PDK4 (Pyruvate Dehydrogenase Kinase, Isoenzyme 4) gene expression profile is shown in Fig 1d. The profile shown that the expression in all tissues in lower at noon. The expression levels in the morning and the evening are approximately the same suggesting that full recovery of the effect during increasing temperature is taking place in the afternoon when temperature is reduced. The protein is also important for maintaining normal blood pH and in preventing the accumulation of ketone bodies under starvation, which may relate to less feeding at noon.

Metabolism

The CABP1 (Calcium Binding Protein 1) gene is involved in calcium mediated cellular signal transduction regulating numerous processes. Its expression profile (Fig 1e) shows a high peak at noon in highland chicken while lowland chicken show no regulation in muscle tissue. The DNAJC12 (DnaJ Heat Shock Protein Family (Hsp40) Member C12) gene shows down regulation in the heart tissue of highland chicken, but not in lowland chicken (Fig 1f). This heat shock protein is involved in complex assembly, protein folding, and export. The profile suggests that the protein metabolism of the heart of highland chicken is affected. Apoptosis may be involved in all tissues and in both chickens. Fig 1g shows the expression profile of the ERF (ETS2 Repressor Factor) gene suggesting increased expression in both chickens and all three tissues.

The ING2 (Inhibitor Of Growth Family Member 2) gene, involved in modulation of the activity of histone acetyltransferase and histone deacetylase complexes regulating gene expression and function in DNA repair and apoptosis (Fig 1h). The expression profile shows a major down regulation of the expression especially in the heart tissue of lowland chickens. Regulation of the expression via this mechanism may be an adaptation to heat stress in lowland chickens. The NR4A3 (Nuclear Receptor Subfamily 4 Group A Member 3) gene (Fig 1i) has a role in the regulation of proliferation, survival and differentiation of many different cell types and also in metabolism and inflammation. The expression profile shows a sharp decreased expression in heart and muscle tissues of highland chickens while the lowland chickens show the opposite expression profiles in both tissues.

The BLVRA (Biliverdin Reductase A) gene is an example of a gene regulated in spleen tissue (Fig 1j). The expression is down regulated during the day, in highland chicken already at noon, in lowland chicken during the afternoon. The gene is involved in heme catabolism, and may therefore relate to blood oxygen transport capacity.

Discussion

We investigated the biological mechanisms underlying the reaction to daytime heat stress in chicken. Regular diurnal changes in physical activity will also affect gene expression profile changes throughout the day. Therefore we used two chicken lines differing for heat adaptation. We used a lowland chicken line used to high noon temperatures, and a highland chicken line not used to this. Both chicken lines were exposed to lowland temperature conditions at the same day. The highland chicken were transported to the lowland location and rested for 20 hours before the onset of the experiment. This recovery time is vital because if the recovery time is too short the effect of transportation is part of the measured response, and if the recovery time is too long the first signs of adaptation may be measured. But what is the best recovery time? Part of the answer may be found in the lairage time after transportation before slaughtering an animal. This is usual 3 hours. In broilers it is called "long-term recovery", and it is effective in lowering plasma corticosterone levels (Zhang et al. 2009). Therefore, it may be assumed that this time effectively reduces transportation stress. However, this work is not about slaughtering the chickens but performing an experiment measuring circadian stress in relation to the (absence of) adaptation to high daytime temperatures. In our choice for 20 hours resting time we are sure that transportation stress is effectively reduced while the animals did not have to time to build up an adaptation response.

We expected differences between the chicken lines in their reactions to lowland daytime temperature variations. Therefore, we analyzed both chicken lines early in the morning, at noon, and late in the evening, i.e. before the daily rise in temperature, at the hottest moment, and after the decline in temperature. We measured the gene expression levels of three tissues: heart, muscle, and spleen. The heart is an organ that has been shown to react to heat stress and is involved in adaptation to environmental heat (Lin et al. 2006), The breast muscle in chicken is the most valuable piece of meat and therefore important for productivity, and the spleen is important for immune functionality. Our results also indicated interactions among these organs showing the importance of investigating several tissues similarly to highlight the biological mechanisms regulated body wide. We compared the gene expression levels among day time points within each chicken line, and per time point between the two chicken lines.

We showed differently expressed genes at each time point in each tissue of both chicken lines. The number of differently expressed genes and the (number of) functional annotation enrichments vary among chickens lines and time points. Not surprisingly, there is a high similarity in the patterns of the number of differently expressed genes and the functional annotation enrichments of the biological mechanisms at a higher regulatory level. In highland chicken the number of differently expressed genes and functional annotations is spleen > muscle > heart, while lowland chicken showed heart > muscle > spleen. When comparing the

differently expressed genes between the two chicken lines we found muscle > heart > spleen. This shows directly that the two chicken lines differ in reaction to heat stress, and that this difference is tissue-specific.

Next we investigated the effect of time of the day related to circadian temperature variation. Here we found major differences between the highland and the lowland chicken. In the heart tissue of highland chicken the largest number of regulated biological mechanisms is in the morning, less in the afternoon, and only a low number of biological mechanisms remaining in the evening. In lowland chicken this is the reverse. This suggests that the heart tissue of highland chicken reacts early to increased temperatures, while the heart tissue in lowland chicken reacts later, especially during declining temperatures in the afternoon. It cannot be excluded however, that this was still a reaction to increasing temperatures in the morning, but late. This correlate well with the observation that in the evening heart tissue was very different from the morning. Because we suggest that the next day the situation in the morning should have been restored, these data indicate that during the night lowland chicken will need to regulate many biological processes. Since lowland chicken are used to the high noon temperatures this may be an adaptation mechanism in heart tissue.

In muscle tissue, highland chicken showed similar levels of regulation throughout the day, while lowland chicken showed more regulation in the morning, suggesting that here lowland chicken reacts early while highland chicken react both early and late. However, since in the morning highland chicken and lowland chicken were highly similar, it is supposed that both chicken lines has similar biological regulated mechanisms. During the afternoon both chicken lines started to be different and a relatively high number of biological mechanisms differs between evening and morning situation in both chicken lines, suggesting that both chicken lines need to have repair mechanisms in muscle tissue during the night. Muscle tissue has a high volume, especially in broiler-type chicken, and this may require more time to respond to environmental heat, and reverse the reaction in the afternoon.

Spleen tissue is remarkably different from the other two tissues, especially in highland chicken. This indicated that regulation of immune processes differed between the two chicken lines. The number of differentially regulated functional annotation enrichments is much higher in highland chicken than in lowland chicken in the morning. Thus, major immune process changes were taking place during rising temperatures in the chicken that were not used to high noon temperatures while this was not the case in adapted chicken. In highland chickens the number of genes showing differential expression between morning and evening time points were low indicating that most processes take place during daytime.

This is in agreement with the high number of functional annotation enrichments differing between evening and morning in highland chicken. Surprisingly, the number of functional annotation enrichments differing between highland and lowland chicken was much lower. This suggests that many of the changed functional annotations in highland chicken belong to the same biological processes.

Concluding, we showed major differences in the regulation of biological functional annotations related to increasing and decreasing temperatures during the day. These differences related to the tissue under investigation and the adaptation of chicken.

Biological mechanisms regulated in relation with circadian temperature changes

To investigate the biological processes underlying all these changes we used functional annotation enrichment software and network analyses. For the latter we used software's to make protein networks and we focused on the hub genes and metabolites, and we studied groups of pathways. The added value of studying groups of pathways rather than single pathways is that it bring together more differently expressed genes and pathways. However, because of the sizes of several datasets some networks were difficult to produce leading to loss of data for groups of pathways. However, data for the most important larger groups will remain. Data from these analyses were both confirming each other and were additive at other places.

Before discussing the regulated biological mechanisms per tissue throughout the day we discuss a general theme found in our tissues in both chicken lines: epigenetics. Chromatin remodeling through both DNA methylation and histone (de)acetylation were reported. Especially transcriptional silencing was noticed, generally related to methylation and deacetylation (Malecová and Morris, 2010; Weinberg and Morris, 2016), although negative regulation of methylation leading to increased gene expression was also found. This can be a general biological process regulating all other biological mechanisms though transcription regulation. Epigenetic processes are of utmost importance for regulating genome-wide transcription activity (Orozco et al. 2015; Karsli-Ceppioglu et al. 2017). This suggest that the impact of temperature increase is such that genome-wide activity changes were induced, explaining the size of several of the datasets. Indeed, DNA methylation profile changes has been linked to response to stress in general (Unternaehrer et al. 2012), and to constant heat stress in particular (Hao et al. 2016), here we link it to circadian heat stress. Epigenomics is also a short-time regulator and much faster than mutation and selection (Berger, 2007). Epigenetic regulation of the circadian clock has been reported (Sahar and Sassone-Corsi, 2013), which makes it likely that also circadian-regulated processes are regulated via epigenetic mechanisms. Our results also indicates that the lowland chickens did not fully

genetically adapt to the temperature conditions, although this epigenetic mechanism was found more in highland chickens than in lowland chickens, and epigenetic modifications may be inherited (Nilsson and Skinner, 2014; Orozco et al. 2015).

Finally, proteins especially regulated during environmental heat are the heat shock proteins. They perform functions dedicated to cellular survival. Indeed, we found HSPs involved in regulation of the energy metabolism, molecular chaperones ensuring correct protein folding for proper protein functioning, and muscle and immune-specific functions. The tissues differ in these functions according to their different functions, i.e. immune functions were regulated in the spleen and the muscle tissue expressed the muscle-specific HSP required for proper actin polymerization. Especially the muscle tissue showed more HSP regulation in highland chicken as compared with lowland chicken suggesting that muscle tissue is especially vulnerable to heat stress, more than the other tissues under investigation. This is in agreement with our other muscle-specific data (see the discussion below).

Heart tissue: Highland chicken heart tissue showed major regulation of protein synthesis in the morning that remains different until the evening. This may have affected the other reported processes including cell-cell contacts, circadian rhythm, apoptosis and mitosis, and cardiac and muscle developmental processes. This showed that heart tissue is under major remodeling during the day. The molecular chaperones and angiogenesis may add to this. Contrary, lowland chicken mainly showed regulation of metabolic pathways: fatty acid synthesis and beta-oxidation, and energy metabolism (mitochondrion). Cell-cell contacts and other cellular annotations were found only in the evening. This again shows that the heart tissue in lowland chicken differs between morning and evening. In both chicken lines, angiogenesis, circadian rhythm and stress response mechanisms were observed indicating that daytime temperature is stressful for chicken hearts, regardless whether the conditions of the animals were adapted to changes in temperatures during the circadian cycle.

Muscle tissue: The data indicate both high similarity in biological processes between the two chicken lines, and major differences of numbers of functional annotations between the two lines. This can be explained by the observation that similar processes were taking place in both chicken lines at different moments during the day. For this we refer to the discussion above. Major general mechanisms include RNA (alternative) splicing protein synthesis and proteolysis, angiogenesis, nerve activity, apoptosis, and response to stress. This clearly showed that muscle tissue heavily react to environmental temperature. This has been shown for muscle tissue in several

chicken lines before (Lara and Rostagno, 2013; Zahoor et al. 2016, 2017). Especially in broiler chickens, muscle tissue is a heavily selected tissue. Moreover, the size and high metabolic activity of muscle tissue may lead to higher vulnerability to heat. Chickens. More blood vessels in muscle tissue can support the loss of metabolically produced heat in the muscle tissue. This will also support nerve activity and tissue apoptosis. Together, it may improve the ability of the muscle tissue to react to heat stress properly. Consequently, there is a relation with immune processes.

Spleen tissue: As we have seen, both heart and muscle tissues have regulated immune related biological mechanisms. Therefore, regulation of expression profiles of immune-related organs like the spleen was expected. Similarly, spleen functional annotations indicate cardiac, muscle, brain, and ear related regulated biological function. This indicated that the immune system has a widespread function, and the organs in an animal are connected as a functional unity (Martinez et al. 2015; Meng et al. 2016; Tidball, 2017). This has been shown before, e.g. Veldman et al. (1984) reported a relation between spleen and ear disorders, and also in the traditional Chinese medicine spleen is seen as a regulator of muscle tissue and heart health (http://www.itmonline.org/5organs/spleen.htm).

Especially, the highland chicken showed a large array of different biological pathways in the morning. This was expected since the functional annotation indicated such a high number of regulated biological functions. However, when they were grouped, it appears that only a limited number of biological functions accounted for these annotations. A major portion relates to cellular division suggesting that the immune system is affected by the environmental temperature. Other major themes were protein metabolism and transport, and molecular chaperones to maintain cellular integrity throughout the day. Several of these were also reported in cattle blood transcriptome analysis (Srikanth et al. 2017). Thus, cellular activity is regulated, and therefore immune capacity may be affected. A major difference between highland and lowland chickens is that highland chickens regulate IL4 biosynthesis, which regulates T helper 1 and 2, and B-cell cell activity in the immune response (Brown and Hural, 1997), while lowland chickens regulate IL17, a T helper 2 cell activity and proinflammatory process regulator (Jin and Dong, 2013). Although the consequence for immune capacity of the chickens is unknown at present, but it may be expected that the animals have different capacity towards different pathogenic groups under heat stress conditions. In lowland chicken energy metabolism and related metabolism (fatty acid, cholesterol) is more regulated than in highland chicken, and the integration between energy metabolism and general metabolism is regulated. This suggests that the function of the immune cells may be

affected. Angiogenesis and apoptosis appear general functions regulated in both chicken lines. These may be opposing biological functions. Angiogenesis will improve the functionality of the spleen, while apoptosis may negatively affect its functionality.

Acclimation to hot environment

In a world affected by global warming environmental heat stress is a real thread to future livestock health and productivity. In this study we showed that organ health, remodeling, and (metabolic) function may be affected, but that the effects differ for each organ and each chicken line. Adaptation of animals to environmental heat may be a slow process and specific for animals with a different adaptation background.

References

Altan Ö, Pabuçcuoğlu A, Altan A, Konyalioğlu S, Bayraktar H (2003) Effect of heat stress on oxidative stress, lipid peroxidation and some stress parameters in broilers. Br Poultry Sci 44:545-550.

Berger SL (2007) The complex language of chromatin regulation during transcription. Nature 447:407–412.

Bindea G, Mlecnik B, Hackl H, Charoentong P, Tosolini M, Kirilovsky A, Fridman WH, Pagès F, Trajanoski Z, Galon J (2009) ClueGO: a Cytoscape plug-in to decipher functionally grouped gene ontology and pathway annotation networks. Bioinformatics 25:1091-1093. http://doi:10.1093/bioinformatics/btp101.

Bottje W, Harrison P (1985) Effect of carbonated water on growth performance of cockerels subjected to constant and cyclic heat stress temperatures. Poultry Sci 64:1285-1292.

Brown MA, Hural J (1997) Functions of IL-4 and control of its expression. Crit Rev Immunol17:1-32.

Dikmen S, Hansen PJ (2009) Is the temperature-humidity index the best indicator of heat stress in lactating dairy cows in a subtropical environment? J Dairy Sci 92:109-116.

Emery DA, Vohra P, Ernst R, Morrison S (1984) The effect of cyclic and constant ambient temperatures on feed consumption, egg production, egg weight, and shell thickness of hens. Poultry Sci 63:2027-2035.

Feeney A, Nilsson E, Skinner MK (2014) Epigenetics and transgenerational inheritance in domesticated farm animals. J Anim Sci Biotechnol 5:48. http://doi:10.1186/2049-1891-5-48.

Fuquay J (1981) Heat stress as it affects animal production. J Anim Sci 52:164-174.

Hao Y, Cui Y, Gu X (2016). Genome-wide DNA methylation profiles changes associated with constant heat stress in pigs as measured by bisulfite sequencing. Sci Rep 6:27507. http://doi.org/10.1038/srep27507.

Harrison P, Biellier H (1969) Physiological response of domestic fowl to abrupt changes of ambient air temperature. Poultry Sci 48:1034-45.

Huang DW, Sherman BT, Lempicki RA (2009A) Systematic and integrative analysis of large gene lists using DAVID Bioinformatics Resources. Nature Protoc 4:44-57.

Huang DW, Sherman BT, Lempicki RA (2009B) Bioinformatics enrichment tools: paths toward the comprehensive functional analysis of large gene lists. Nucleic Acids Res 37:1-13.

Janke O, Tzschentke B (2010) Long-Lasting Effect of Changes in Incubation Temperature on Heat Stress Induced Neuronal Hypothalamic c-Fos Expression in Chickens. Open Ornithol 3:150-155.

Jin W, Dong C (2013) IL-17 cytokines in immunity and inflammation. Emerging Microbes and Infections. 2013;2(9):e60-. http://doi:10.1038/emi.2013.58.

Kanehisa M, Goto S (2000) KEGG: Kyoto encyclopedia of genes and genomes. Nucleic Acids Res 28:27–30.

Kanehisa M, Goto S, Sato Y, Kawashima M, Furumichi M, Tanabe M (2014) Data, information, knowledge and principle: back to metabolism in KEGG. Nucleic Acids Res 42:D199–D205.

Karsli-Ceppioglu S, Dagdemir A, Judes G, Lebert A, Penault-Llorca F, Bignon Y-J, Bernard-Gallon D (2017) The Epigenetic Landscape of Promoter Genome-wide Analysis in Breast Cancer. Sci Rep 7:6597. http://doi:10.1038/s41598-017-06790-z.

Kim J-M, Lim K-S, Byun M, Lee K-T, Yang Y-r, Park M, Lim D, Chai H-H, Bang H-T, Hwangbo J, Choi Y-h, Cho Y-M, Park J-E (2017) Identification of the acclimation genes in transcriptomic responses to heat stress of White Pekin duck. Cell Stress Chaperon (2017) 22:787–797. http://doi:10.1007/s12192-017-0809-6.

Kotak S, Larkindale J, Lee U, von Koskull-Döring P, Vierling E, Scharf K-D (2007) Complexity of the heat stress response in plants. Curr Opin Plant Biol 10:310-316.

Kuhn M, Szklarczyk D, Pletscher-Frankild S, Blicher TH, von Mering C, Jensen LJ, Bork P (2014) STITCH 4: integration of protein-chemical interactions with user data. Nucleic Acid Res 42:D401-407.

Lara LJ, Rostagno MH (2013) Impact of Heat Stress on Poultry Production. Animals 3:356-369. http://doi:10.3390/ani3020356.

Li C, Wang X, Wang G, Li N, Wu C (2011) Expression analysis of global gene response to chronic heat exposure in broiler chickens (Gallus gallus) reveals new reactive genes. Poultry Sci 90:1028–1036. https://doi.org/10.3382/ps.2010-01144.

Lin H, Decuypere E, Buyse J (2006A) Acute heat stress induces oxidative stress in broiler chickens. Comp Biochem Phys A 144:11-17.

Lin H, Jiao H, Buyse J, Decuypere E (2006B). Strategies for preventing heat stress in poultry. W Poultry Sci 62:71-86. http://doi:10.1079/WPS200585.

Loh B, Maier I, Winar A, Janke O, Tzschentke B (2004) Prenatal Development of Epigenetic Adaptation Processes in Poultry: Changes in Metabolic and Neuronal Thermoregulatory Mechanisms. Av Poult Biol Rev 15:119-128.

Loyau T, Métayer-Coustard S, Berri C, Crochet S, Cailleau-Audouin E, Sannier M, Chartrin P, Praud C, Hennequet-Antier C, Rideau N, Couroussé N, Mignon-Grasteau S, Everaert N, Duclos MJ, Yahav S, Tesseraud S, Collin A (2014A) Thermal manipulation during embryogenesis has long-term effects on muscle and liver metabolism in fast-growing chickens. PLoS One 2014;9(9):e105339. http://doi:10.1371/journal.pone.0105339.

Loyau T, Collin A, Yenisey Ç, Crochet S, Siegel PB, Akşit M, Yalçin S (2014B) Exposure of embryos to cyclically cold incubation temperatures durably affects energy metabolism and anti-oxidant pathways in broiler chickens. Poult Sci 93:1–9. http://doi:10.3382/ps.2014-03881.

Loyau T, Bedrani L, Berri C, Métayer-Coustard S, Praud C, Coustham V, Mignon-Grasteau S, Duclos MJ, Tesseraud S, Rideau N, Hennequet-Antier C, Everaert N, Yahav S, Collin A (2015) Cyclic variations in incubation conditions induce adaptive responses to later heat exposure in chickens: a review. Animal 9:76–85.

Loyau T, Hennequet-Antier C, Coustham V, Berri C, Leduc M, Crochet S, Sannier M, Duclos MJ, Mignon-Grasteau S, Tesseraud S, Brionne A, Métayer-Coustard S, Moroldo M, Lecardonnel J, Martin P, Lagarrigue S, Yahav, S Collin A (2016) Thermal manipulation of the chicken embryo triggers differential gene expression in response to a later heat challenge. BMC Genomics 17:329. http://doi.org/10.1186/s12864-016-2661-y.

Lu C (1989) Effects of heat stress on goat production. Small Ruminant Res 2:151-162.

Malecová B, Morris KV (2010) Transcriptional gene silencing mediated by non-coding RNAs. Cur Opin Mol Ther 12:214–222.

Marai I, El-Darawany A, Fadiel A, Abdel-Hafez M (2007) Physiological traits as affected by heat stress in sheep—a review. Small Ruminant Res 71:1-12.

Martinez SR, Gay MS, Zhang L (2015) Epigenetic mechanisms in heart development and disease. Drug Discov Today 20:799-811. http://doi:10.1016/j.drudis.2014.12.018.

Mashaly M, Hendricks 3rd G, Kalama M, Gehad A, Abbas A, Patterson P (2004) Effect of heat stress on production parameters and immune responses of commercial laying hens. Poultry Sci 83:889-894.

Meng X, Yang J, Dong M, Zhang K, Tu E, Gao Q, Chen W, Zhang C, Zhang Y (2016) Regulatory T cells in cardiovascular diseases. Nature Rev Cardiol 13:167–179.

Moraes VMB, Malheiros RD, Bruggeman V, Collin A, Tona K, Van As P, Onagbesan OM, Buyse J, Decuypere E, Macari M (2003) Effect of thermal conditioning during embryonic development on aspects of physiological responses of broilers to heat stress. J Therm Biol 28:133-140, ISSN 0306-4565, https://doi.org/10.1016/S0306-4565(02)00049-9.

Nishimura D (2004) A View From the Web. BioCarta. www.biocarta.com Biotech Software & Internet Rep 2:117-120.

Orozco LD, Morselli M, Rubbi L, Guo W, Go J, Shi H, Lopez D, Furlotte NA, Bennett BJ, Farber CR, Ghazalpour A, Zhang MQ, Bahous R, Rozen R, Lusis AJ, Pellegrini M (2015) Epigenome-wide association of liver methylation patterns and complex metabolic traits in mice. Cell Metab 21:905-917.

Phillips C, Piggins D (1992) Farm animals and the environment. CAB International, Wallingford OX10 8DE, UK. ISBN : 0851987885

Renaudeau D, Collin A, Yahav S, De Basilio V, Gourdine J,Collier R (2012) Adaptation to hot climate and strategies to alleviate heat stress in livestock production. Animal, 6:707-728. http://doi:10.1017/S1751731111002448.

Sahar S, Sassone-Corsi P (2013) The epigenetic language of circadian clocks. Handb Exp Pharmacol.217:29-44. http://doi:10.1007/978-3-642-25950-0_2.

Shannon P, Markiel A, Ozier O, Baliga NS, Wang JT, Ramage D, Amin N, Schwikowski B, Ideker T (2003) Cytoscape: a software environment for integrated models of biomolecular interaction networks. Genome Res 13:2498-2504.

Silanikove N (1992) Effects of water scarcity and hot environment on appetite and digestion in ruminants: a review. Livest Prod Sci 30:175-194.

Srikanth K, Kwon A, Lee E, Chung H (2017). Characterization of genes and pathways that respond to heat stress in Holstein calves through transcriptome analysis. Cell Stress Chaperon 22: 29-42.

Szklarczyk D, Franceschini A, Wyder S, Forslund K, Heller D, Huerta-Cepas J, Simonovic M, Roth A, Santos A, Tsafou KP, Kuhn M, Bork P, Jensen LJ, von Mering C (2015} STRING v10: protein-protein interaction networks, integrated over the tree of life. Nucleic Acid Res 43:D447-D542.

Szklarczyk D, Santos A, von Mering C, Jensen LJ, Bork P, Kuhn M (2016) STITCH 5: augmenting protein-chemical interaction networks with tissue and affinity data. Nucleic Acid Res 44:D380-D384.

Szklarczyk D, Morris JH, Cook H, Kuhn M, Wyder S, Simonovic M, Santos A, Doncheva NT, Roth A, Bork P, Jensen LJ, von Mering C (2017) The STRING database in 2017: quality-controlled protein-protein association networks, made broadly accessible. Nucleic Acid Res 45:D362-D368.

Te Pas MFW, Wijnberg ID, Hoekman AJW, De Graaf-Roelfsema E, Keizer HA, Van Breda E, Ducro B, and Van der Kolk JH (2013) Skeletal muscle transcriptome profiles related to different training intensities and detraining in Standardbred horses: A search for overtraining biomarkers. Vet. J. 197: 717-723. http://dx.doi.org/10.1016/j.tvjl.2013.03.052.

Tidball JG (2017) Regulation of muscle growth and regeneration by the immune system. Nat Rev Immunol 17:165-178. http://doi:10.1038/nri.2016.150.

Thornton PK, van de Steeg J, Notenbaert A, Herrero M (2009) The impacts of climate change on livestock and livestock systems in developing countries: A review of what we know and what we need to know. Agric Syst 101:113-127. https://doi.org/10.1016/j.agsy.2009.05.002.

Unternaehrer E, Luers P, Mill J, Dempster E, Meyer AH, Staehli S, Lieb R, Hellhammer DH, Meinlschmidt G (2012) Dynamic changes in DNA methylation of stress-associated genes (OXTR, BDNF) after acute psychosocial stress. Transl Psych 14;2:e150. http://doi:10.1038/tp.2012.77.

Veldman JE, Roord JJ, O'connor AF, Shea JJ (1984), Autoimmunity and inner ear disorders: An immune-complex mediated sensorineural hearing loss. The Laryngoscope 94:501–507. http://doi:10.1288/00005537-198404000-00014

Wang SH, Cheng CY, Tang PC, Chen CF, Chen HH, Lee Y-P, Huang S-Y (2015) Acute Heat Stress Induces Differential Gene Expressions in the Testes of a Broiler-Type Strain of Taiwan Country Chickens. PLOS ONE 10(5): e0125816. https://doi.org/10.1371/journal.pone.0125816.

Weinberg MS, Morris KV (2016) Transcriptional gene silencing in humans, Nucleic Acid Res 44:6505–6517. https://doi.org/10.1093/nar/gkw139.

West J (2003) Effects of heat-stress on production in dairy cattle. J Dairy Sci 86:2131-2144.

Yahav S, Goldfeld S, Plavnik I, Hurwitz S (1995) Physiological responses of chickens and turkeys to relative humidity during exposure to high ambient temperature. J Therm Biol 20:245-253.

Yossifoff M, Kisliouk T, Meiri N (2008) Dynamic changes in DNA methylation during thermal control establishment affect CREB binding to the brain-derived neurotrophic factor promoter. Eur J Neurosci 28:2267–2277. http://doi:10.1111/j.1460-9568.2008.06532.x

Young RA (1990) Stress proteins and immunology. Ann Rev Immunol 8:401-420.

Zahoor I, Mitchell MA, Hall S, Beard PM, Gous RM, De Koning DJ, Hocking PM (2016) Predicted optimum ambient temperatures for broiler chickens to dissipate metabolic heat do not affect performance or improve breast muscle quality. Br Poult Sci 57:134-141. http://doi:10.1080/00071668.2015.1124067.

Zahoor I, de Koning DJ, Hocking PM. (2017) Transcriptional profile of breast muscle in heat stressed layers is similar to that of broiler chickens at control temperature. Genet Sel Evol 49:69. http://doi:10.1186/s12711-017-0346-x.

Zhang L, Yue HY, Zhang HJ, Xu L, Wu SG, Yan HJ, Gong YS, Qi GH (2009) Transport stress in broilers: I. Blood metabolism, glycolytic potential, and meat quality. Poultry Sci 88:2033–2041. https://doi.org/10.3382/ps.2009-00128

Fig 1. Visualization of circadian expression profiles in three tissues of highland and lowland chicken. a: Col5A2 (Collagen Type V Alpha 2 Chain); b: HSPB3 (Heat shock protein B3); c: RBM15 (RNA Binding Motif Protein 15); d: PDK4 (Pyruvate Dehydrogenase Kinase, Isoenzyme 4); e: CABP1 (Calcium Binding Protein 1); f: DNAJC12 (DnaJ Heat Shock Protein Family (Hsp40) Member C12); g: ERF (ETS2 Repressor Factor); h: ING2 (Inhibitor Of Growth Family Member 2); i: NR4A3 (Nuclear Receptor Subfamily 4 Group A Member 3); j: BLVRA (Biliverdin Reductase A).

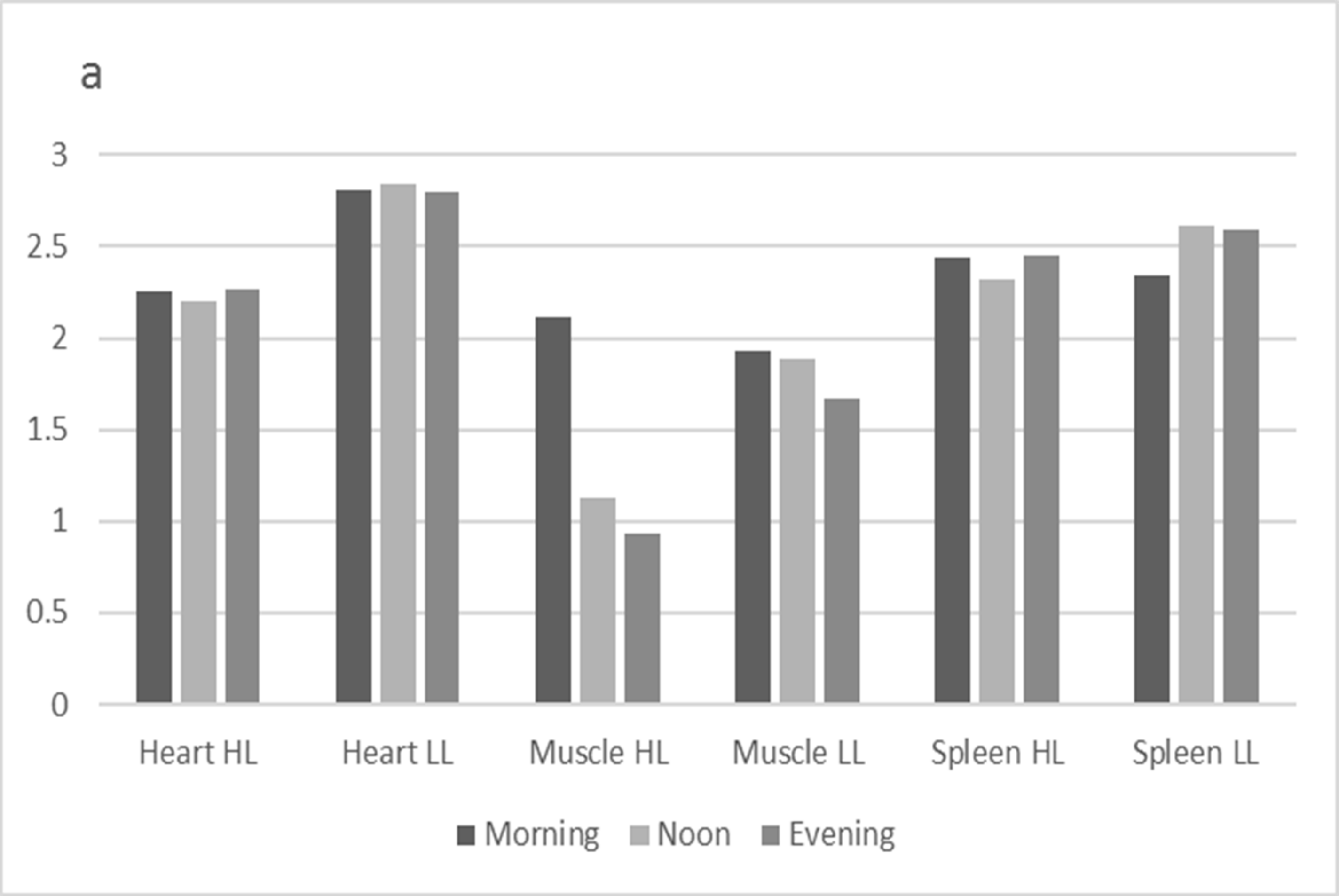
a
3
2.5
2
1.5
1
0.5
0
Heart HL
Heart LL
Muscle HL
Muscle LL
Spleen HL
Spleen LL
Morning
Noon
Evening

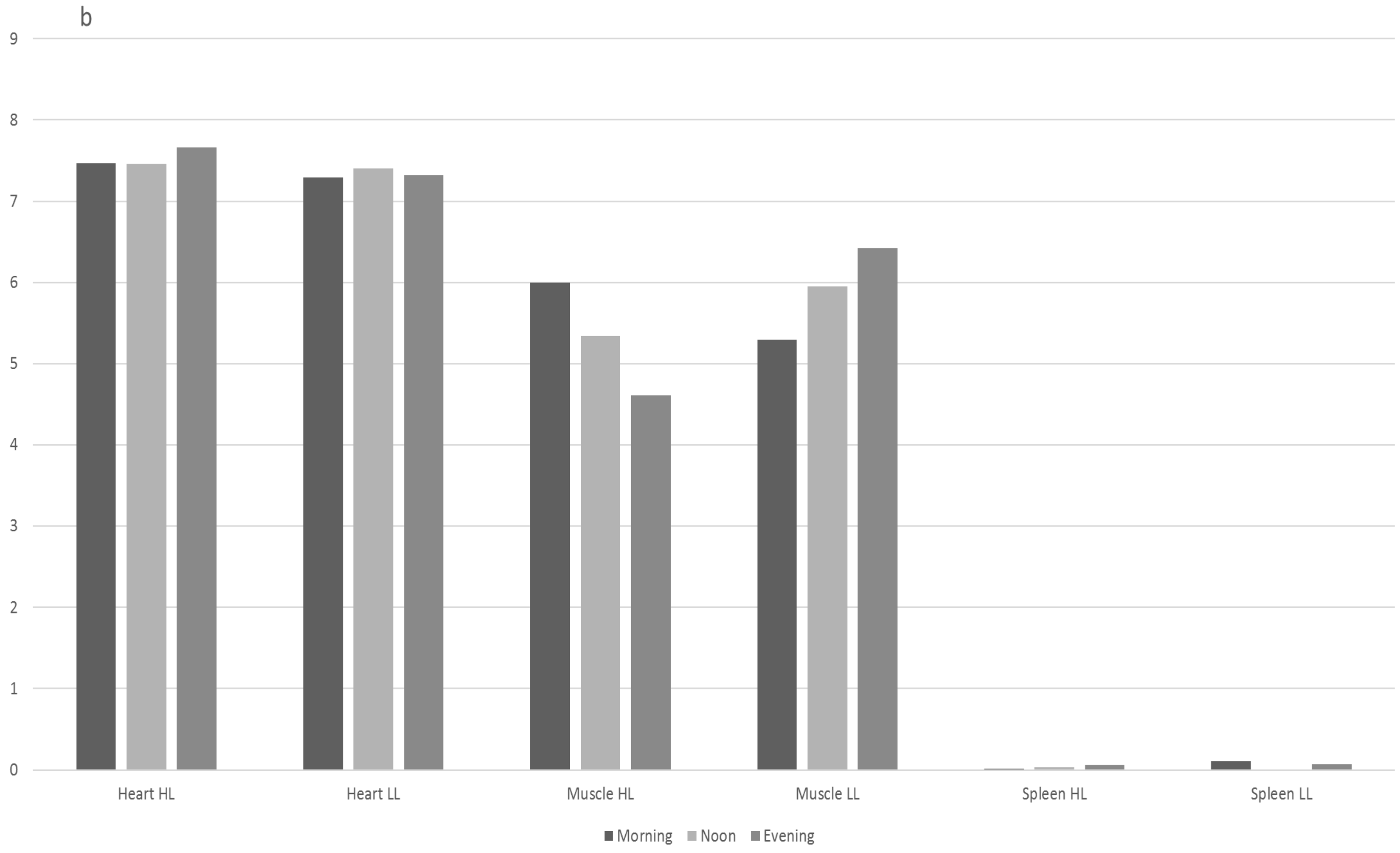
b
9
8
7
6
5
4
3
2
1
0
Heart HL
Heart LL
Muscle HL
Muscle LL
Spleen HL
Spleen LL
Morning
Noon
Evening

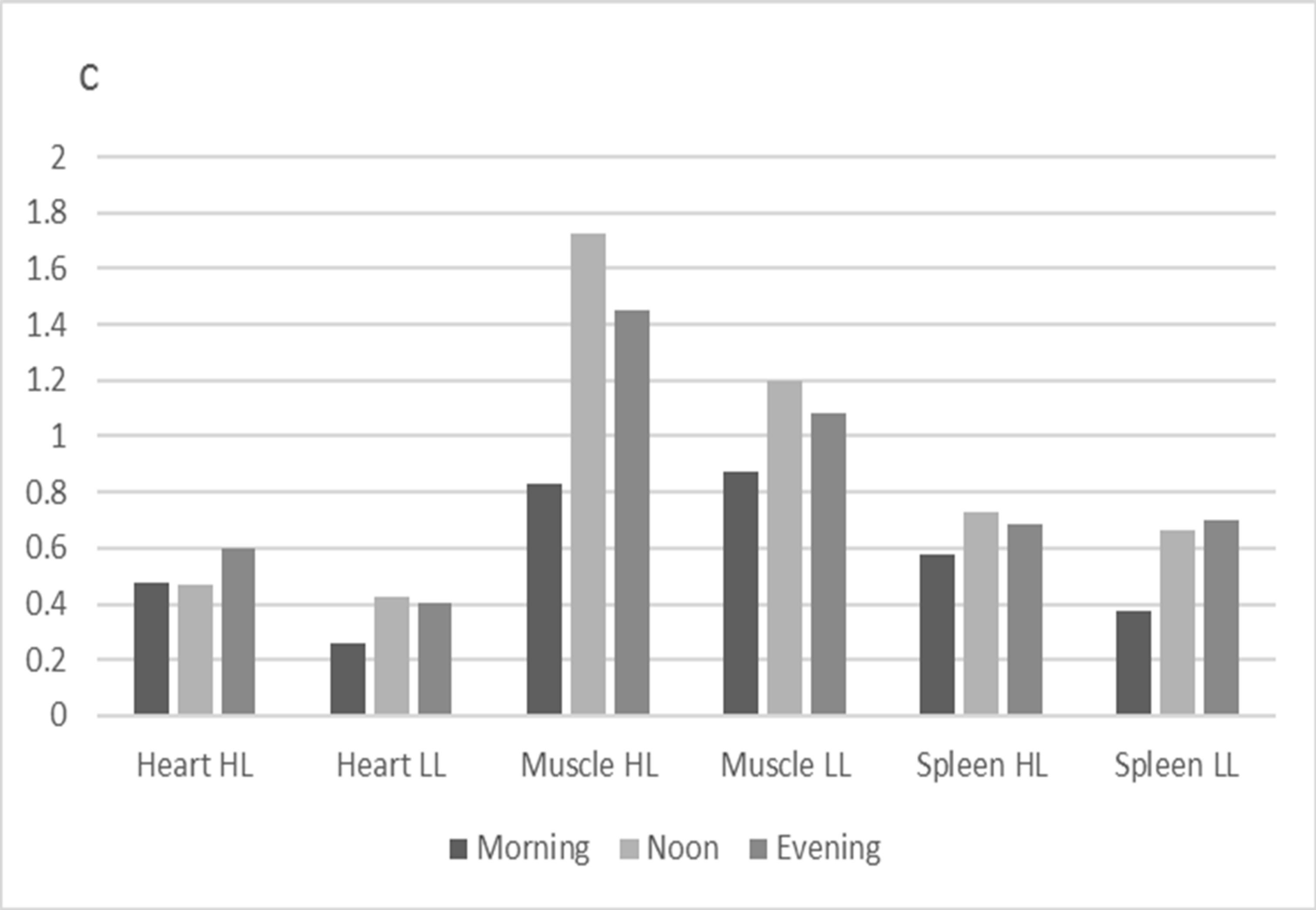
C
2
1.8
1.6
1.4
1.2
1
0.8
0.6
0.4
0.2
0
Heart HL
Heart LL
Muscle HL
Muscle LL
Spleen HL
Spleen LL
Morning
Noon
Evening

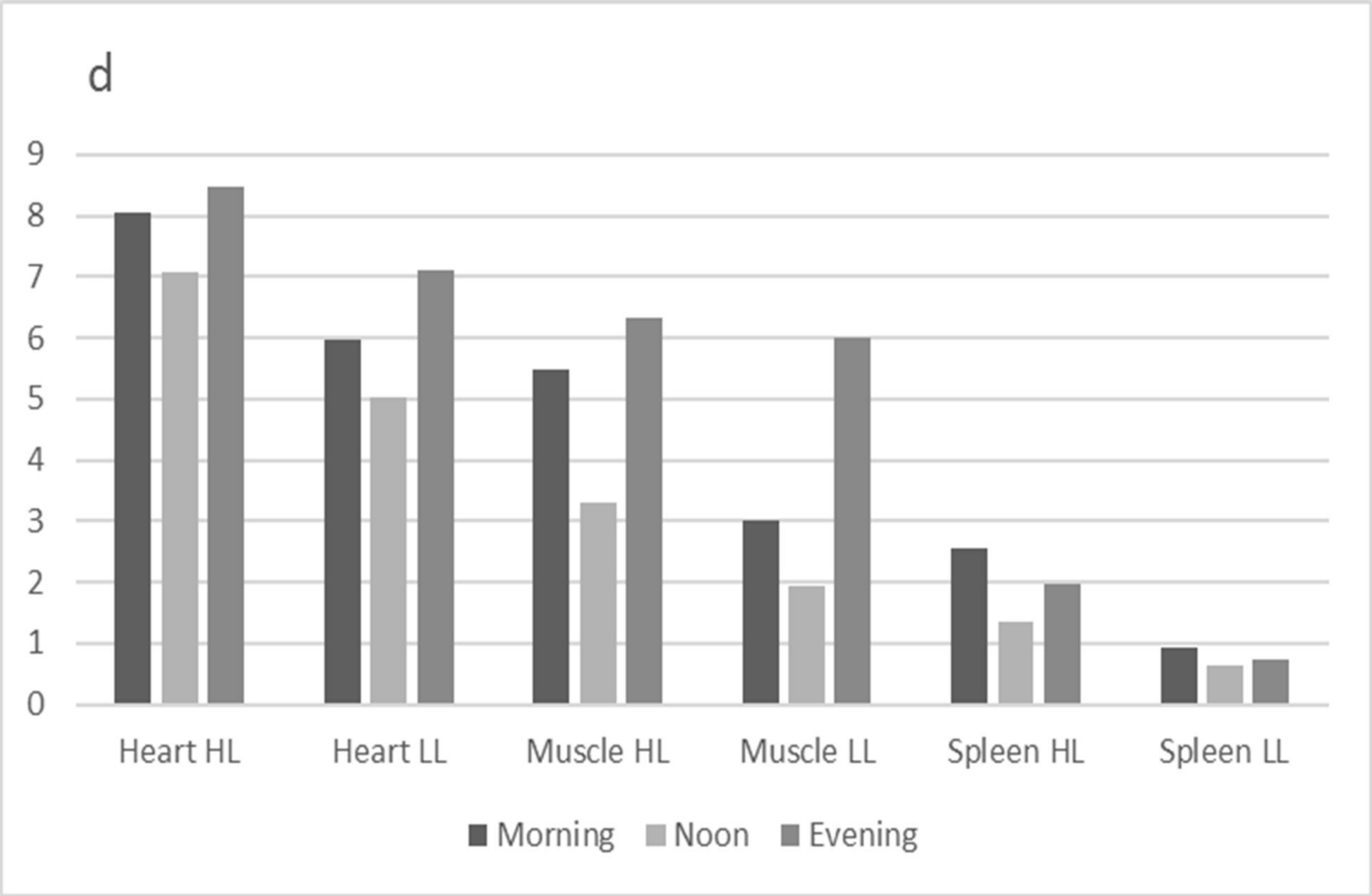
d
9
8
7
6
5
4
3
2
1
0
Heart HL
Heart LL
Muscle HL
Muscle LL
Spleen HL
Spleen LL
Morning
Noon
Evening

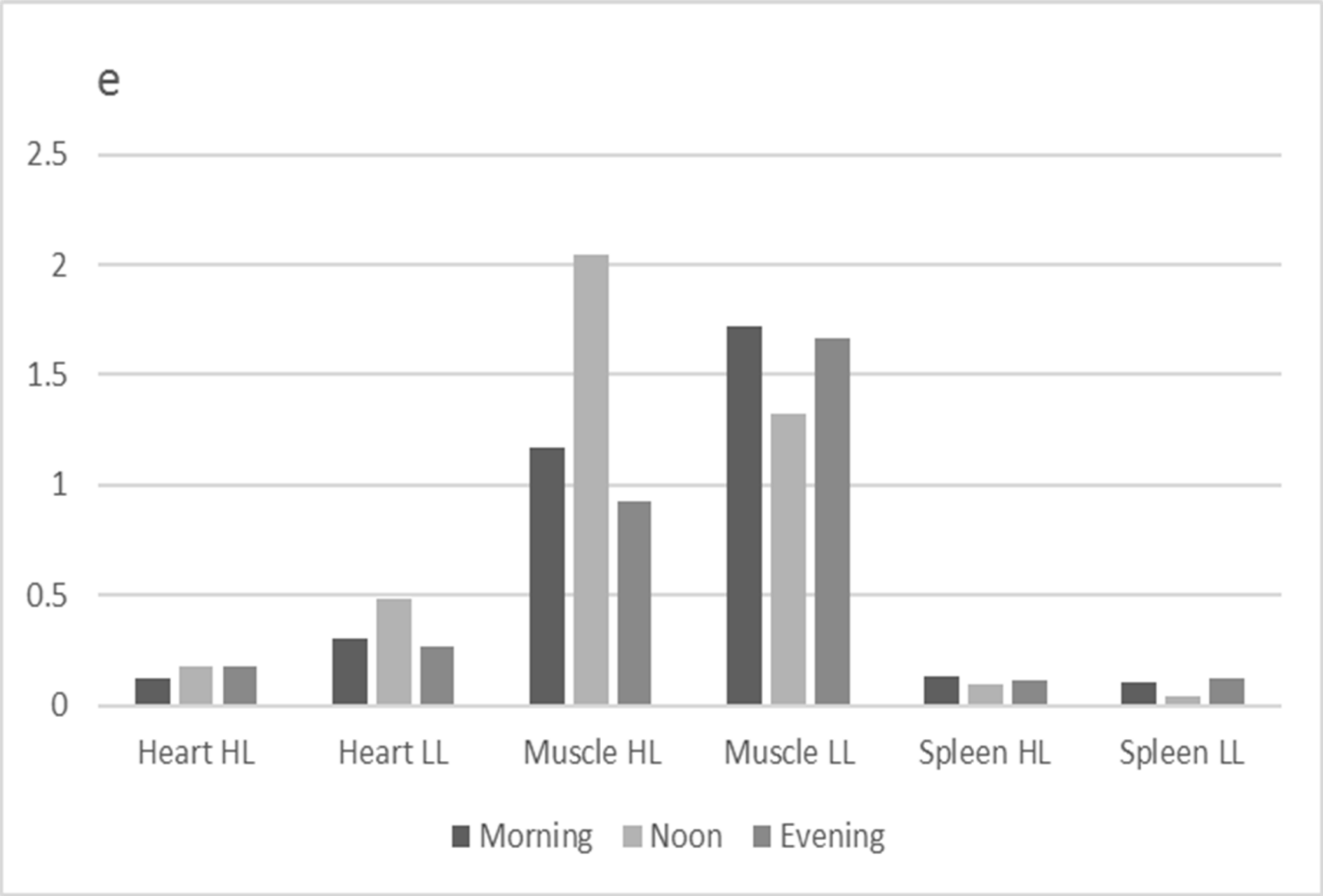
e
2.5
2
1.5
1
0.5
0
Heart HL
Heart LL
Muscle HL
Muscle LL
Spleen HL
Spleen LL
Morning
Noon
Evening

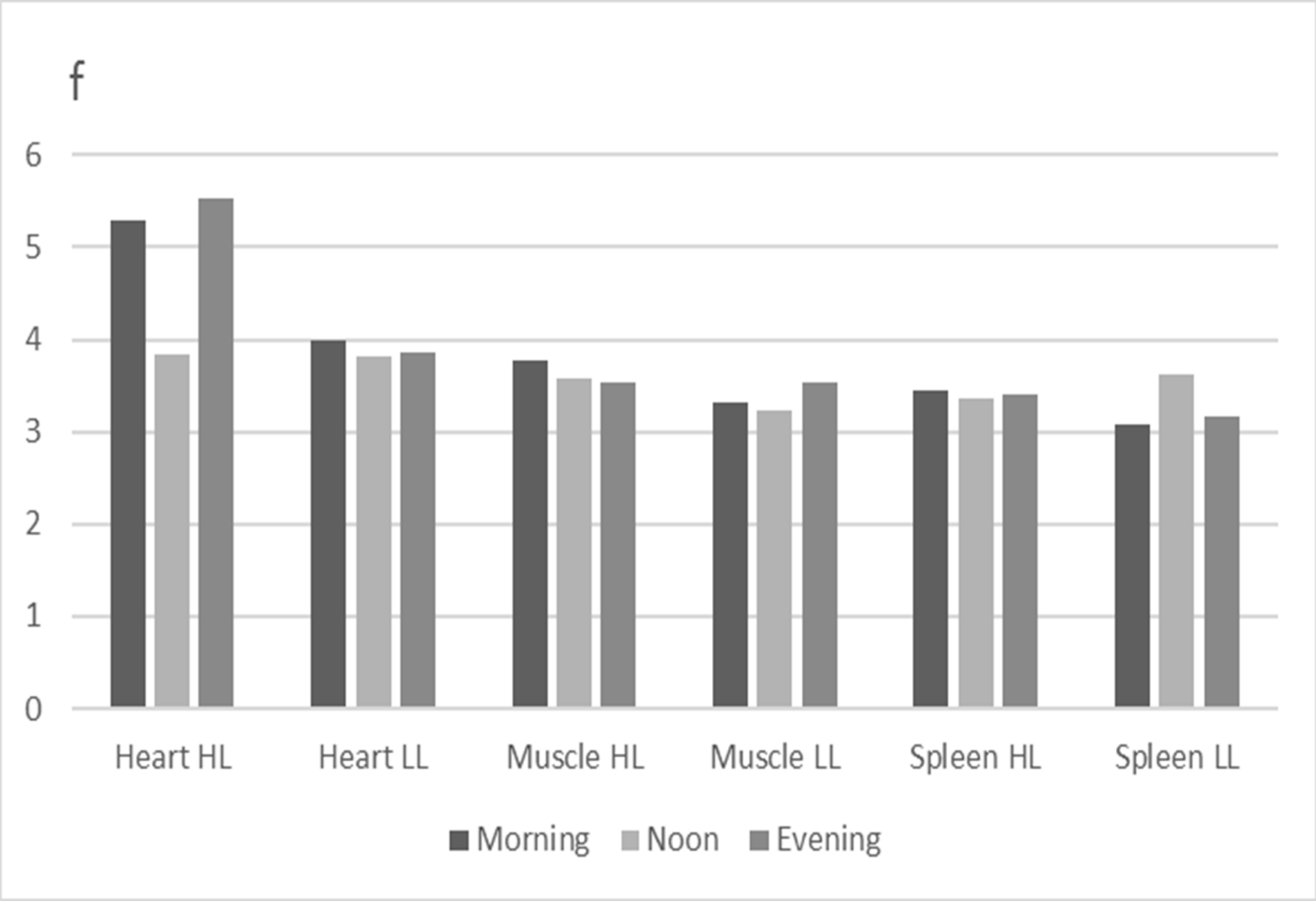
f
6
5
4
3
2
1
0
Heart HL
Heart LL
Muscle HL
Muscle LL
Spleen HL
Spleen LL
Morning
Noon
Evening

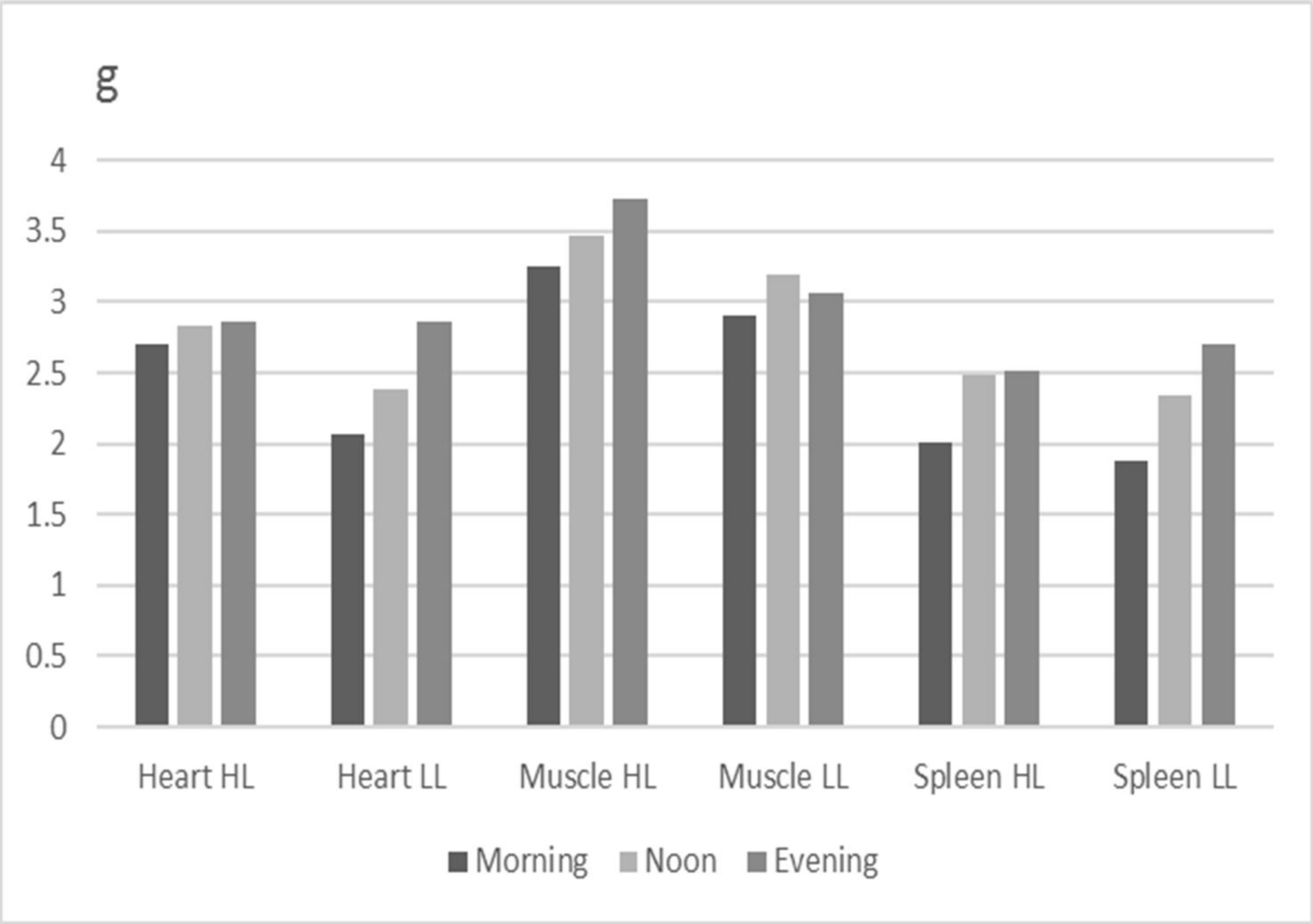
g
4
3.5
3
2.5
2
1.5
1
0.5
0
Heart HL
Heart LL
Muscle HL
Muscle LL
Spleen HL
Spleen LL
Morning
Noon
Evening

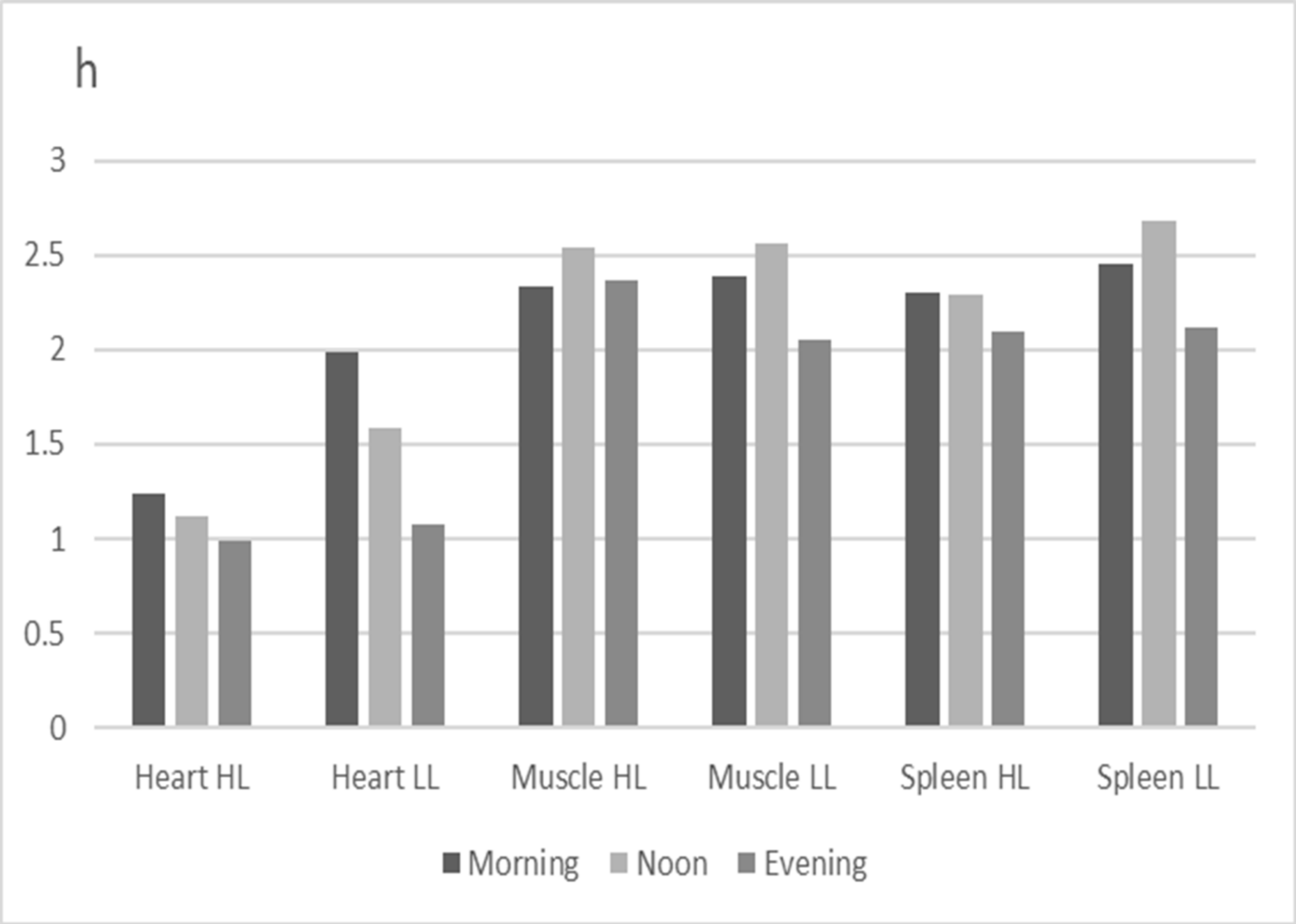
h
3
2.5
2
1.5
1
0.5
0
Heart HL
Heart LL
Muscle HL
Muscle LL
Spleen HL
Spleen LL
Morning
Noon
Evening

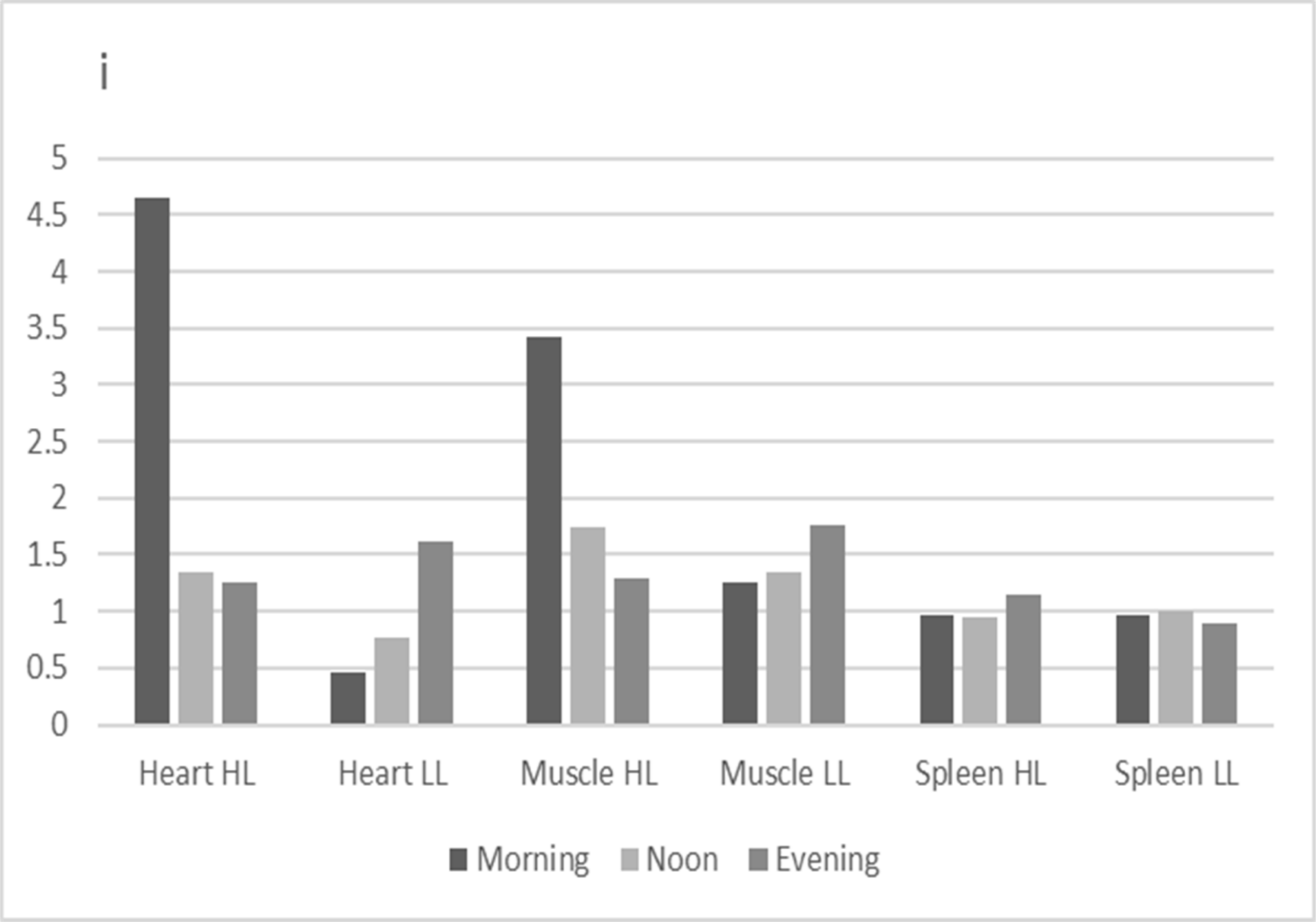
i
5
4.5
4
3.5
3
2.5
2
1.5
1
0.5
0
Heart HL
Heart LL
Muscle HL
Muscle LL
Spleen HL
Spleen LL
Morning
Noon
Evening

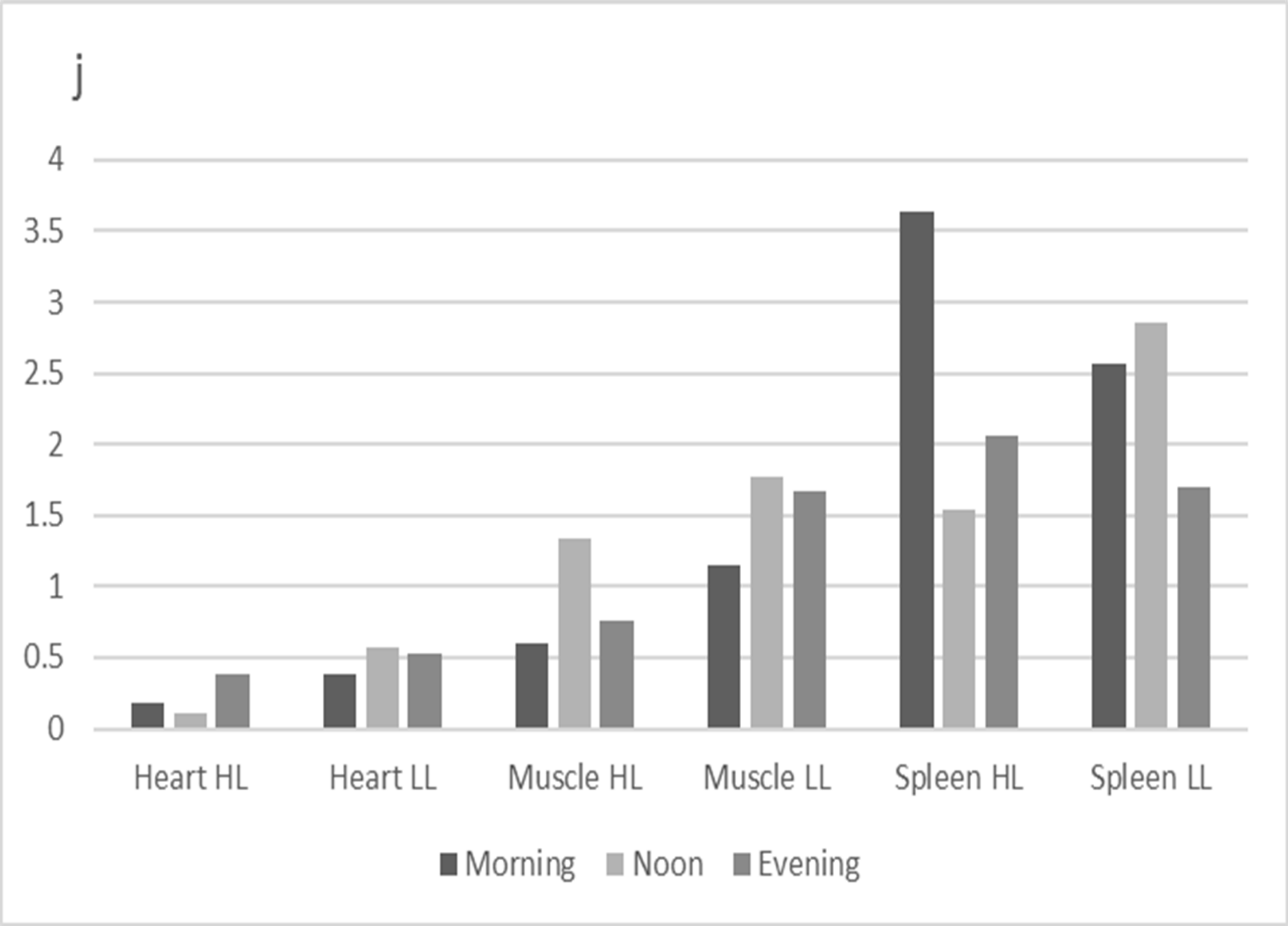
j
4
3.5
3
2.5
2
1.5
1
0.5
0
Heart HL
Heart LL
Muscle HL
Muscle LL
Spleen HL
Spleen LL
Morning
Noon
Evening

Table 1

The numbers of differentially expressed genes in three tissues of highland and lowland chicken analyzed at three time point during the day related to normal lowland circadian environmental temperatures. Number of differentially expressed genes as recognized by DAVID for analysis. The chicken (Gallus gallus, GG) EMSEMBLE numbers were converted by DAVID into human (Homo sapiens, HS) official gene symbols.

**Comparison**	**Heart**		**Muscle**		**Spleen**	
	**GG**	**HS**	**GG**	**HS**	**GG**	**HS**
**Highland vs. Lowland – all time points**	707	570	1900	1561	512	396
• **Morning**	376	319	304	250	364	290
• **Noon**	459	356	914	731	453	356
• **Evening**	287	222	945	766	273	194
**Highland – all time points**	946	715	1380	1102	2023	1575
• **Morning – noon**	366	274	551	426	965	788
• **Noon – evening**	369	272	467	375	575	415
• **Morning – evening**	372	290	653	536	990	780
**Lowland – all time points**	2248	1808	1265	1033	1143	869
• **Morning – noon**	297	210	548	455	409	318
• **Noon – evening**	805	659	474	379	316	232
• **Morning – evening**	1612	1319	463	375	571	433

Table 2

The numbers of functional annotations enriched using the differentially expressed genes (Table 1) in three tissues of highland and lowland chicken analyzed at three time point during the day related to normal lowland circadian environmental temperatures. GG: Gallus gallus (chicken), HS Homos sapiens (human).

**Comparison**	**Heart**		**Muscle**		**Spleen**	
	**GG**	**HS**	**GG**	**HS**	**GG**	**HS**
**Highland vs. Lowland – all time points**	0	246	61	897	5	153
• **Morning**	0	162	0	51	0	68
• **Noon**	0	87	14	335	1	143
• **Evening**	0	55	17	340	2	138
**Highland – all time points**	14	427	23	391	60	1342
• **Morning – noon**	21	359	2	223	67	1229
• **Noon – evening**	1	76	3	119	0	215
• **Morning – evening**	0	38	21	215	11	878
**Lowland – all time points**	37	823	19	339	6	336
• **Morning – noon**	0	15	22	235	1	133
• **Noon – evening**	12	293	0	148	1	89
• **Morning – evening**	40	834	0	181	7	254

Table 3

Functional annotations of network hub genes and nuclei created with STRING and STITCH software tools comparing Ethiopian highland and lowland chicken at three different time point during the day: in the morning, at noon, and in the evening measured under lowland conditions. Heart, (breast) muscle, and spleen tissues were analyzed. Functional annotations shared among two or three tissues are shown in italics. Metabolites appearing as central regulators in the networks are shown apart. Please note that several networks were too large to analyze due to large numbers of differentially expressed genes. These data are either missing or incomplete.

**Analysis group**	**Functional annotations protein hubs and nuclei in the networks, relevant metabolites**		
	**Heart**	**Muscle**	**Spleen**
**Highland vs. Lowland – all time points**	*Mitochondrion / Energy metabolism*<br>*Mitosis*<br>*Histone methylation*<br>*Circadian Rhythm*<br>Collagen<br>Histone deacetylation	Network too dense to analyze	*Mitochondrion / Energy metabolism*<br>*Mitosis / apoptosis*<br>*Histone methylation*<br>*Circadian Rhythm*<br>Ribosome<br>General metabolism<br>Immune mechanisms<br>Cell adhesion<br>Cellular components
	Calcium	Saturated Fatty Acids	*Calcium*<br>Dopamine
• **Morning**	*Mitosis / apoptosis*<br>*Circadian Rhythm*<br>*Histone deacetylation*<br>Transport<br>Insulin receptor mechanism	*Mitosis / apoptosis*<br>*Circadian Rhythm*<br>Immune<br>Angiogenesis<br>Ribosome / tRNA	*Mitosis / apoptosis*<br>*Histone deacetylation / methylation*<br>Protein transport<br>Ribosome<br>Transcription factor

	Immune	synthesis	expression<br>Protein metabolism<br>Signal transduction
• **Noon**	*Angiogenesis*<br>*Mitosis / apoptosis*<br>*Ribosome*<br>*Immune-related*<br>*Mitochondrion / energy metabolism*<br>*Molecular chaperone*	*Angiogenesis*<br>*Mitosis / apoptosis*<br>*Ribosome*<br>*Immune-related*<br>*Stress response*<br>*Splicing*<br>*Protein transport*<br>Collagen / ECM	*Angiogenesis*<br>*Mitosis / apoptosis*<br>*Ribosome*<br>*Immune*<br>*Mitochondrion / energy metabolism*<br>*Stress response*<br>*Chaperone / protein folding*<br>*Splicing*<br>*Protein transport / metabolism*
		Calcium	
• **Evening**	*Mitosis*<br>Angiogenesis<br>Splicing<br>Ribosome<br>Stress-fibers	*Mitosis*<br>*Mitochondrion*<br>Deacetylation<br>Immune<br>Signaling	*Mitosis / stress / apoptosis*<br>*Mitochondrion*<br>Cellular components / ECM
		Iron / Calcium	
**Highland – all time points**	Mitosis / apoptosis<br>Ribosome<br>Splicing<br>Angiogenesis<br>Heart muscle components	Network too dense to analyze	Network too dense to analyze

	Molecular chaperone<br>Stress / immune circadian rhythm		
• **Morning – noon**	*Circadian rhythm*<br>*Ribosome*<br>*Immune*<br>*Histone deacetylase*<br>Transcriptional silencing<br>*Mitosis / apoptosis*<br>*Molecular chaperone*	*Circadian rhythm*<br>*Ribosome*<br>*Immune*<br>*Histone deacetylase*<br>*Molecular chaperone* (cell and Mitochondrion)<br>Splicing (stress response?)<br>Cell adhesion / communication / ECM	*Molecular chaperone*<br>Protein metabolism<br>*Mitosis / apoptosis*
			Calcium
• **Noon – evening**	*Mitosis*<br>Histone<br>*Mitochondrion*<br>Heart functioning	*Mitosis / apoptosis*<br>*(De)acetylation / methylation*<br>*Splicing*<br>*Molecular chaperone*<br>*Immune*<br>Ribosome	*Mitosis / apoptosis*<br>*(De)acetylation*<br>*Energy metabolism*<br>*Splicing / mRNA metabolism*<br>*Molecular chaperone*<br>*Immune processes*<br>Protein metabolism / Transport<br>Transcription factor expression<br>Angiogenesis<br>Cell adhesion<br>Signal transduction

	Dopamine		
• **Morning – evening**	*Splicing / RNA processing*<br><br>*Mitosis*<br>*Ribosome*<br>Chaperone<br>Angiogenesis<br>Gap junction (synchronized contraction of the heart)	*Splicing*<br>*Mitochondrion / energy metabolism*<br>*Mitosis*<br><br>(De)acetylation / methylation<br>Adherence / ECM	 *Mitochondrion*<br><br><br>*Ribosome*<br>Angiogenesis / Lymphatic vasculature development<br>Chromatin remodeling
	Dopamine	Iron<br>Organosulfer	
**Lowland – all time points**	Deacetylase<br>Saturated fatty acids<br>Cardiovascular disease associated	Ribosome	Network too dense to analyze
• **Morning – noon**	*Mitosis*<br>*Angiogenesis*<br><br>Mitochondrion	*Mitosis / apoptosis*<br><br>*Splicing*<br>Ribosome<br>Acetylation / methylation / (telomerase?)<br>Expression regulation<br>Adipose tissue development	*Mitosis / apoptosis*<br>*Angiogenesis*<br>*Splicing*<br>Integration of energy metabolism and metabolism<br>Cholesterol metabolism<br>Stress activated<br>Circadian rhythm / clock<br>Immune<br>Chaperone (heat shock induction)
			Calcium
• **Noon – evening**	*Mitosis / apoptosis*	*Mitosis / apoptosis*	*Down regulation of apoptosis*<br>*Energy metabolism*

	Mitochondrion<br>*Fatty acid biosynthesis*<br>*Circadian rhythm*<br><br>Immune	 <br><br><br>*Ribosome (synthesis)*<br>Angiogenesis<br>Protein transport	*Fatty acid biosynthesis*<br><br>*Circadian rhythm*<br>*Ribosome*<br>Splicing<br>Transcription factors
• **Morning – evening**	*Mitochondrion*<br>*Fatty acid beta oxidation*<br>*Mitosis / apoptosis*<br>*Stress response (high blood pressure)*	*Energy metabolism*<br><br><br>*Mitosis / apoptosis*<br>*Stress response*<br>*Splicing*<br>*Ribosome*<br>Immune<br>Transport of proteins<br>Circadian rhythm<br>General metabolism Cell adhesion	*Mitochondrion / Energy metabolism / cholesterol metabolism*<br>*Mitosis / apoptosis (less)*<br><br>*Splicing*<br>*Ribosome*<br>Angiogenesis

Table 4

Analysis of groups of physiological pathways comparing Ethiopian highland and lowland chicken at three different time point during the day under the lowland environmental conditions: in the morning, at noon, and in the evening of heart (A), (breast) muscle (B), and spleen (C) tissues. All groups larger than two pathways are shown, with groups containing three pathways are shown in grey. Please note that several datasets were too large to analyze due to large numbers of differentially expressed genes. To solve this the software was set to maximum specificity, leading to less results. These analyses are marked with an asterisk.

A: Heart

**Animal Groups**	**Major pathways - groups of pathways**
**Highland vs. Lowland – all time points**	ATP synthesis coupled transport; Coenzyme biosynthetic process; Cellular carbohydrate catabolic process - Positive regulation of B cell activation; Positve regulation of proteasomal protein catabolic process - cGMP-PKG signaling pathway - Negative regulation of muscle contraction; Negative regulation of muscle hypertrophy; Myoblast differentiation - Regulation of phospholipase C activity; Cognition - Rap1 signaling; B cell receptor signaling pathway; Chronic myeloid leukemia; Cytokine production involved in immune response / Regulation of fat cell differentiation; Positive regulation of fat cell differentiation; Asymmetric cell division / Inner ear receptor cell development; establishment of planar polarity / DNA strand elongation / Neutral lipid process; Glycerolipid metabolism / Microtubule-based mocement; Cilium movement / Sulfur amino acid metabolic process / Glycosphigolipid metabolic process / Collagen fibril organization / Mitochondrial translation initiation / Lipid storage / Negative regulation of transmembrane transport / Rho GTPase cycle / Regulation of RNA stability / Negative regulation of G-protein coupled receptor protein signaling pathway
morning	Associative learning / Sister chromatid cohesion / Epithelium cilium movement involved in determination left-right asymmetry / Cardiac muscle cell development
noon	Toll-like receptor TLR1-TLR2 signaling pathway; Lymphocyte homeostasis; Positive regulation of lipid metabolic process / Regulation of membrane repolarization / Positive regulation of cAMP biosynthetic process / Heart looping / Cell cycle DNA replication / Trans-Golgi network vesicle budding / Protein targeting to vacuole / GPI anchor biosynthetic process

evening	Processing of capped intronless pre-mRNA / Activation of matrix metalloproteinases / Assembly of collagen fibrils and other multimeric structures / epithelial cell morphogenesis
**Highland * – all time points**	mRNA catabolic process
morning-noon	mRNA processing / Nuclear transcribed mRNA catabolic process exonucleolytic; cell-cell junction organization; Regulation of systemic arterial blood pressure; Oxygen transport
noon-evening	Response to auditory stimulus / Regulation of adaptive immune response based on somatic recombination of immune receptors built from immunoglobulin superfamily domains / RHO GTPases Activate Formins / Regulation of potassium transport / Regulation of transcription from RNA polymerase II promoter / Lens morphogenesis in camera-type eye / Adherens junction / Cellular response to glucose starvation
morning-evening	Unfolded protein response (UPR); RNA surveillance / Cell-cell jnuction assembly / Negative regulation of circadian rhythm
**Lowland * – all time points**	Positive regulation of metabolic process; Positive regulation of molecular function; Protein modification process / Organelle organization / Cell cycle
morning-noon	Coenzyme biosynthetic process / Arachidonic acid metabolism / Mesonephric tubule morphogenesis / Regulation of phospholipase C activity / Cellular component disassembly involved in execution phase of apoptosis
noon-evening *	Regulation of early endosome to recycling endosome transport / 3'-UTR mediated mRNA destabilization / Coenzyme A catabolic process / Optic placode formation involved in camera-type eye formation / Amniotic stem cell differentiation / Vagus nerve morphogenesis / L-ascorbic acid biosynthetic process / Negative regulation of cellular amino acid metabolic processes / Signal transduction involved in intra-S DNA damage checkpoint / Nuclear mRNA surveillance of mRNA 3'-end processing / Negative regulation of interleukin-6-mediated signaling pathway

morning-evening *	Regulation of interleukin-4 biosynthetic process; Interleukin-4 biosynthetic process; primitive erythrocyte differentiation - Negative regulation of histone H3-K4 methylation; Negative regulation of centrosome duplication - Skeletal muscle thin filament assembly / Negative regulation of filopodium assembly / Activation of meiosis involved in egg activation / Regulation of DNA templated transcription termination / 3'-UTR mediated mRNA destabilization / Lateral mesoderm cell fate commitment / Geranyl diphosphate metabolicprocess / Coenzyme A catabolic process / Positive regulation of sequestering of calcium ion / Amniotic stemm cell differentiation / Regulation of protein tetramerization / Coenzyme A transport / Heme A biosynthetic process / Negative regulation of uterine smooth muscle contraction / protein processing in phagocytic vesicle / Positive regulation of protein deubiquitination / Histone H3-K27 acetylation / Memebrane depolarization during AV node cell action potential / diadenosine polyphosphate metabolic process / Transcription initiation from RNA polymerase I promoter large rRNA transcript

B: Muscle

**Animal Groups**	Major pathways - groups of pathways
**Highland vs. Lowland * – all time points**	Beta-alanine biosynthetic process; Negative regulation of vascular endothelial growth factor signaling pathway / Cell migration involved in metanephros development; Medium chain fatty acid transport; Phosphatidylserine biosynthetic process / Regulation of centromeric sister chromatid cohesion / VEGF-activated neuropilin signaling pathway / Regulation of membrane depolarization during cardiac muscle cell action potential / Acetate metabolic process / Regulation of sulfur amino acid metabolic process / Regulation of telomere maintenance via semi-conservative replication / Dipeptide membrane transport / Activation of meiosis involved in egg activation / Positive reguation of centrosome duplication / Cervix development / Glycosyl ceramide biosynthetic process / Negative regulation of skeletal muscle cell differentiation / Negative regulation of eosinophil activation / N-terminal protein palmitoylation / Lysine import / Coenzyme A transport / Positive regulation of TRAIL production / Wnt signaling pathway involved in digestive tract morphogenesis / UDP-N-acetylglucose transport / Regulation of intrinsic apoptotic signaling pathway in response to osmotic stress by p53 class mediatior / Negative regulation of glucocorticoid mediated signaling pathway / Cytoplasmic translational elongation / Elastin biosynthetic process / Transcription initiation from RNA polymerase I promoter for nuclear large rRNA transcript

morning *	Negative regulation of proteasome ubiquitin-dependent protein catabolic process / Glycerophospholipid biosynthesis / Cellular carbohydrate catabolic process / protein O-linked glycosylation
noon *	Negative regulation of filopodium assembly / Dipeptide transmembrane transport / Cervix development / Induction by symbiont of host defense response / Endosome to lysosome transport via multivesicular body sorting pathway / Regulation of microglial cell activation / Regulation of protein import into mitochondrial outer membrane / Coronary vein morphogenesis / Negative regulation of nucleobase-containing compound transport / Regulation of neutrophil apoptotic process / Regulation of natural killer cell cytokine production / Succinate transport
evening *	Mitochondrial RNA 3'-end processing / Limb joint morphogenesis / N-terminal amino acid methylation / Reversal of alkylation damage by DNA dioxygenases / Lysine import / Coronary morphogenesis / Positive regulation of TRAIL production / Positive regulation of Schwann cell differentiation / Atrial ventricular junction remodelling / TIRAP-dependent toll-like receptor signaling pathway / Negative regulation of phosphatidylinositol biosynthetic process / Regulation of intrinsic apoptotic signaling pathway response to osmotic stress by p53 class mediator / CVT pathway/ Negative regulation of glucocorticoid mediated signaling pathway / Elastin biosynthetic process / Acetate biosynthetic process / Transcription initiation from RNA polymerase I promoter for nuclear large rRNA transcript
**Highland * – all time points**	Canonical Wnt signaling pathway involved in positive regulation of epithelial to mesenchimal transition / Induction by symbiont of host defense response / Regulation of DNA templated transcription termination / Mitochondrial RNA surveillance / ... / Vagus nervus morphogenesis
morning-noon	Blood vessel endothelial cell migration; positive regulation of cellular carbohydrate metabolic process - Signaling by NOTCH1; Primary neural tube formation; Hormone biosynthetic process; Retinol metabolic process - Protein deacetylation; Alpha linolenic acid (ALA) metabolism / Regulation of interleukin 12 biosynthetic process; Interleukin 12 biosynthetic process / GABA synthesis, release, reuptake and degradation / Dolichol-linked oligosaccharide biosynthetic process / Protein homotetramerization / DNA-templated transcription termination / Histone H4 acetylation / IRE1-mediated unfolded protein response / Termination of G-protein coupled receptor signaling pathway
noon-evening	Positive regulation of viral transcription / Proteasome maturation / Regulation of stress fiber assembly / Negative regulation of G-protein coupled receptor protein signaling pathway / Vitamine transport / Organophosphate ester transport / rRNA processing

morning-evening	Retrograde neurotrophin signaling; DNA-templated transcription elongation; Transcription elongation from RNA polymerase II promoter; Ca2+ pathway - Regulation of epithelial cell differentiation - Morphogenesis of embryonic epithelium - Regulation of erythrocyte differentiation; Positive regulation of peptide hormone secretion; Unsaturated fatty acid biosynthetic process; PPAR signaling pathway - Mitotic spindle organization; Positive regulation of proteasomal ubiquitin-dependent protein catabolic process; Negative regulation of proteasomal protein catabolic process
**Lowland * – all time points**	Positive regulation of branching involved in lung morphogenesis / Negative regulation of sequestering of triglyceride / Histone H3-K36 trimethylation / Positive regulation of centrosome duplication / Regulation of blood vessel endothelial cell proliferation involved in sprouting angiogenesis / Induction by symbiont of host defense response / Regulation of DNA-templated transcription termination / Marginal zone B cell differentiation / Coronary vein morphogenesis / Negative regulation of nucleobase-containing compound transport / Regulation of neutrophil apoptotic process / Cadmium ion homeostasis / Golgi to plasma membrane CFTR protein transport / L-ascorbic acid biosynthetic process / Xanthine metabolic process / CVT pathway / Negative regulation of phospholipase C activity / Succinate transport / Negative regulation of intestinal phytosterol absorption / Transcription initiation from RNA polymerase I promoter for nuclear large rRNA transcript
morning-noon	Photoperiodism; PPARA activates gene expression; Glycerolipid metabolism / Ribonucleoprotein complex biogenesis / Regulation of double-strand break repair via homologous recombination / Signal transduction involved in cell cycle checkpoint / eye morphogenesis / Cellular response to mechanical stimulus / response to vitamin / Transcription elongation from RNA polymerase I promoter / Histone methylation / Positive regulation of stress-activated MAPK cascade / Recruitment of mitotic centrosome proteins and complexes / Regulation of mRNA stability / Positive regulation of leukocyte chemotaxis / Pre-NOTCH expression and processing / Cellular monovalent inorganic cation homeostasis
noon-evening	Male gonad development; Regulation of response to extracellular stimulus - Blood vessel endothelial cell proliferation involved in sprouting angiogenesis - Negative regulastion of DNA biosynthetic process; Positive regulation of stem cell proliferation ; Negative regulation of nuclear division (with muscle pathways!); Neural tube patterning / positive regulation of axon extension / Glycolysis + Gluconeogenesis / SUMOylation of DNA damage response and repair proteins / response to ionizing radiation

morning-evening	Glutamate receptor signaling pathway; Oxytocin signaling pathway - Adipocytokine signaling pathway - Negative regulation of mononuclear cell proliferation; Regulation of mesenchymal cell proliferation; mesenchymal cell proliferation - TGF-beta signaling pathway; Embryonic digit morphogenesis; Osteoblast proliferation - Shigellosis / DNA-templated transcription termination; mRNA3'-end processing / Response to ammonium ion/ NRAGE signals death through JNK / Glycerolipid metabolism / Centrosome cycle / Mitotic G1-G1S phases / Regulation of plasma membrane organization

C: Spleen

**Animal Groups**	Major pathways - groups of pathways
**Highland vs. Lowland – all time points**	Detection of mechanical stimulus involved in sensory perception of sound; Response to mechanical stimulus; Neuroactive ligand-receptor interaction; Associative learning - Monovalent inorganic cation homeostasis; Hormone biosynthetic process / Lymphocyte homeostasis / Response to acid chemical / Entry into host cell / Positive regulation of Rho protein signal transduction
morning	Maintenance of protein location in cell; Regulation of chromosomal segregation; Anaphase-promoting complex-dependent proteasomal ubiquitin-dependent protein catabolic process; E2F mediated regulation of DNA replication / Protein processing in endoplasmic reticulum / Regulation of fibroblast proliferation
noon	Organelle biosynthesis and maintenance; Chromosome segregation - Anchoring of the basal body to the plasma membrane / Negative regulation of protein localization to nucleus / Trans-Golgi network vesicle budding / Mitotic spindle organization / Regulation of mRNA splicing, via spliceosome
evening	...... Too small to mention any .....

**Highland * – all time points**	Regulation of glycolytic process by regulation of transcription from RNA polymerase II; Regulation of platelet-derived growth factor receptor-alpha signaling pathway / Interleukin 4 biosynthetic process / Regulation of phosphatidylcholine biosynthetic process / Maintenance of mitotic sister chromatid cohesion / Positive regulation of ER to Golgi vesicle-mediated transport / Protein localization to nonmotile primary cilium / Tongue muscle cell differentiation / Negative regulation of eosinophil activation / Positve regulation of oocyte development / Dosage compensation by inactivation of X chromosome / Spermidiane catabolic process / Positive regulation of aldosterone metabolism / Mitochondrial mRNA catabolic process / Regulation of protein import into mitochondrial outer membrane / Positive regulation of chromatin silencing / Cerebellar cortex structural organization / Proline catabolism / Protein localization to vacuolar membrane / Synaptic vesicle uncoating / Response to biotin / Receptor mediated virion attachement to host cell / Dolichol metabolic process
morning-noon	PI metabolism; Phosphatidylinositol dephosphorylation - Steroid hormone secretion; C21 steroid hormone biosynthetic process - Regulation of receptor biosynthetic process - Pre-NOTCH expression and processing; Signaling by NOTCH - ARMS-mediated activation; Golgi cisternae pericentriolar stack reorganization; condensation of prometaphase chromosomes; Nuclear envelope breakdown; M phase; mRNA transport; Transcription organization from RNA polymerase III promoter; Cytosolic sensors of pathogen-associated DNA; TRAF3-depedent IRF activation pathway; BBsome-mediated cargo-targeting to cilium; Folding of actin by CCT/TriC;- Mitotic prometaphase (close to M phase); RHO GTPases activate formin; Mitotic sister chromatic segregation; Cell cycle; Cell cycle checkpoints; Regulation of spindle organization; Histone monoubiquitination; Histone H3 acetylation; Peptidyl-lysine methylation; Regulation of PLK1 activity at G2/M trnasition; Regulation of protein phosphatase type 2A activity / Anterograde axon cargo trasport / Thromboxane signaling through TP receptor / Cochlea morphogenesis / Methionine metabolic process / Mitochondrial transdlation termination / Cytoplasmic mRNA processing body assembly / COPII-coated vesicle budding / RNA destabilization / Negative regulation of DNA recombination / Protein K63-linked deubiquitination / C-protein coupled purinergic nucleotide receptor signaling pathway
noon-evening	Interleukin 4 signaling; Prolactin signaling pathway / NOTCH intracellular domain regulates transcription / Protein localization to microtubuli cytoskeleton / Postreplication repair / trans Golgi network vesicle budding / NLS-bearing protein import into nucleus / Positive regulation of chromatin

	modification / Cytoskeleton-dependent intracellular transport
morning-evening *	SHC-related events triggered by IGF-1R; Regulation of anoikis / Nucleotide excision repair / Regulation of water loss via skin; conversion from APC/C:Cdc20 to APC/C:Cdh1 in late anaphase; APC-Cdc20 mediated degradation of NEK2A / Cerebral cortex neuron differentiation; Regulation of phosphatidylcholine biosynthetic process / Mitotic chromosome condensation / Nucleotide-sugar metabolic process / Protein export / Transport of the SLBP-independent mature mRNA / Terpenoid backbone biosynthesis / Negative regulation of protein dephosphorylation / Phospholipase C-activating dopamine receptor signaling pathway / Positive regulation of RNA splicing / S-adenosylmethionine cycle / Huntington's disease / CDP-diacylglycerol biosynthetic process / ER to Golgi vesicle-mediated transport / Mitochondrial ATP synthesis coupled proton transport / CBP1(S) activates chaperone genes / SNARE complex assembly / Mitotic prometaphase / Regulation of hypoxia-inducible factor (HIF) by oxygen / Aorta morphogenesis / Centriole replication
**Lowland * – all time points**	Negative regulation of osteoclast differentiation; Copper ion homeostasis; Microtubule cytoskeleton organization involved in mitosis; Regulation of centriole elongation; heart formation; Spermatic nucleus differentiation; Autophagic vacuole assembly; CVT pathway - Subpallium development; Anterograde axon cargo transport; Cochlea morphogenesis; DNA hypermethylation; Positive regulation of transcription from RNA polymerase II promoter in response to stress; Sphingolipid catabolic process - Positive regulation of peptidyl-threonine phosphorylation; Mitochondrial biosynthesis; Pre-NOTCH expression and processing; Retrograde transport, endosome to Golgi - FOXO signaling pathway; Oocyte maturation / Circadian regulation of translation / Transcription from RNA polymerase I promoter / Protein refolding / Vitamin digestion and absorption / Cellular response to heat stress; Mitochondrial translation initiation / Regulation of T-helper 1 type immune response / Regulation of fatty acid beta-oxidation / Regulation of intracellular steroid hormone receptor signaling pathway / Cholesterol biosynthesis / Glycosylphosphatidylinositol (GPI)-anchor biosynthesis / Energy coupled proton trnasmembrane transport, afgainst electrochemical gradient / Positive regulation of signal transduction by P53 class mediator / Metabolism of porphyrins / SUMOyl DNA damage response and repair process / Phosphatidylethanol metabolic process / Negative regulation of insulin secretion / Embryonic placenta morphogenesis / G-protein coupled

	purinergic receptor signaling pathway / Positive regulation of receptor internalization / Synaptic vesicle fusion to presynaptic membrane
morning-noon	Interleukin-17 production; Regulation of interleukin-17 production / Mitotic metaphase plate congression / Cholesterol biosynthesis / Fructose and mannose metabolism / Neutral lipid biosynthetic process
noon-evening	Mitochondrial translation / Nucleoside triphosphate biosynthetic process
morning-evening *	Regulation of centrosome cycle; Cytoplasmic microtubule organization; Regulation of proteasomal ubiquitin-dependent protein catabolic process; PI3K events in ERBB3 signaling; PI3K events in ERBB4 signaling; Antigen activates B cell receptor (BCR) leading to generation of second messengers; Prostate cancer; Negative regulation of cellular response to oxidative stress; Synthesis of PE - AKT phosphorylates targets in the nucleus; Negative regulation of smooth muscle differentiation - Organelle transport along microtubule / ER to Golgi vesicle-mediated transport / Protein localization to kinetochore / Regulation of intracellular steroid hormone receptor signaling pathway; GPI anchor metabolic process / Mismatch repair / neural tube closure
