# Supplementary figures and images for: Transcriptomic profiles of muscle, heart, and spleen in reaction to circadian heat stress in Ethiopian highland and lowland male chicken (part 2 of 2)
Source: Cell Stress Chaperones. 2018 Dec 18;24(1):175–94. doi: 10.1007/s12192-018-0954-6 (PMC6363629; doi:10.1007/s12192-018-0954-6)

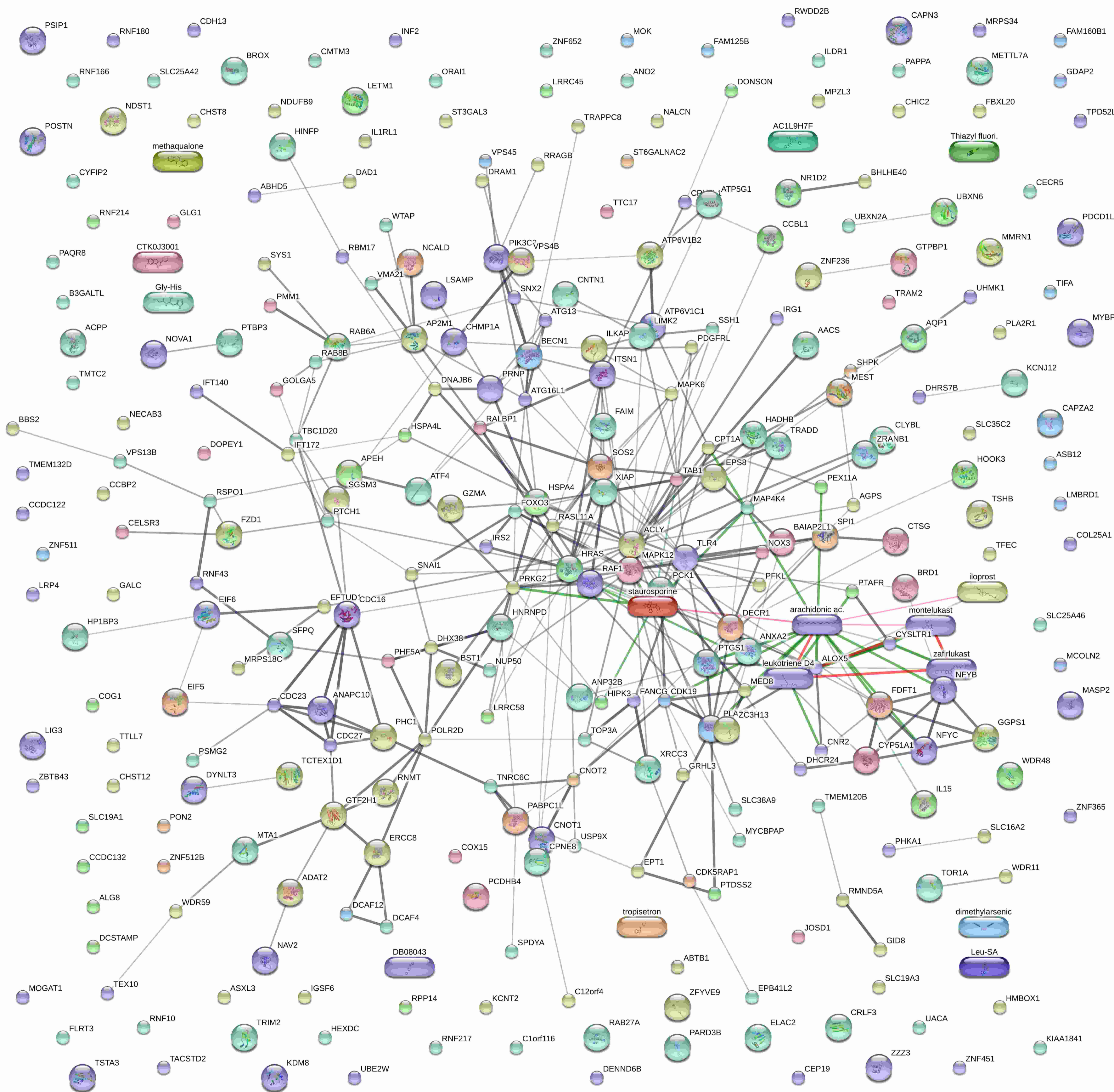

Supplement: Supplementary file 6 — Network analysis figures. All figures were converted to pdf files. (ZIP 47344 kb) [file 12192_2018_954_MOESM6_ESM.zip › Spleen Lowland morning-noon - stitch.pdf]

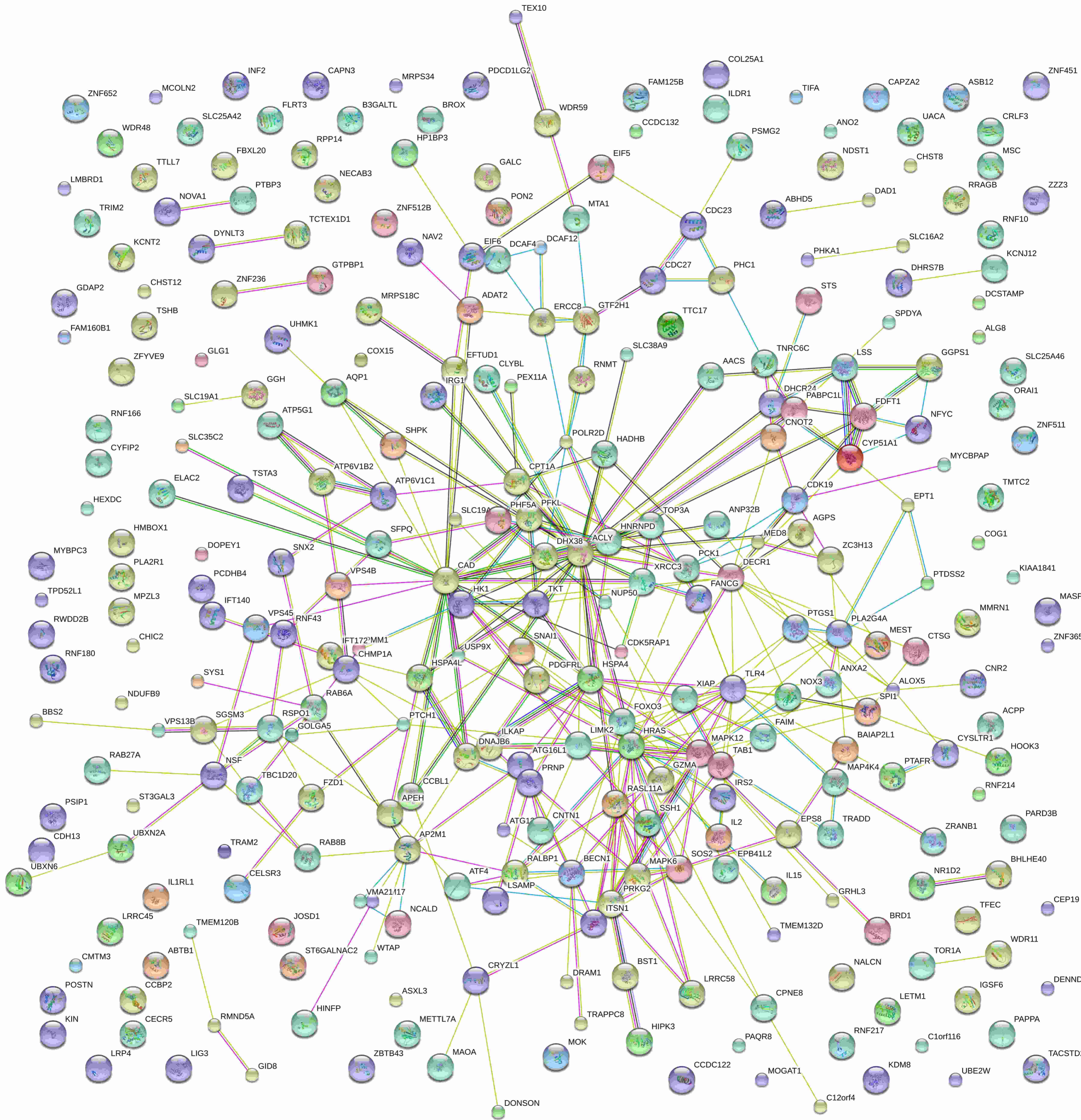

Supplement: Supplementary file 6 — Network analysis figures. All figures were converted to pdf files. (ZIP 47344 kb) [file 12192_2018_954_MOESM6_ESM.zip › Spleen Lowland morning-noon - string.pdf]

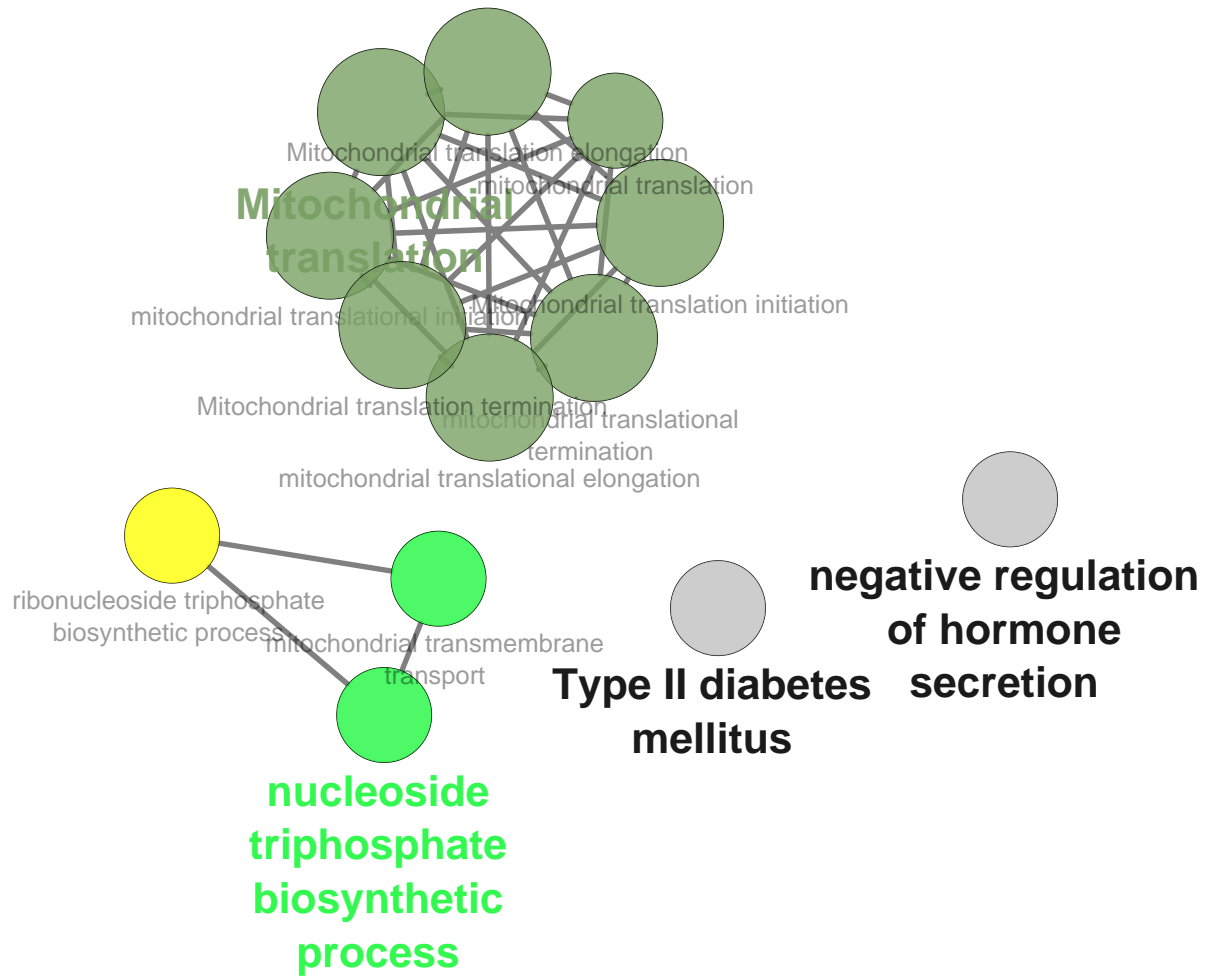

Supplement: Supplementary file 6 — Network analysis figures. All figures were converted to pdf files. (ZIP 47344 kb) [file 12192_2018_954_MOESM6_ESM.zip › Spleen Lowland noon-evening - Cytoscape-ClueGo.pdf]

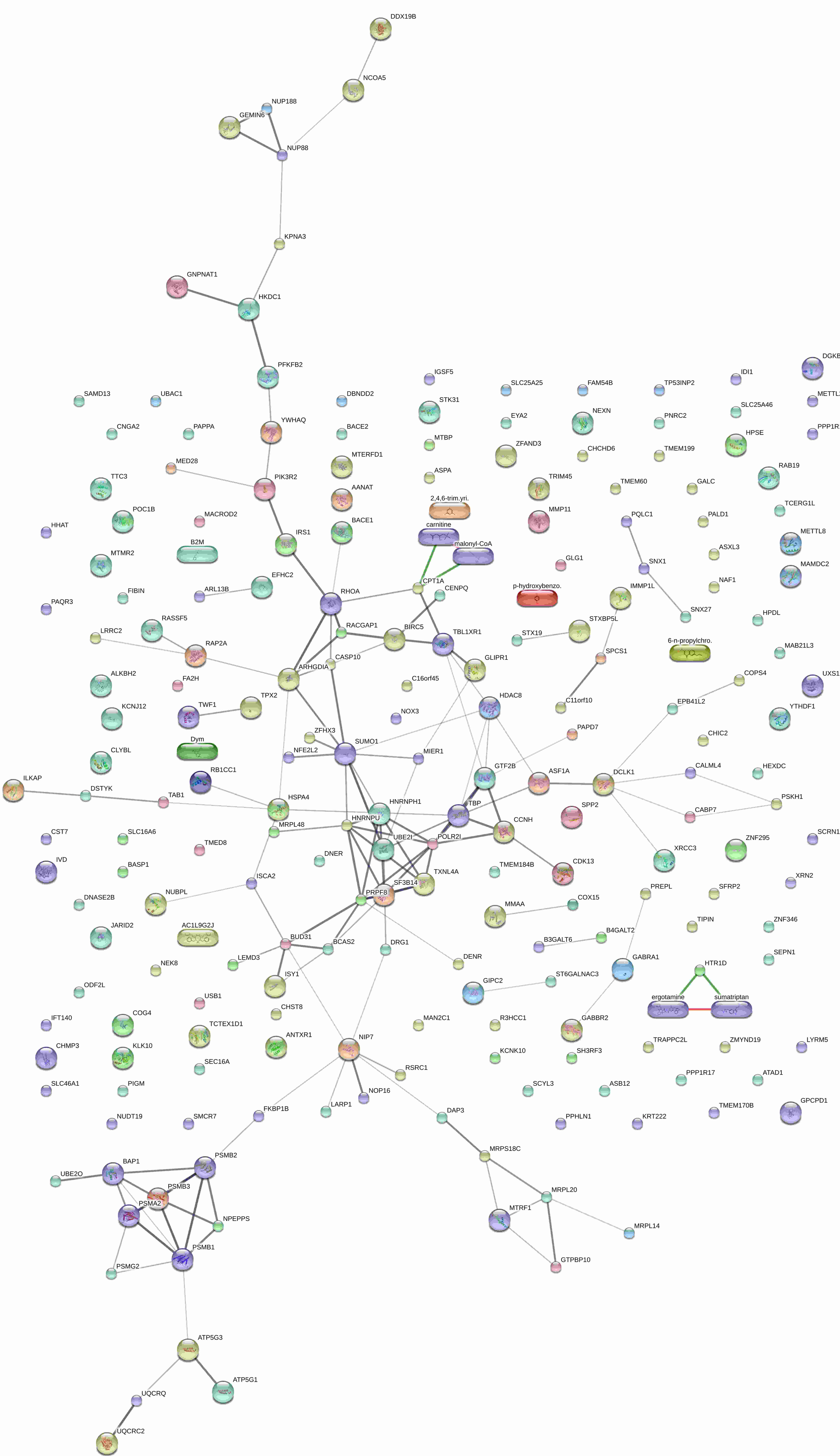

Supplement: Supplementary file 6 — Network analysis figures. All figures were converted to pdf files. (ZIP 47344 kb) [file 12192_2018_954_MOESM6_ESM.zip › Spleen Lowland noon-evening - stitch.pdf]

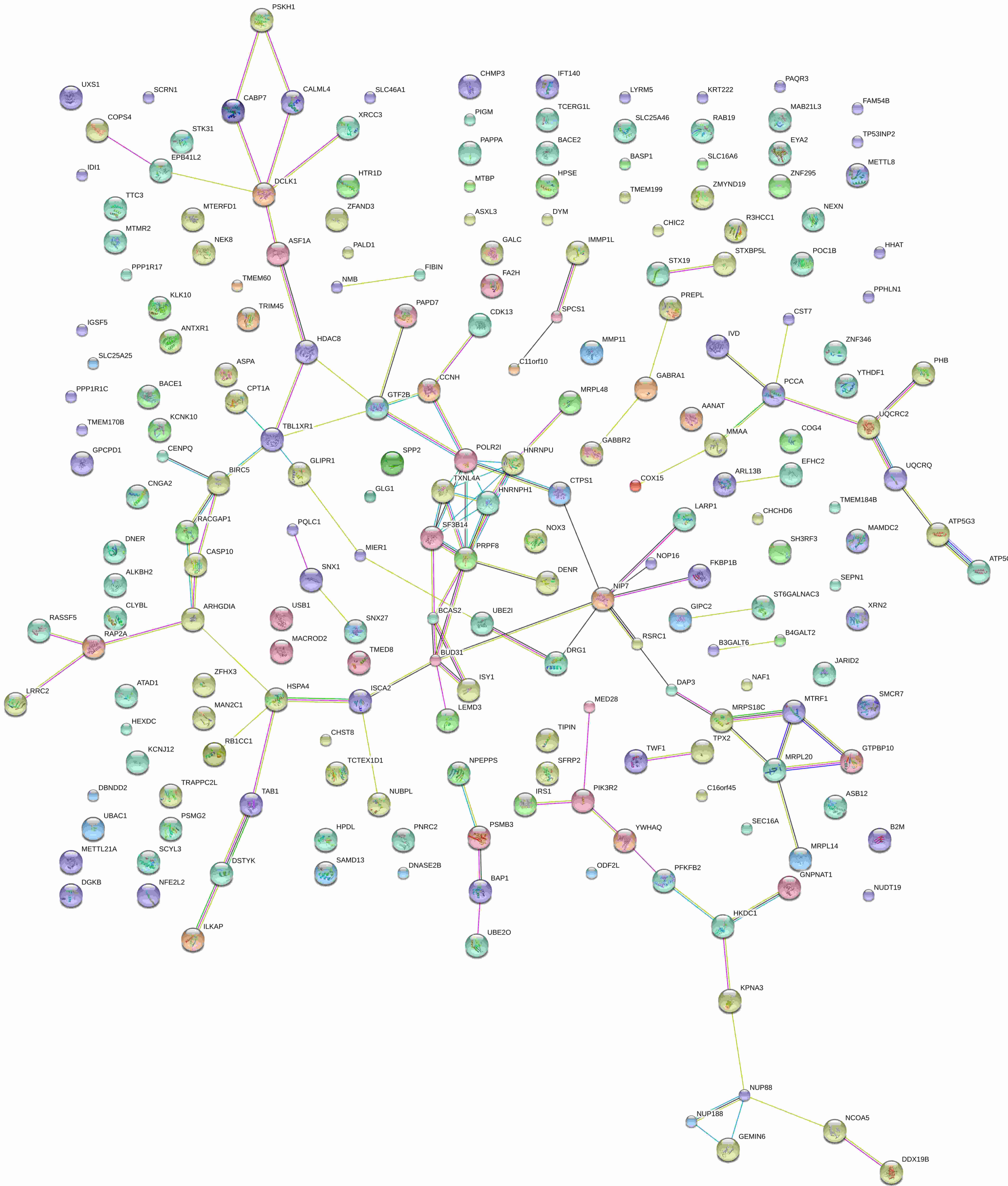

Supplement: Supplementary file 6 — Network analysis figures. All figures were converted to pdf files. (ZIP 47344 kb) [file 12192_2018_954_MOESM6_ESM.zip › Spleen Lowland noon-evening - string.pdf]
