# Supplementary figures and images for: Transcriptomic profiles of muscle, heart, and spleen in reaction to circadian heat stress in Ethiopian highland and lowland male chicken (part 1 of 2)
Source: Cell Stress Chaperones. 2018 Dec 18;24(1):175–94. doi: 10.1007/s12192-018-0954-6 (PMC6363629; doi:10.1007/s12192-018-0954-6)

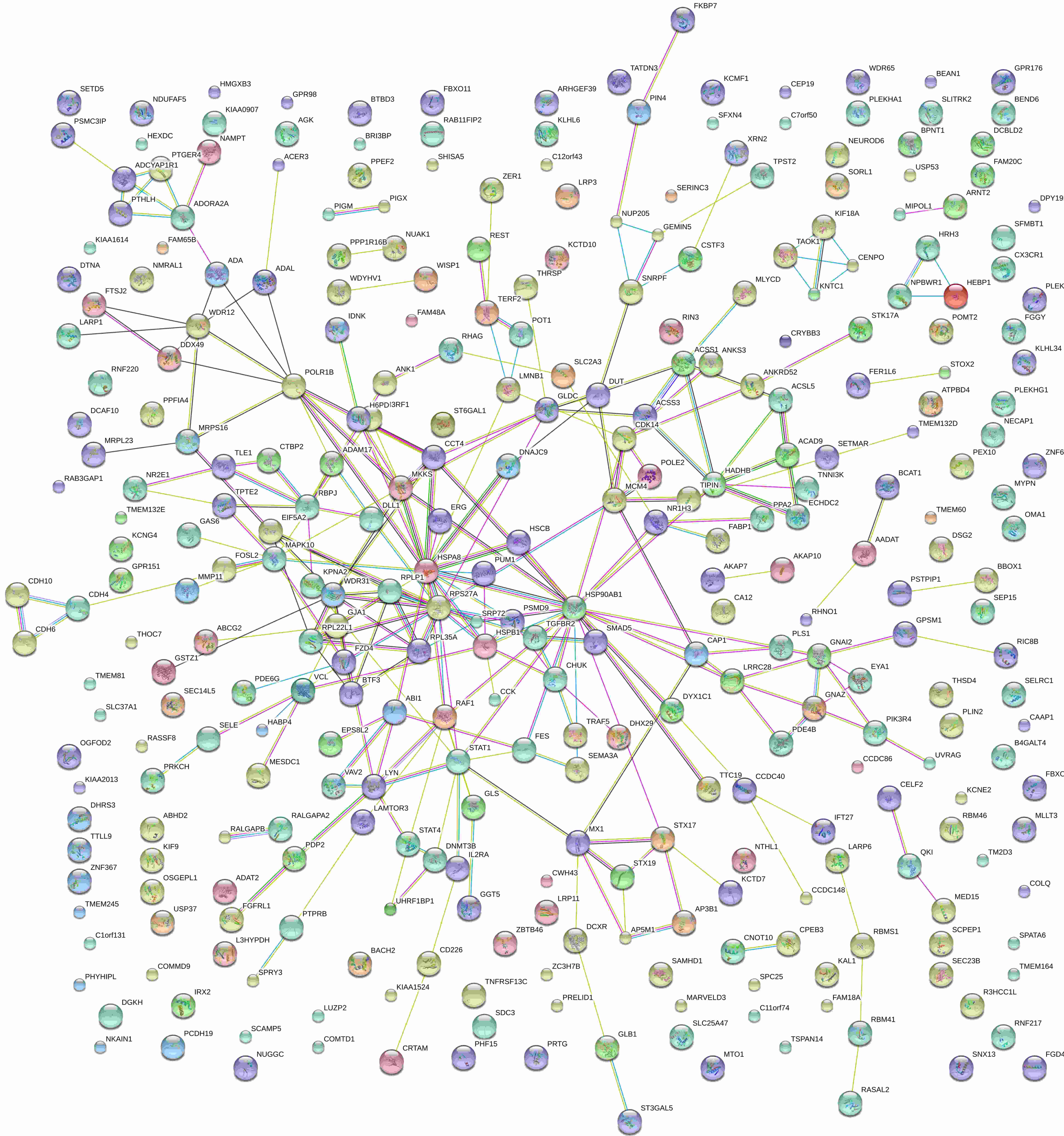

Supplement: Supplementary file 6 — Network analysis figures. All figures were converted to pdf files. (ZIP 47344 kb) [file 12192_2018_954_MOESM6_ESM.zip › Heart highland - lowland noon - string.pdf]

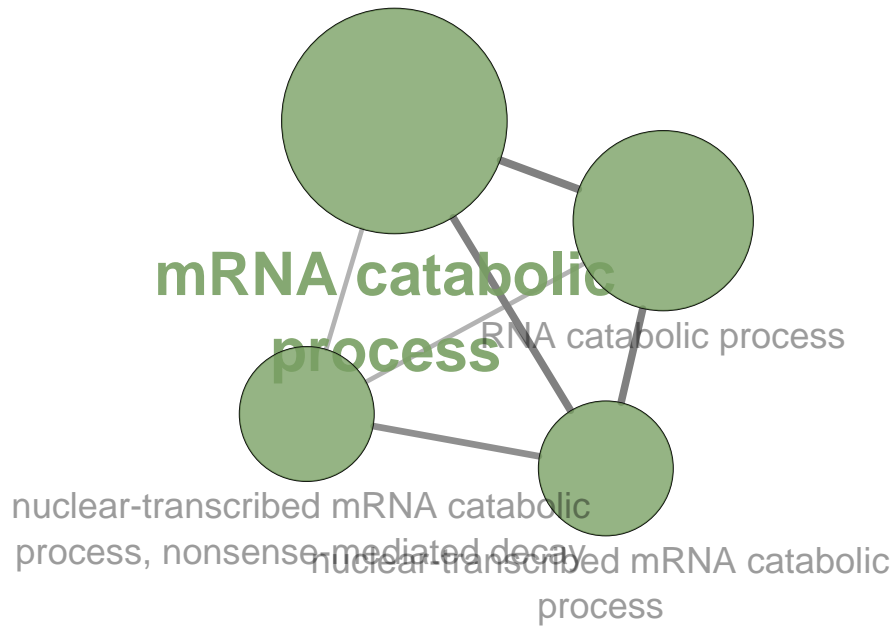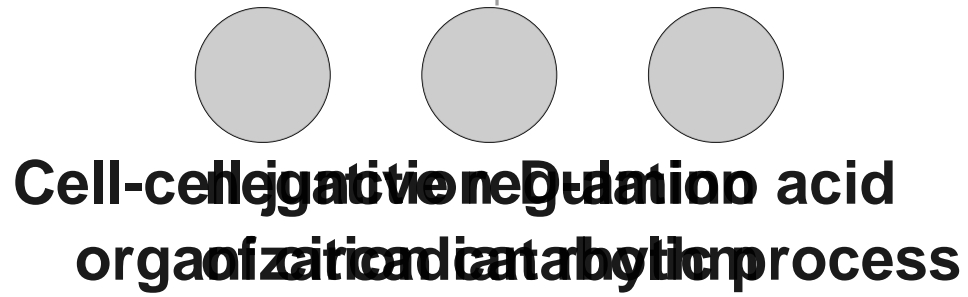

Supplement: Supplementary file 6 — Network analysis figures. All figures were converted to pdf files. (ZIP 47344 kb) [file 12192_2018_954_MOESM6_ESM.zip › Heart highland all - Cytoscape-ClueGo.pdf]

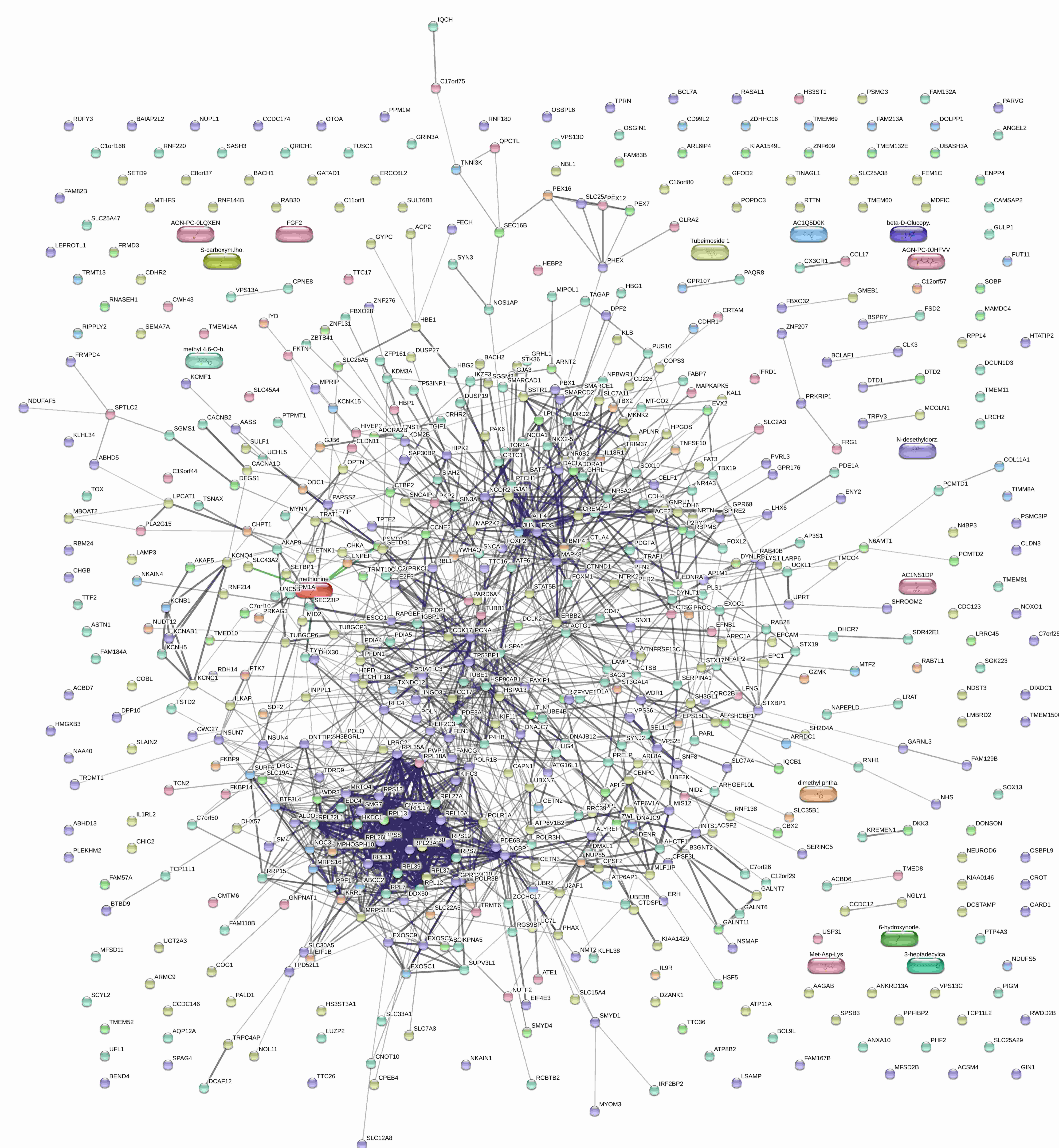

Supplement: Supplementary file 6 — Network analysis figures. All figures were converted to pdf files. (ZIP 47344 kb) [file 12192_2018_954_MOESM6_ESM.zip › Heart highland all - stitch.pdf]

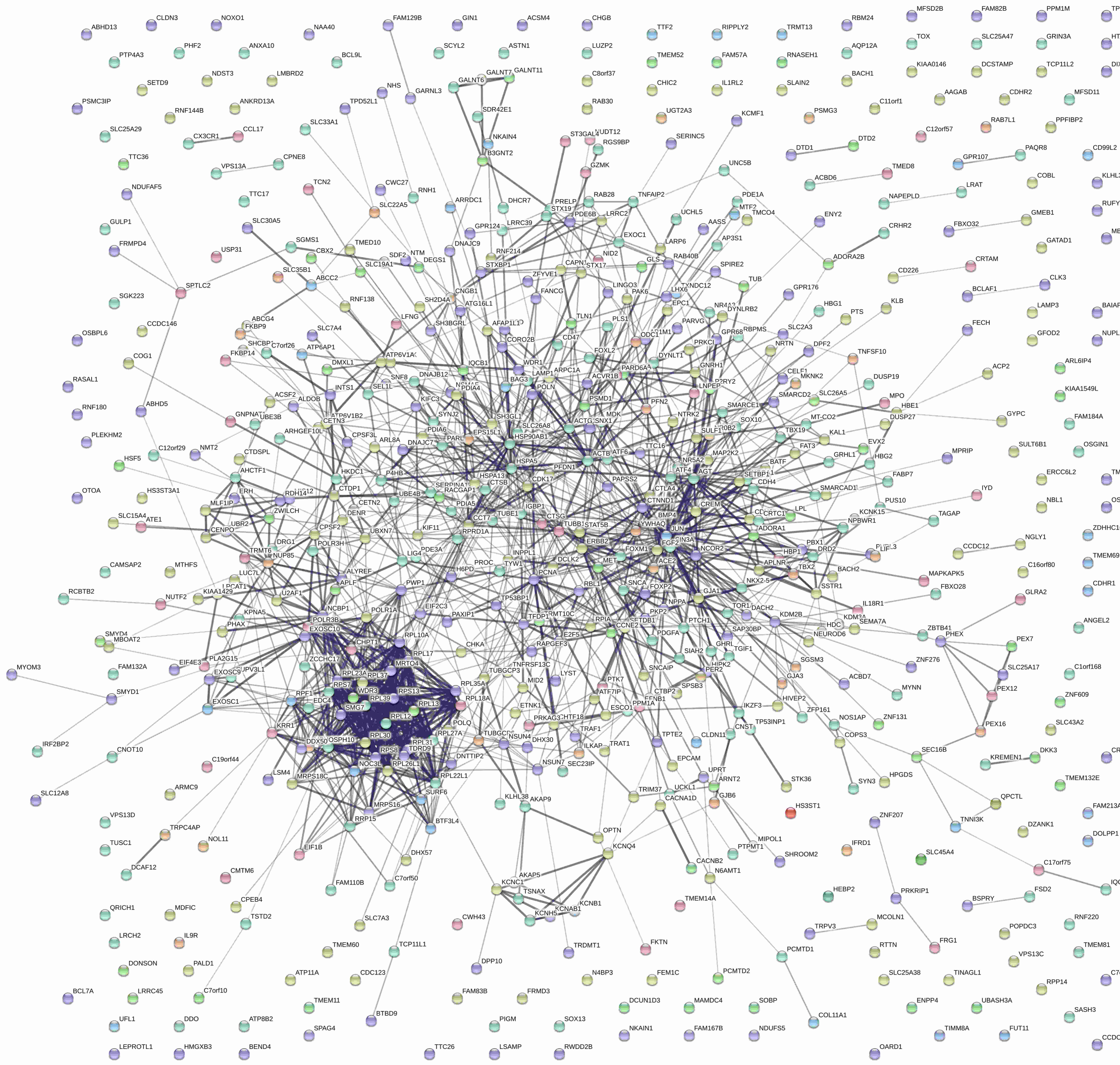

Supplement: Supplementary file 6 — Network analysis figures. All figures were converted to pdf files. (ZIP 47344 kb) [file 12192_2018_954_MOESM6_ESM.zip › Heart highland all - string.pdf]

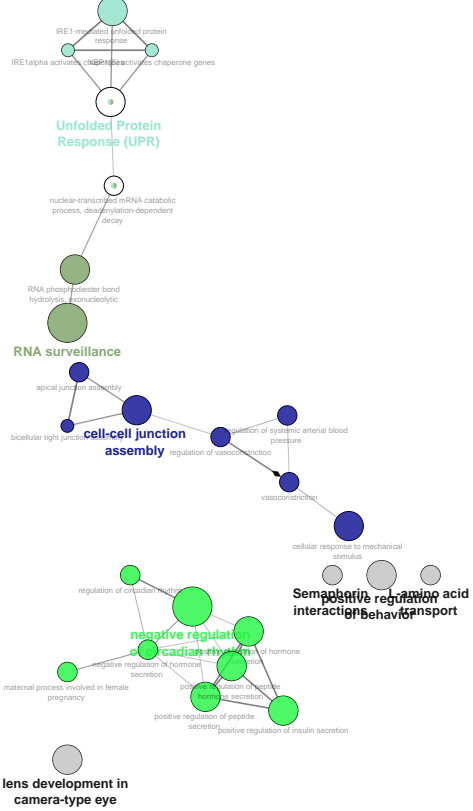

Supplement: Supplementary file 6 — Network analysis figures. All figures were converted to pdf files. (ZIP 47344 kb) [file 12192_2018_954_MOESM6_ESM.zip › Heart highland morning-evening - Cytoscape-ClueGo.pdf]

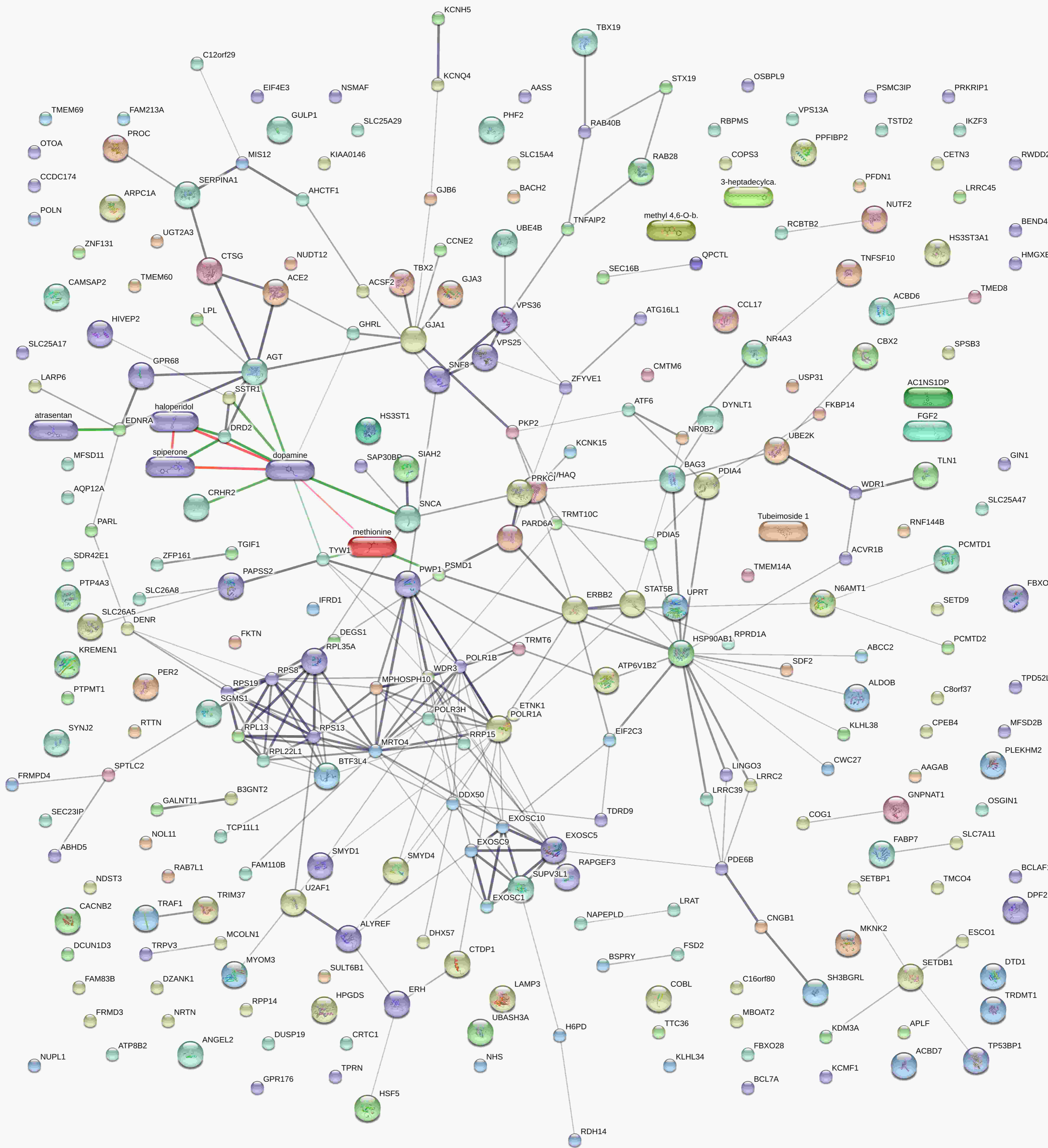

Supplement: Supplementary file 6 — Network analysis figures. All figures were converted to pdf files. (ZIP 47344 kb) [file 12192_2018_954_MOESM6_ESM.zip › Heart highland morning-evening - stitch.pdf]

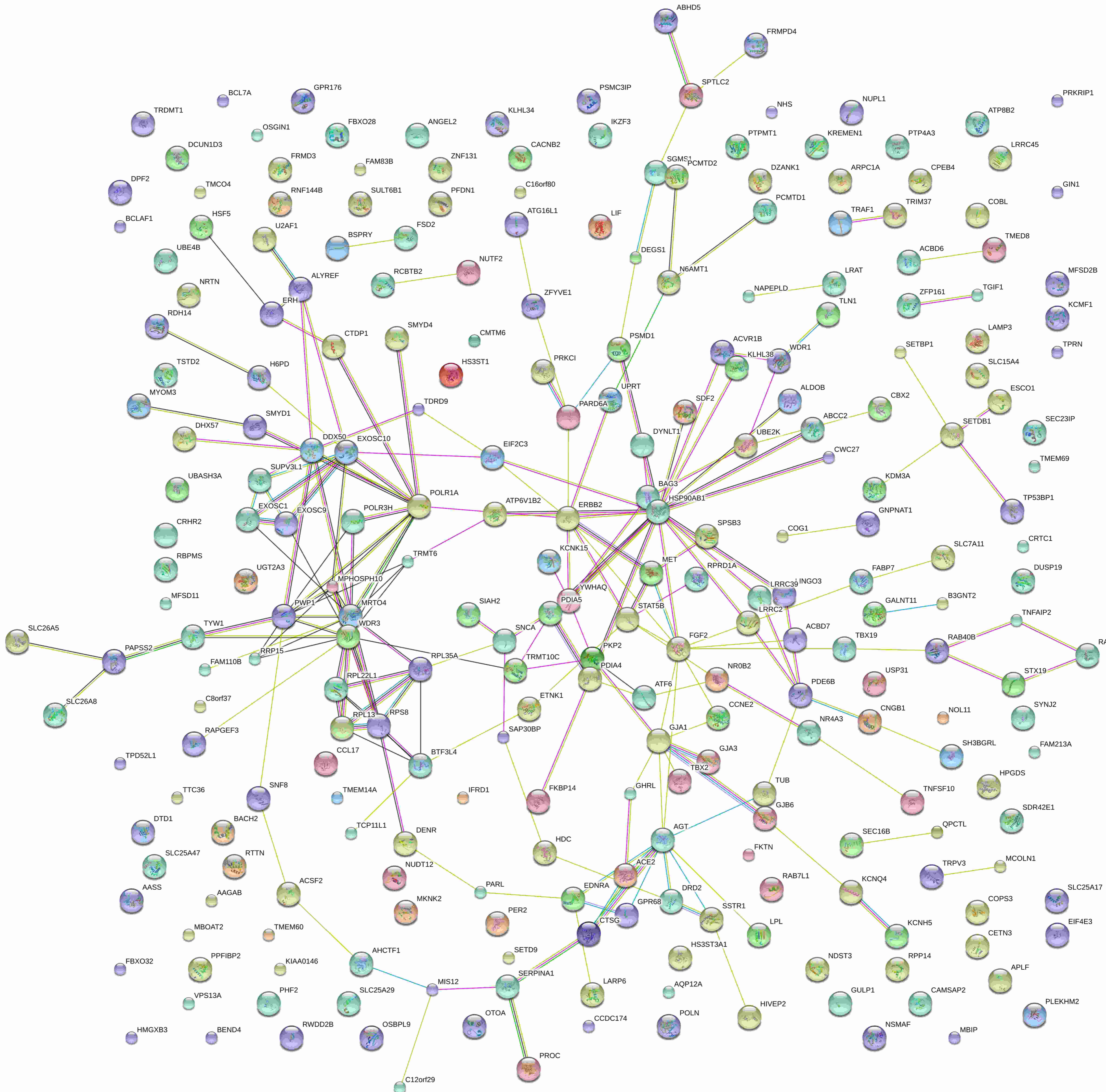

Supplement: Supplementary file 6 — Network analysis figures. All figures were converted to pdf files. (ZIP 47344 kb) [file 12192_2018_954_MOESM6_ESM.zip › Heart highland morning-evening - string.pdf]

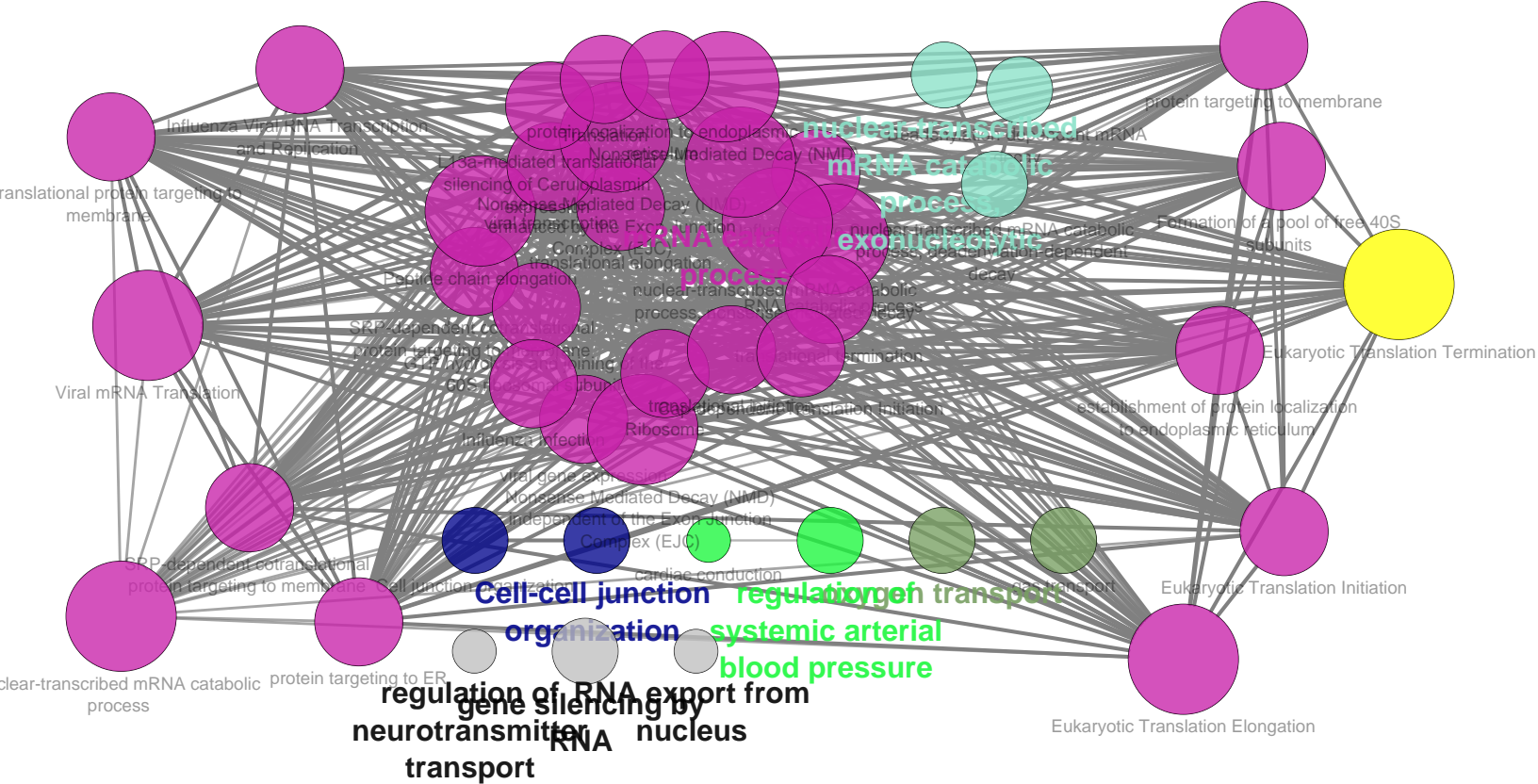

Supplement: Supplementary file 6 — Network analysis figures. All figures were converted to pdf files. (ZIP 47344 kb) [file 12192_2018_954_MOESM6_ESM.zip › Heart highland morning-noon - Cytoscape-ClueGo.pdf]

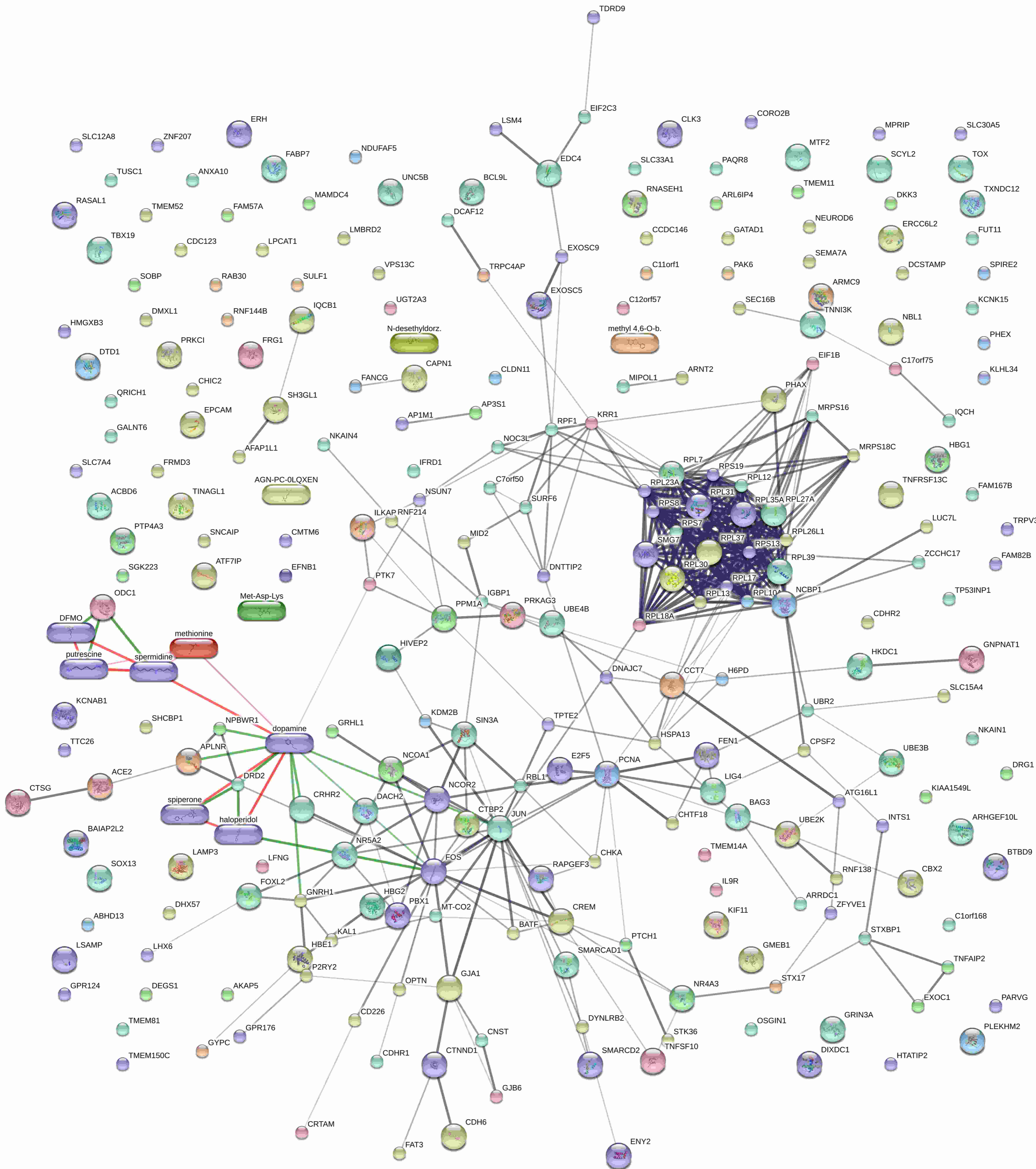

Supplement: Supplementary file 6 — Network analysis figures. All figures were converted to pdf files. (ZIP 47344 kb) [file 12192_2018_954_MOESM6_ESM.zip › Heart highland morning-noon - stitch.pdf]

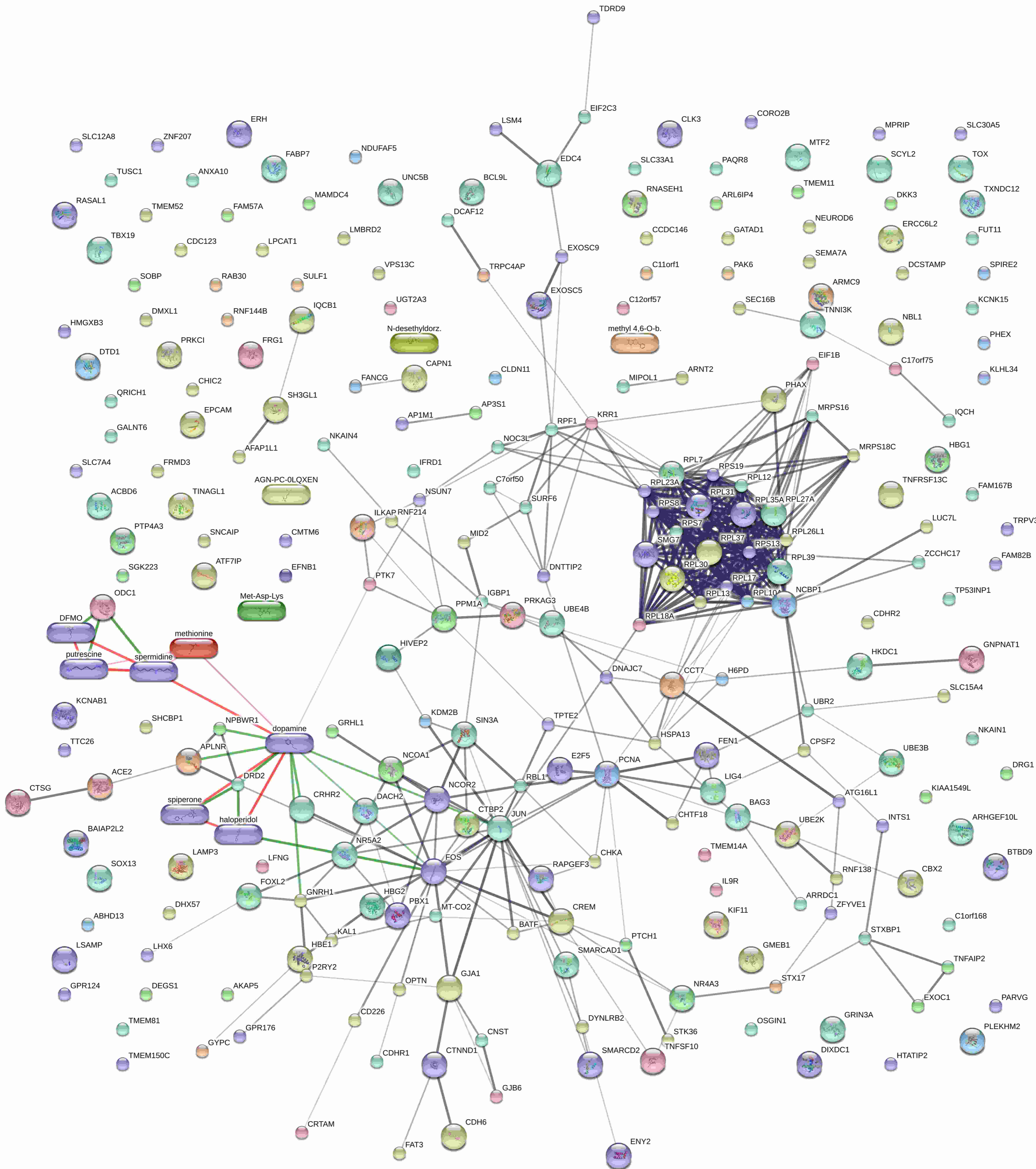

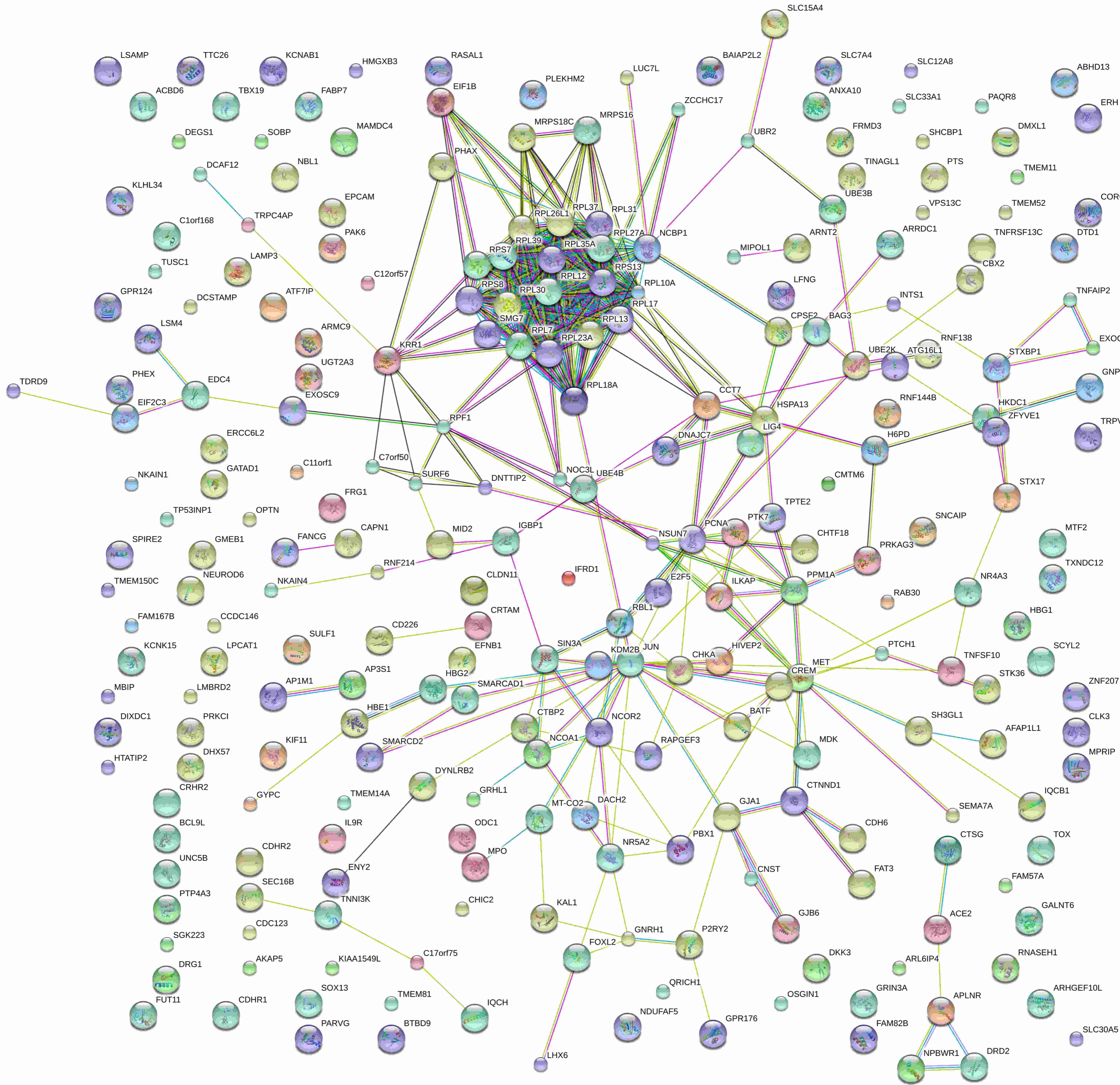

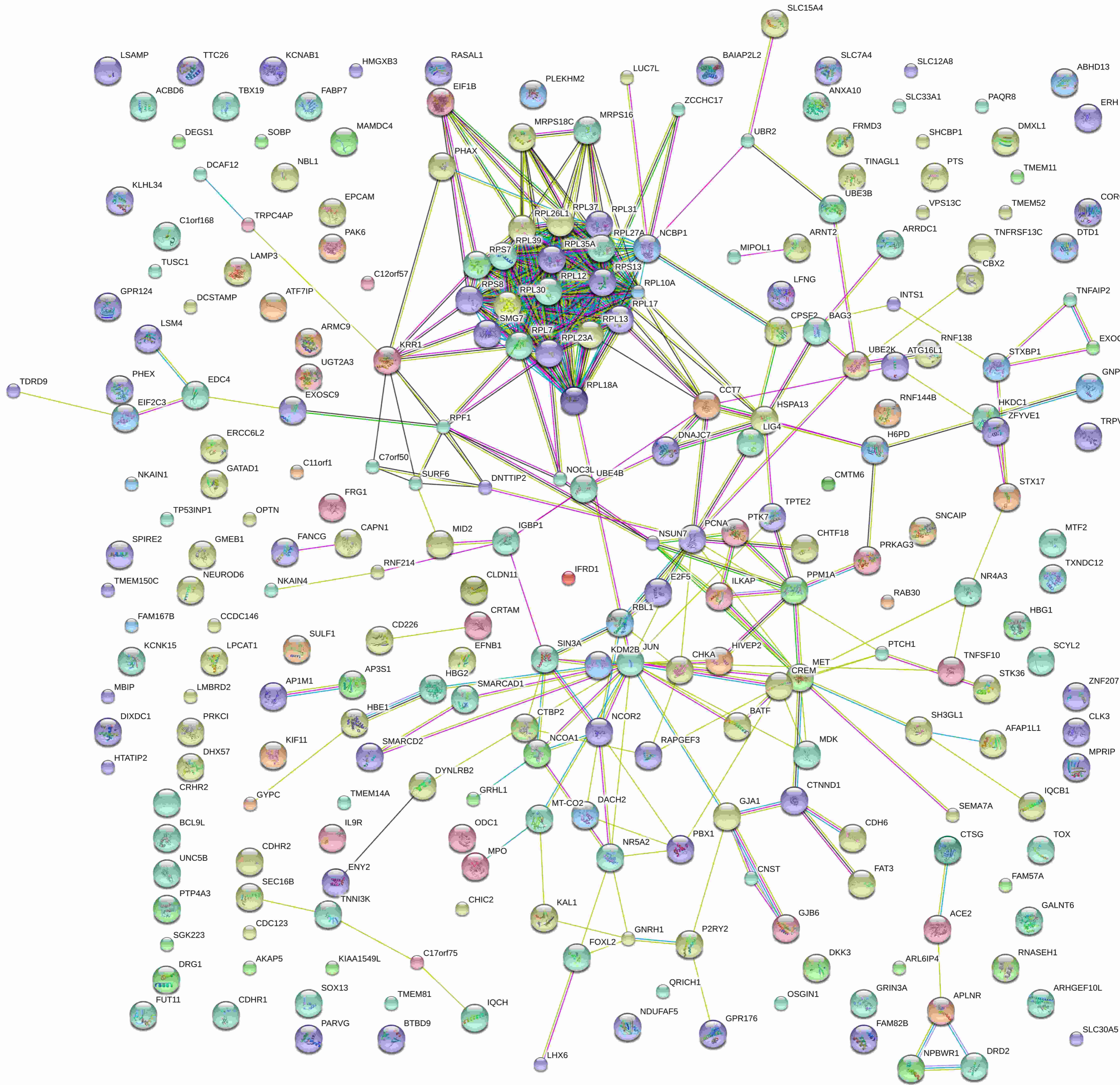

Supplement: Supplementary file 6 — Network analysis figures. All figures were converted to pdf files. (ZIP 47344 kb) [file 12192_2018_954_MOESM6_ESM.zip › Heart highland morning-noon - string.pdf]

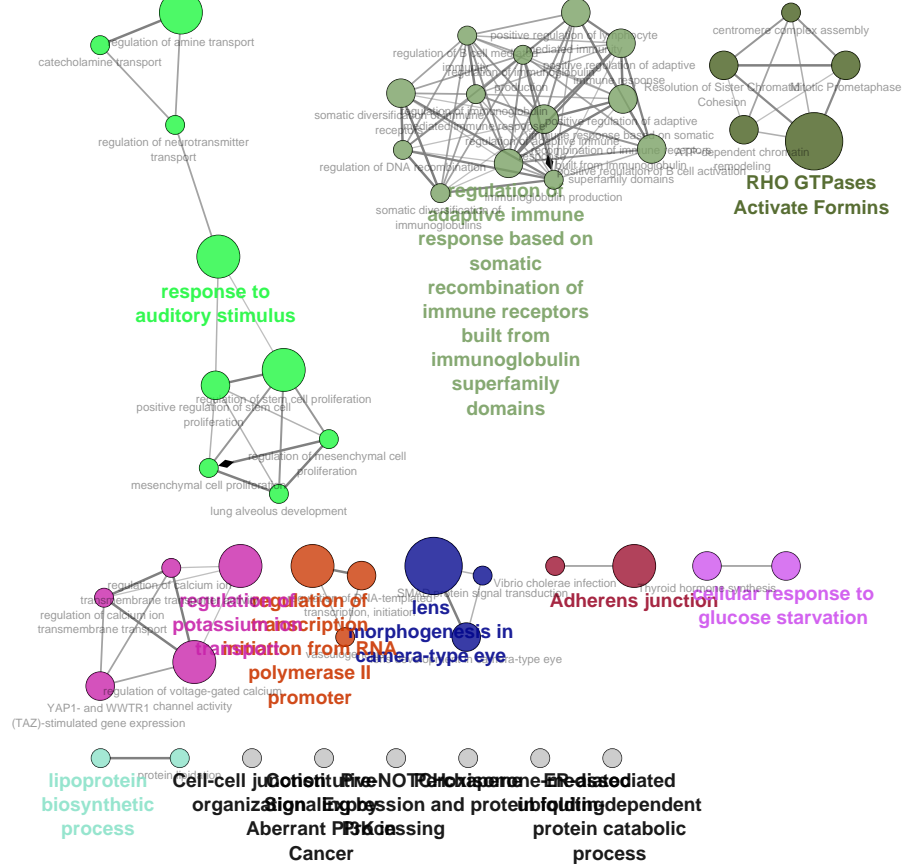

Supplement: Supplementary file 6 — Network analysis figures. All figures were converted to pdf files. (ZIP 47344 kb) [file 12192_2018_954_MOESM6_ESM.zip › Heart highland noon-evening - Cytoscape-ClueGo.pdf]

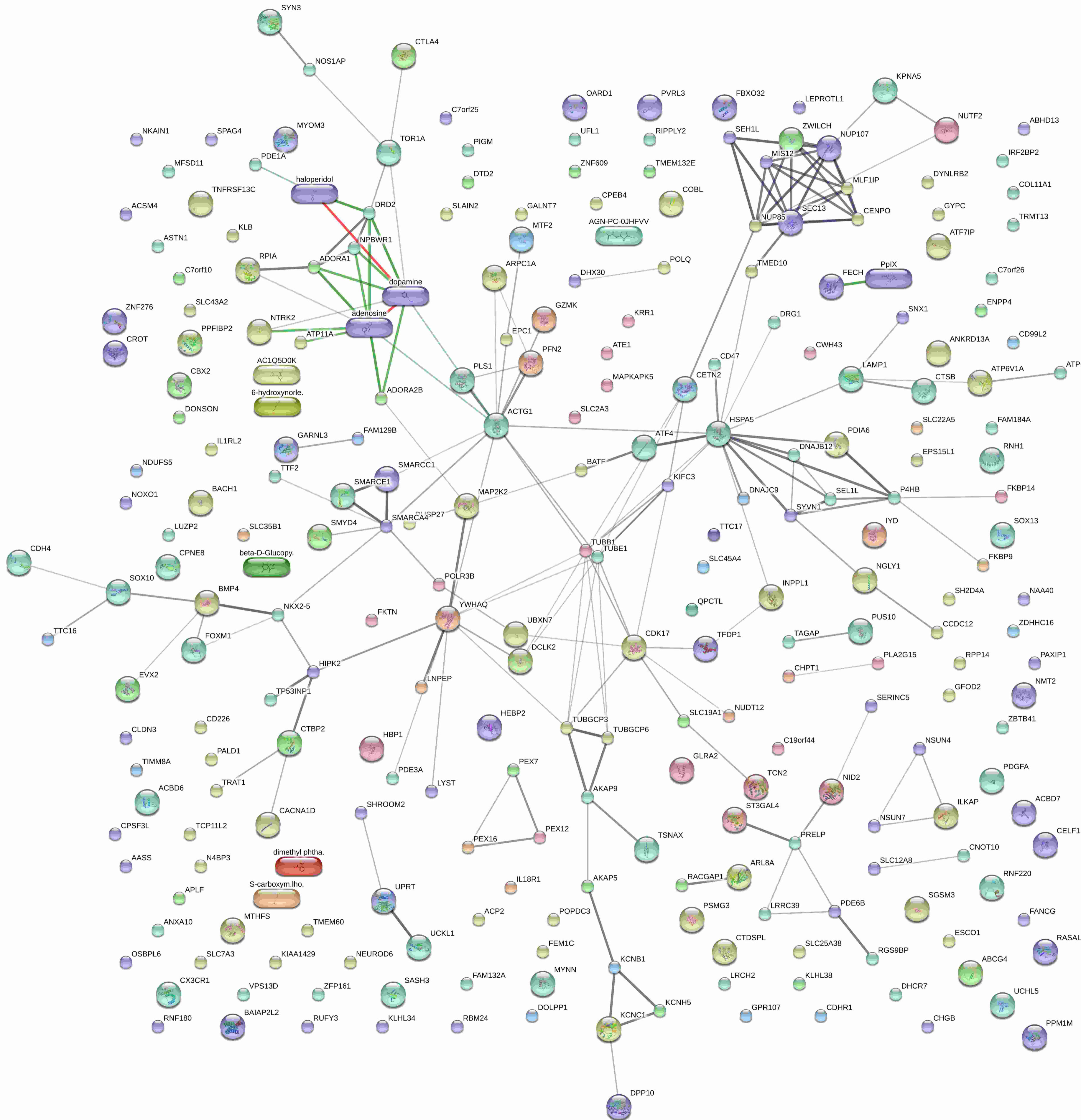

Supplement: Supplementary file 6 — Network analysis figures. All figures were converted to pdf files. (ZIP 47344 kb) [file 12192_2018_954_MOESM6_ESM.zip › Heart highland noon-evening - stitch.pdf]

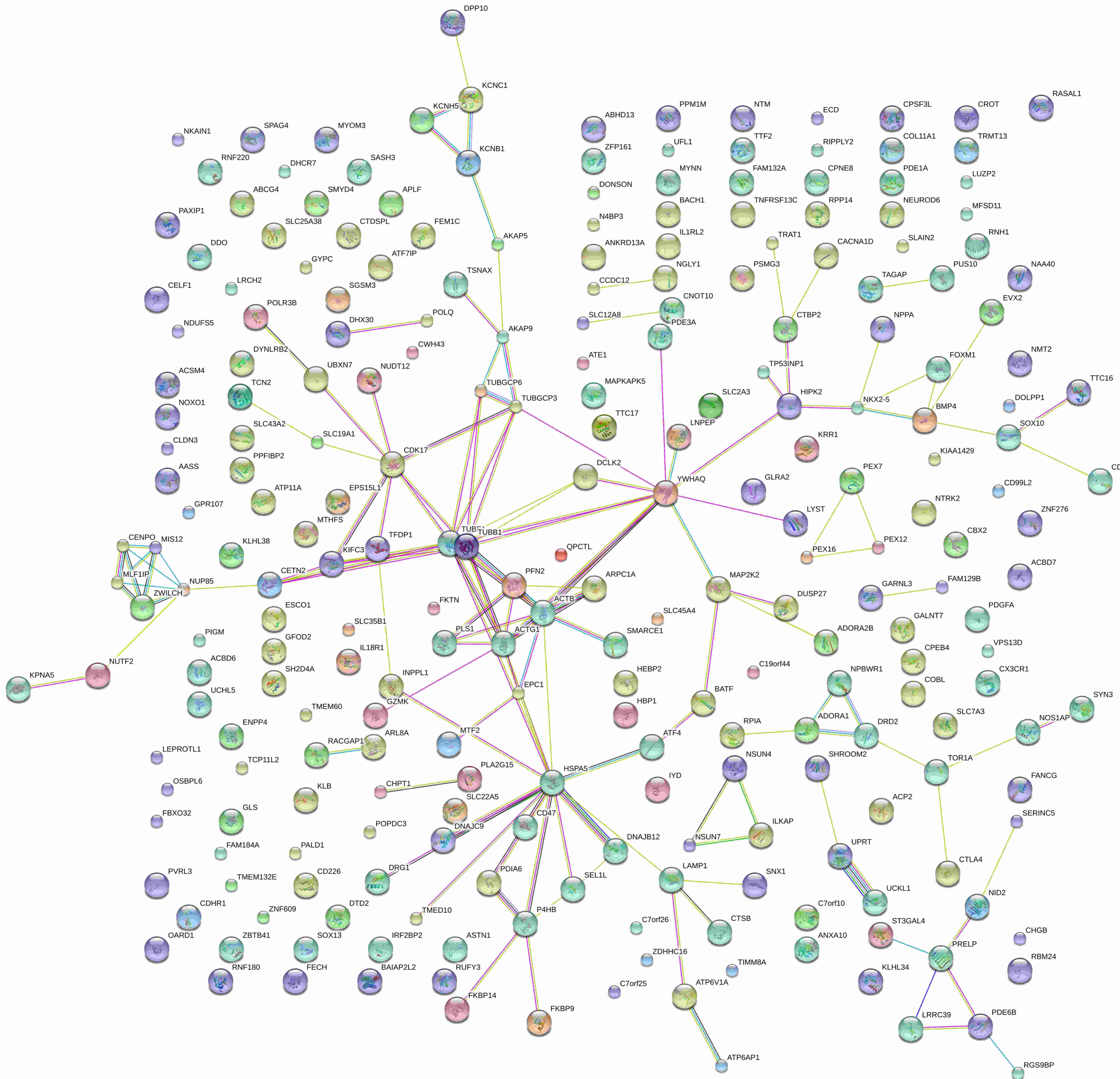

Supplement: Supplementary file 6 — Network analysis figures. All figures were converted to pdf files. (ZIP 47344 kb) [file 12192_2018_954_MOESM6_ESM.zip › Heart highland noon-evening - string.pdf]

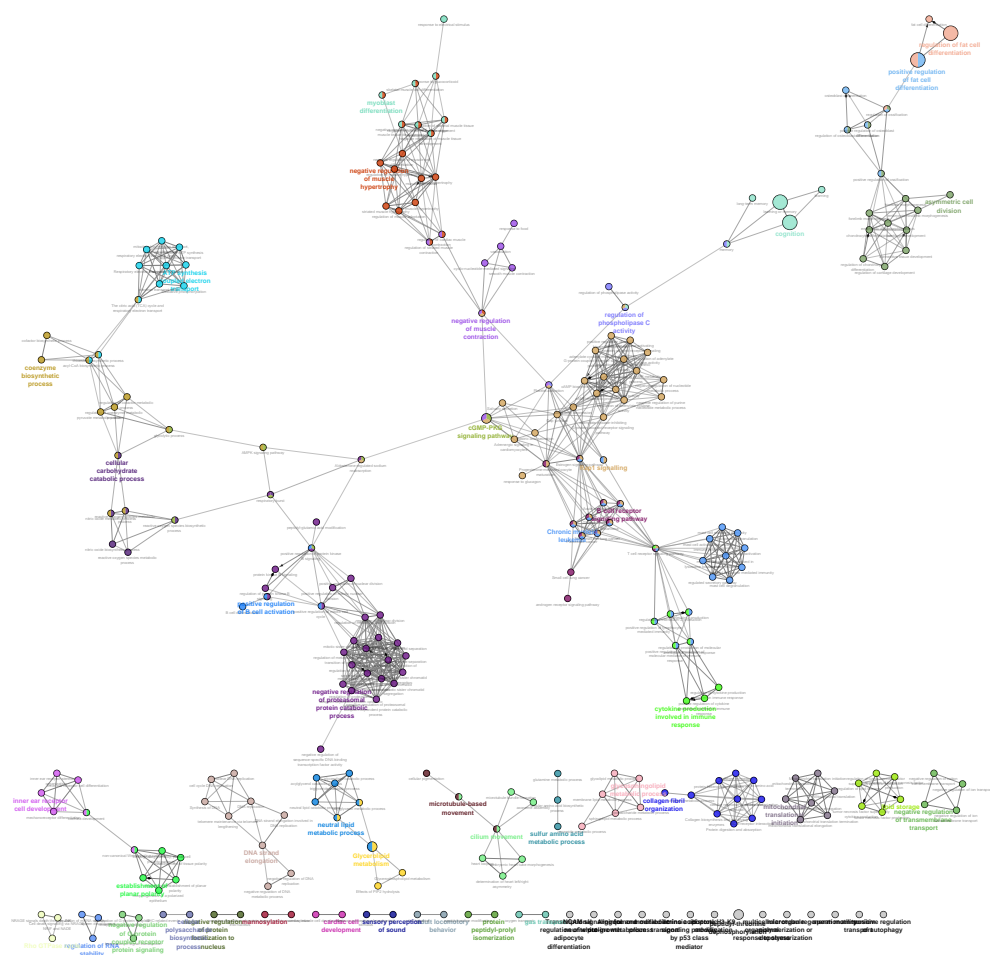

Supplement: Supplementary file 6 — Network analysis figures. All figures were converted to pdf files. (ZIP 47344 kb) [file 12192_2018_954_MOESM6_ESM.zip › Heart highland-lowland all - Cytoscape-ClueGo.pdf]

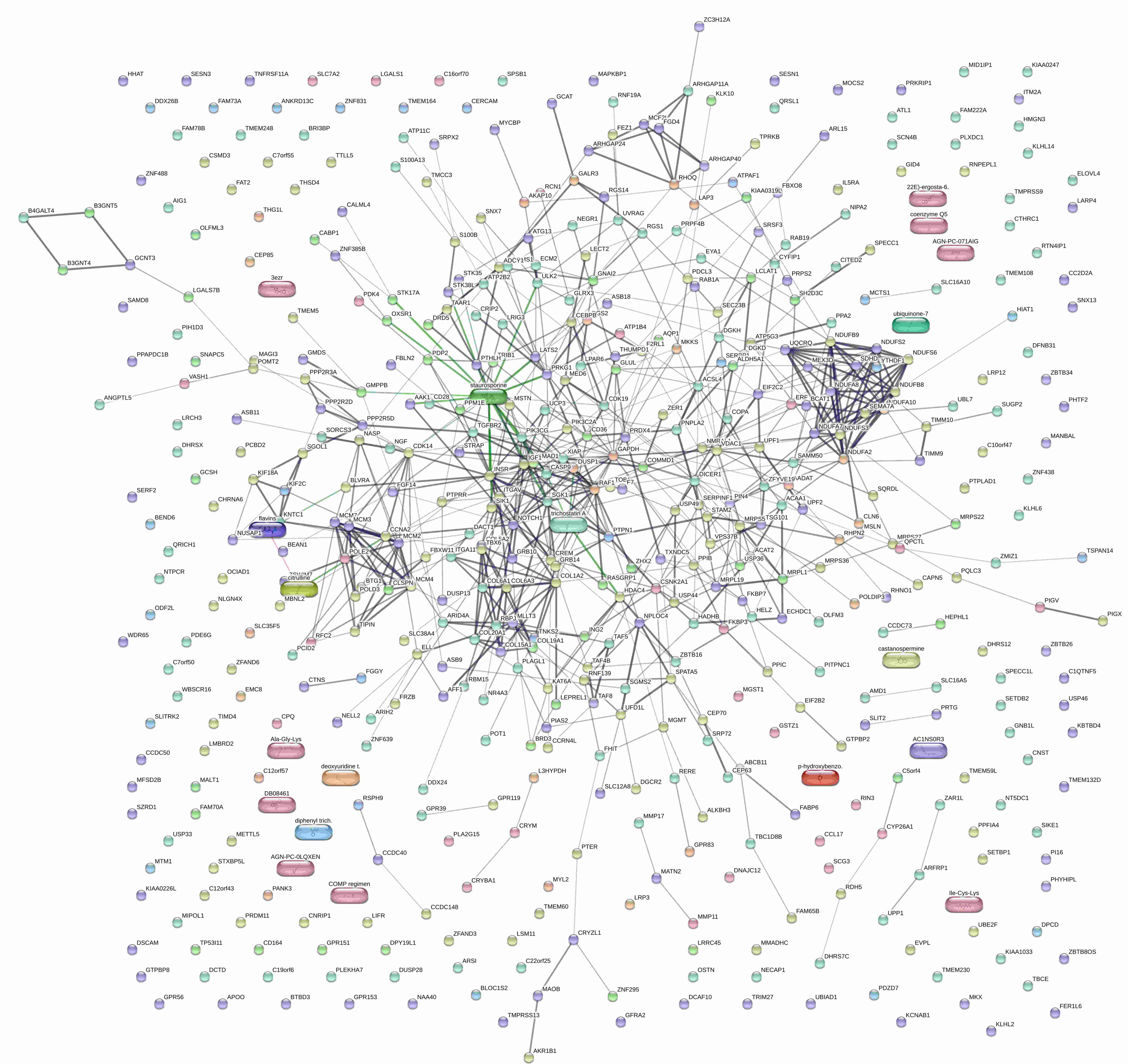

Supplement: Supplementary file 6 — Network analysis figures. All figures were converted to pdf files. (ZIP 47344 kb) [file 12192_2018_954_MOESM6_ESM.zip › Heart highland-lowland all - stitch.pdf]

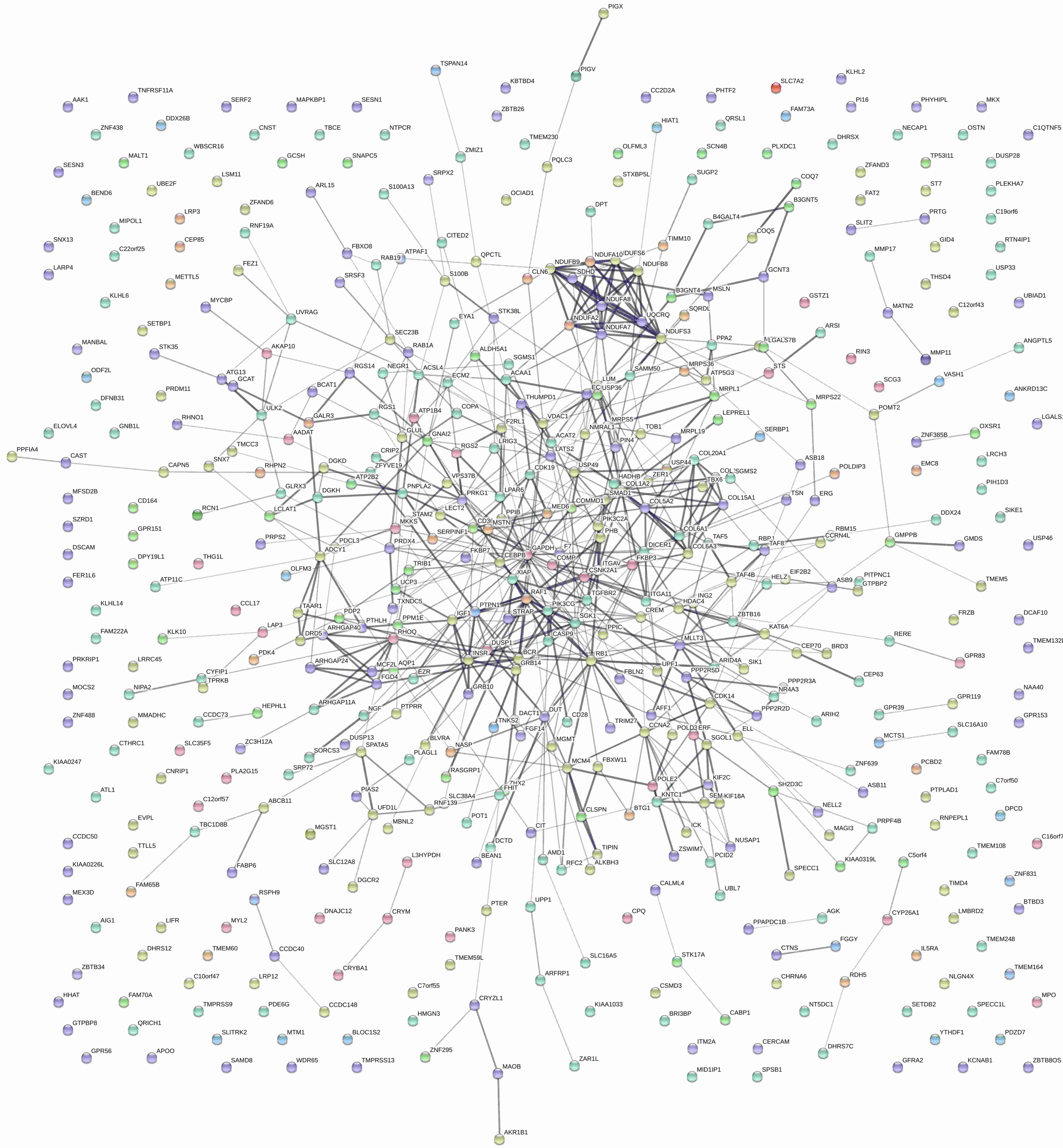

Supplement: Supplementary file 6 — Network analysis figures. All figures were converted to pdf files. (ZIP 47344 kb) [file 12192_2018_954_MOESM6_ESM.zip › Heart highland-lowland all - string.pdf]

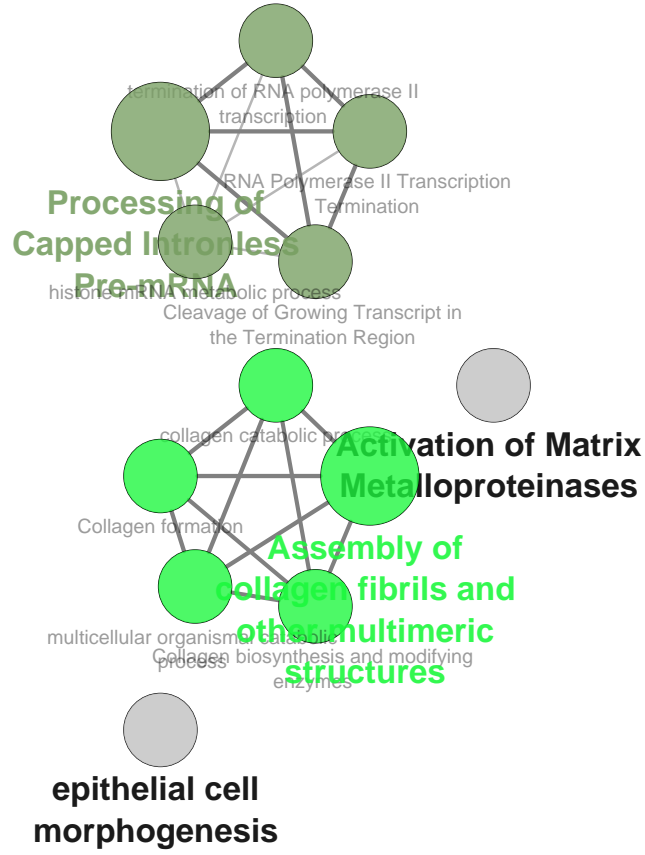

Supplement: Supplementary file 6 — Network analysis figures. All figures were converted to pdf files. (ZIP 47344 kb) [file 12192_2018_954_MOESM6_ESM.zip › Heart highland-lowland evening - Cytoscape-ClueGo.pdf]

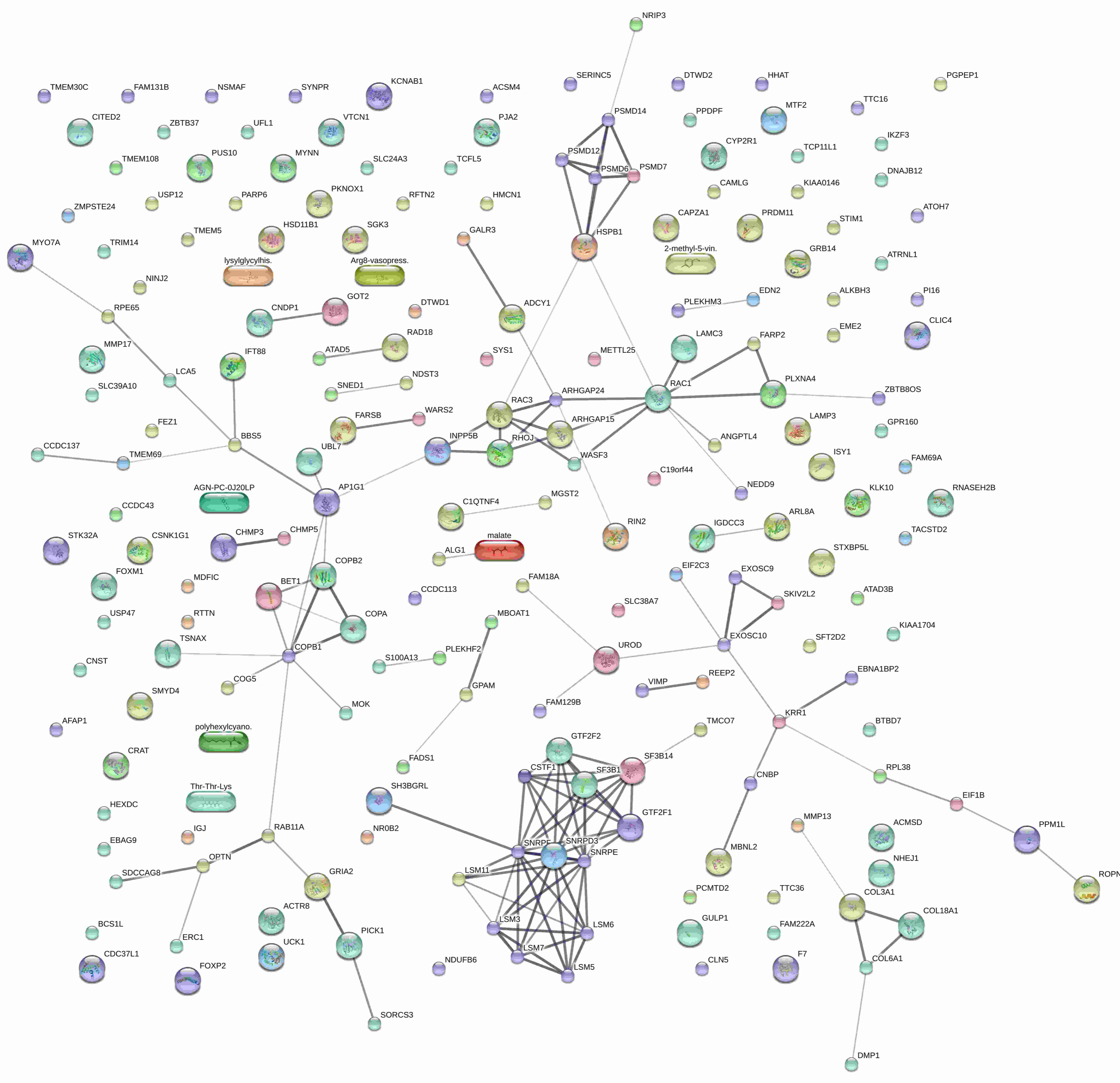

Supplement: Supplementary file 6 — Network analysis figures. All figures were converted to pdf files. (ZIP 47344 kb) [file 12192_2018_954_MOESM6_ESM.zip › Heart highland-lowland evening - stitch.pdf]

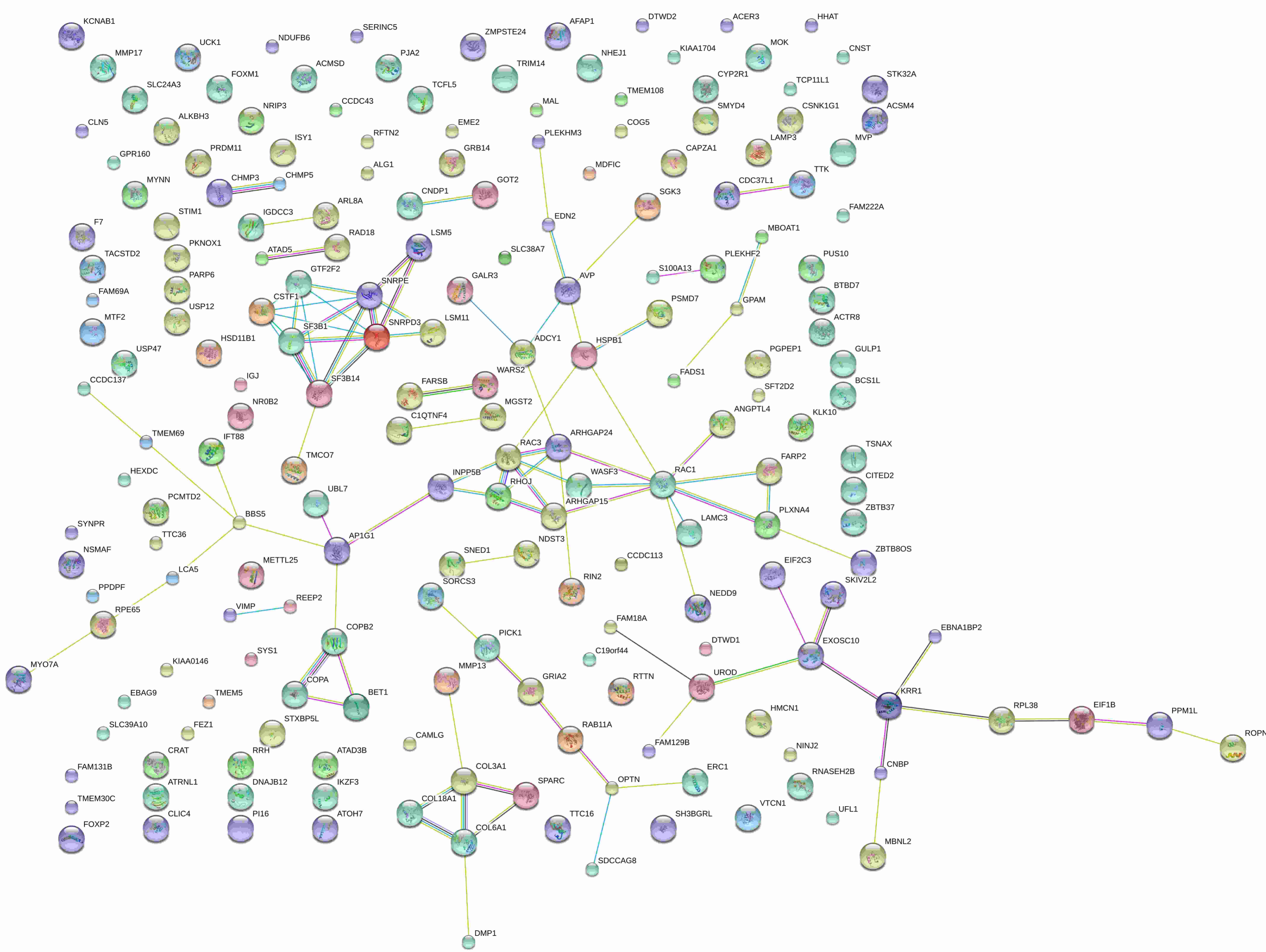

Supplement: Supplementary file 6 — Network analysis figures. All figures were converted to pdf files. (ZIP 47344 kb) [file 12192_2018_954_MOESM6_ESM.zip › Heart highland-lowland evening - string.pdf]

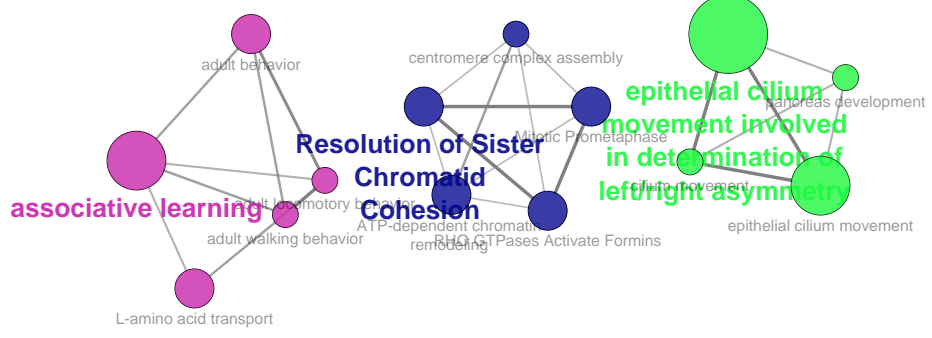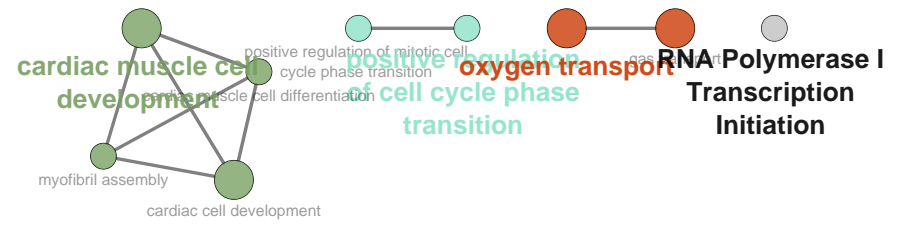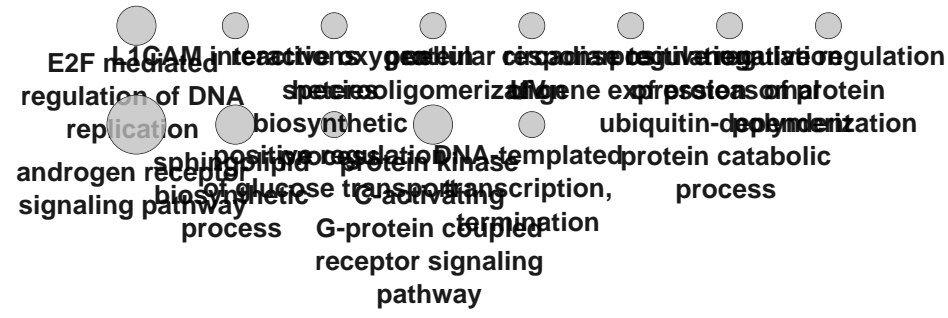

Supplement: Supplementary file 6 — Network analysis figures. All figures were converted to pdf files. (ZIP 47344 kb) [file 12192_2018_954_MOESM6_ESM.zip › Heart highland-lowland morning - Cytoscape-ClueGo.pdf]

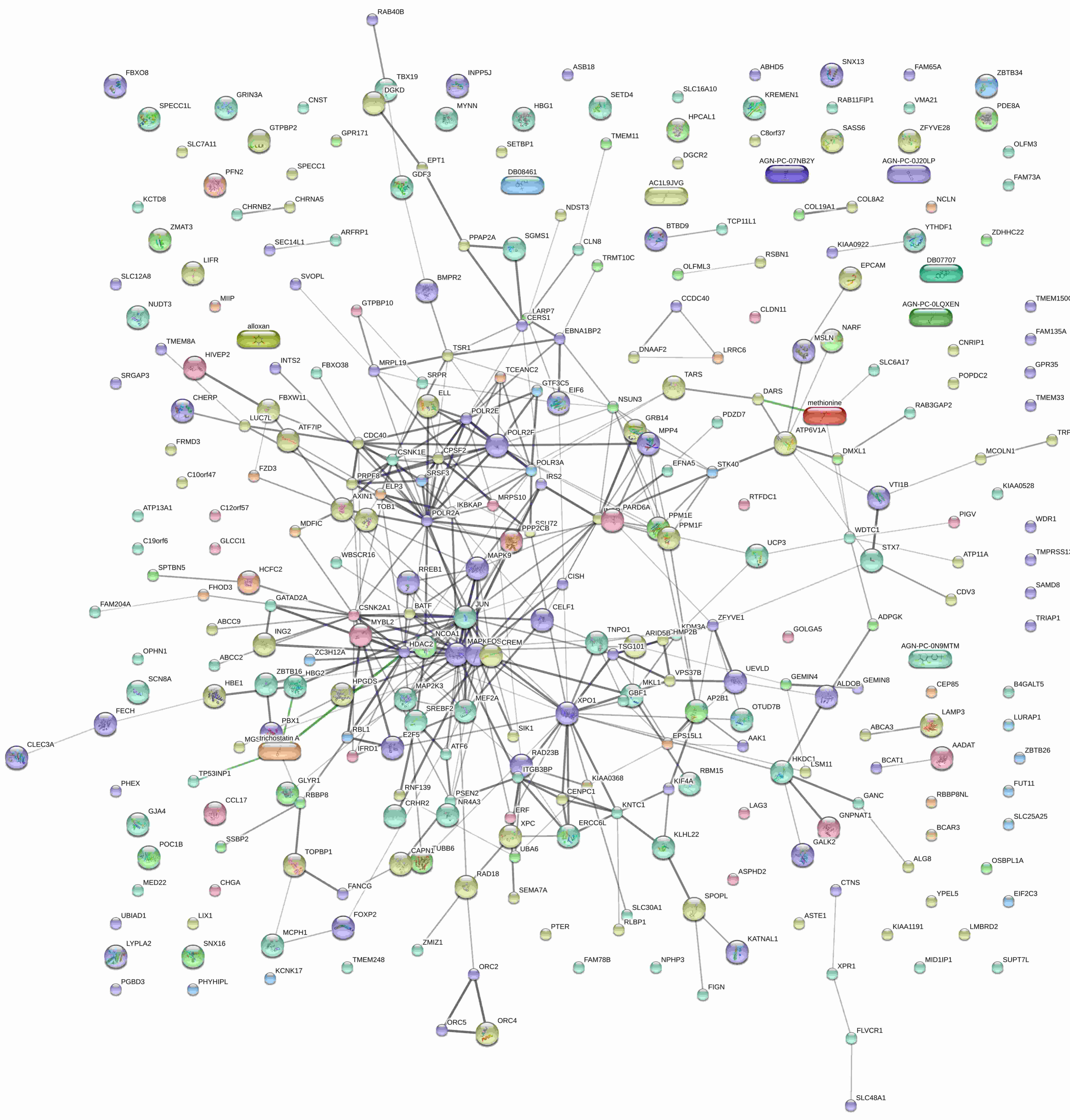

Supplement: Supplementary file 6 — Network analysis figures. All figures were converted to pdf files. (ZIP 47344 kb) [file 12192_2018_954_MOESM6_ESM.zip › Heart highland-lowland morning - stitch.pdf]

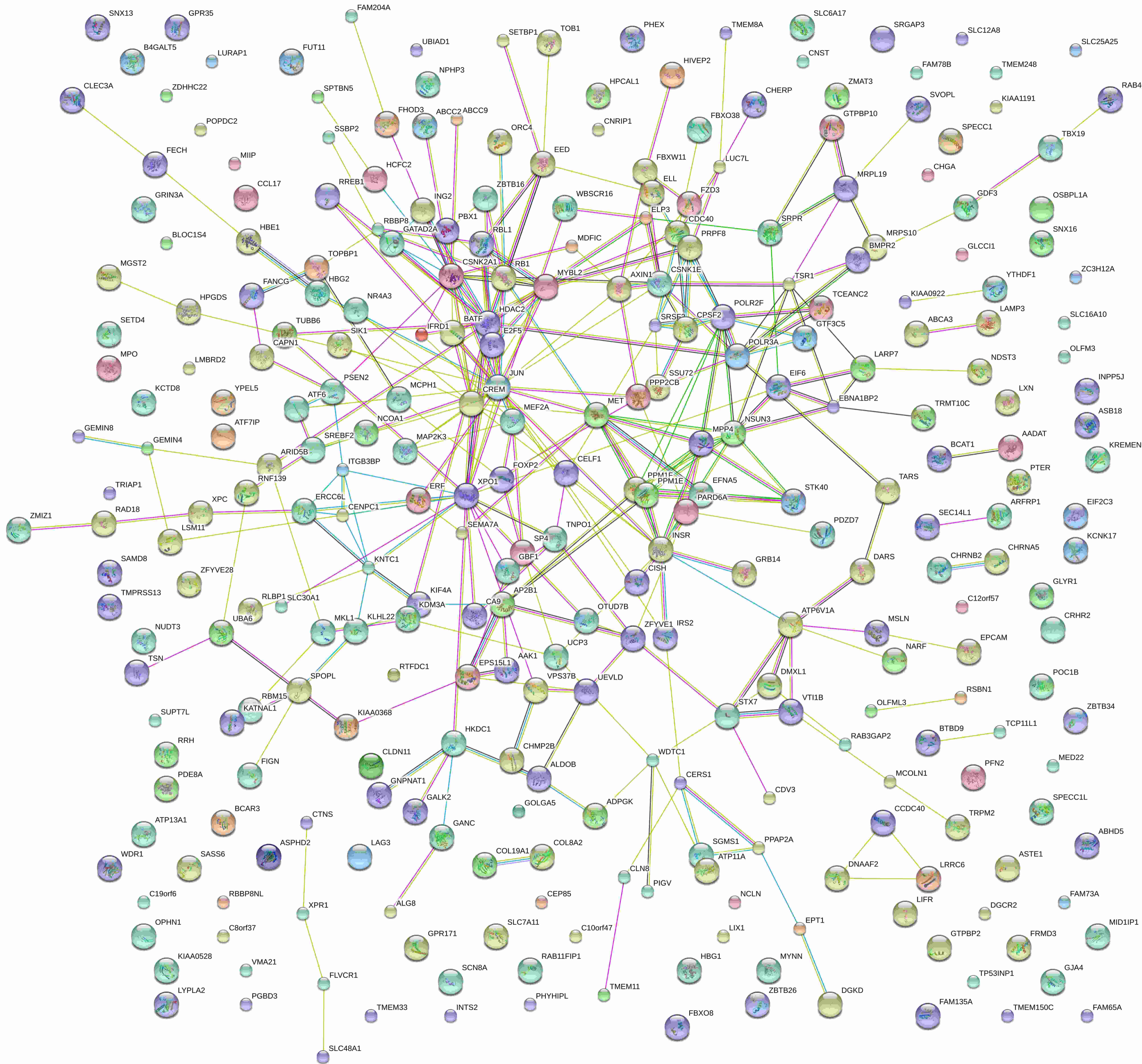

Supplement: Supplementary file 6 — Network analysis figures. All figures were converted to pdf files. (ZIP 47344 kb) [file 12192_2018_954_MOESM6_ESM.zip › Heart highland-lowland morning - string.pdf]

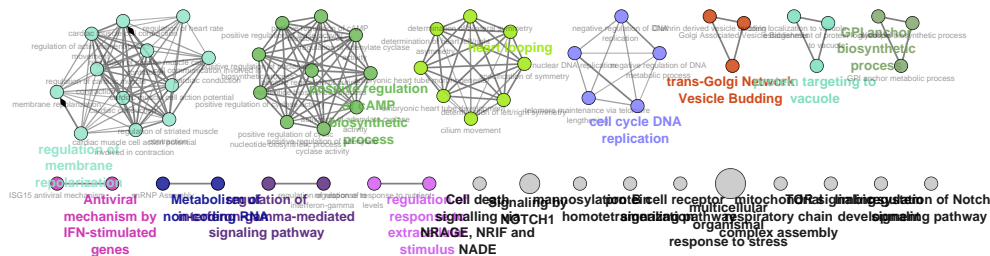

Supplement: Supplementary file 6 — Network analysis figures. All figures were converted to pdf files. (ZIP 47344 kb) [file 12192_2018_954_MOESM6_ESM.zip › Heart highland-lowland noon - Cytoscape-ClueGo.pdf]

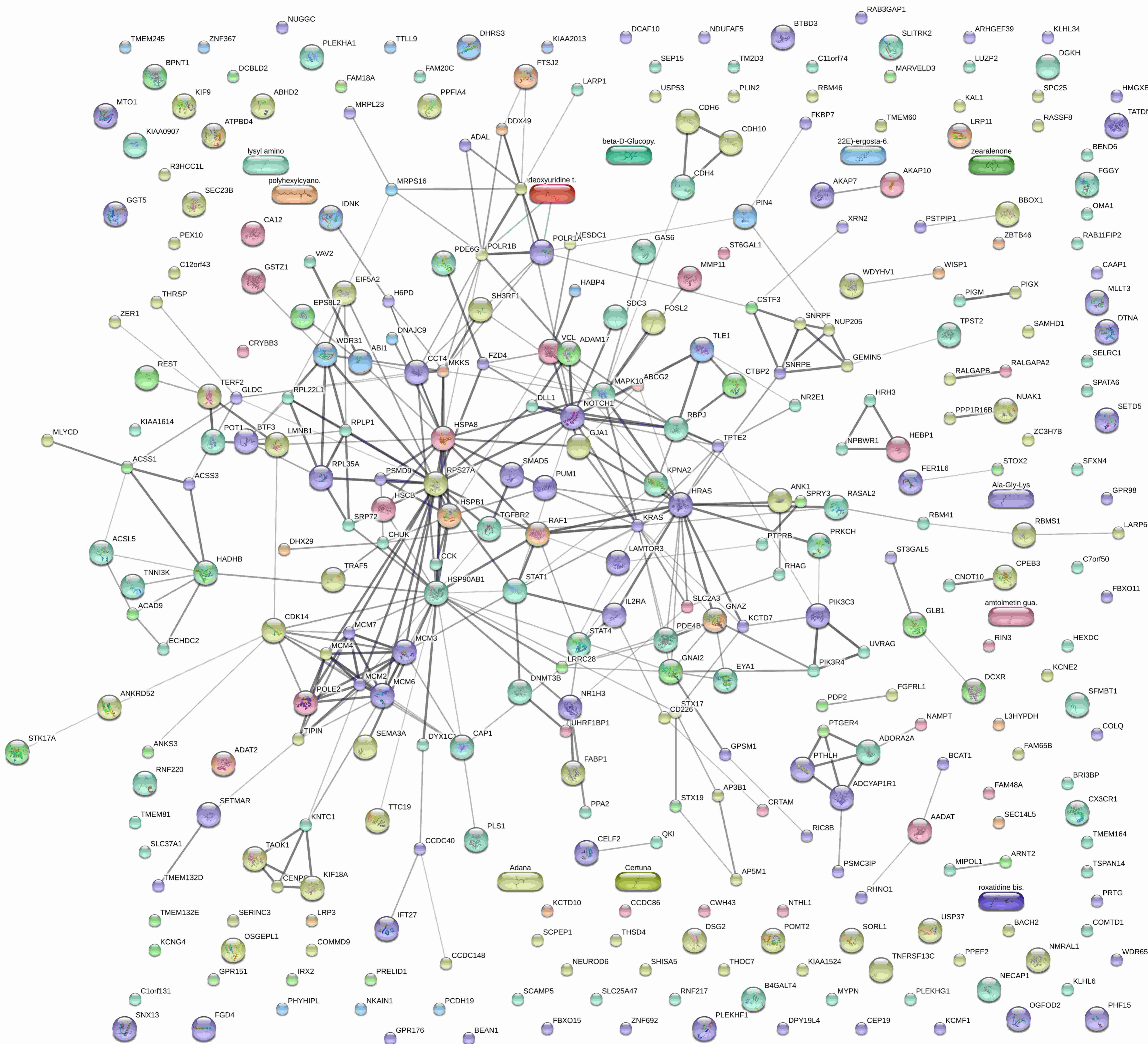

Supplement: Supplementary file 6 — Network analysis figures. All figures were converted to pdf files. (ZIP 47344 kb) [file 12192_2018_954_MOESM6_ESM.zip › Heart highland-lowland noon - stitch.pdf]

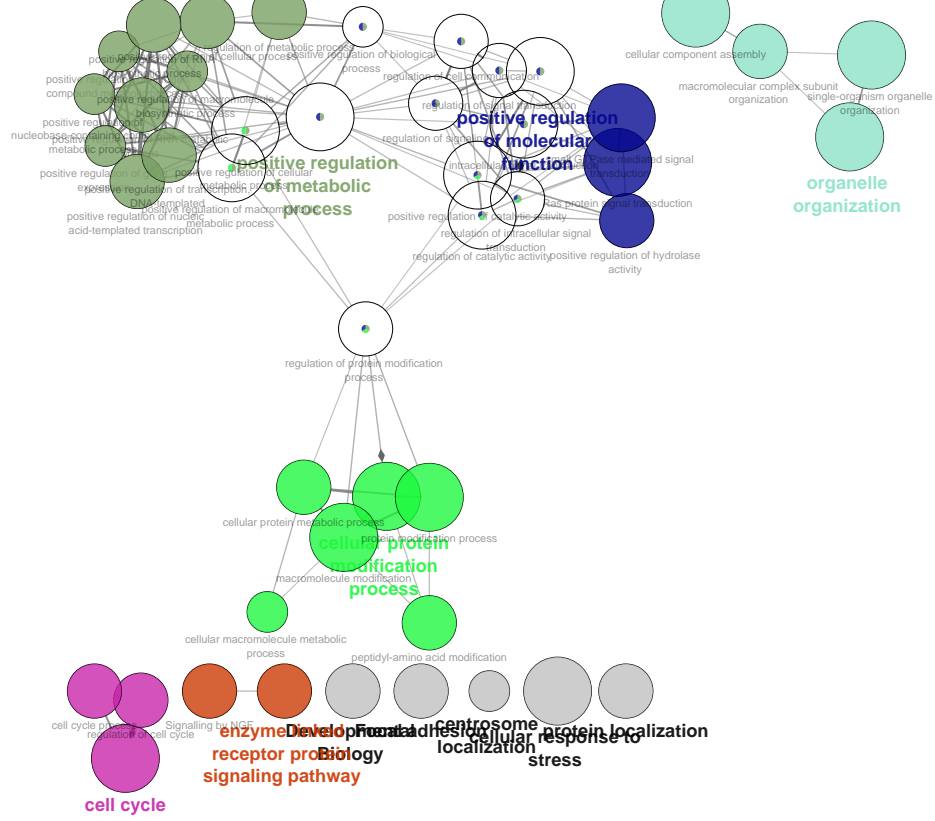

Supplement: Supplementary file 6 — Network analysis figures. All figures were converted to pdf files. (ZIP 47344 kb) [file 12192_2018_954_MOESM6_ESM.zip › Heart Lowland all - Cytoscape-ClueGo.pdf]

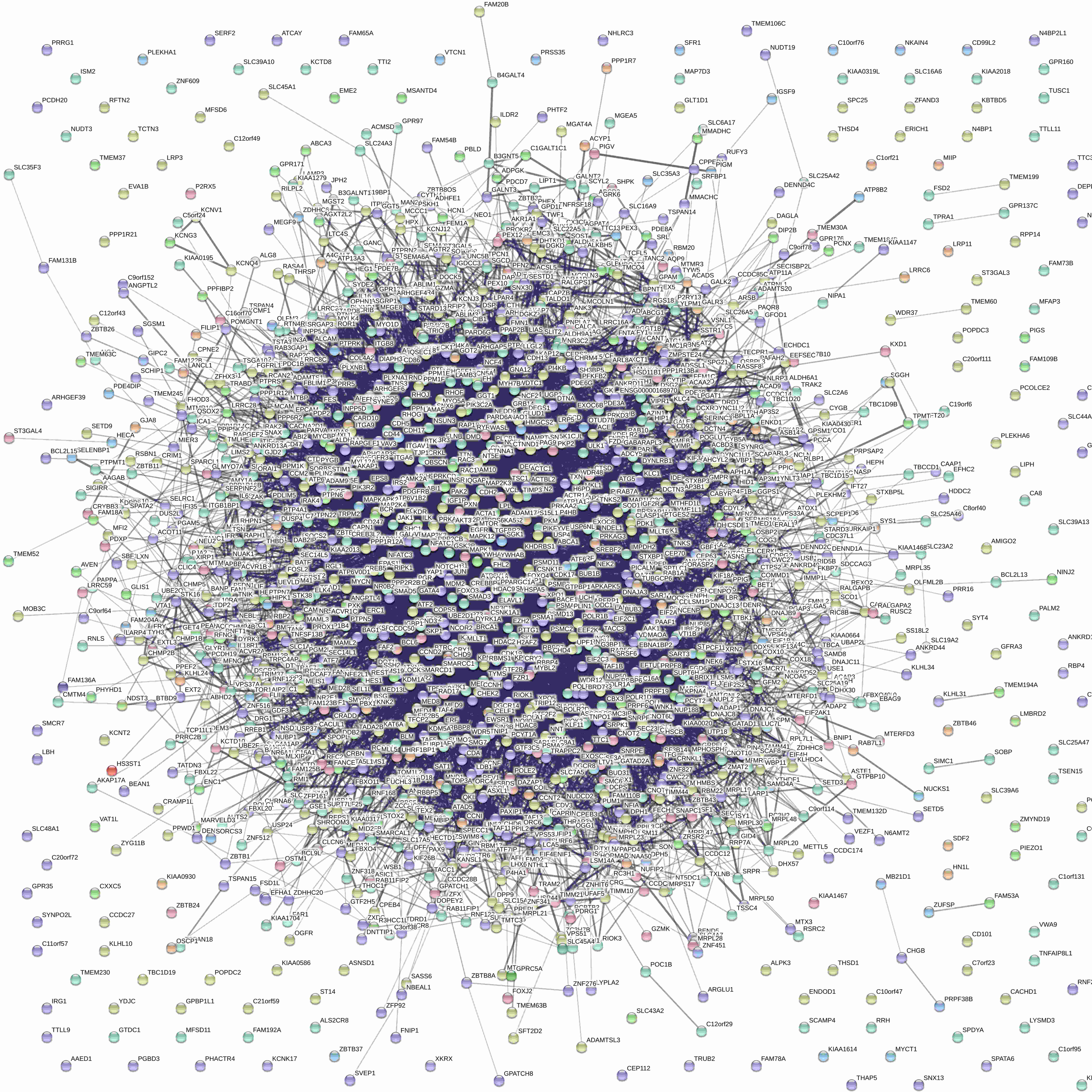

Supplement: Supplementary file 6 — Network analysis figures. All figures were converted to pdf files. (ZIP 47344 kb) [file 12192_2018_954_MOESM6_ESM.zip › Heart Lowland all - string.pdf]

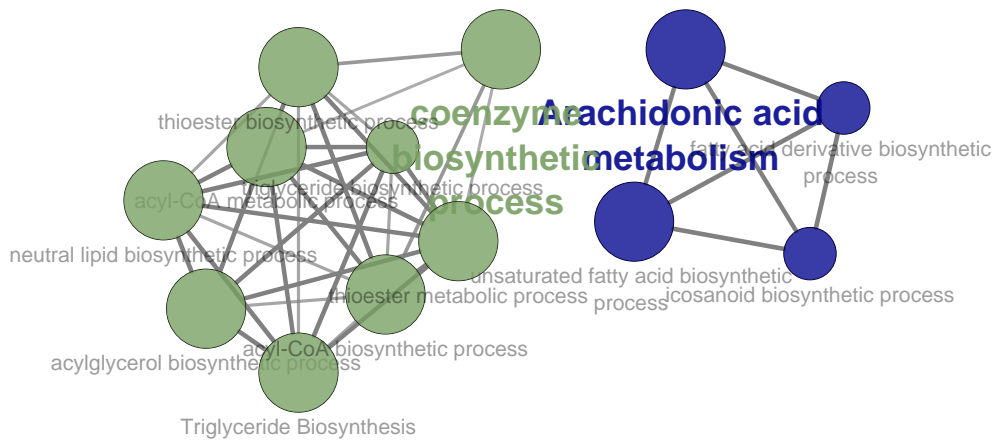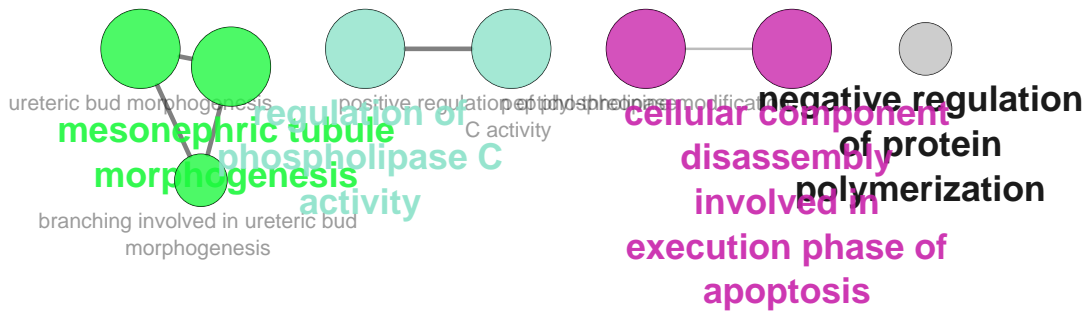

Supplement: Supplementary file 6 — Network analysis figures. All figures were converted to pdf files. (ZIP 47344 kb) [file 12192_2018_954_MOESM6_ESM.zip › Heart Lowland morning - noon - Cytoscape-ClueGo.pdf]

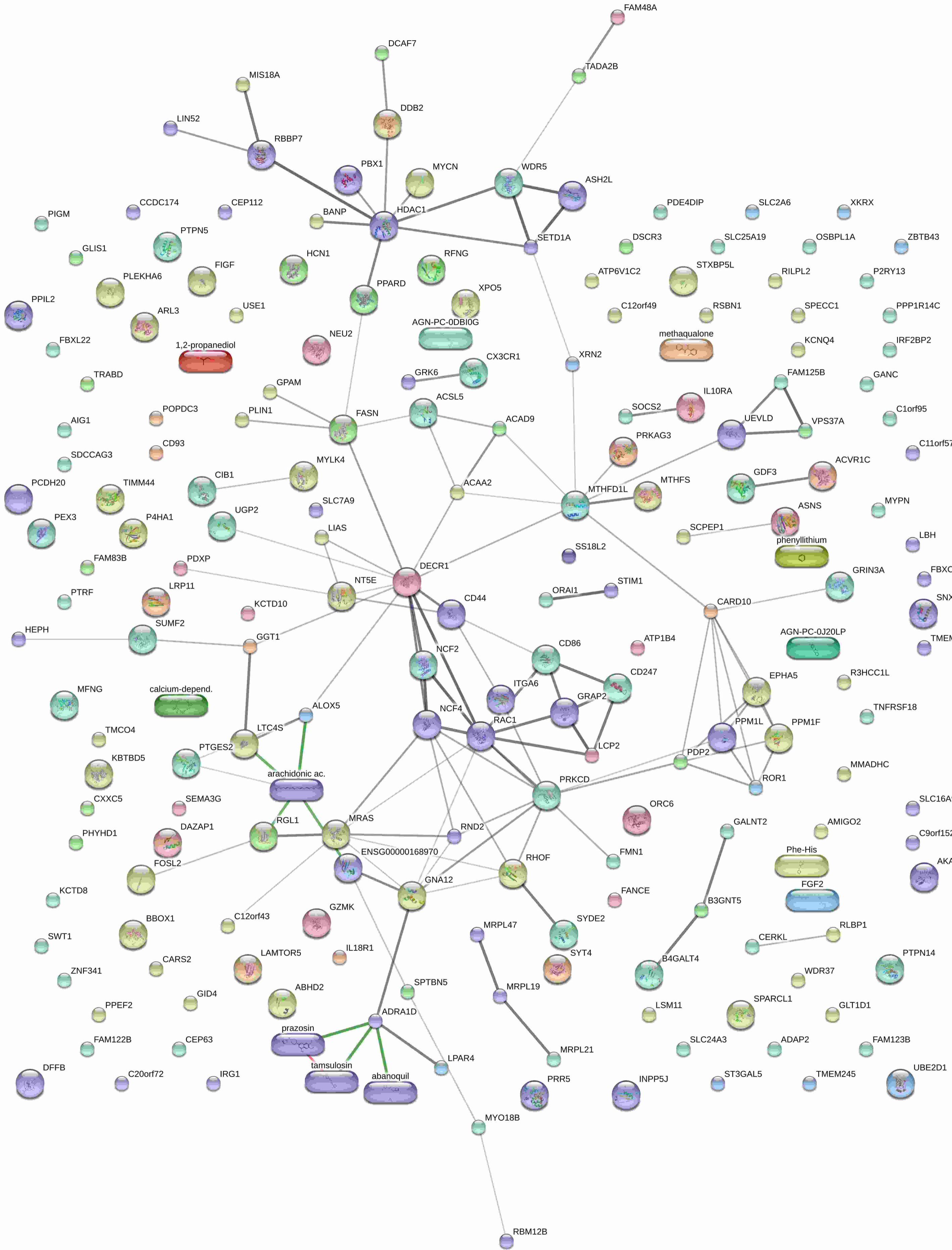

Supplement: Supplementary file 6 — Network analysis figures. All figures were converted to pdf files. (ZIP 47344 kb) [file 12192_2018_954_MOESM6_ESM.zip › Heart Lowland morning - noon - stitch.pdf]

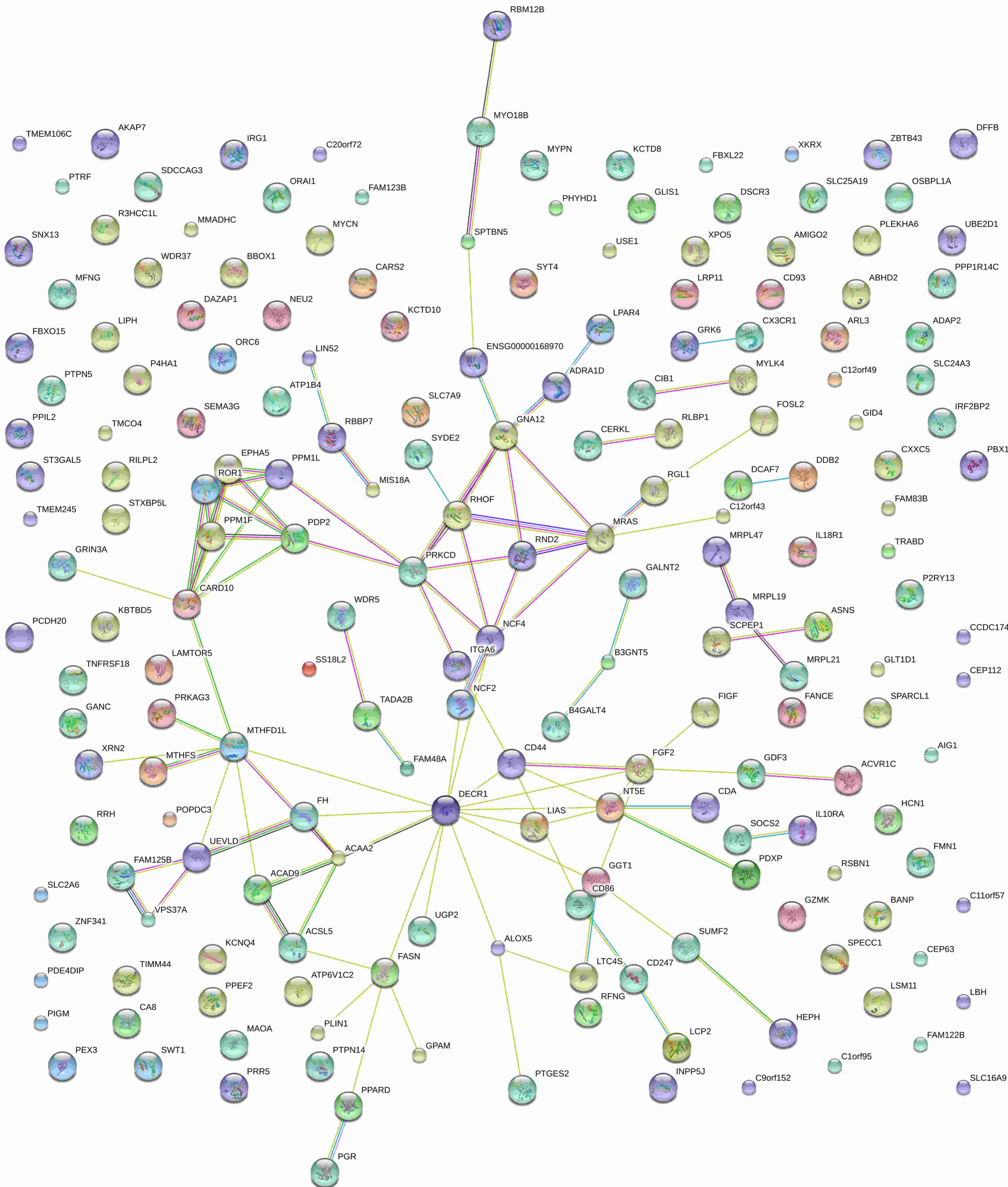

Supplement: Supplementary file 6 — Network analysis figures. All figures were converted to pdf files. (ZIP 47344 kb) [file 12192_2018_954_MOESM6_ESM.zip › Heart Lowland morning - noon - string.pdf]

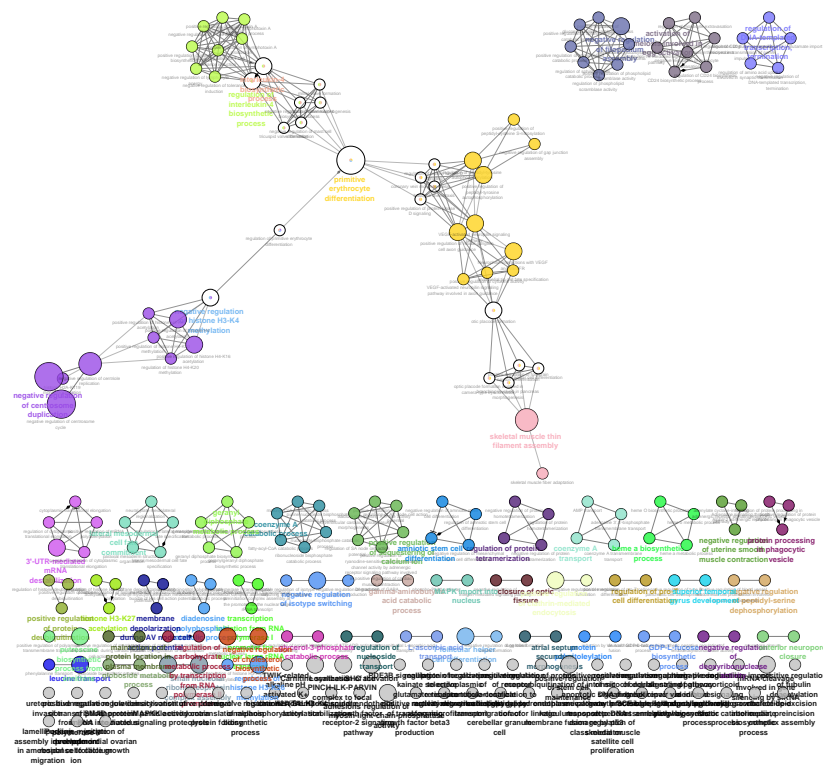

Supplement: Supplementary file 6 — Network analysis figures. All figures were converted to pdf files. (ZIP 47344 kb) [file 12192_2018_954_MOESM6_ESM.zip › Heart Lowland morning-evening - Cytoscape-ClueGo.pdf]

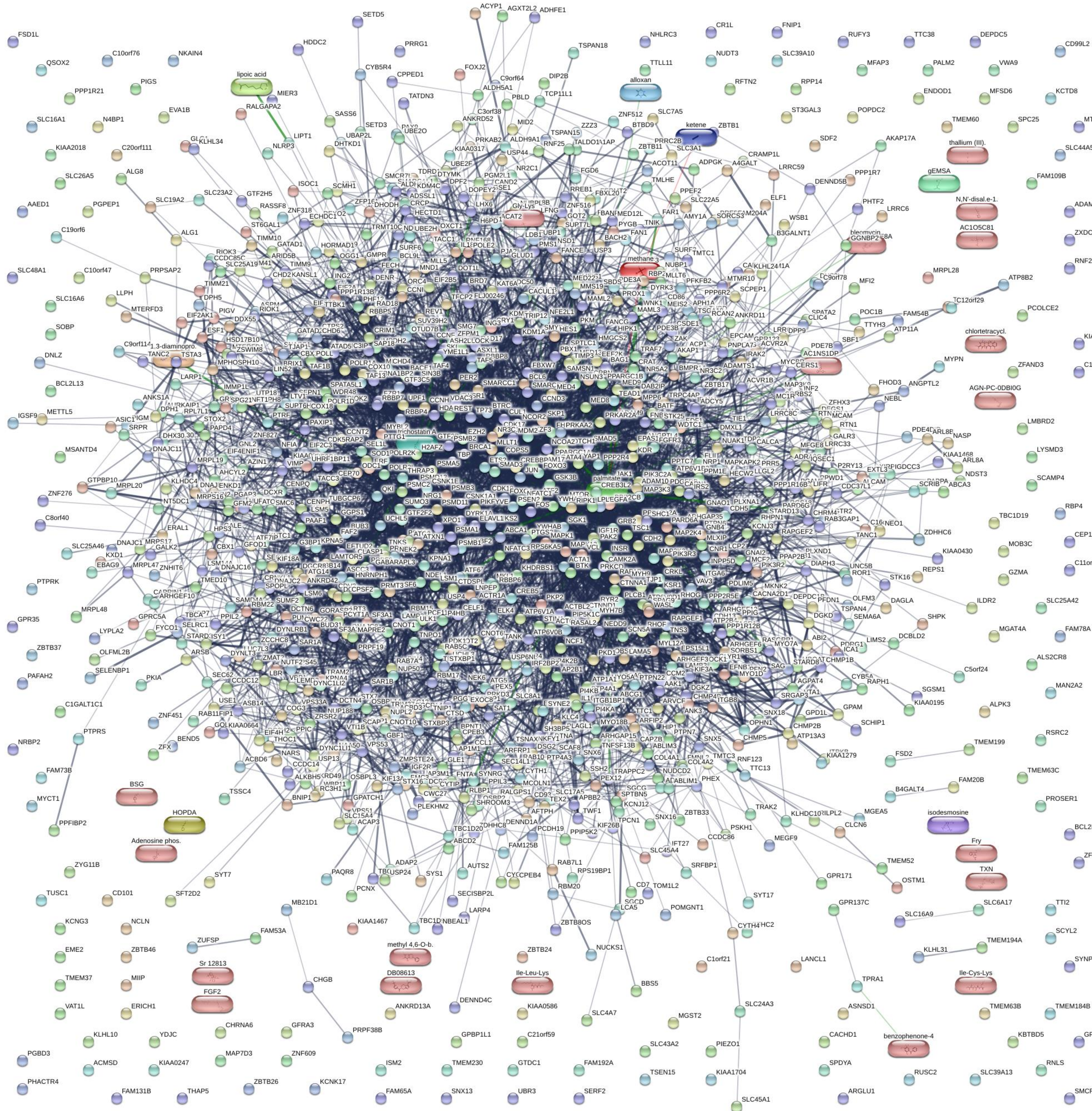

Supplement: Supplementary file 6 — Network analysis figures. All figures were converted to pdf files. (ZIP 47344 kb) [file 12192_2018_954_MOESM6_ESM.zip › Heart Lowland morning-evening - stitch.pdf]

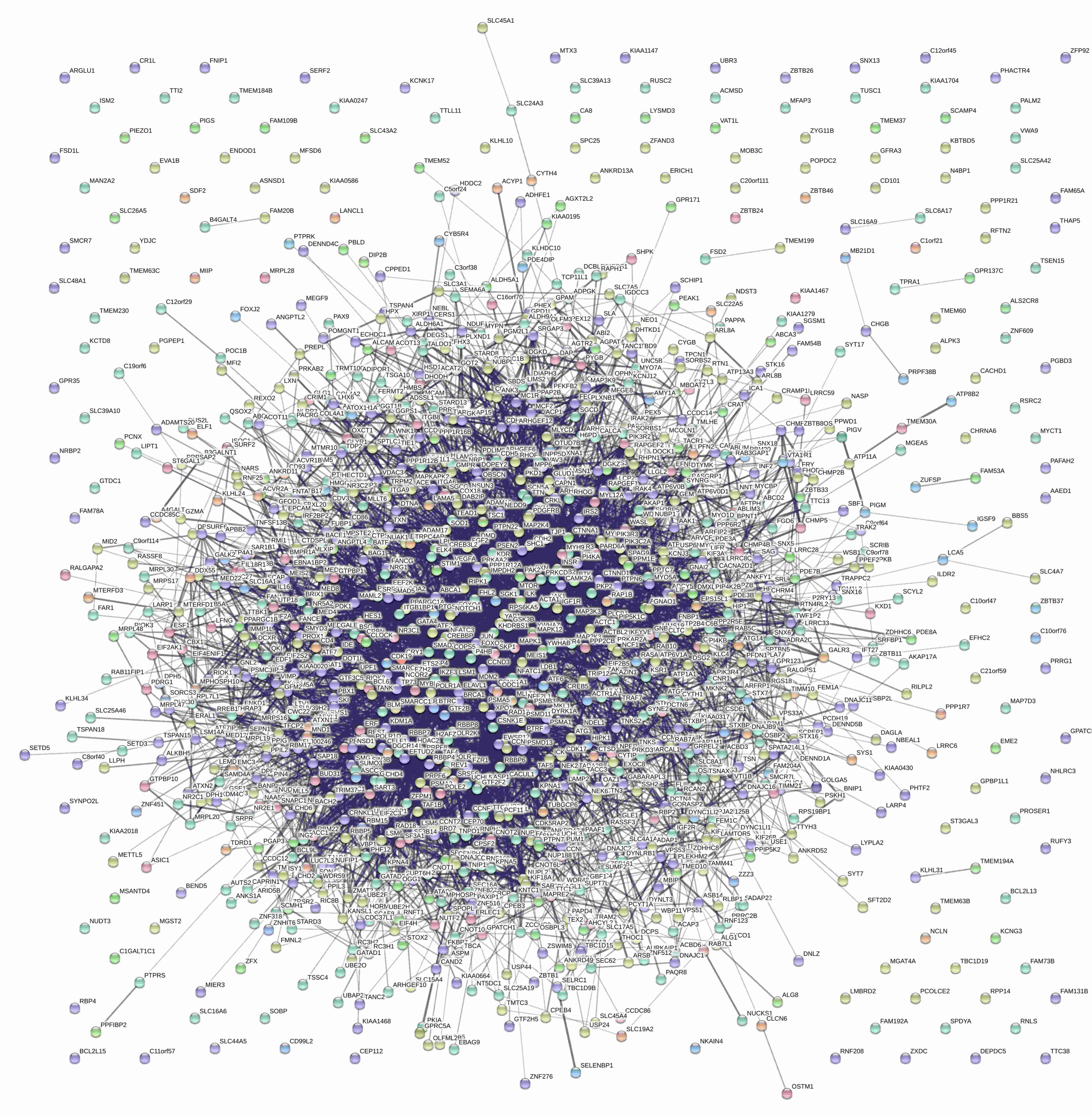

Supplement: Supplementary file 6 — Network analysis figures. All figures were converted to pdf files. (ZIP 47344 kb) [file 12192_2018_954_MOESM6_ESM.zip › Heart Lowland morning-evening - string.pdf]

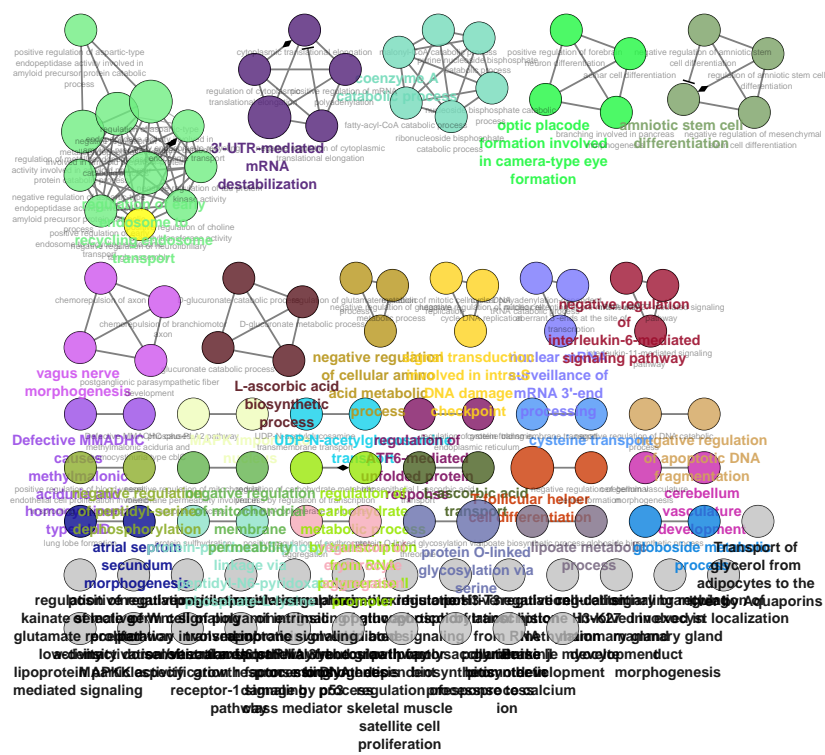

Supplement: Supplementary file 6 — Network analysis figures. All figures were converted to pdf files. (ZIP 47344 kb) [file 12192_2018_954_MOESM6_ESM.zip › Heart Lowland noon-evening - Cytoscape-ClueGo.pdf]

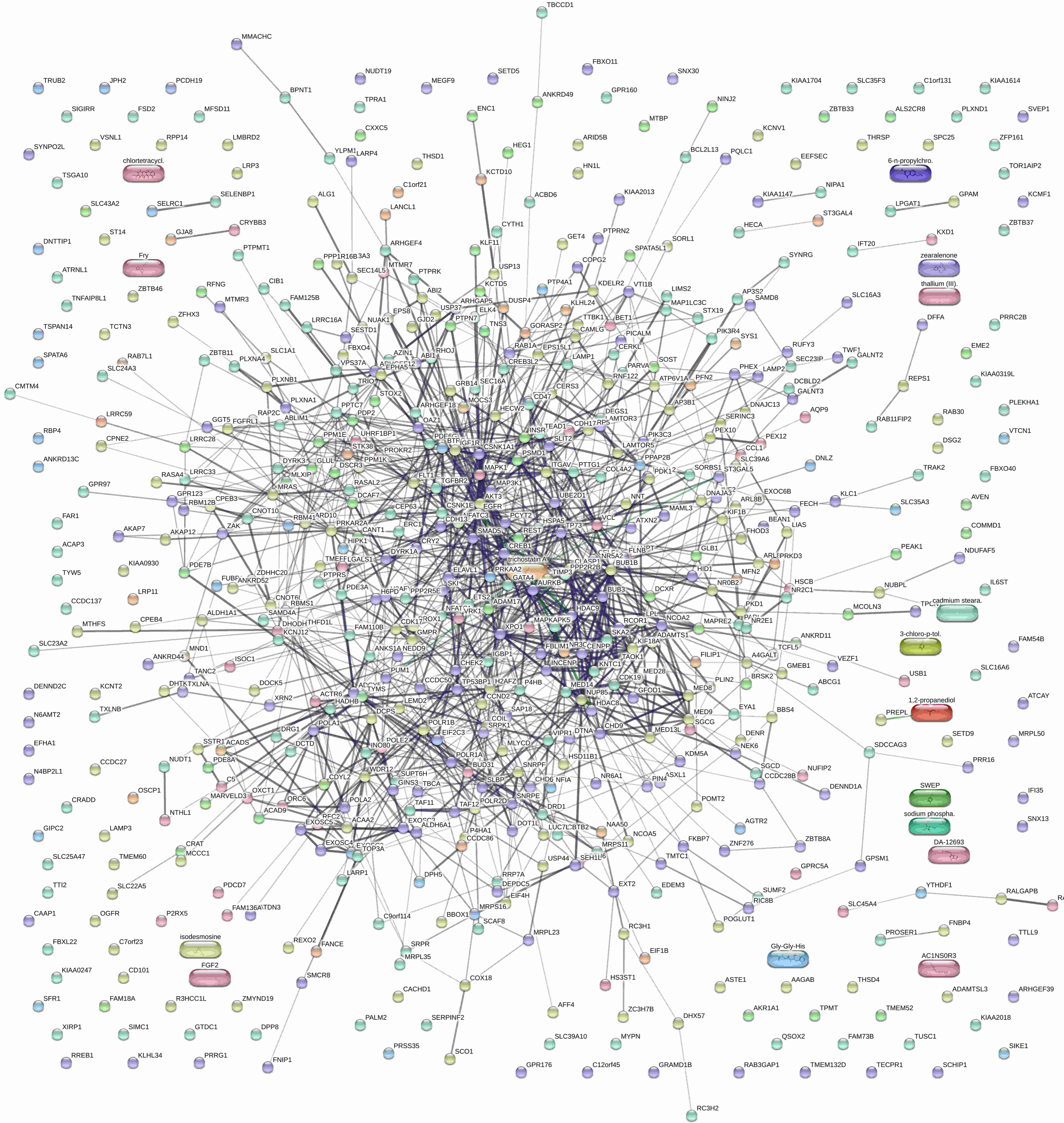

Supplement: Supplementary file 6 — Network analysis figures. All figures were converted to pdf files. (ZIP 47344 kb) [file 12192_2018_954_MOESM6_ESM.zip › Heart Lowland noon-evening - stitch.pdf]

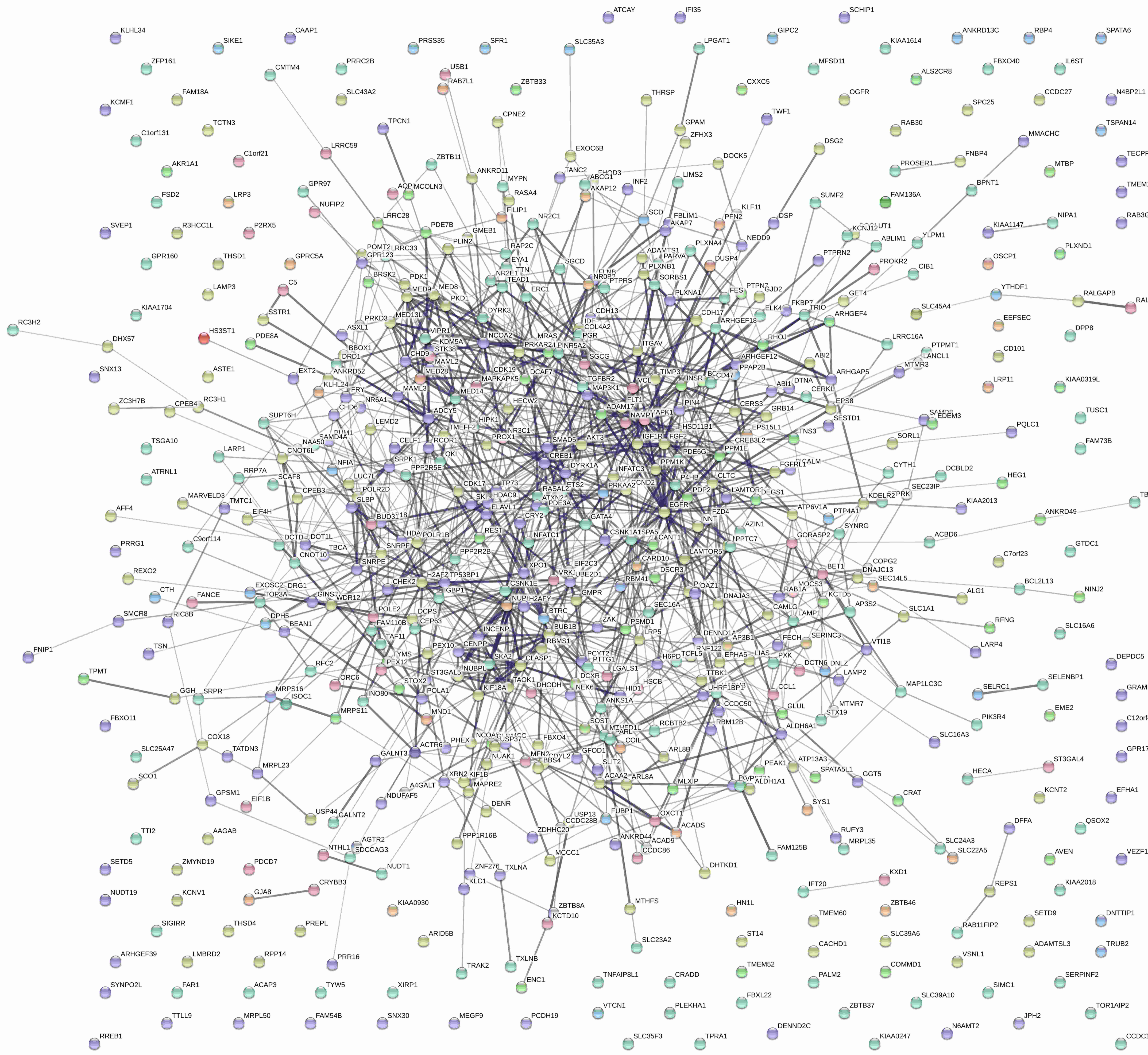

Supplement: Supplementary file 6 — Network analysis figures. All figures were converted to pdf files. (ZIP 47344 kb) [file 12192_2018_954_MOESM6_ESM.zip › Heart Lowland noon-evening - string.pdf]

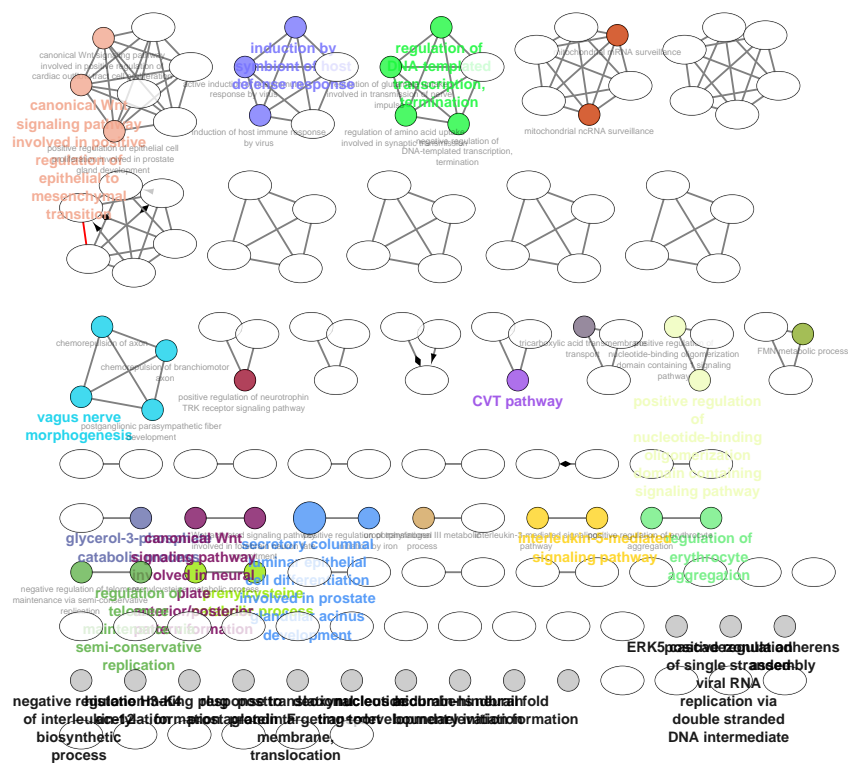

Supplement: Supplementary file 6 — Network analysis figures. All figures were converted to pdf files. (ZIP 47344 kb) [file 12192_2018_954_MOESM6_ESM.zip › Muscle Highland all - Cytoscape-ClueGo.pdf]

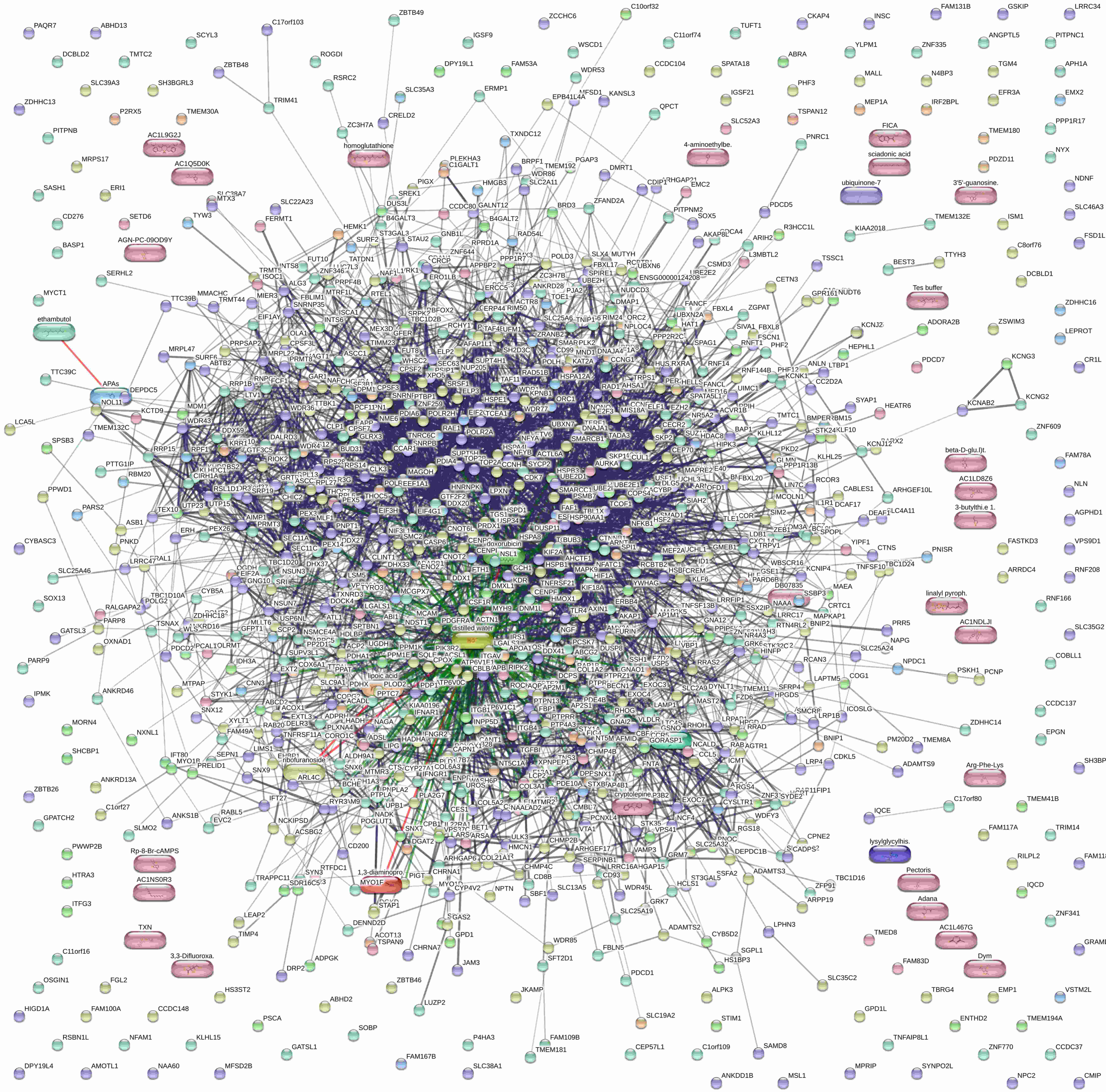

Supplement: Supplementary file 6 — Network analysis figures. All figures were converted to pdf files. (ZIP 47344 kb) [file 12192_2018_954_MOESM6_ESM.zip › Muscle Highland all - stitch.pdf]

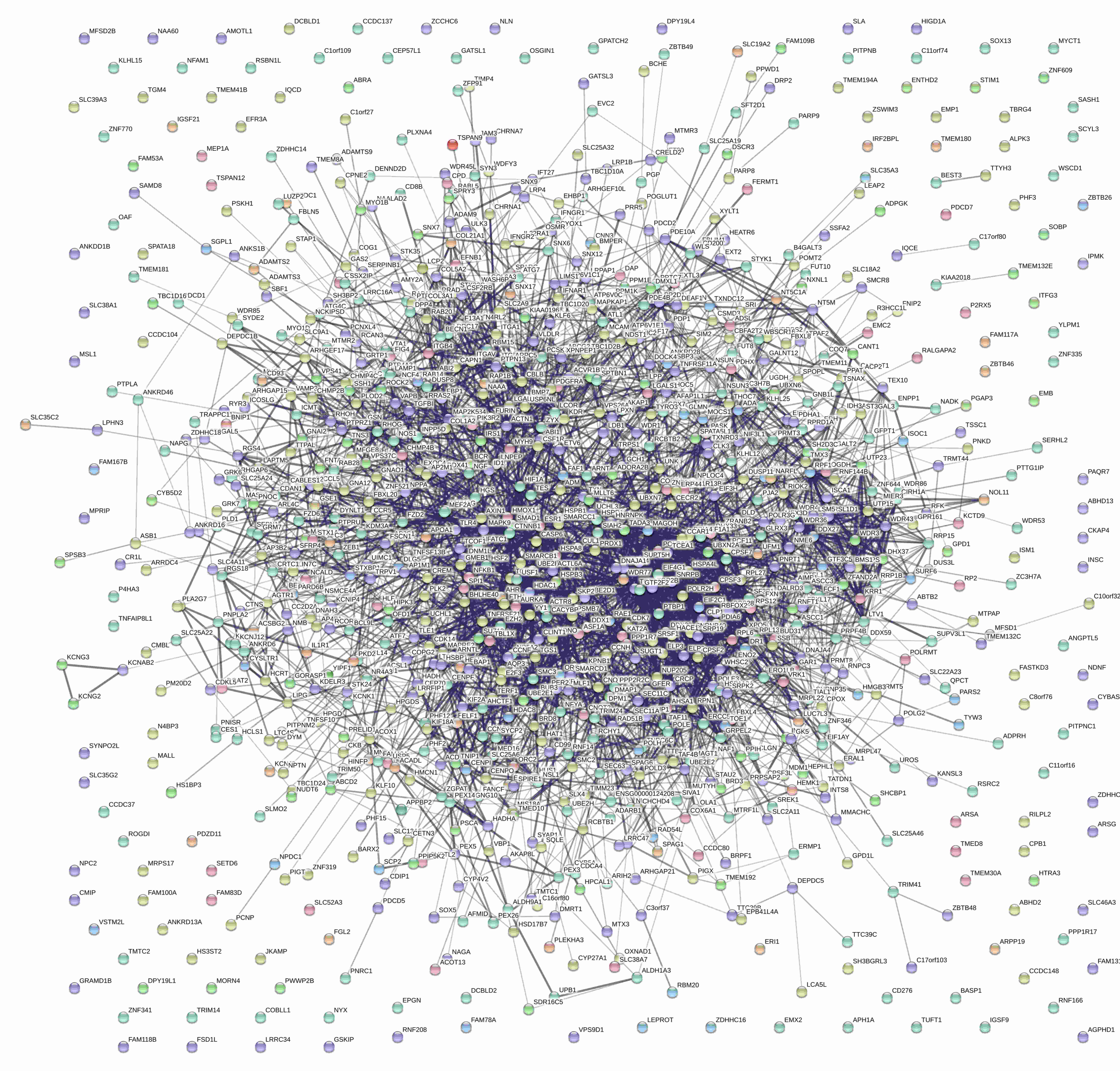

Supplement: Supplementary file 6 — Network analysis figures. All figures were converted to pdf files. (ZIP 47344 kb) [file 12192_2018_954_MOESM6_ESM.zip › Muscle Highland all - string.pdf]

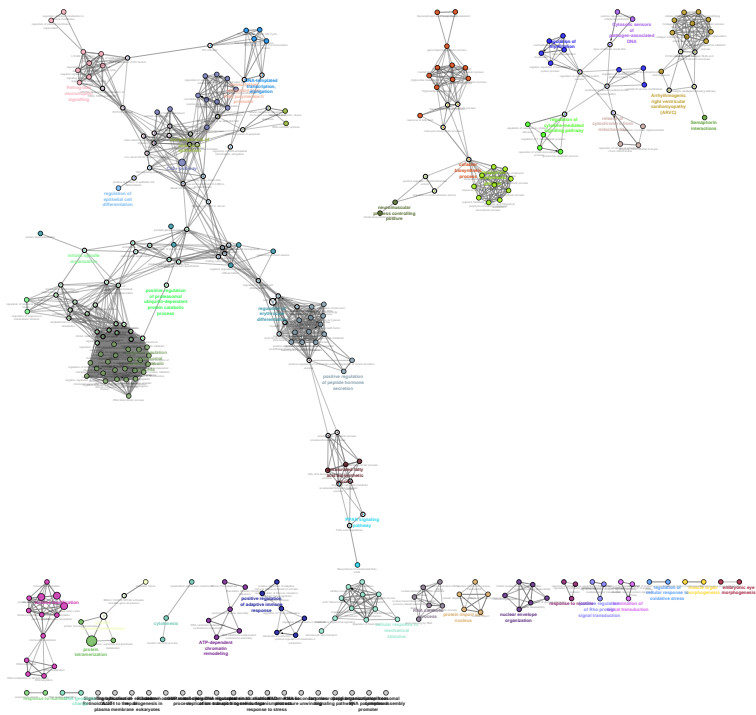

Supplement: Supplementary file 6 — Network analysis figures. All figures were converted to pdf files. (ZIP 47344 kb) [file 12192_2018_954_MOESM6_ESM.zip › Muscle Highland morning-evening - Cytoscape-ClueGo.pdf]

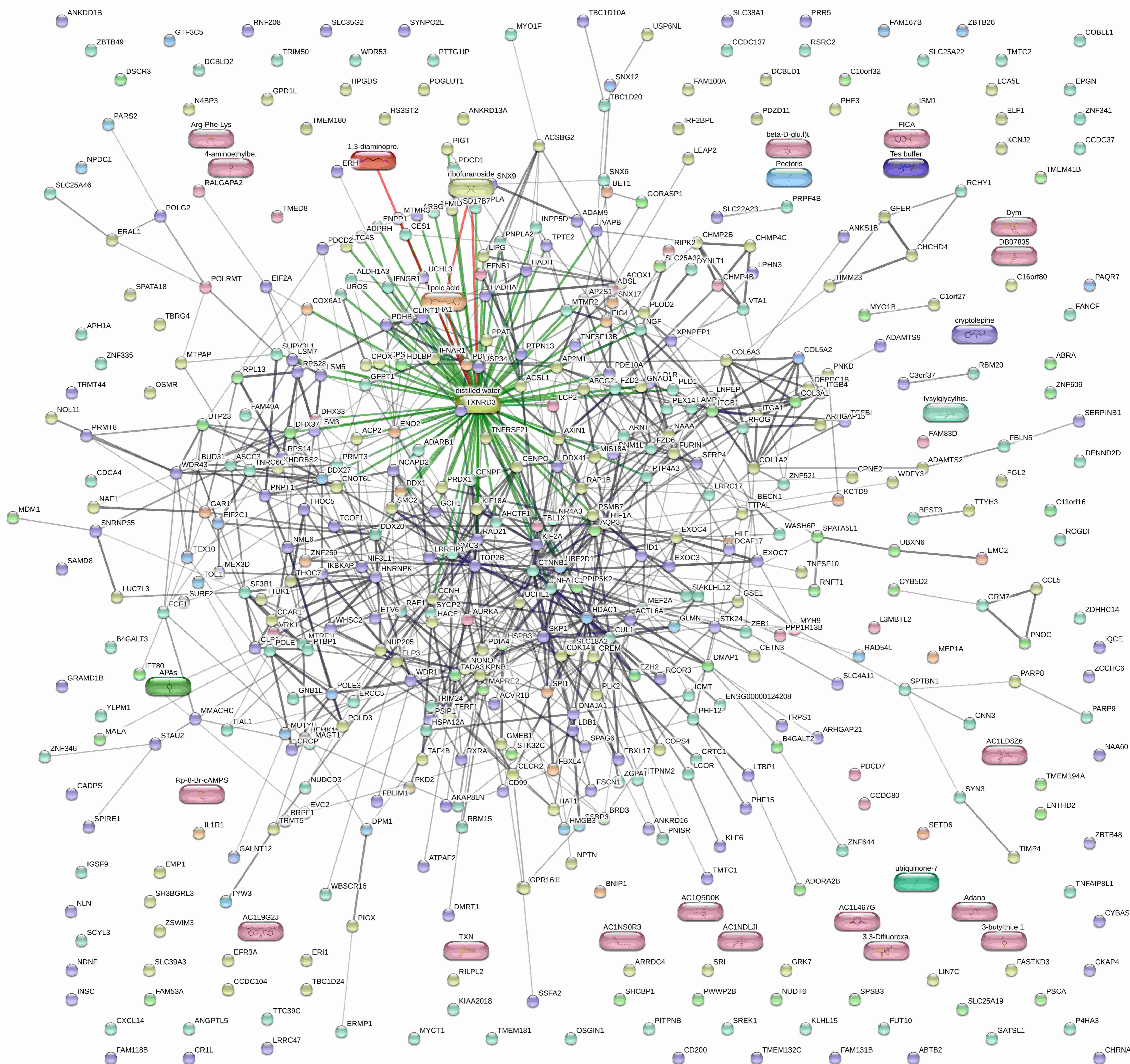

Supplement: Supplementary file 6 — Network analysis figures. All figures were converted to pdf files. (ZIP 47344 kb) [file 12192_2018_954_MOESM6_ESM.zip › Muscle Highland morning-evening - stitch.pdf]

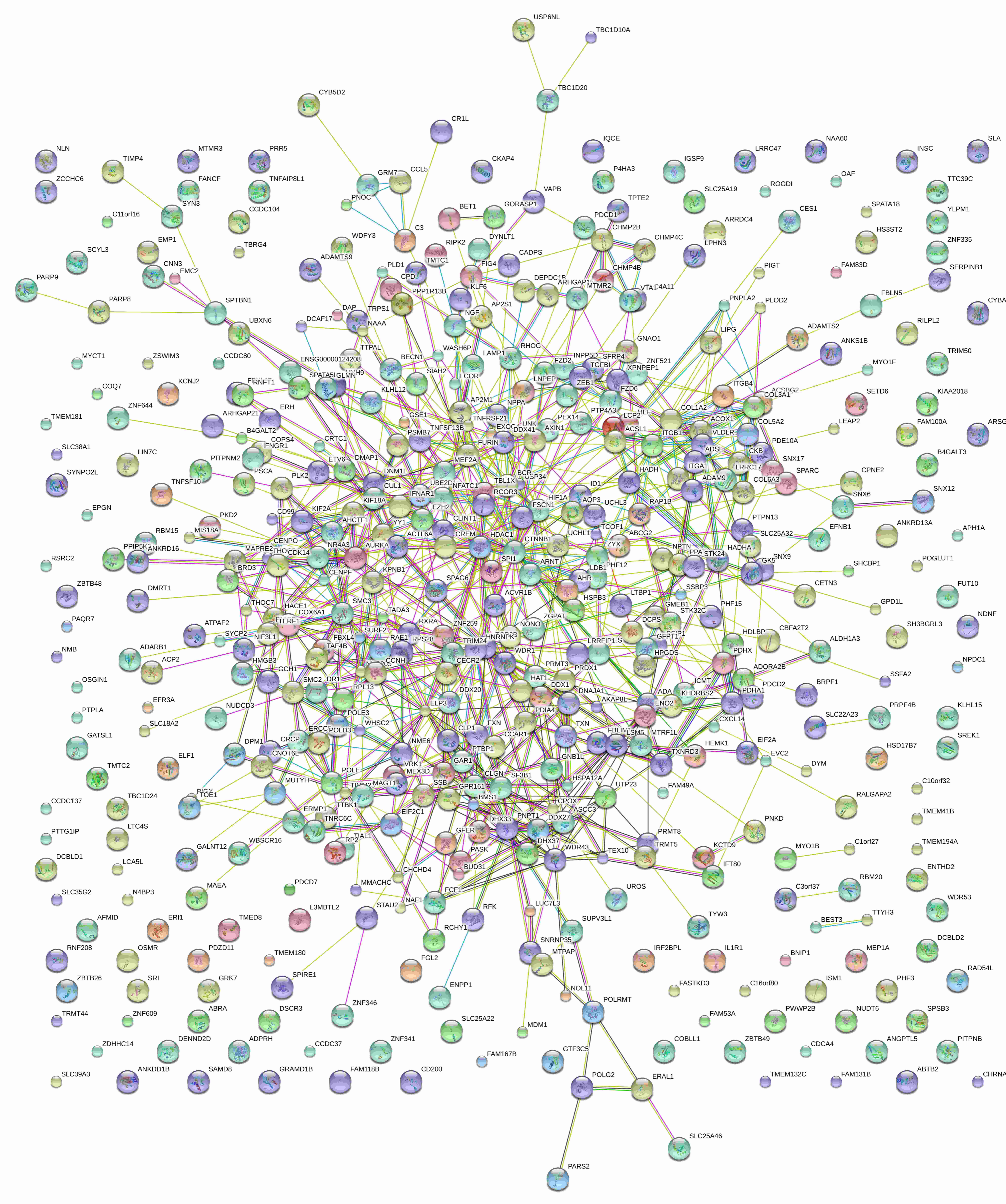

Supplement: Supplementary file 6 — Network analysis figures. All figures were converted to pdf files. (ZIP 47344 kb) [file 12192_2018_954_MOESM6_ESM.zip › Muscle highland morning-evening - string.pdf]

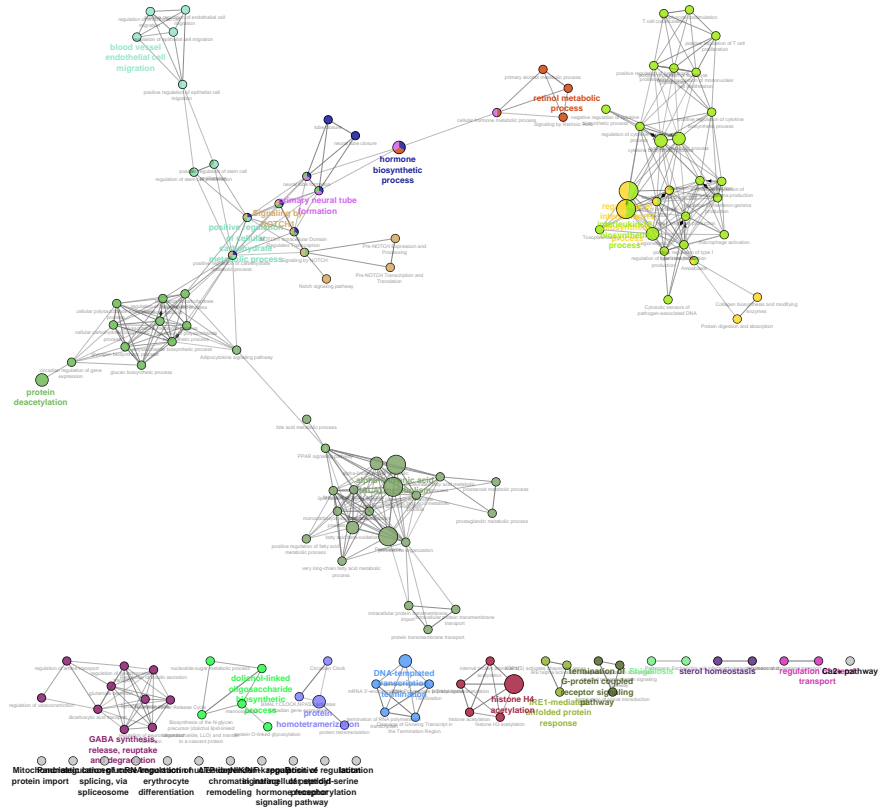

Supplement: Supplementary file 6 — Network analysis figures. All figures were converted to pdf files. (ZIP 47344 kb) [file 12192_2018_954_MOESM6_ESM.zip › Muscle Highland morning-noon - Cytoscape-ClueGo.pdf]

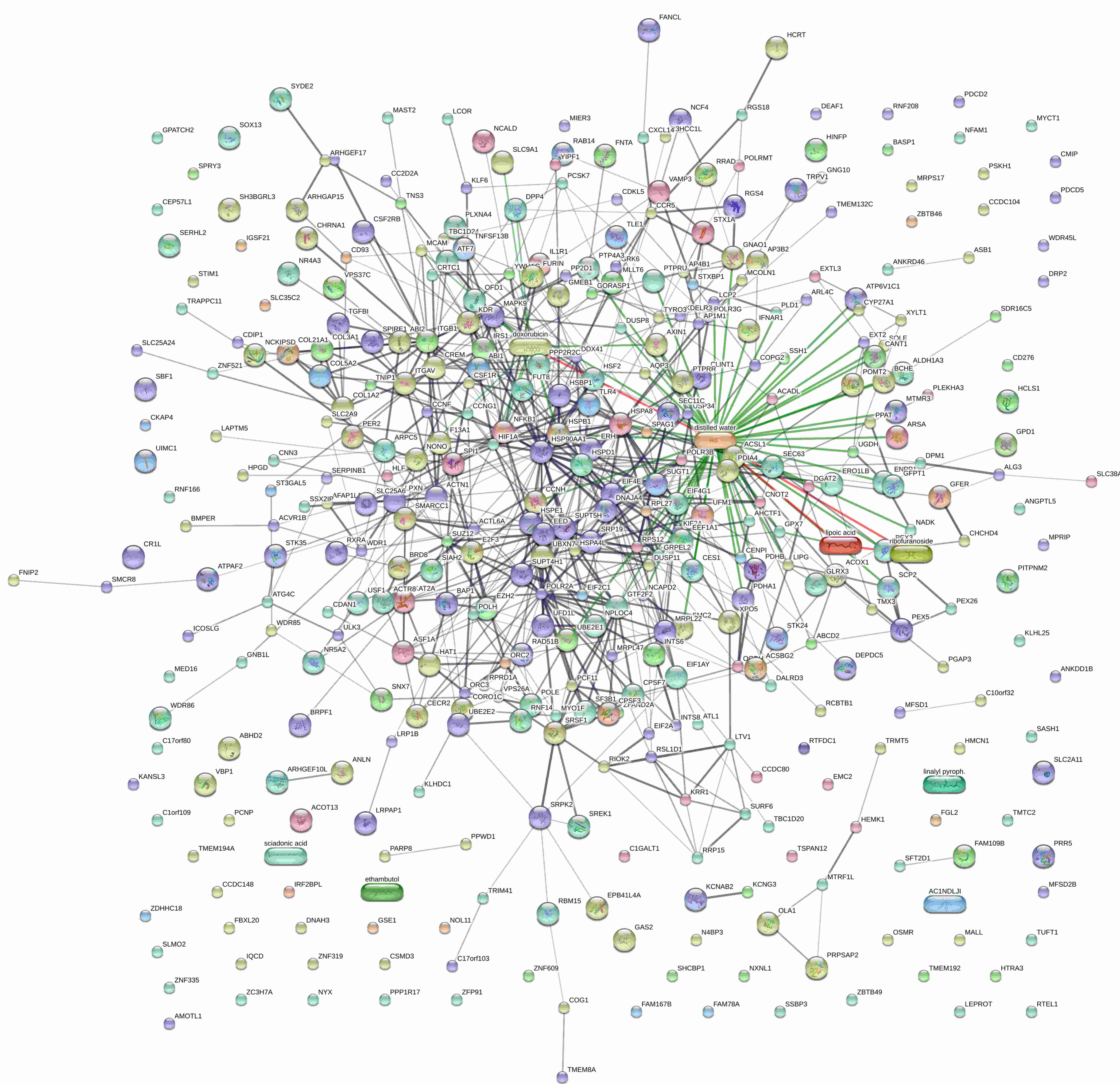

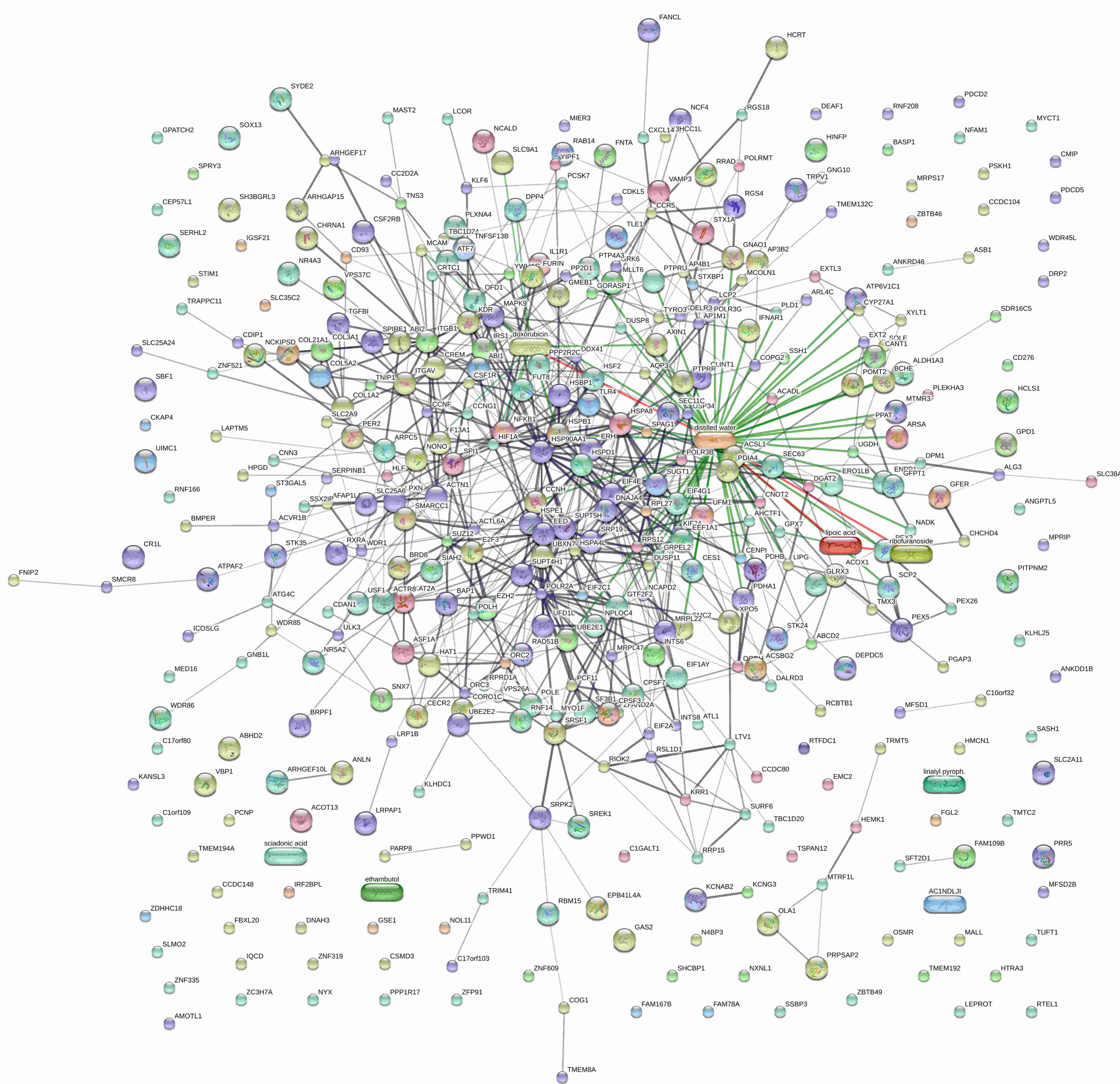

Supplement: Supplementary file 6 — Network analysis figures. All figures were converted to pdf files. (ZIP 47344 kb) [file 12192_2018_954_MOESM6_ESM.zip › Muscle Highland morning-noon - stitch.pdf]

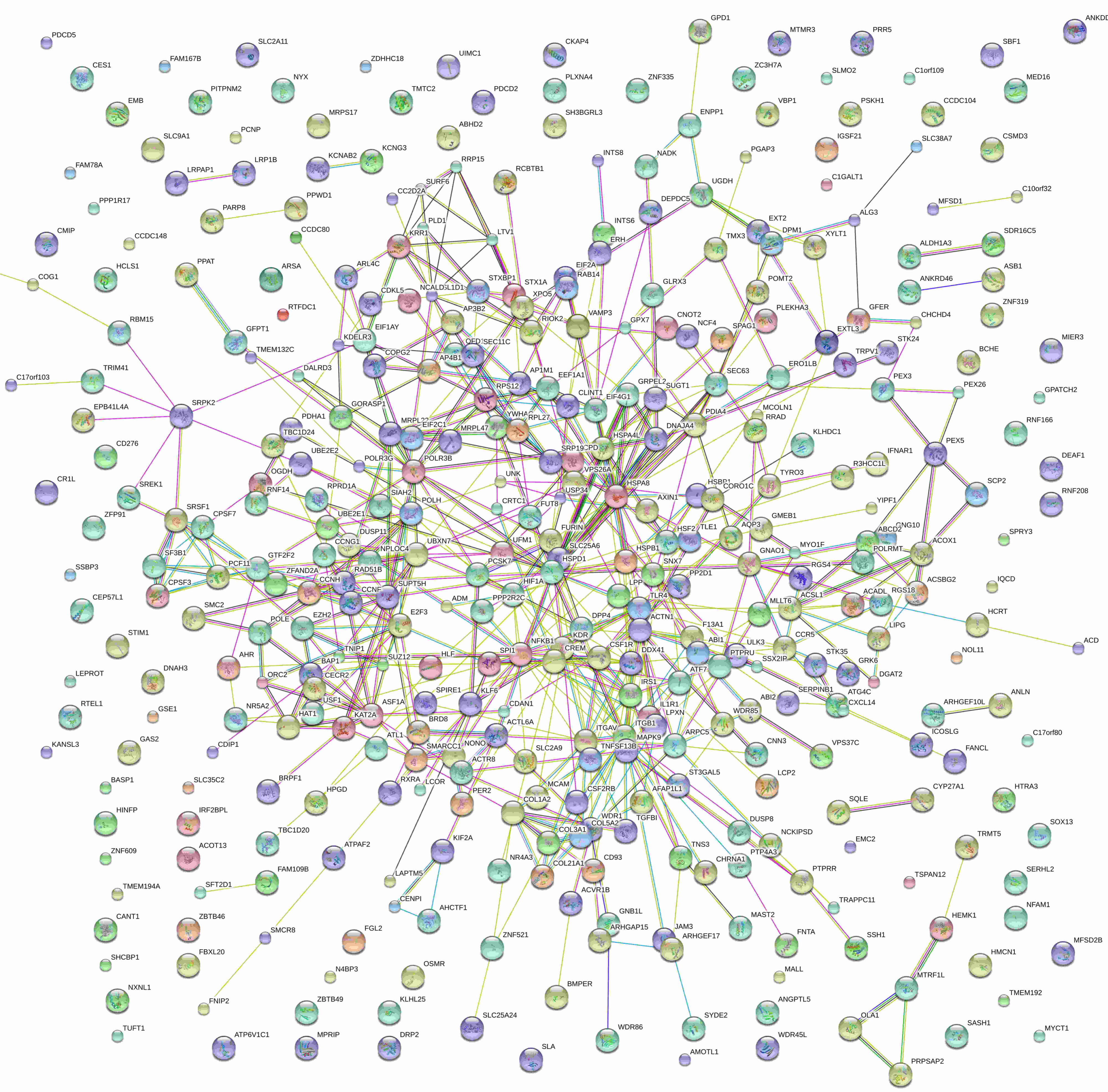

Supplement: Supplementary file 6 — Network analysis figures. All figures were converted to pdf files. (ZIP 47344 kb) [file 12192_2018_954_MOESM6_ESM.zip › Muscle Highland morning-noon - string.pdf]

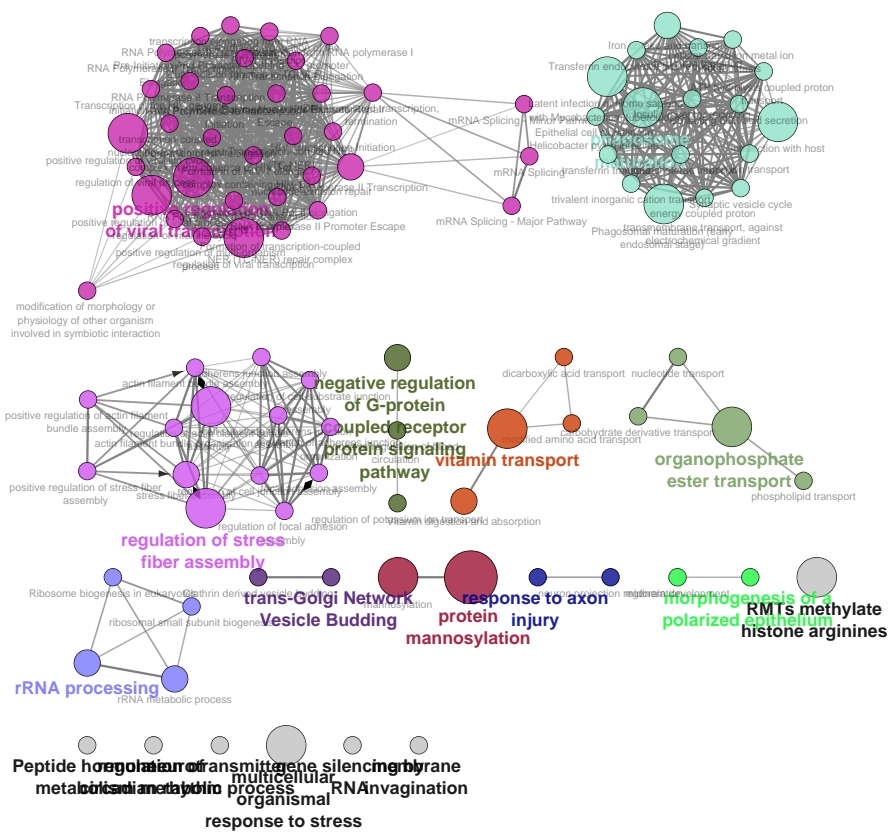

Supplement: Supplementary file 6 — Network analysis figures. All figures were converted to pdf files. (ZIP 47344 kb) [file 12192_2018_954_MOESM6_ESM.zip › Muscle Highland noon-evening - Cytoscape-ClueGo.pdf]

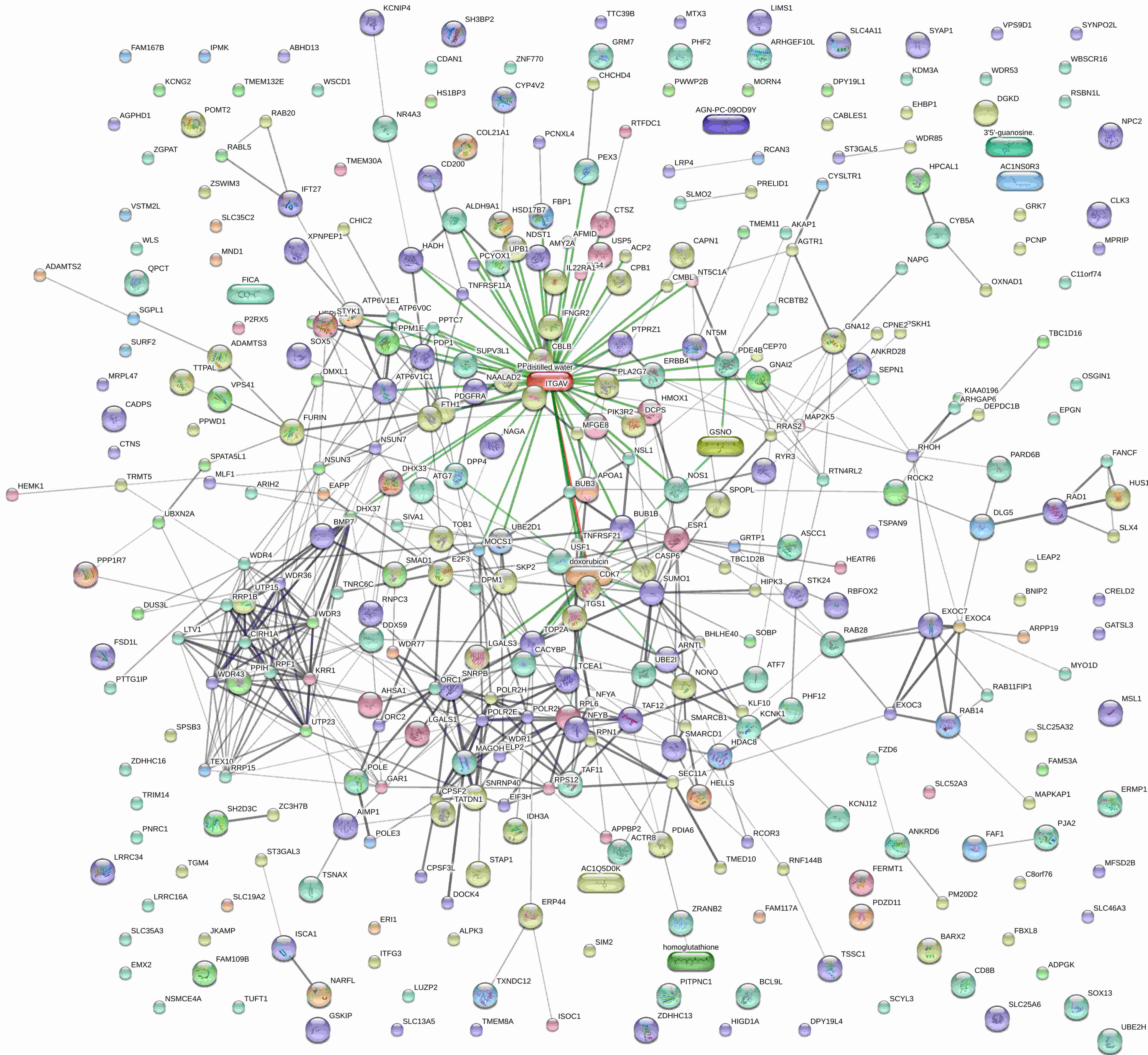

Supplement: Supplementary file 6 — Network analysis figures. All figures were converted to pdf files. (ZIP 47344 kb) [file 12192_2018_954_MOESM6_ESM.zip › Muscle Highland noon-evening - stitch.pdf]

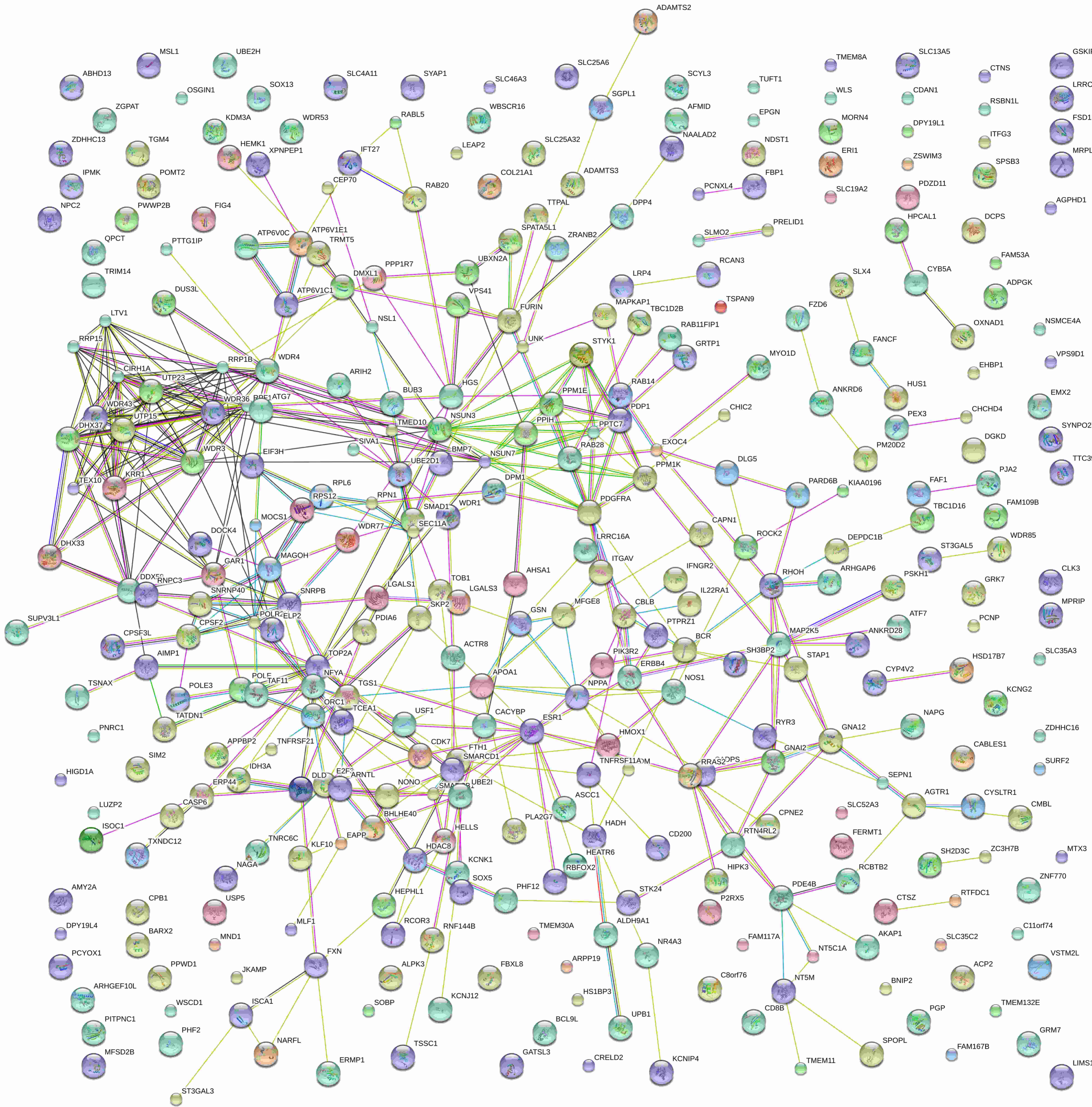

Supplement: Supplementary file 6 — Network analysis figures. All figures were converted to pdf files. (ZIP 47344 kb) [file 12192_2018_954_MOESM6_ESM.zip › Muscle Highland noon-evening - string.pdf]

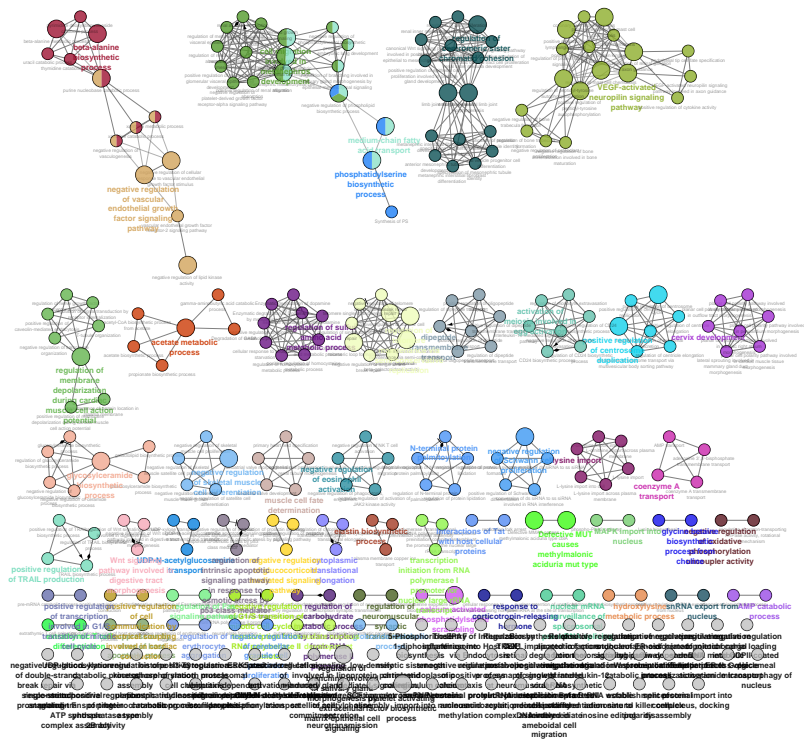

Supplement: Supplementary file 6 — Network analysis figures. All figures were converted to pdf files. (ZIP 47344 kb) [file 12192_2018_954_MOESM6_ESM.zip › Muscle Highland-lowland all - Cytoscape-ClueGo.pdf]

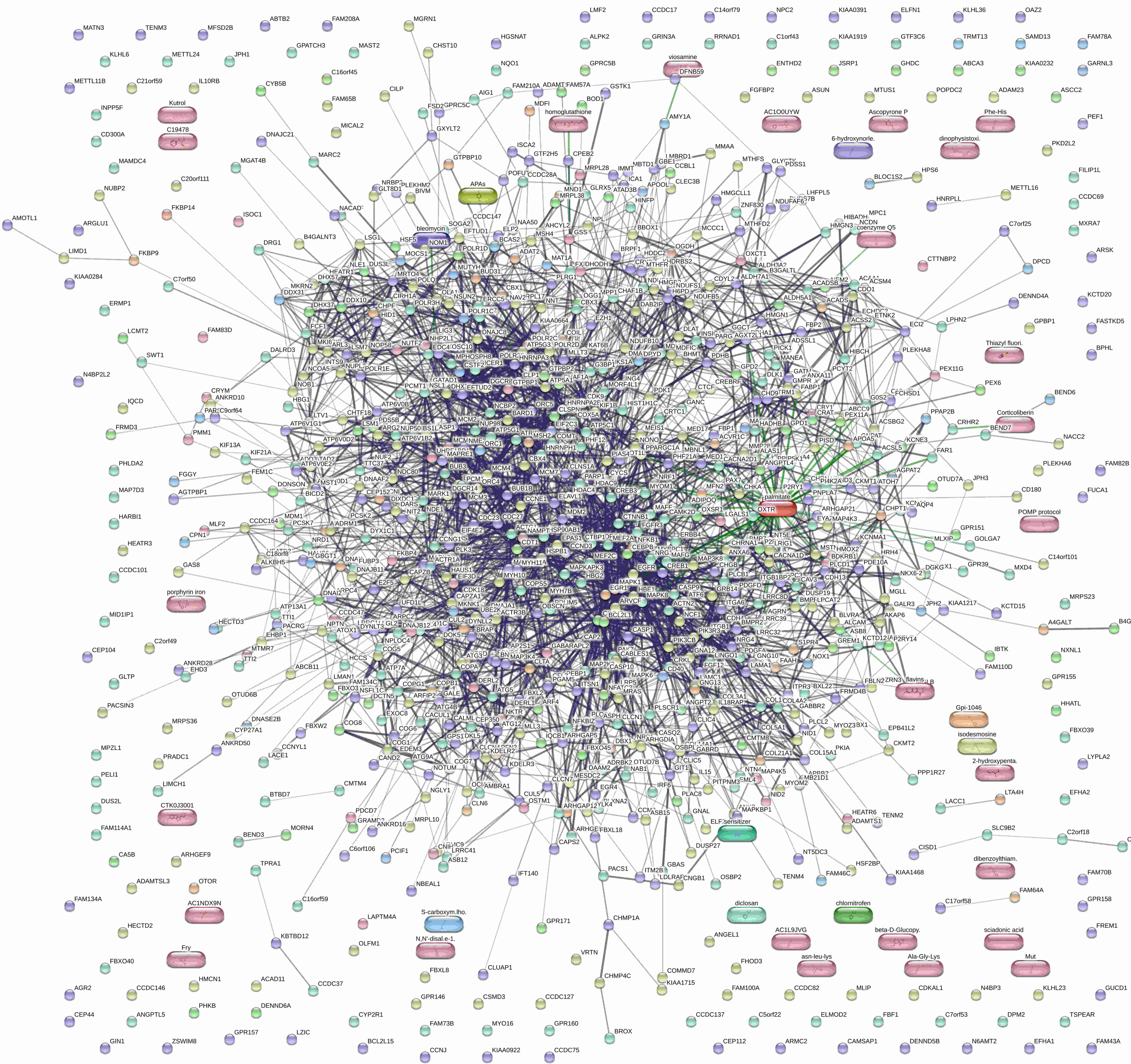

Supplement: Supplementary file 6 — Network analysis figures. All figures were converted to pdf files. (ZIP 47344 kb) [file 12192_2018_954_MOESM6_ESM.zip › Muscle Highland-lowland all - stitch.pdf]

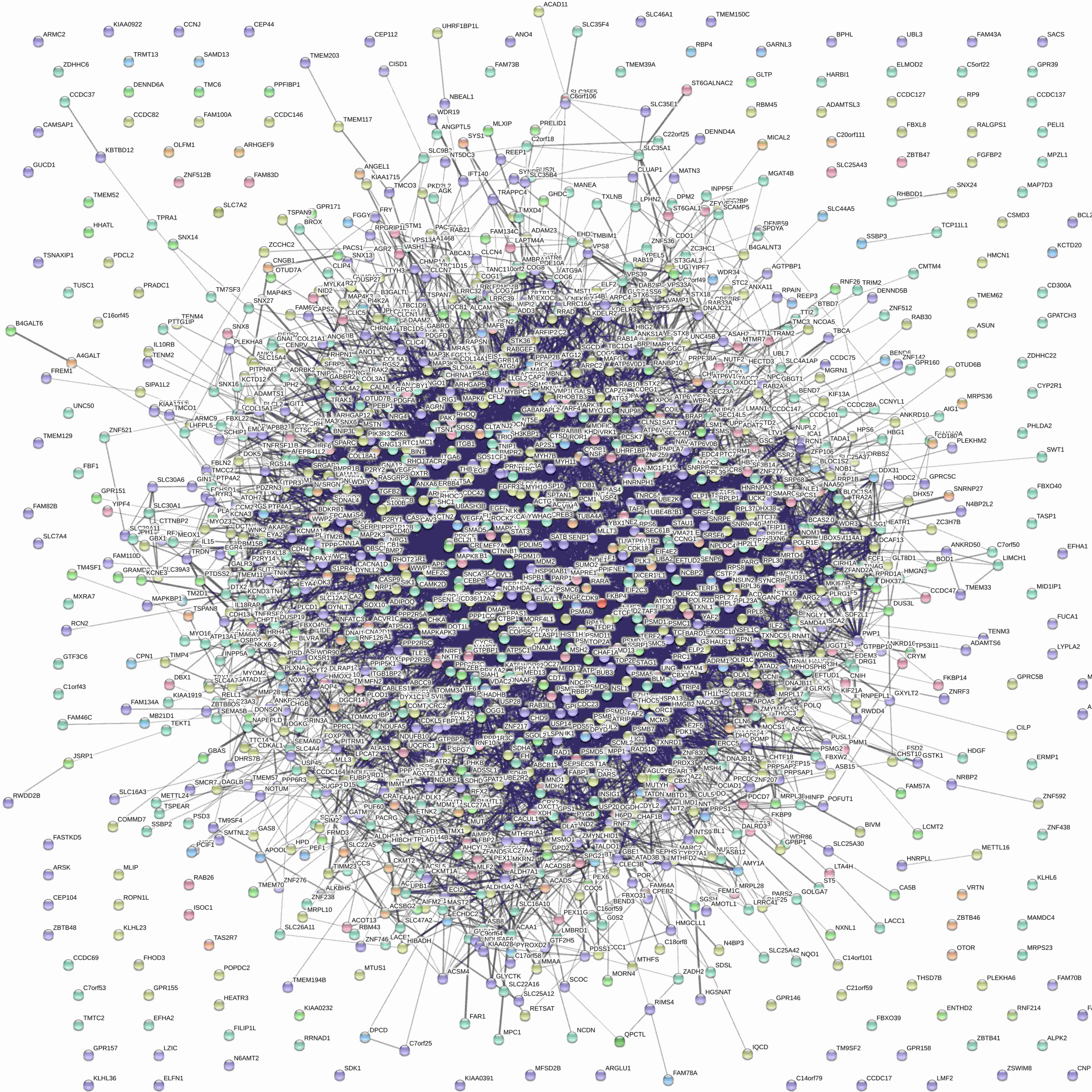

Supplement: Supplementary file 6 — Network analysis figures. All figures were converted to pdf files. (ZIP 47344 kb) [file 12192_2018_954_MOESM6_ESM.zip › Muscle Highland-lowland all - string.pdf]

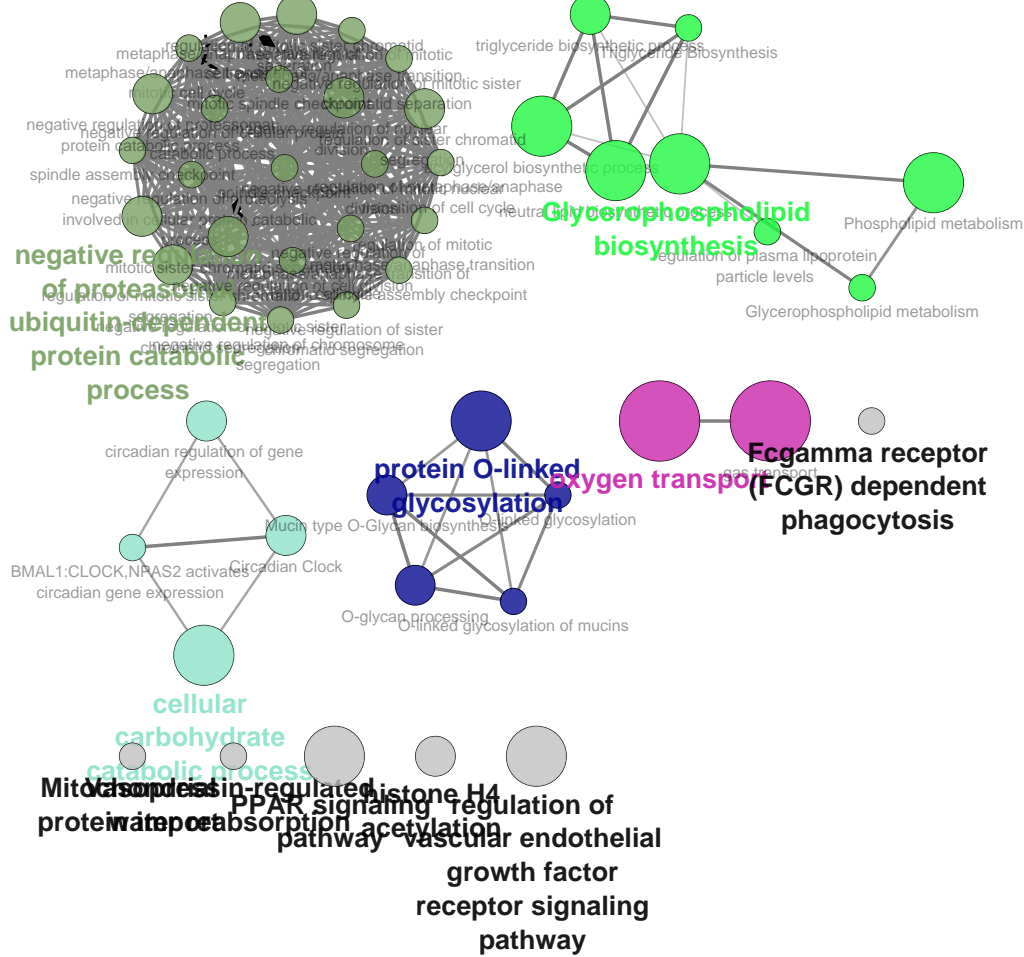

Supplement: Supplementary file 6 — Network analysis figures. All figures were converted to pdf files. (ZIP 47344 kb) [file 12192_2018_954_MOESM6_ESM.zip › Muscle Highland-lowland evening - Cytoscape-ClueGo.pdf]

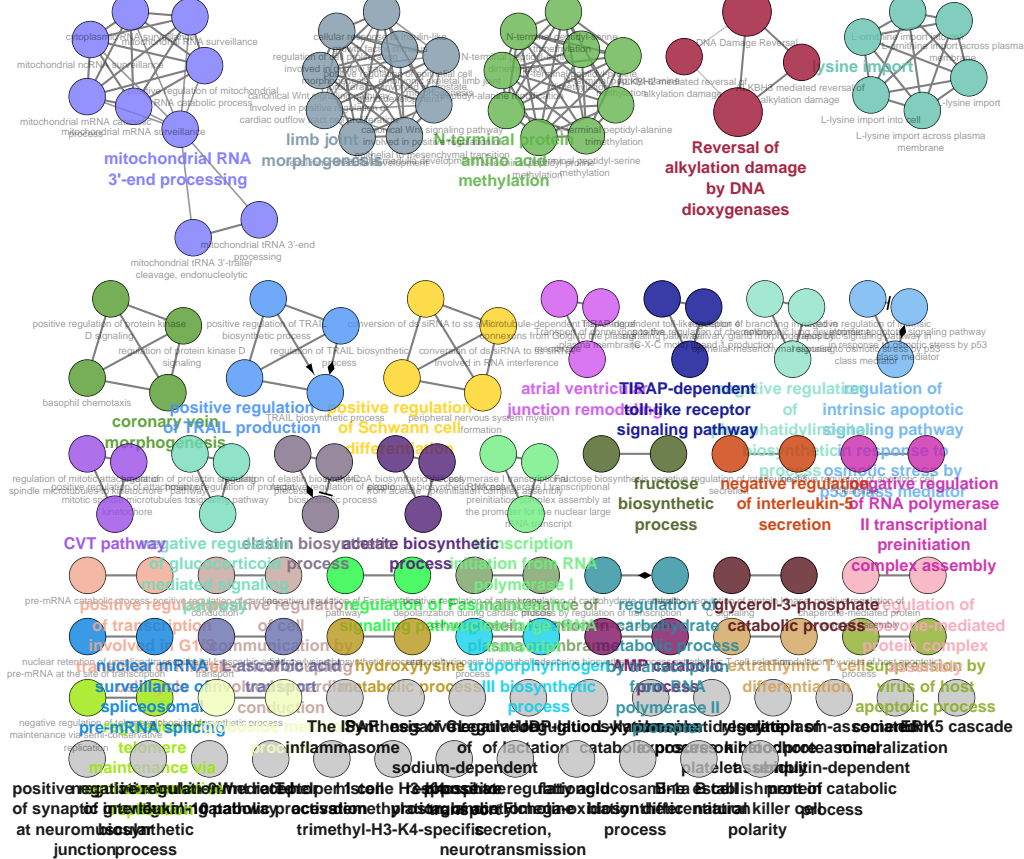

Supplement: Supplementary file 6 — Network analysis figures. All figures were converted to pdf files. (ZIP 47344 kb) [file 12192_2018_954_MOESM6_ESM.zip › Muscle Highland-lowland evening - Cytoscape-ClueGo.pdf]

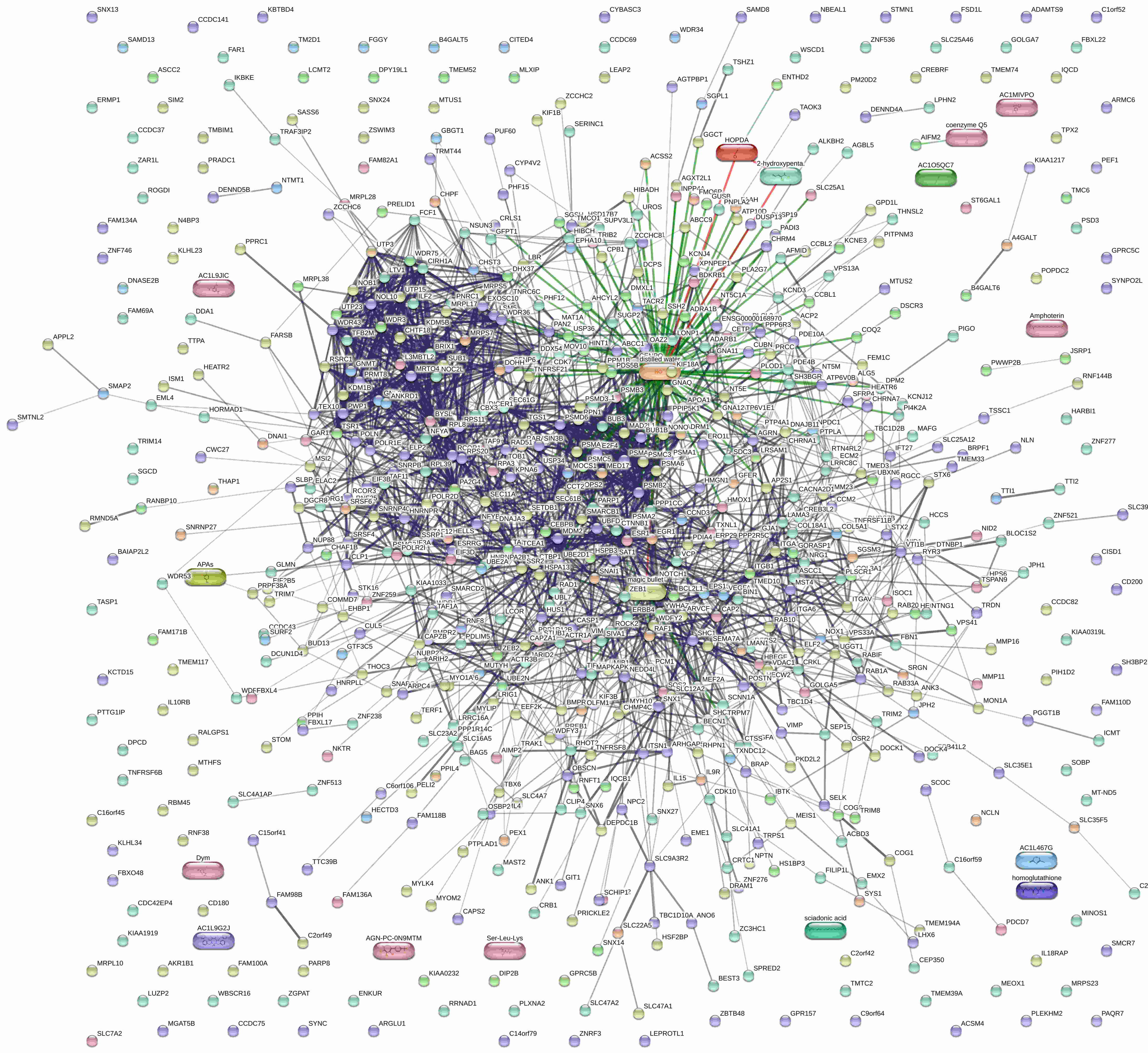

Supplement: Supplementary file 6 — Network analysis figures. All figures were converted to pdf files. (ZIP 47344 kb) [file 12192_2018_954_MOESM6_ESM.zip › Muscle Highland-lowland evening - stitch.pdf]

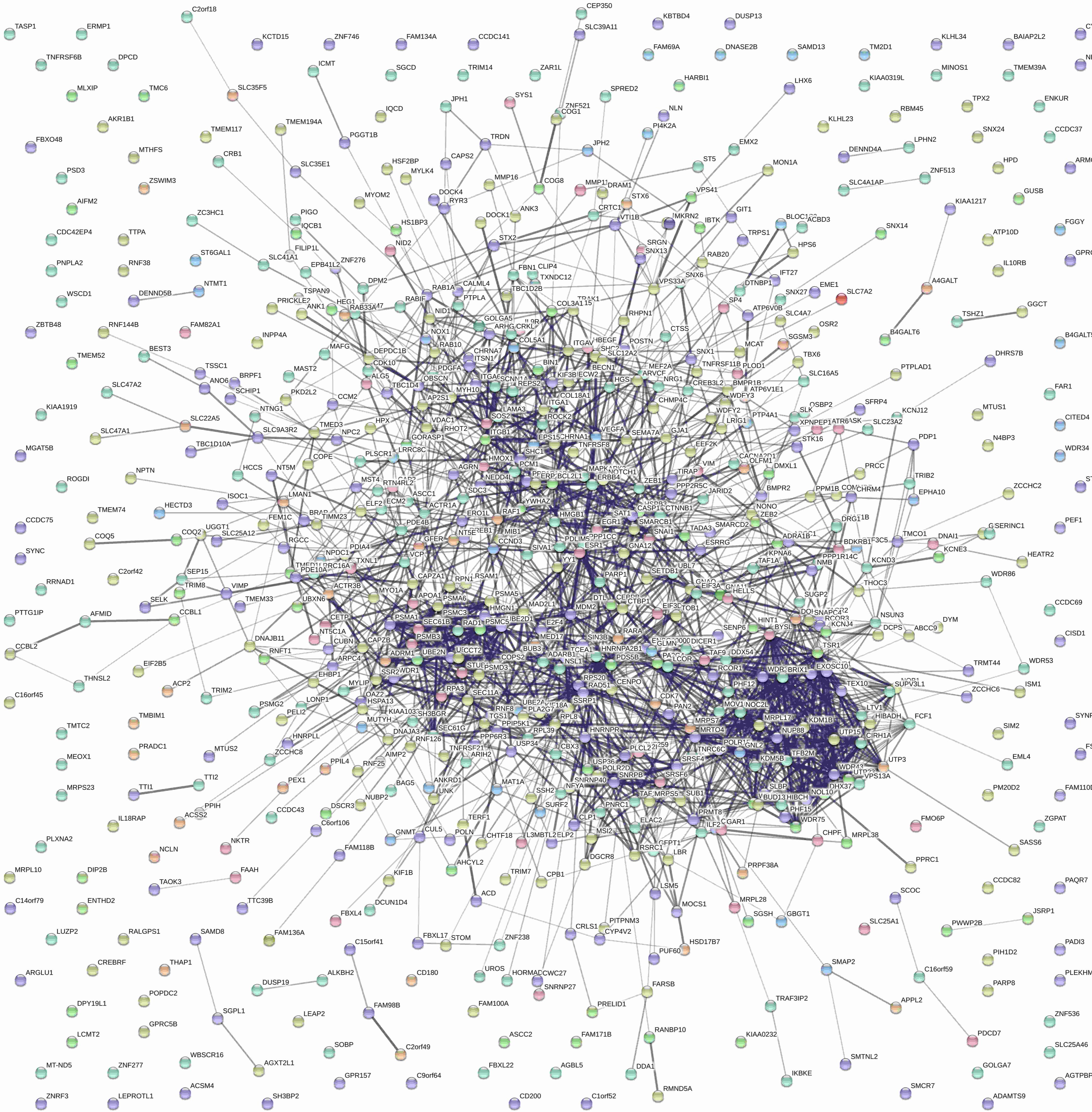

Supplement: Supplementary file 6 — Network analysis figures. All figures were converted to pdf files. (ZIP 47344 kb) [file 12192_2018_954_MOESM6_ESM.zip › Muscle Highland-lowland evening - string.pdf]

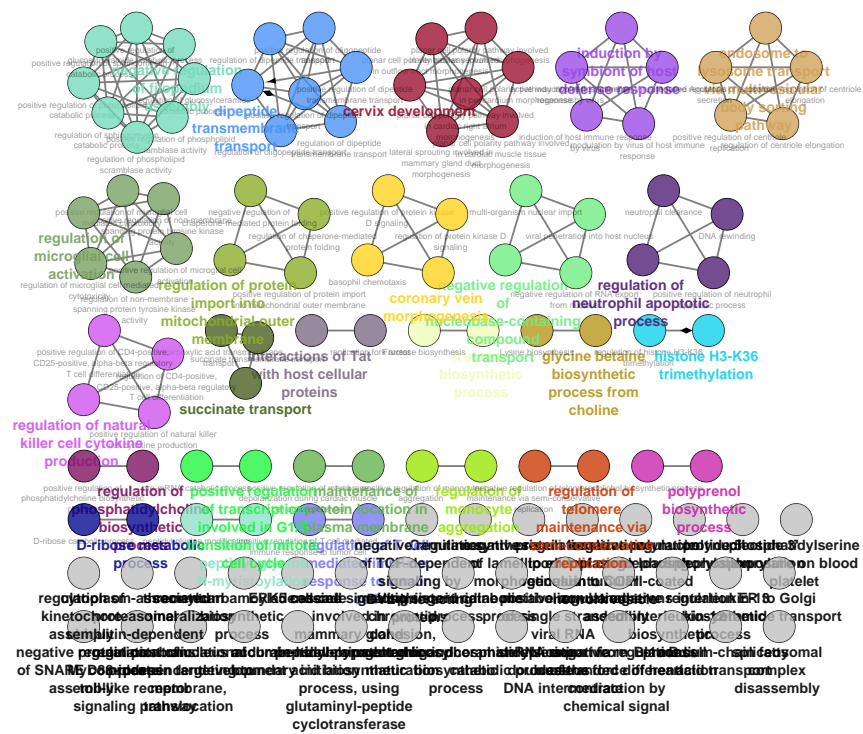

Supplement: Supplementary file 6 — Network analysis figures. All figures were converted to pdf files. (ZIP 47344 kb) [file 12192_2018_954_MOESM6_ESM.zip › Muscle Highland-lowland noon - Cytoscape-ClueGo.pdf]

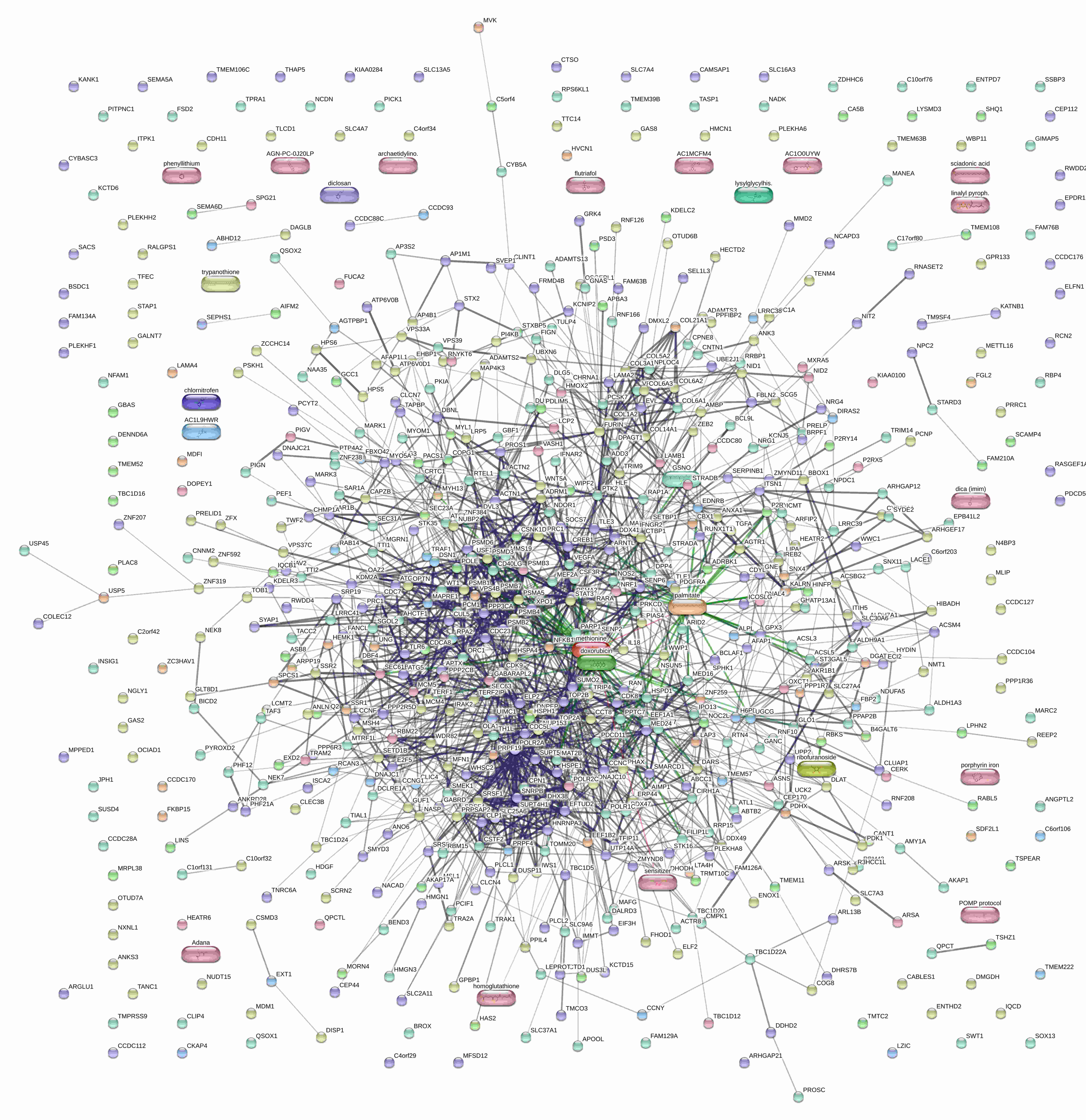

Supplement: Supplementary file 6 — Network analysis figures. All figures were converted to pdf files. (ZIP 47344 kb) [file 12192_2018_954_MOESM6_ESM.zip › Muscle Highland-lowland noon -stitch.pdf]

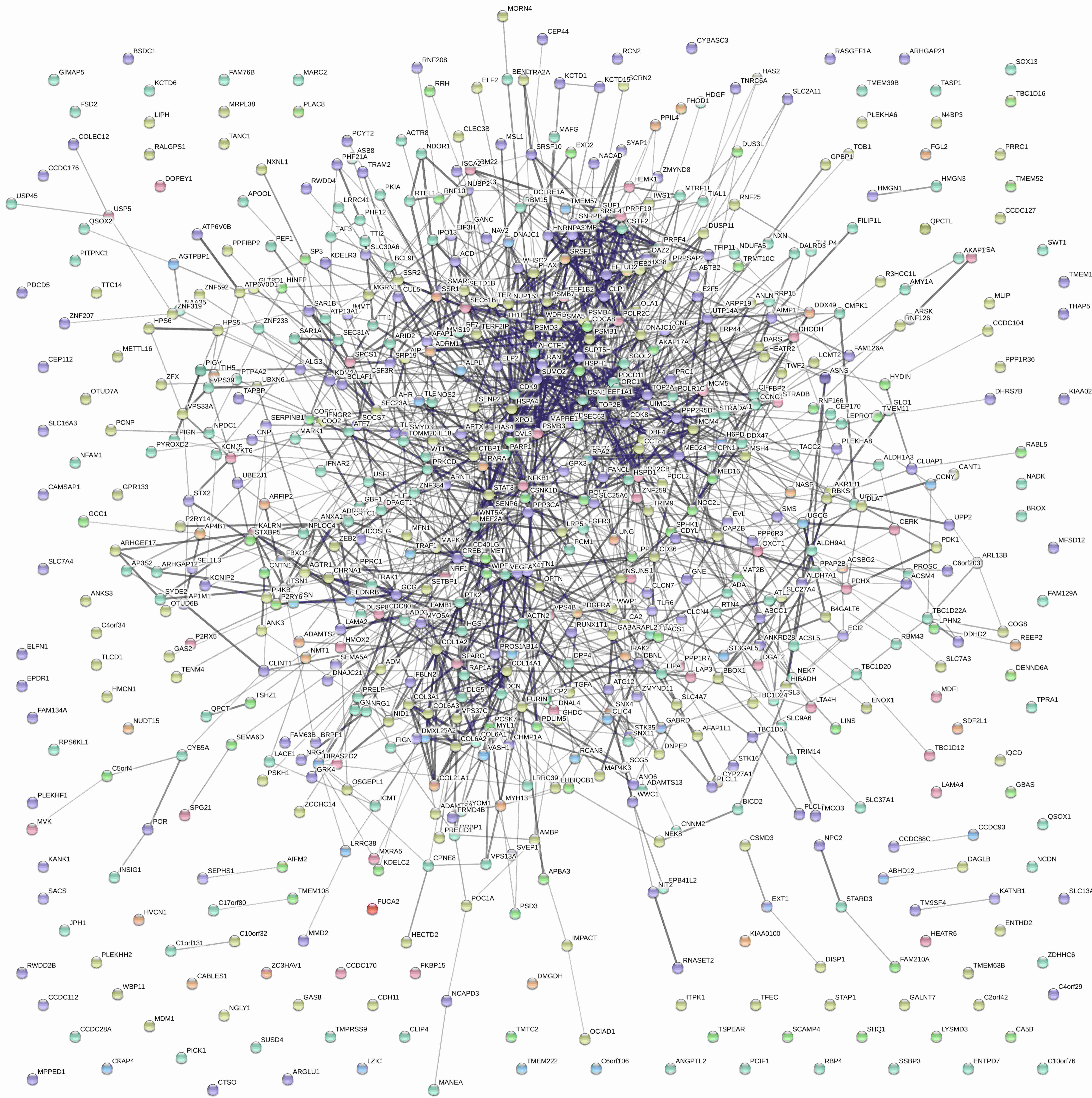

Supplement: Supplementary file 6 — Network analysis figures. All figures were converted to pdf files. (ZIP 47344 kb) [file 12192_2018_954_MOESM6_ESM.zip › Muscle Highland-lowland noon -string.pdf]

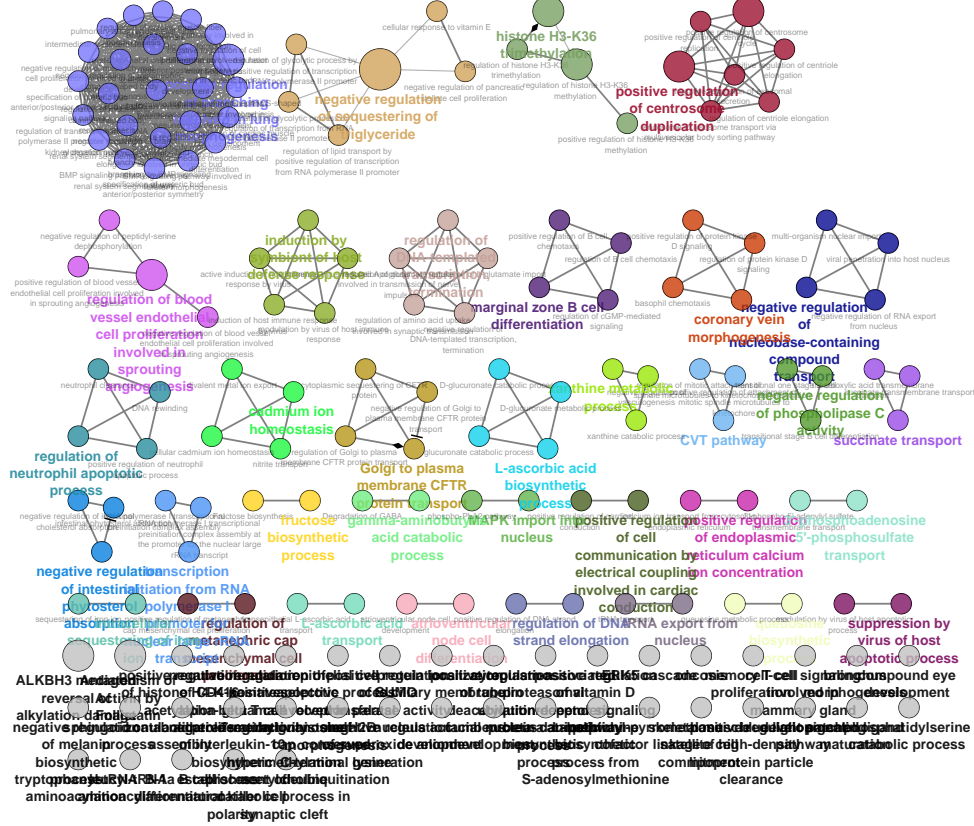

Supplement: Supplementary file 6 — Network analysis figures. All figures were converted to pdf files. (ZIP 47344 kb) [file 12192_2018_954_MOESM6_ESM.zip › Muscle Lowland all -Cytoscape-ClueGo.pdf]

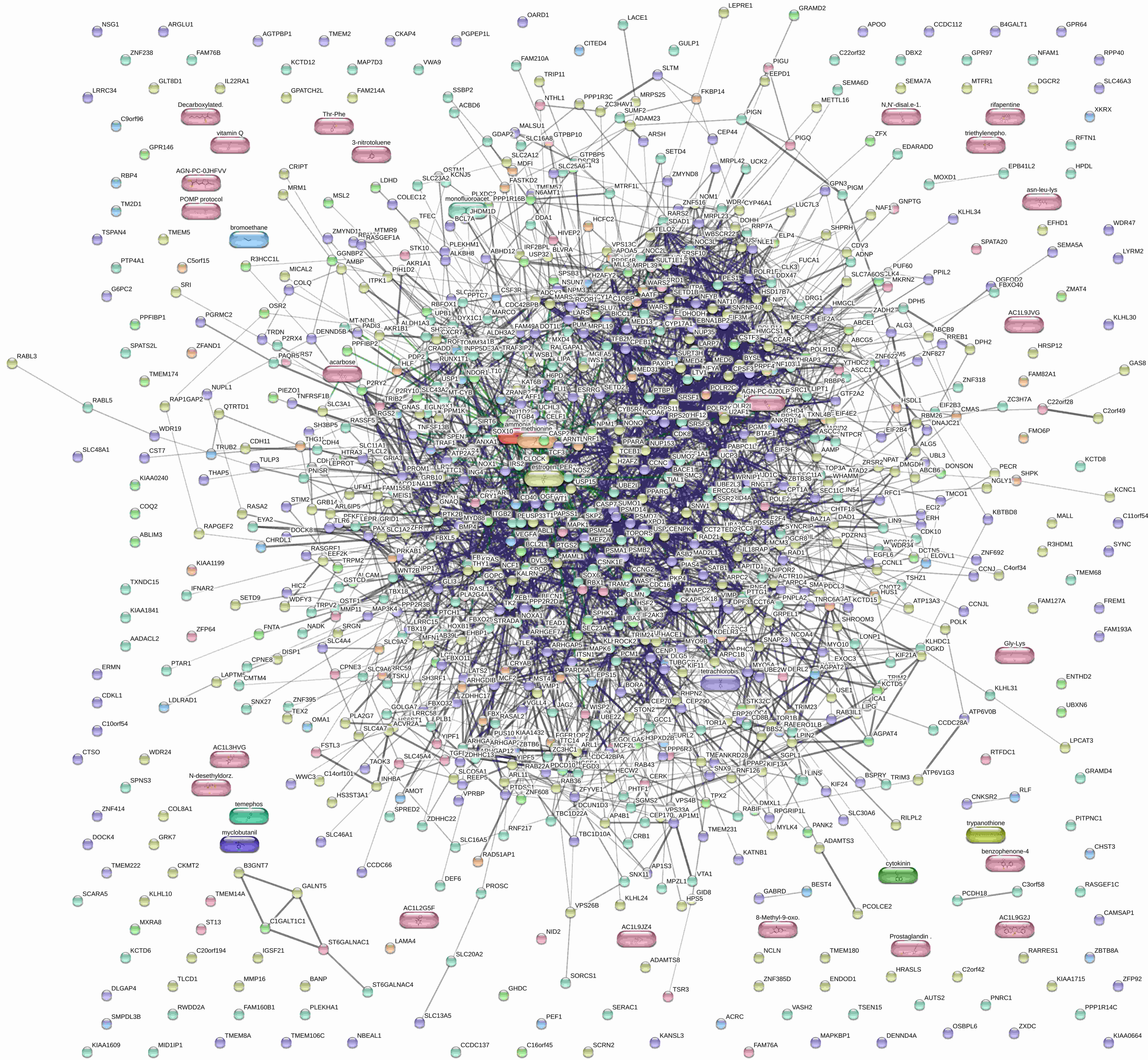

Supplement: Supplementary file 6 — Network analysis figures. All figures were converted to pdf files. (ZIP 47344 kb) [file 12192_2018_954_MOESM6_ESM.zip › Muscle Lowland all -stitch.pdf]

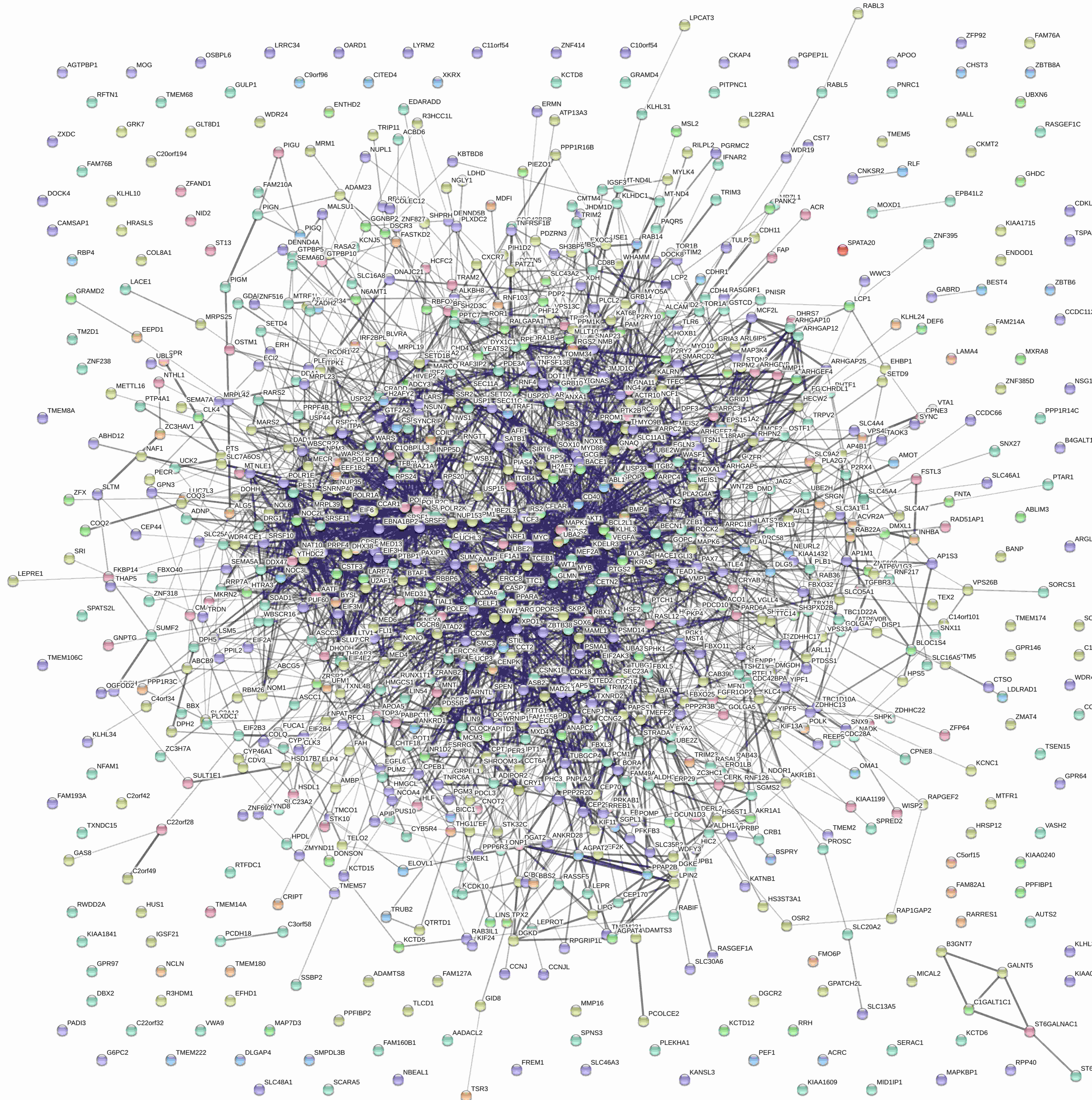

Supplement: Supplementary file 6 — Network analysis figures. All figures were converted to pdf files. (ZIP 47344 kb) [file 12192_2018_954_MOESM6_ESM.zip › Muscle Lowland all -string.pdf]

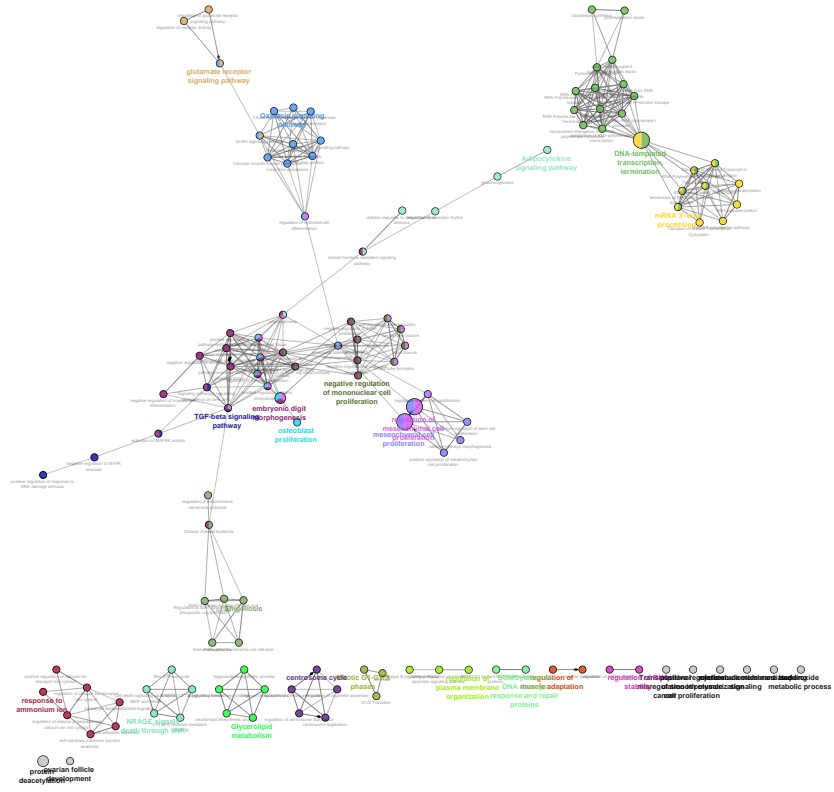

Supplement: Supplementary file 6 — Network analysis figures. All figures were converted to pdf files. (ZIP 47344 kb) [file 12192_2018_954_MOESM6_ESM.zip › Muscle Lowland morning-evening - Cytoscape-ClueGo.pdf]

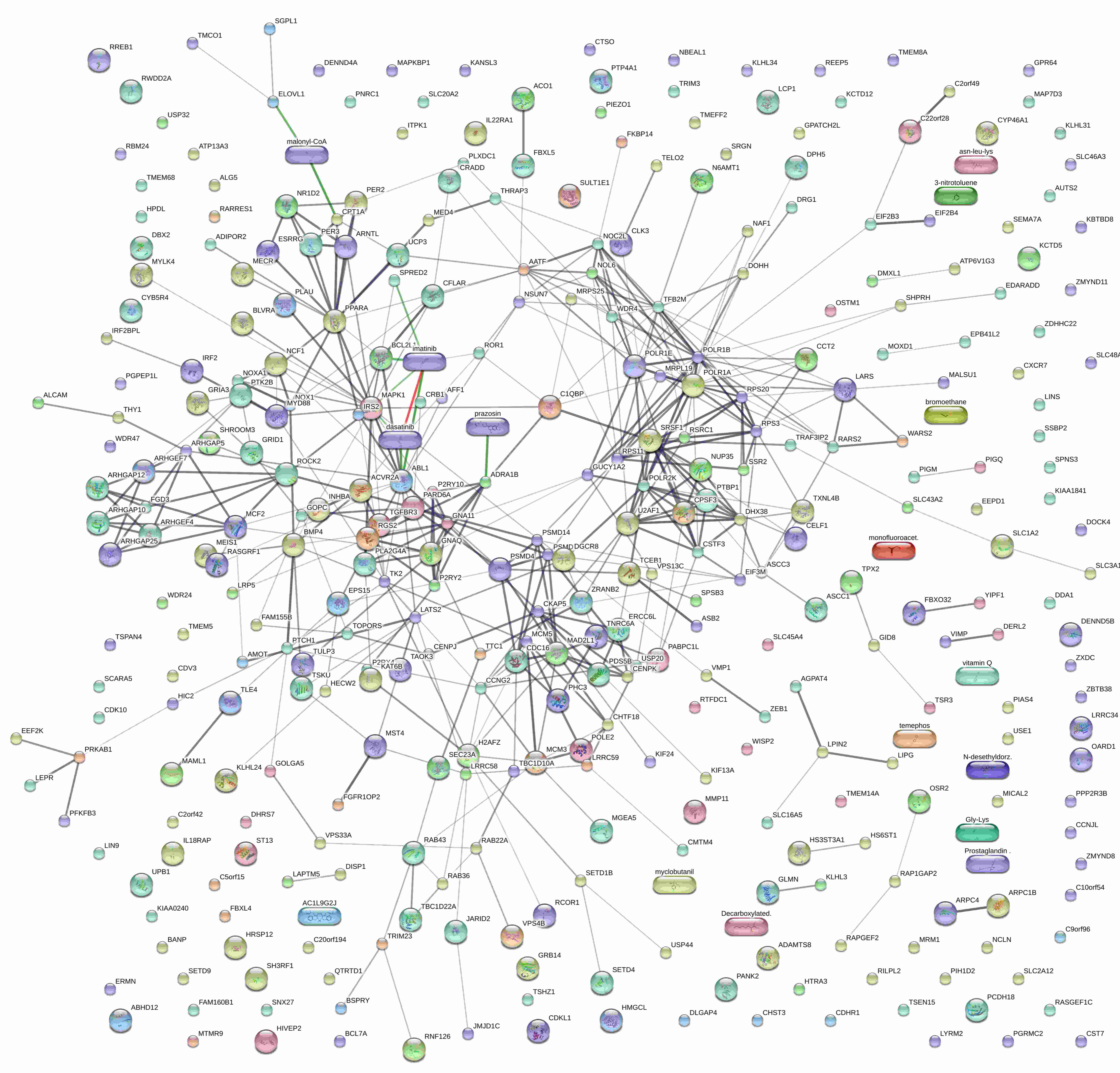

Supplement: Supplementary file 6 — Network analysis figures. All figures were converted to pdf files. (ZIP 47344 kb) [file 12192_2018_954_MOESM6_ESM.zip › Muscle Lowland morning-evening -stitch.pdf]

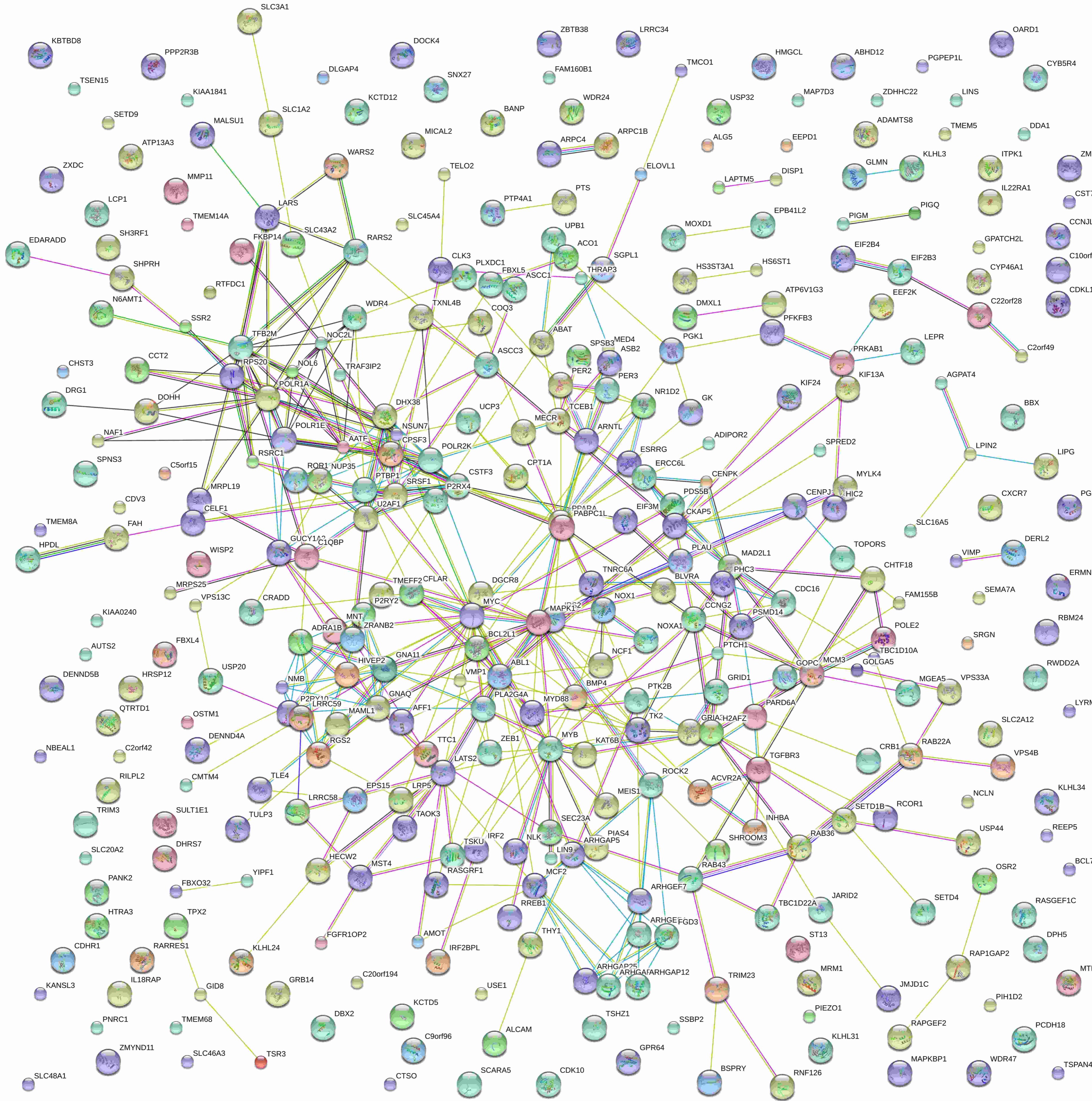

Supplement: Supplementary file 6 — Network analysis figures. All figures were converted to pdf files. (ZIP 47344 kb) [file 12192_2018_954_MOESM6_ESM.zip › Muscle Lowland morning-evening -string.pdf]

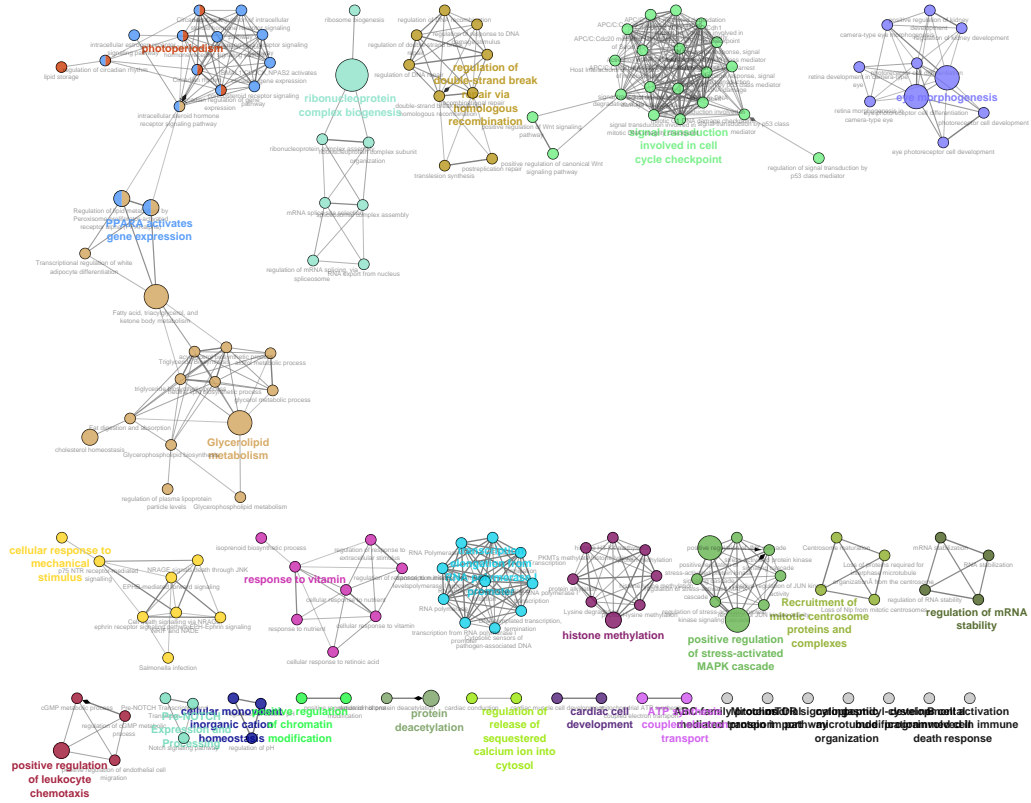

Supplement: Supplementary file 6 — Network analysis figures. All figures were converted to pdf files. (ZIP 47344 kb) [file 12192_2018_954_MOESM6_ESM.zip › Muscle Lowland morning-noon -Cytoscape-ClueGo.pdf]

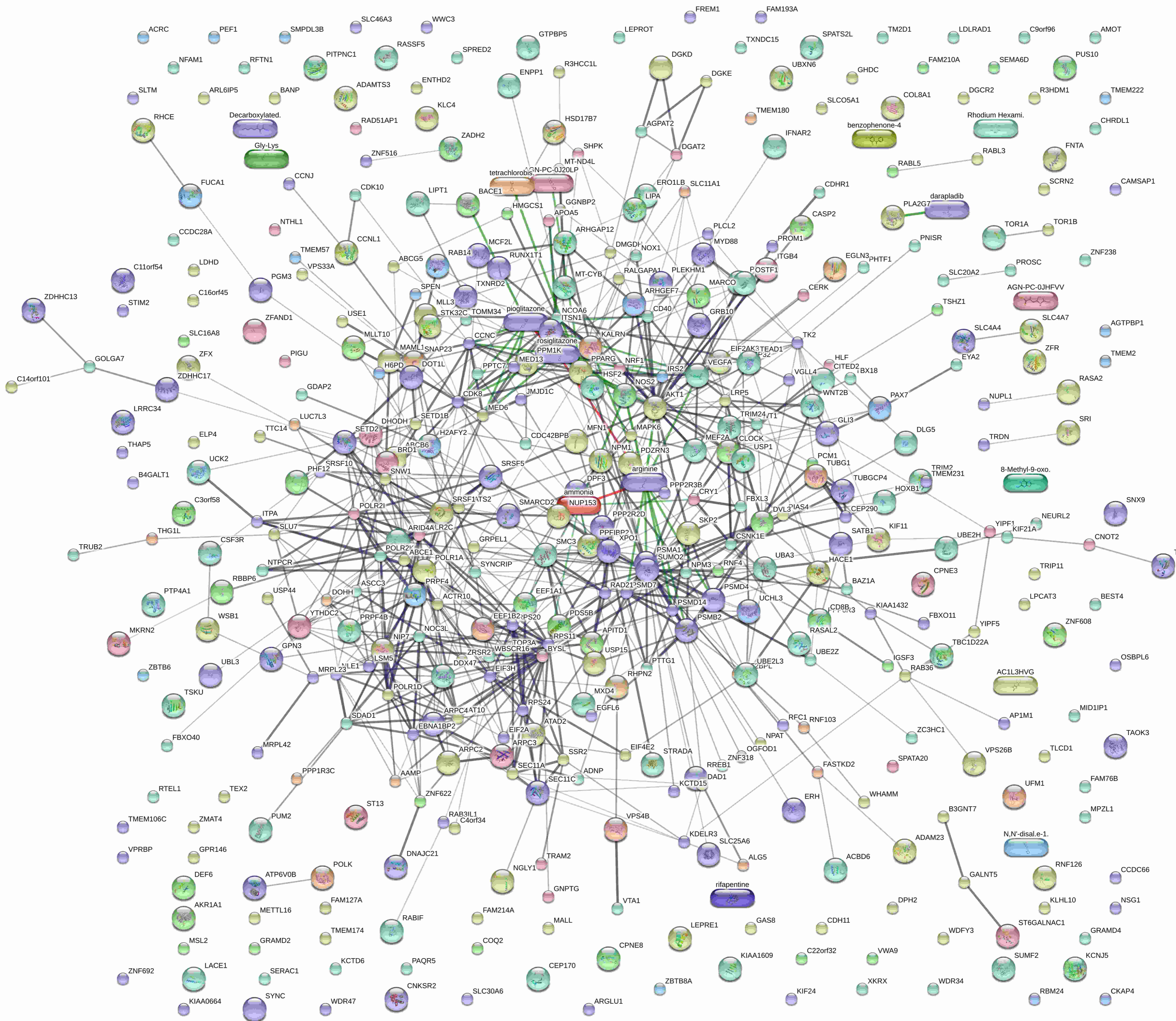

Supplement: Supplementary file 6 — Network analysis figures. All figures were converted to pdf files. (ZIP 47344 kb) [file 12192_2018_954_MOESM6_ESM.zip › Muscle Lowland morning-noon -stitch.pdf]

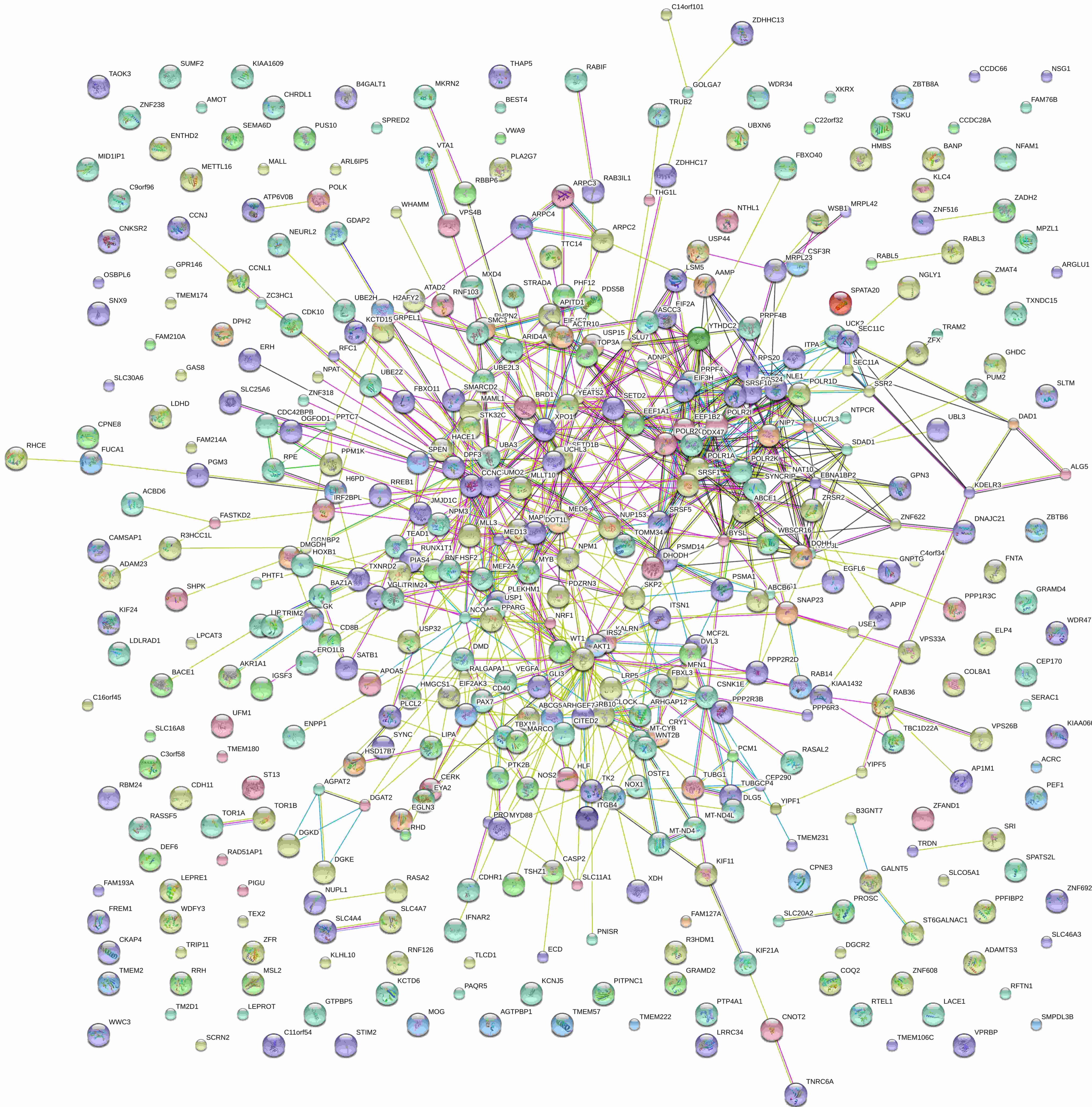

Supplement: Supplementary file 6 — Network analysis figures. All figures were converted to pdf files. (ZIP 47344 kb) [file 12192_2018_954_MOESM6_ESM.zip › Muscle Lowland morning-noon -string.pdf]

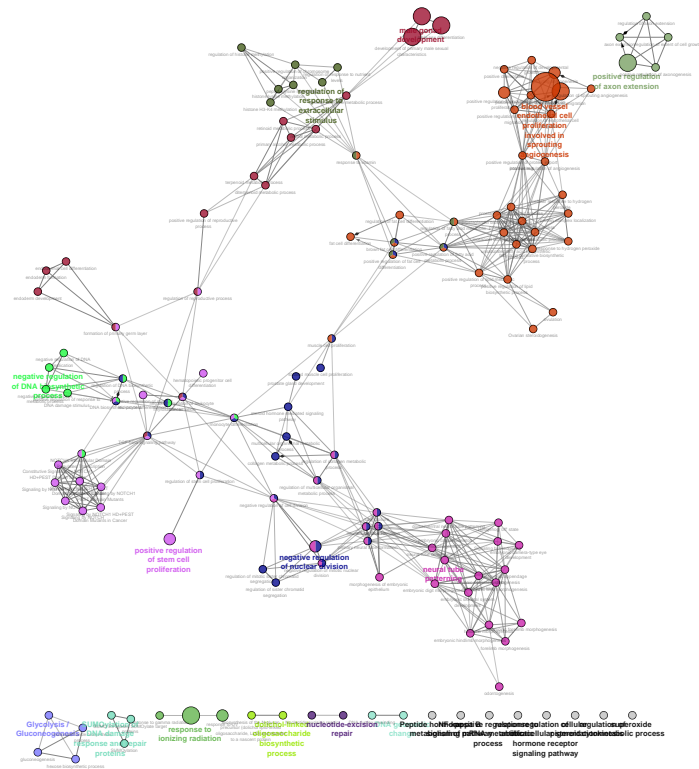

Supplement: Supplementary file 6 — Network analysis figures. All figures were converted to pdf files. (ZIP 47344 kb) [file 12192_2018_954_MOESM6_ESM.zip › Muscle Lowland noon-evening -Cytoscape-ClueGo.pdf]

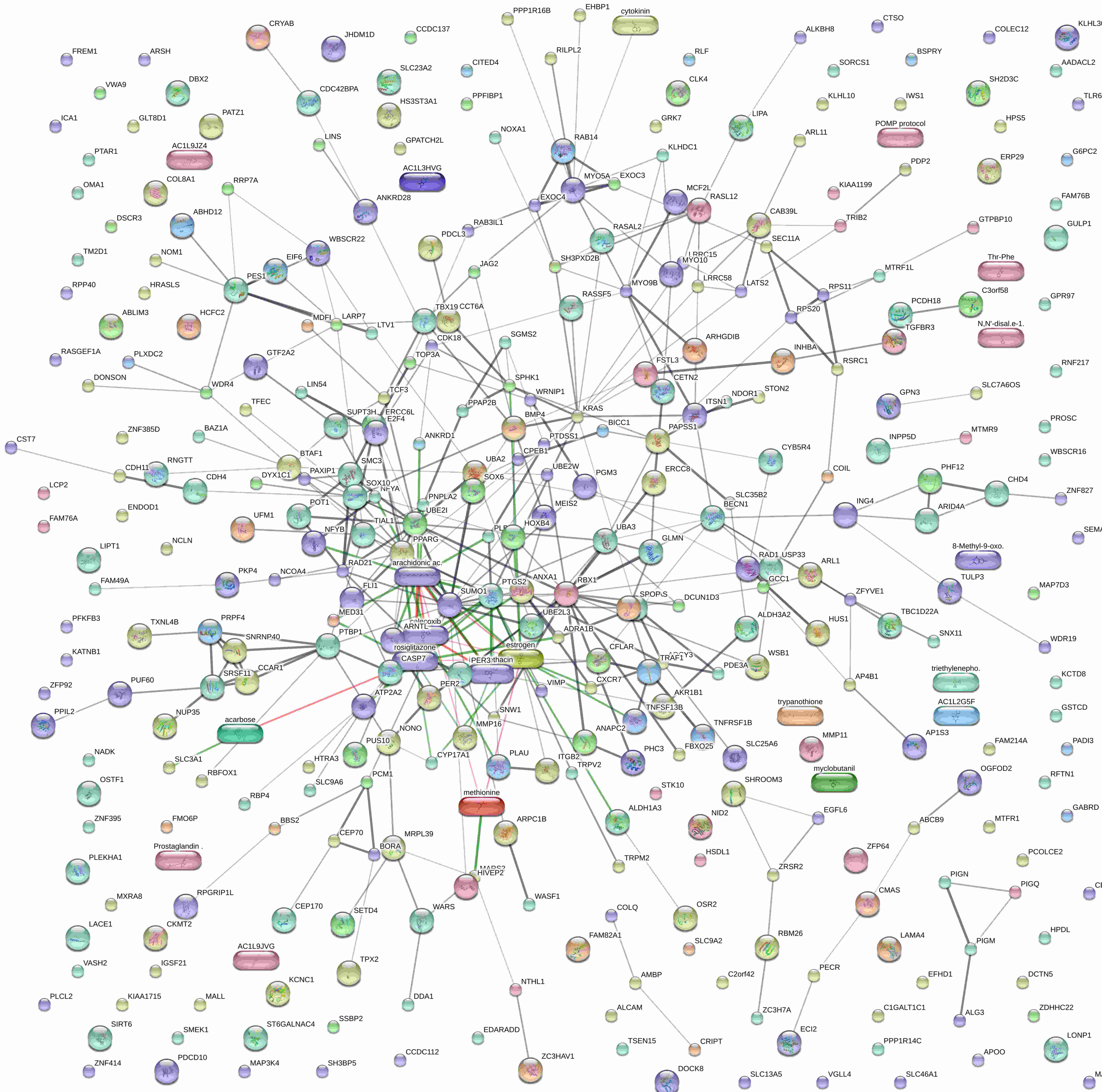

Supplement: Supplementary file 6 — Network analysis figures. All figures were converted to pdf files. (ZIP 47344 kb) [file 12192_2018_954_MOESM6_ESM.zip › Muscle Lowland noon-evening -stitch.pdf]

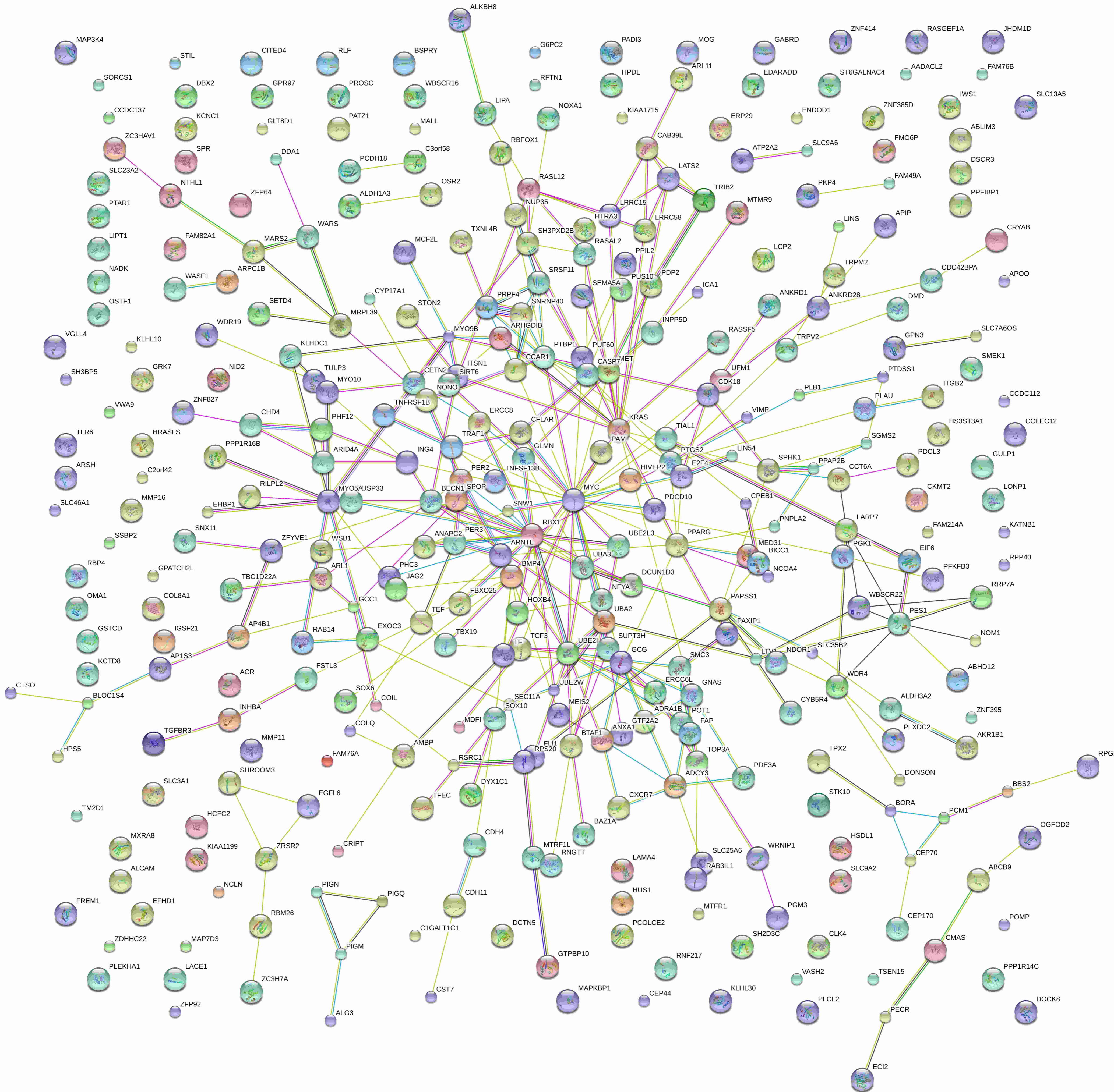

Supplement: Supplementary file 6 — Network analysis figures. All figures were converted to pdf files. (ZIP 47344 kb) [file 12192_2018_954_MOESM6_ESM.zip › Muscle Lowland noon-evening -string.pdf]

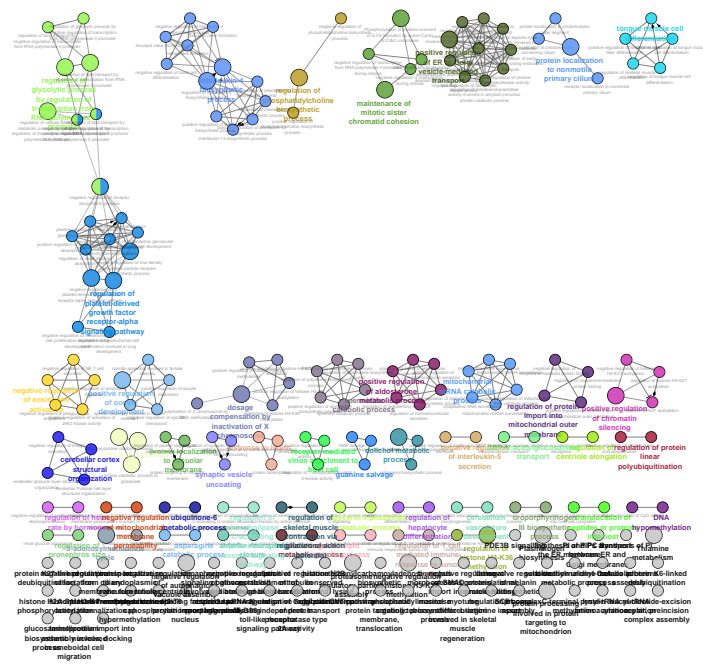

Supplement: Supplementary file 6 — Network analysis figures. All figures were converted to pdf files. (ZIP 47344 kb) [file 12192_2018_954_MOESM6_ESM.zip › Spleen Highland all - Cytoscape-ClueGo.pdf]

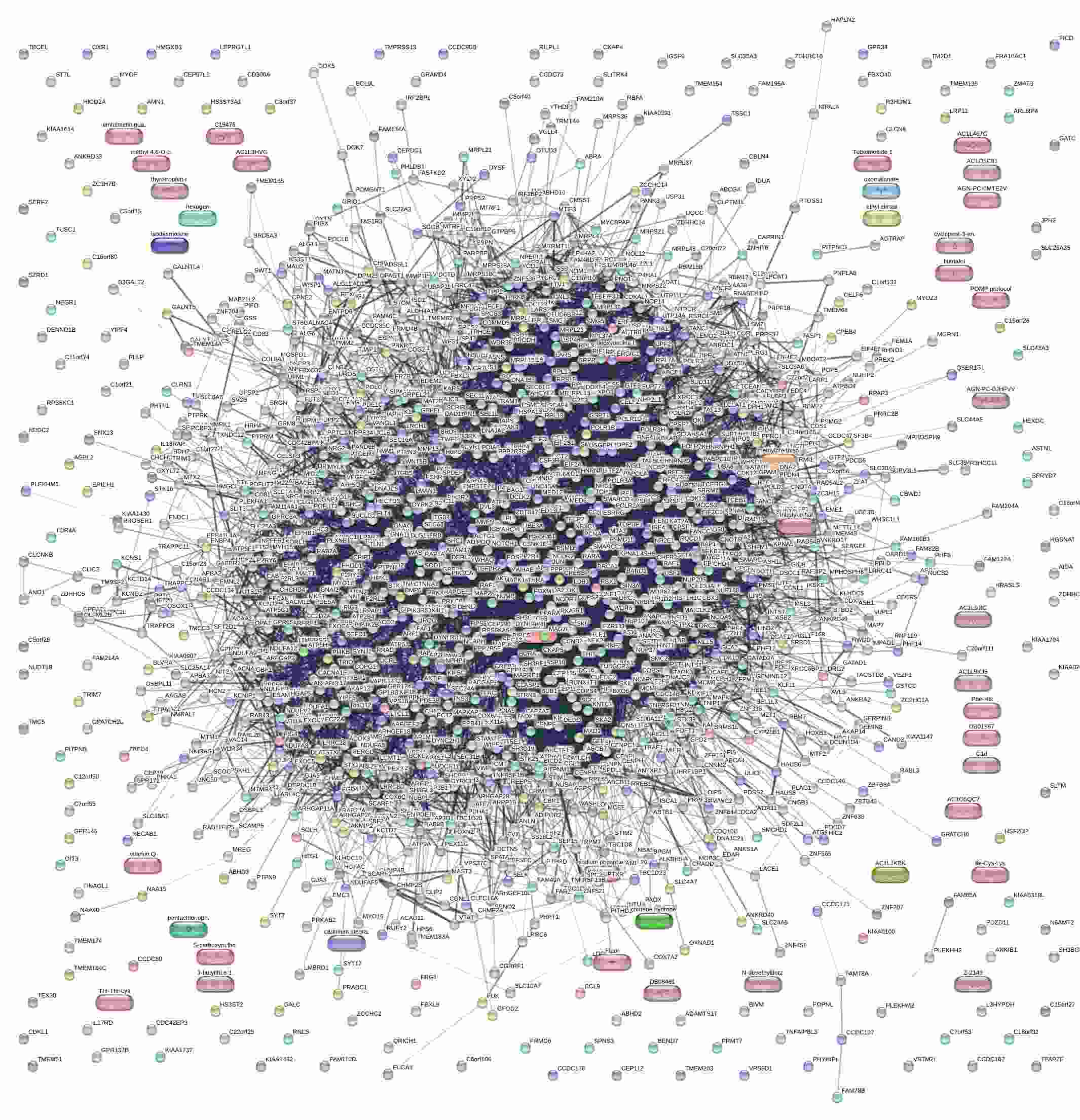

Supplement: Supplementary file 6 — Network analysis figures. All figures were converted to pdf files. (ZIP 47344 kb) [file 12192_2018_954_MOESM6_ESM.zip › Spleen Highland all - stitch.pdf]

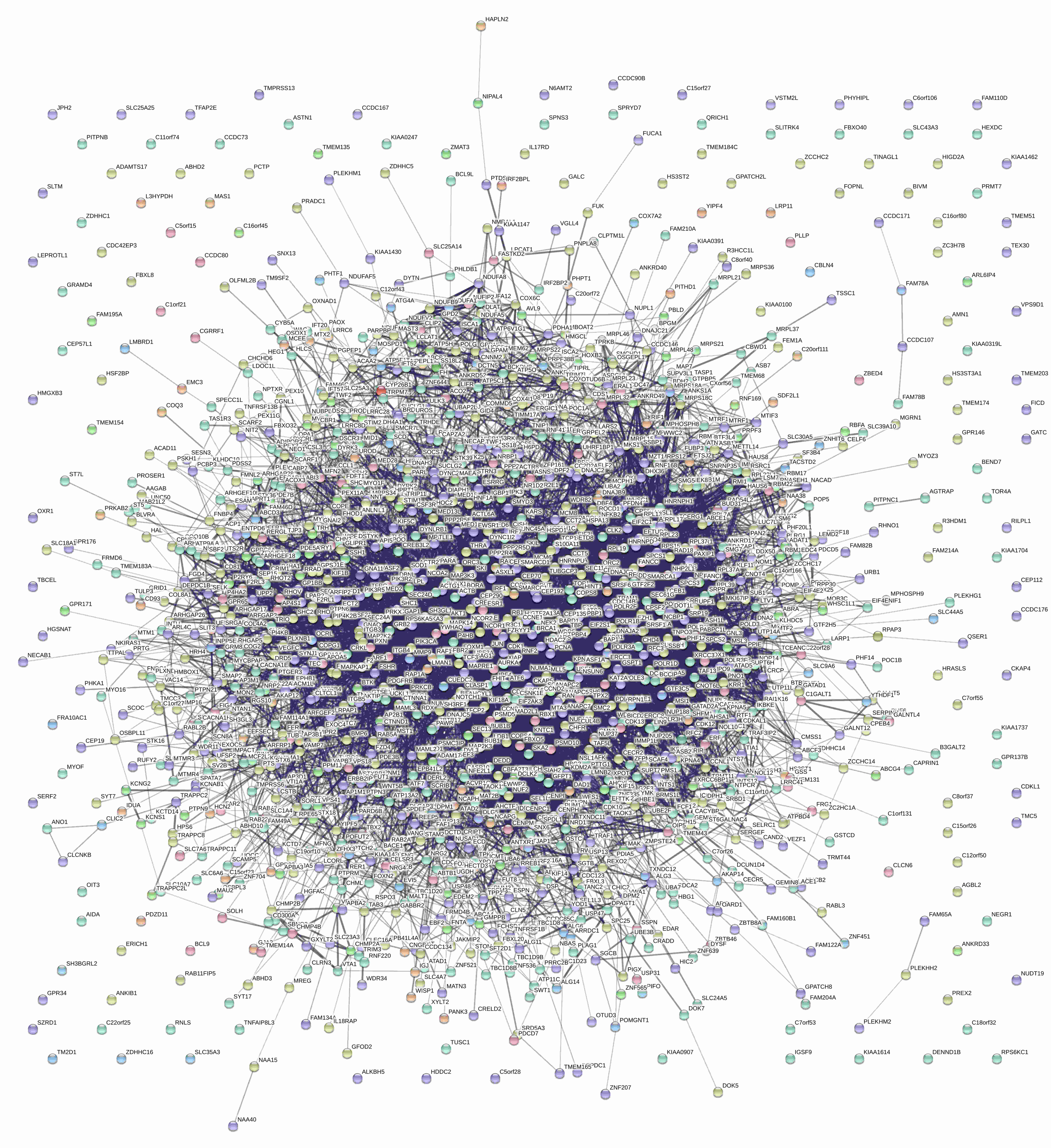

Supplement: Supplementary file 6 — Network analysis figures. All figures were converted to pdf files. (ZIP 47344 kb) [file 12192_2018_954_MOESM6_ESM.zip › Spleen Highland all - string.pdf]

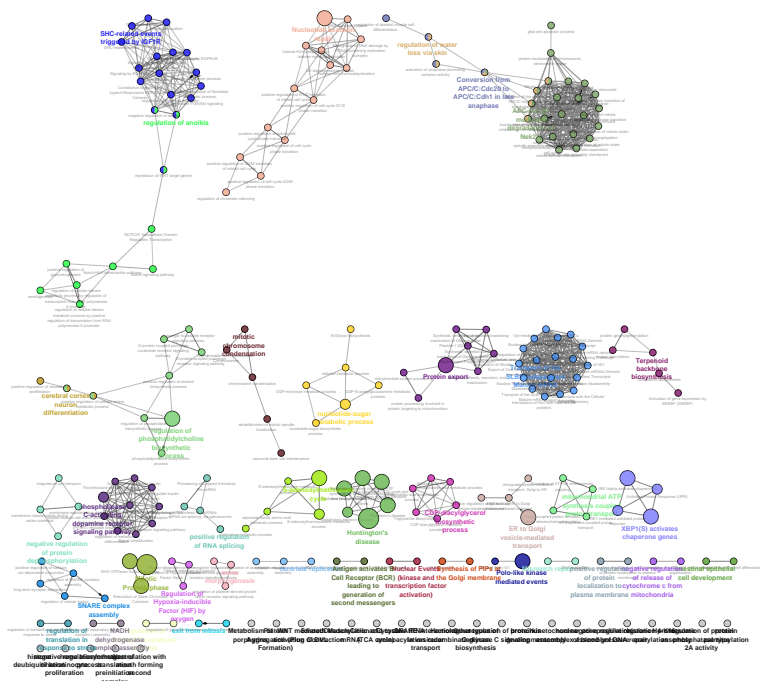

Supplement: Supplementary file 6 — Network analysis figures. All figures were converted to pdf files. (ZIP 47344 kb) [file 12192_2018_954_MOESM6_ESM.zip › Spleen Highland morning-evening - Cytoscape-ClueGo.pdf]

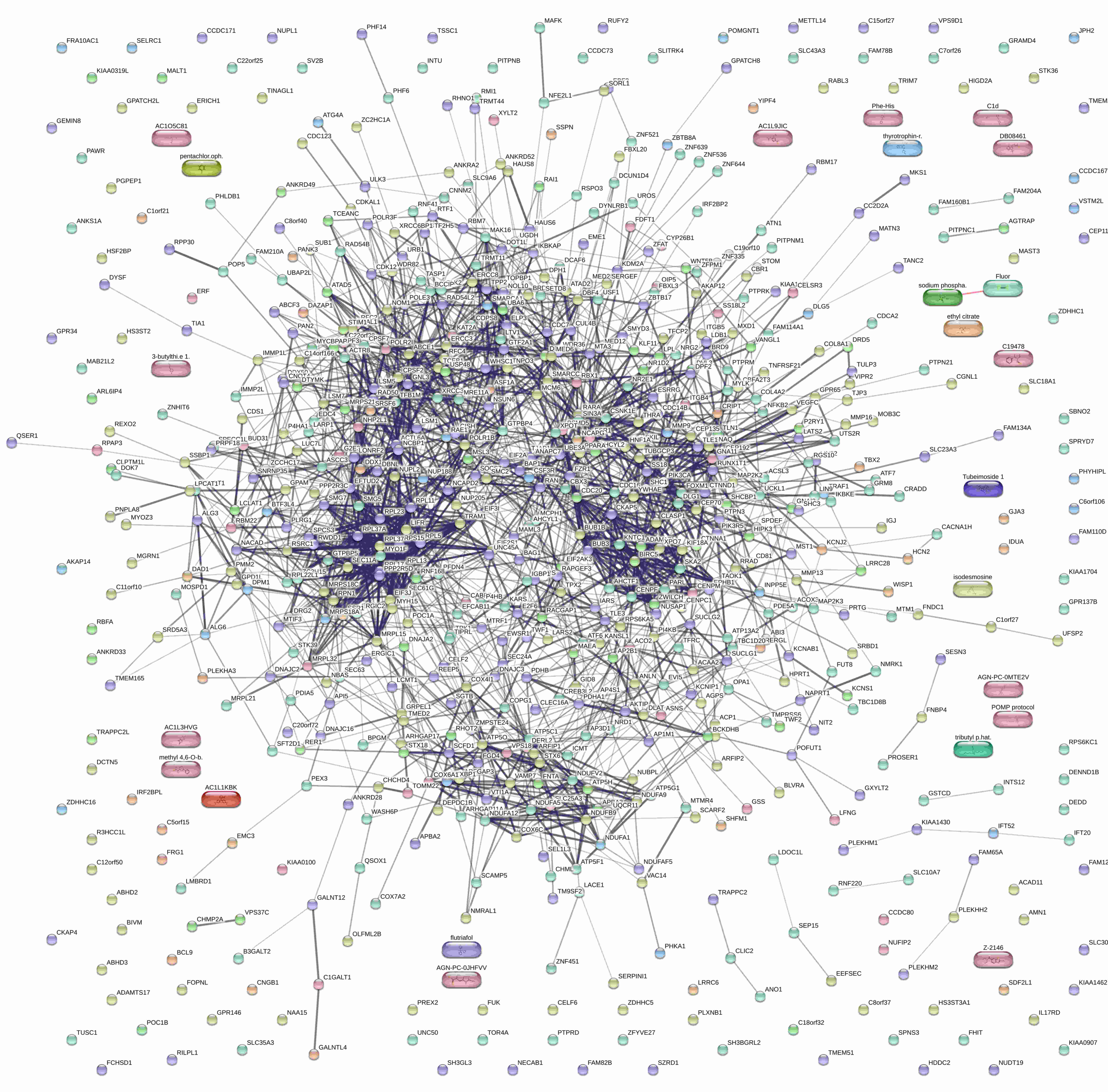

Supplement: Supplementary file 6 — Network analysis figures. All figures were converted to pdf files. (ZIP 47344 kb) [file 12192_2018_954_MOESM6_ESM.zip › Spleen Highland morning-evening - stitch.pdf]

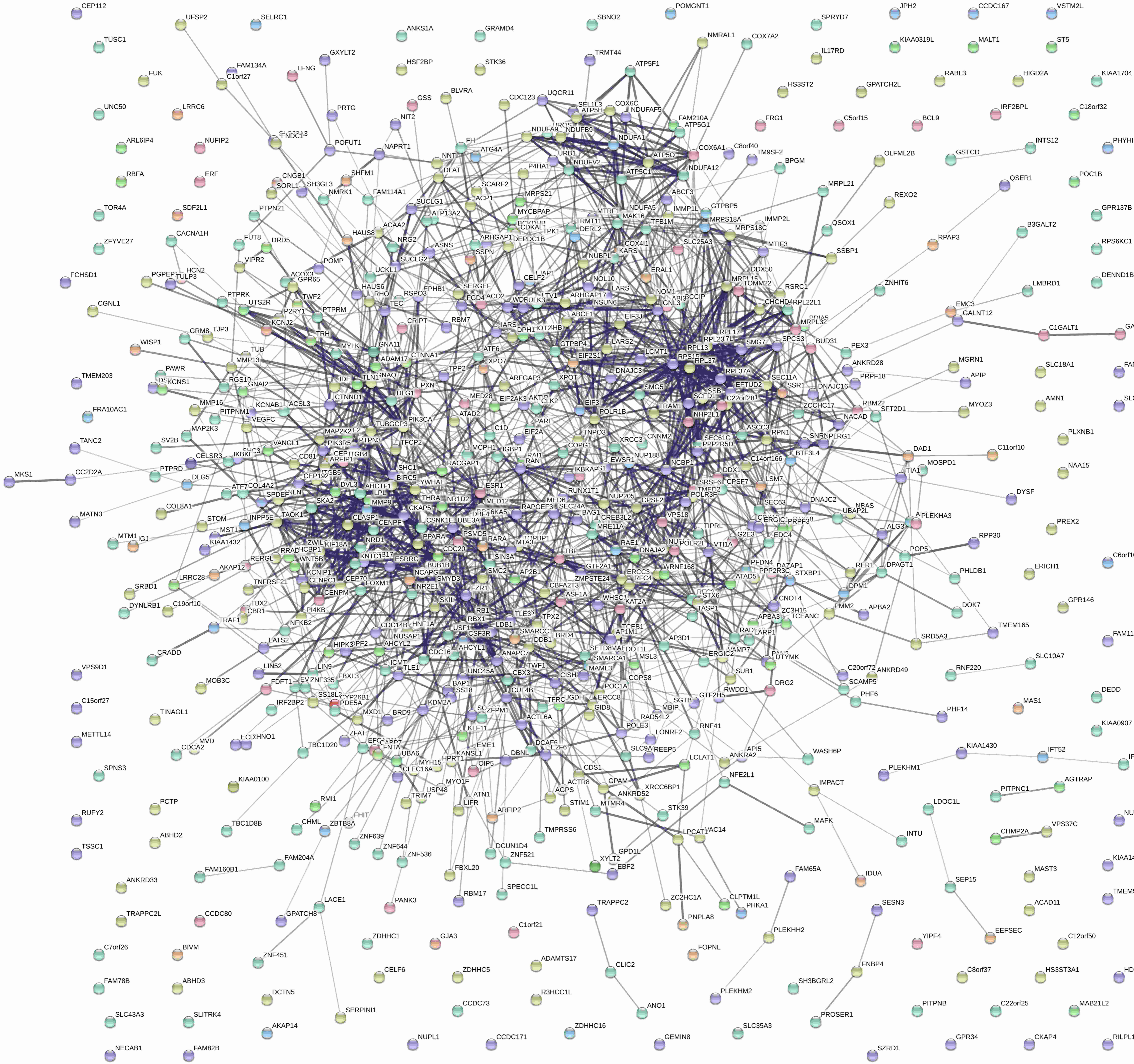

Supplement: Supplementary file 6 — Network analysis figures. All figures were converted to pdf files. (ZIP 47344 kb) [file 12192_2018_954_MOESM6_ESM.zip › Spleen Highland morning-evening - string.pdf]

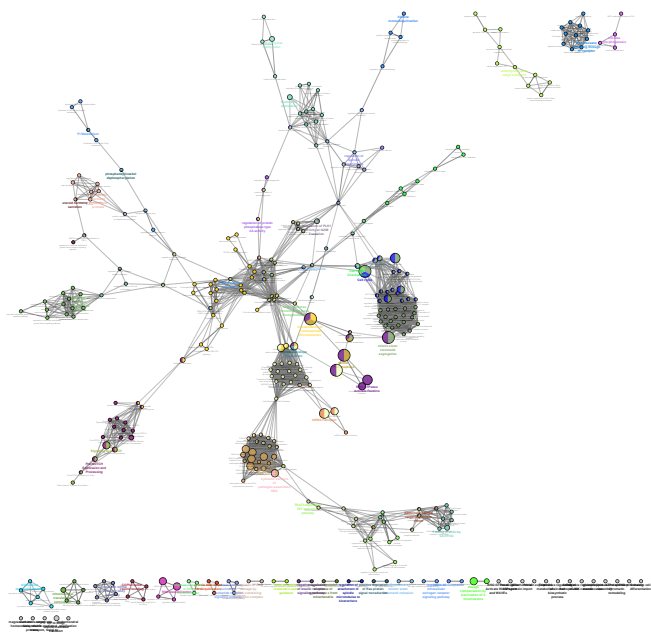

Supplement: Supplementary file 6 — Network analysis figures. All figures were converted to pdf files. (ZIP 47344 kb) [file 12192_2018_954_MOESM6_ESM.zip › Spleen Highland morning-noon - Cytoscape-ClueGo.pdf]

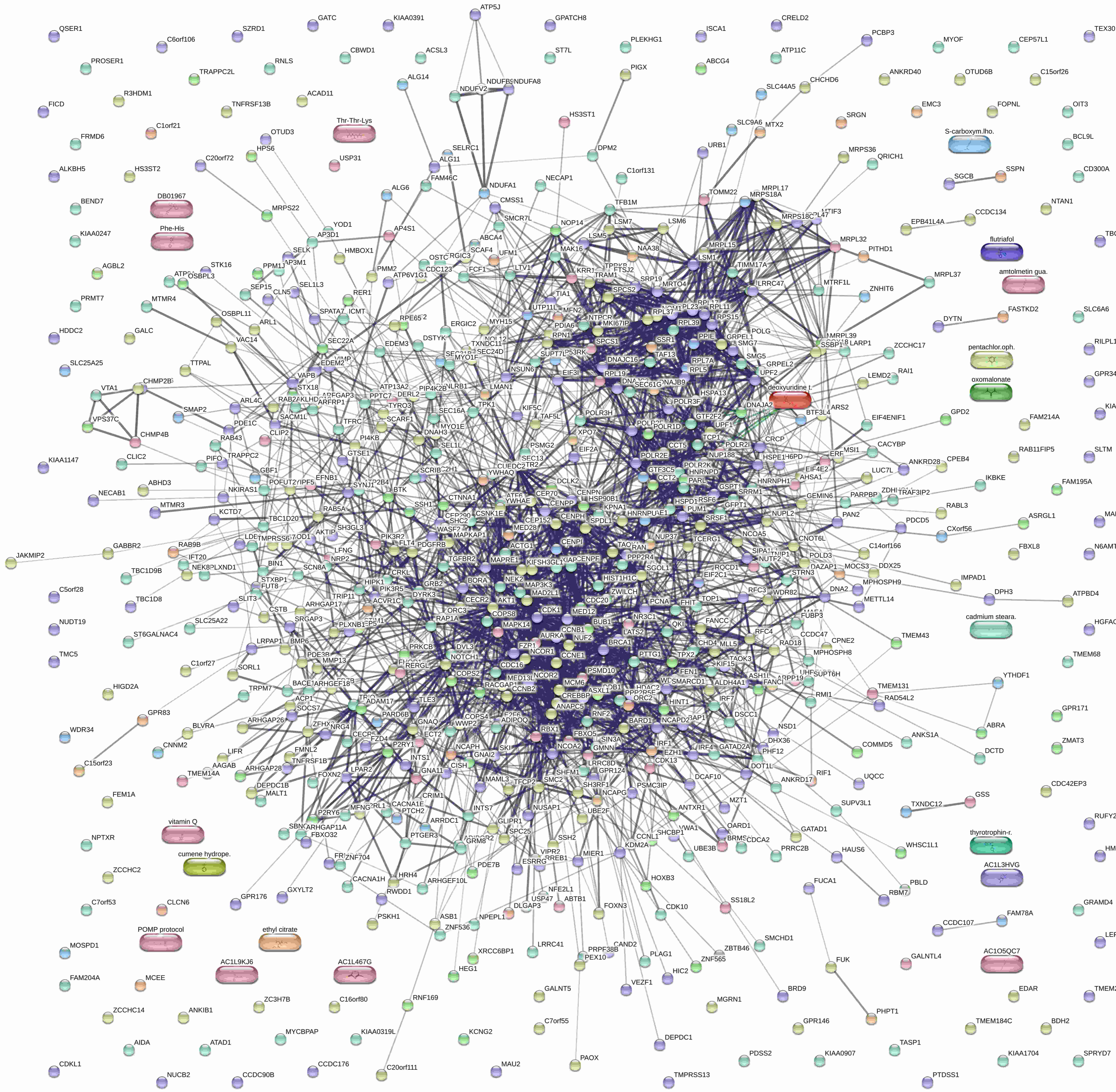

Supplement: Supplementary file 6 — Network analysis figures. All figures were converted to pdf files. (ZIP 47344 kb) [file 12192_2018_954_MOESM6_ESM.zip › Spleen Highland morning-noon - stitch.pdf]

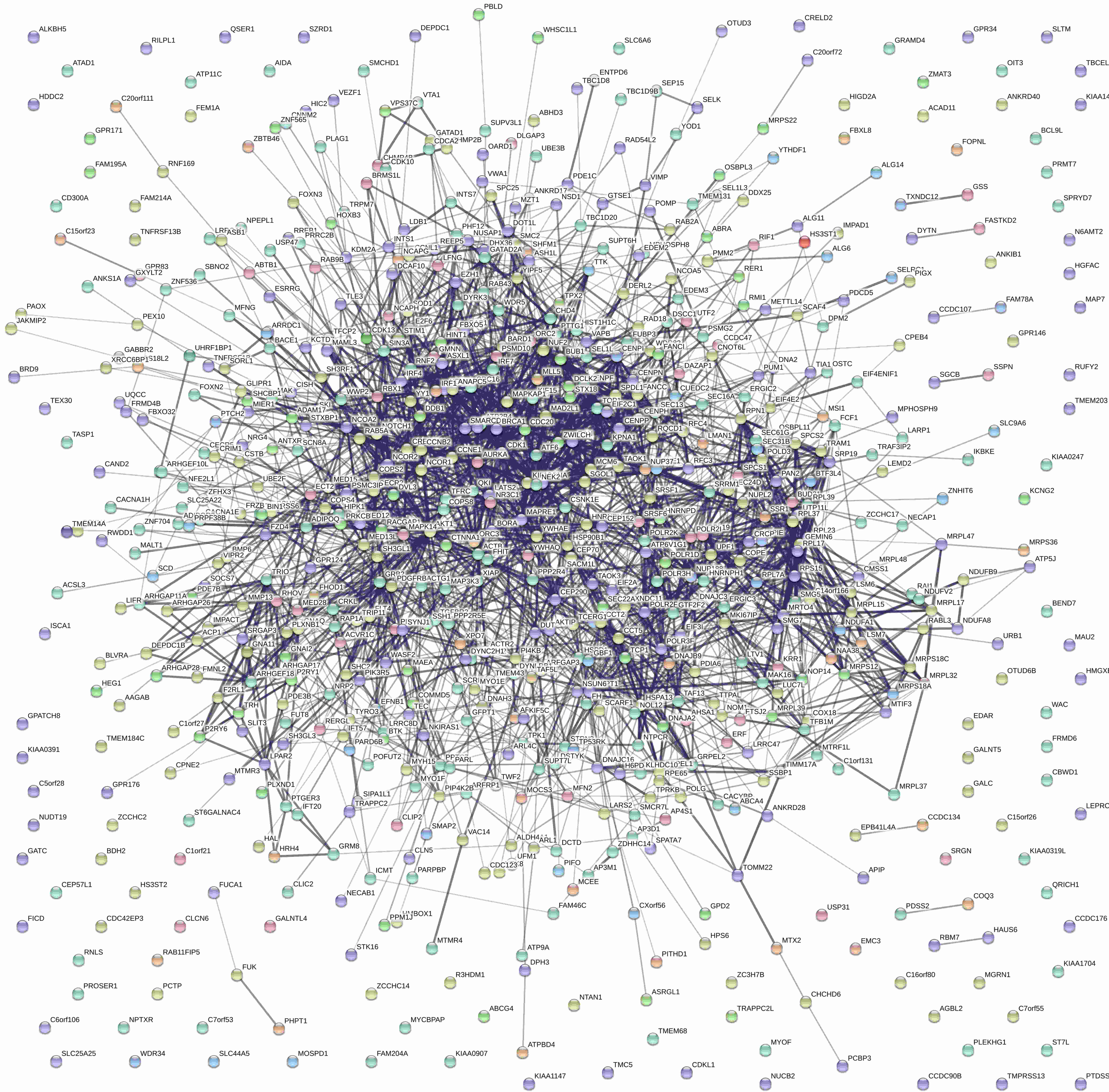

Supplement: Supplementary file 6 — Network analysis figures. All figures were converted to pdf files. (ZIP 47344 kb) [file 12192_2018_954_MOESM6_ESM.zip › Spleen Highland morning-noon - string.pdf]

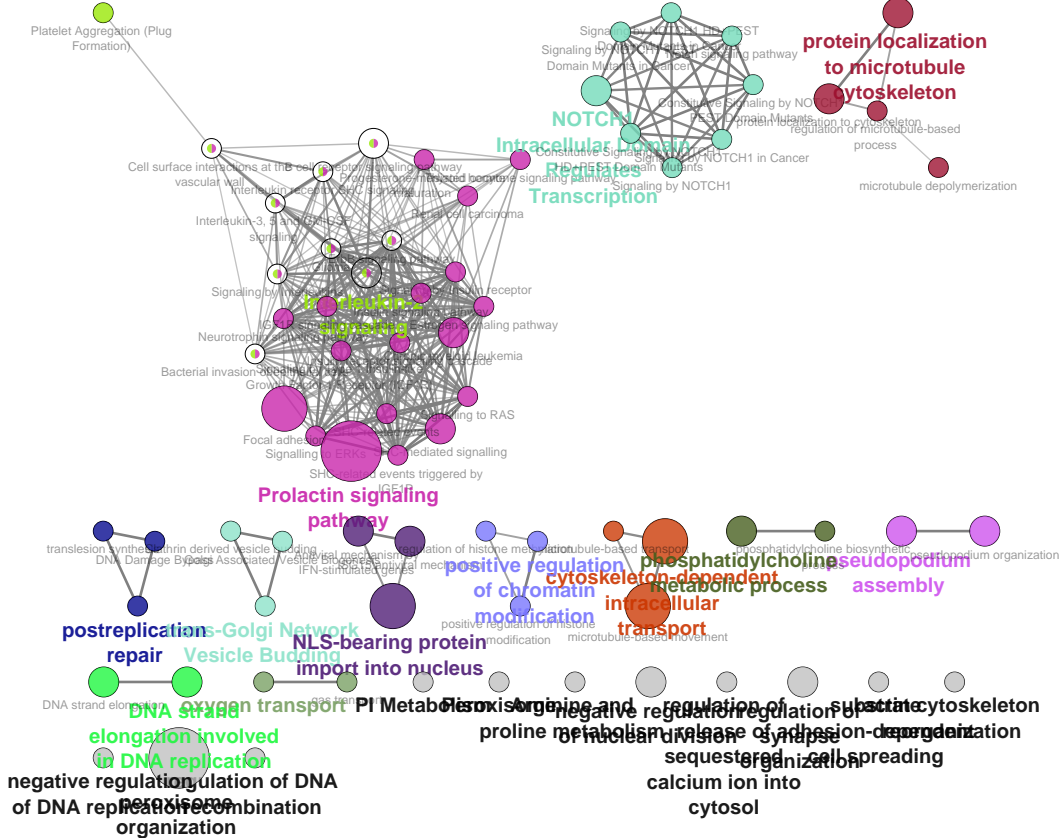

Supplement: Supplementary file 6 — Network analysis figures. All figures were converted to pdf files. (ZIP 47344 kb) [file 12192_2018_954_MOESM6_ESM.zip › Spleen Highland noon-evening - Cytoscape-ClueGo.pdf]

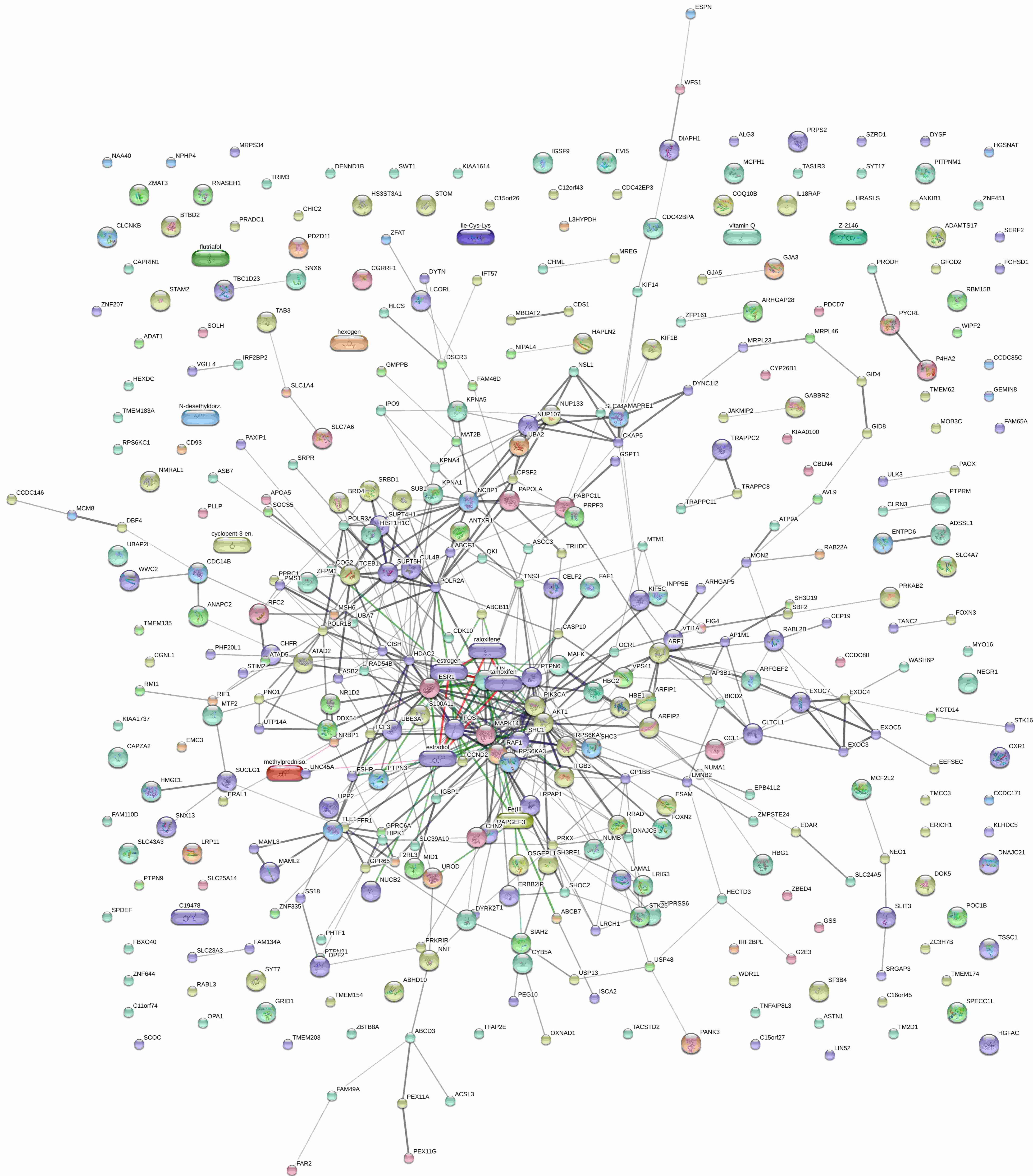

Supplement: Supplementary file 6 — Network analysis figures. All figures were converted to pdf files. (ZIP 47344 kb) [file 12192_2018_954_MOESM6_ESM.zip › Spleen Highland noon-evening - stitch.pdf]

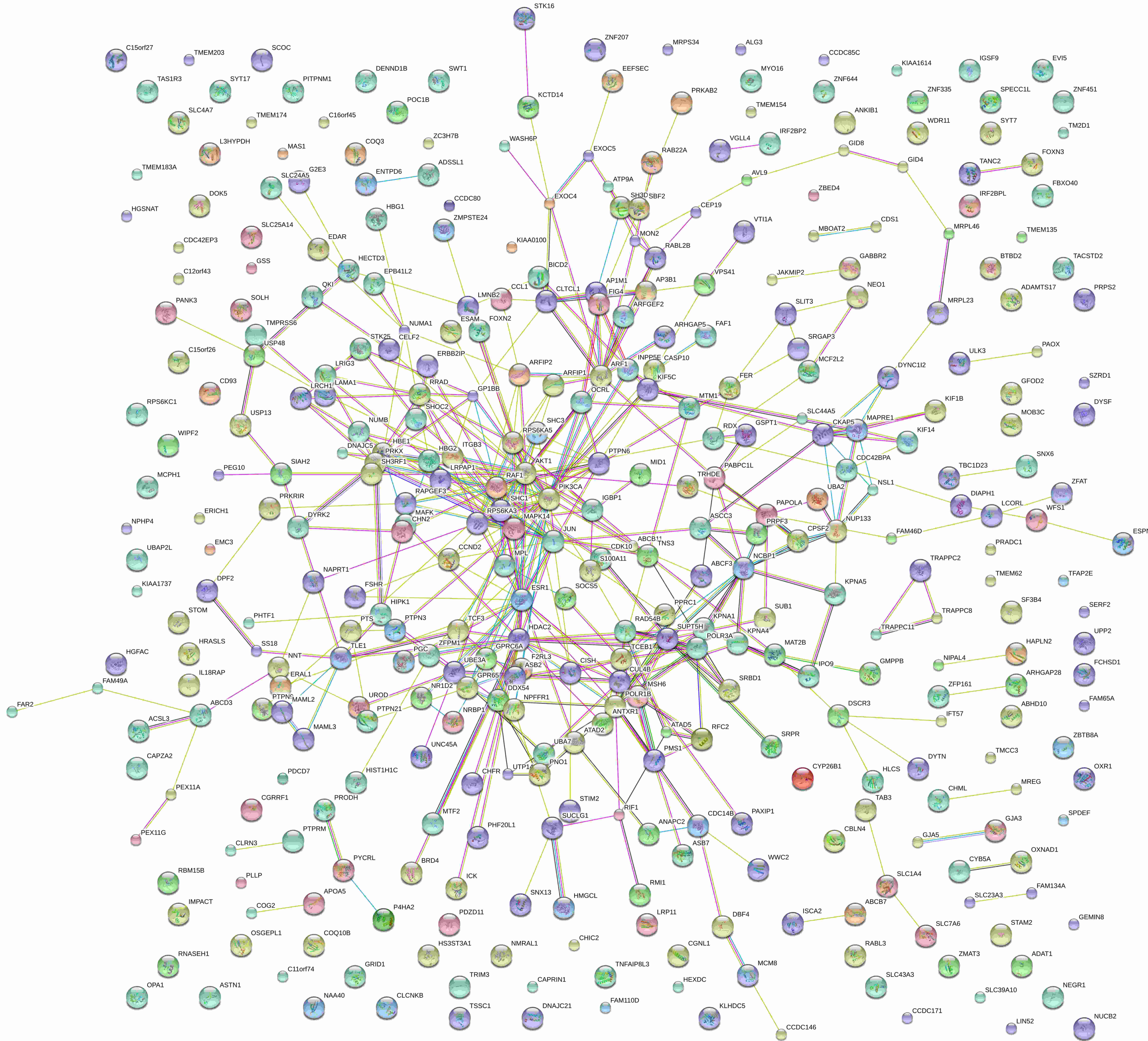

Supplement: Supplementary file 6 — Network analysis figures. All figures were converted to pdf files. (ZIP 47344 kb) [file 12192_2018_954_MOESM6_ESM.zip › Spleen Highland noon-evening -string .pdf]

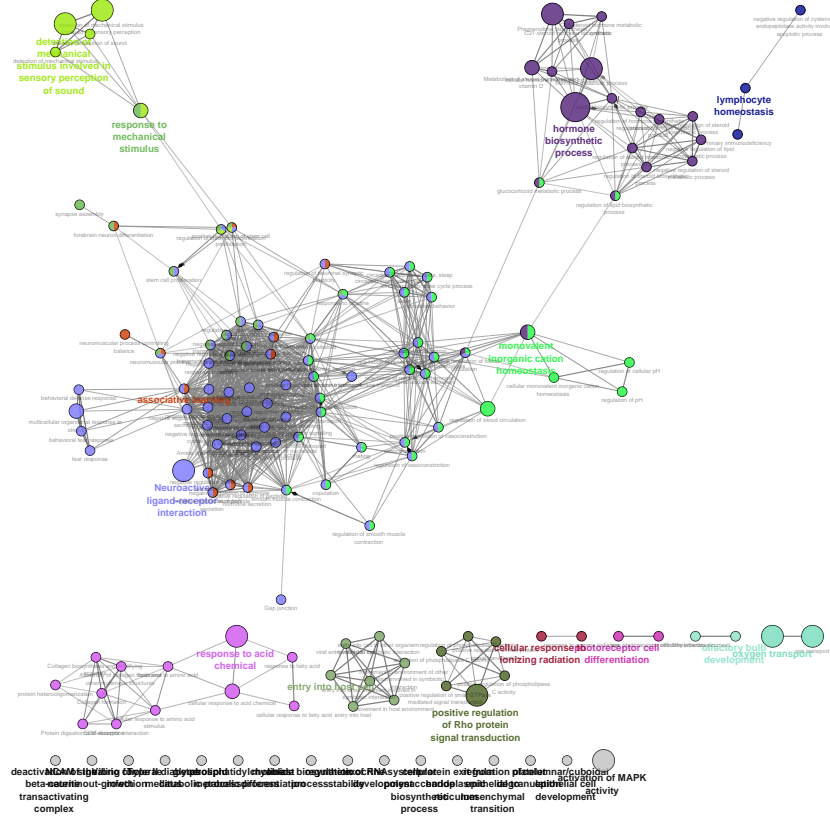

Supplement: Supplementary file 6 — Network analysis figures. All figures were converted to pdf files. (ZIP 47344 kb) [file 12192_2018_954_MOESM6_ESM.zip › Spleen Highland-lowland all - Cytoscape-ClueGo.pdf]

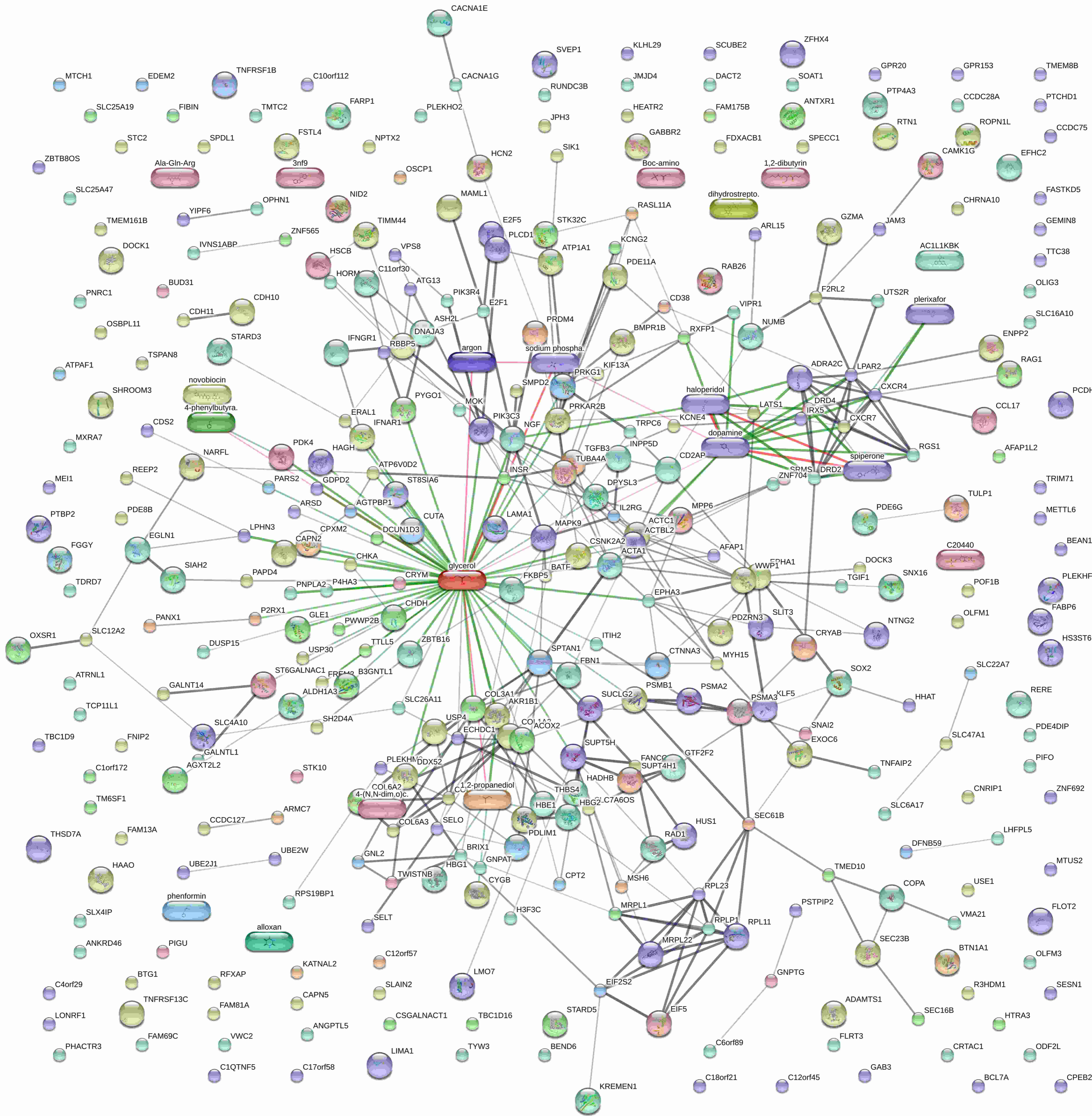

Supplement: Supplementary file 6 — Network analysis figures. All figures were converted to pdf files. (ZIP 47344 kb) [file 12192_2018_954_MOESM6_ESM.zip › Spleen Highland-lowland all - stitche.pdf]

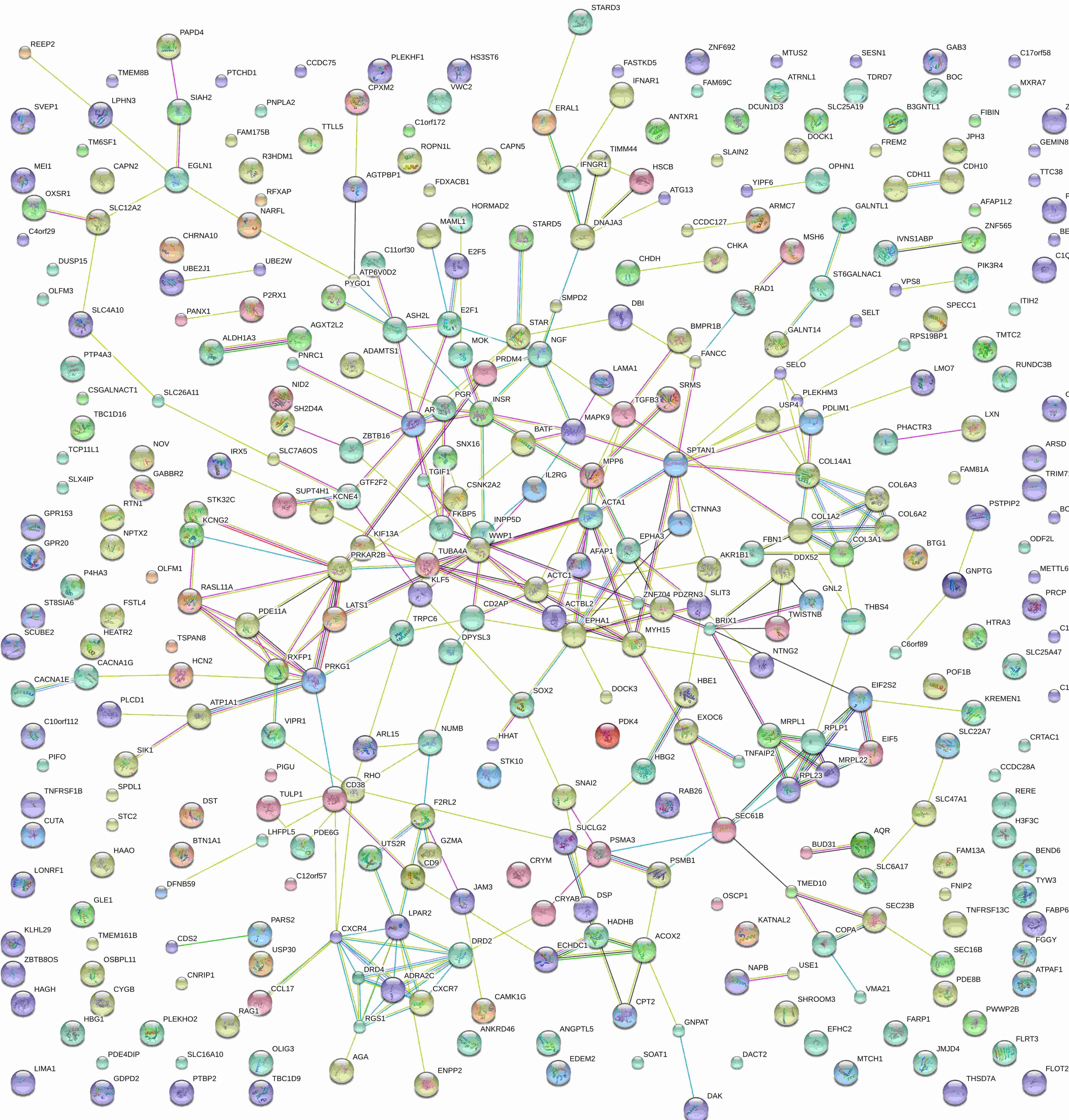

Supplement: Supplementary file 6 — Network analysis figures. All figures were converted to pdf files. (ZIP 47344 kb) [file 12192_2018_954_MOESM6_ESM.zip › Spleen Highland-lowland all - string.pdf]

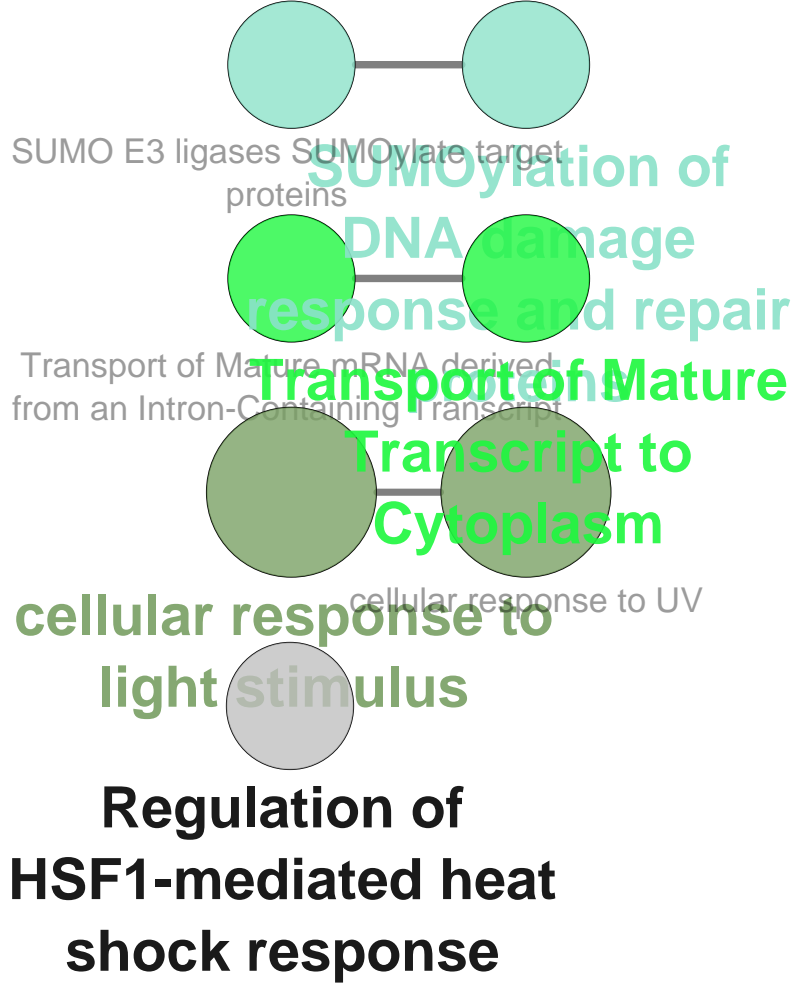

Supplement: Supplementary file 6 — Network analysis figures. All figures were converted to pdf files. (ZIP 47344 kb) [file 12192_2018_954_MOESM6_ESM.zip › Spleen Highland-lowland evening - Cytoscape-ClueGo.pdf]

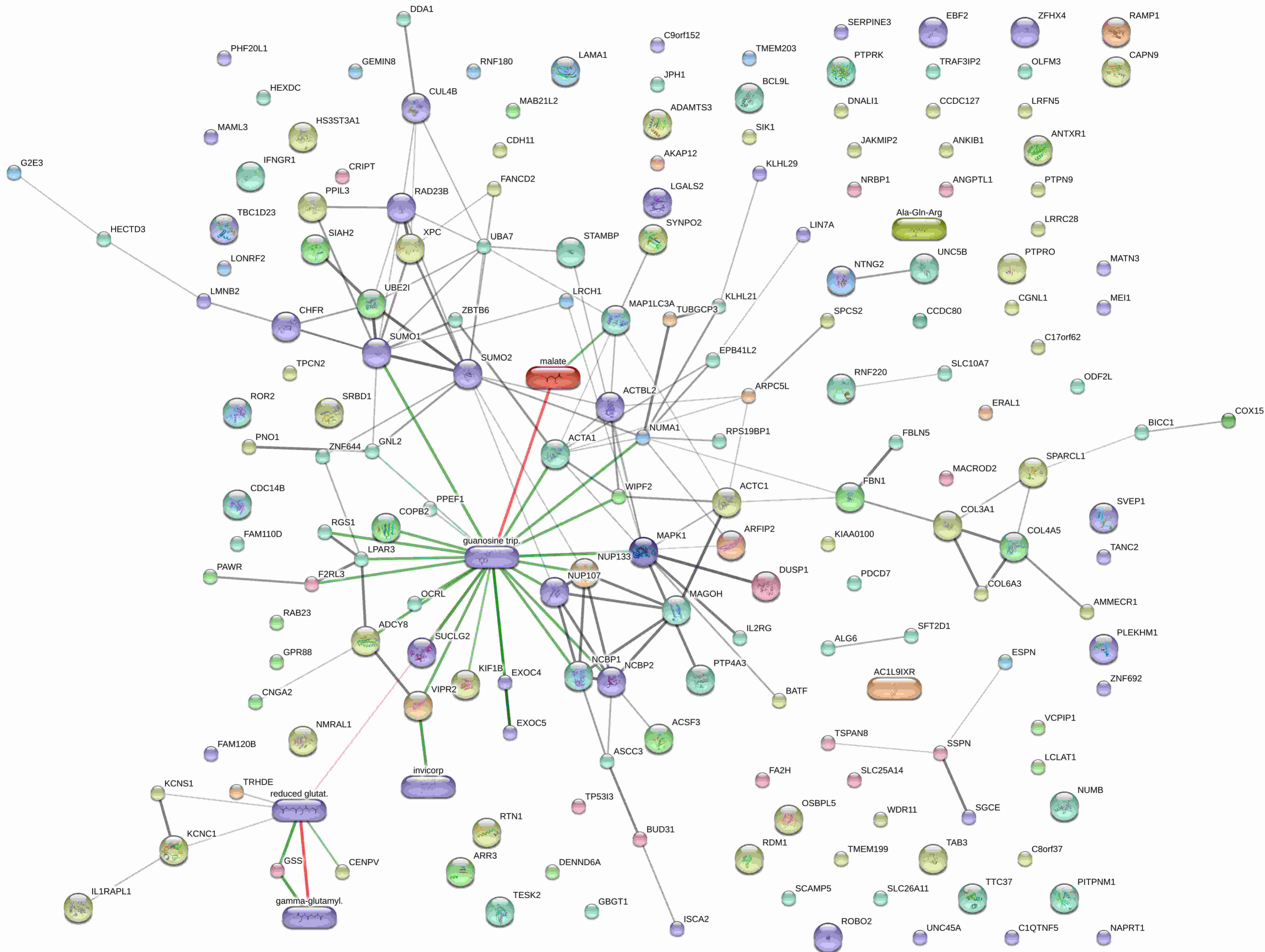

Supplement: Supplementary file 6 — Network analysis figures. All figures were converted to pdf files. (ZIP 47344 kb) [file 12192_2018_954_MOESM6_ESM.zip › Spleen Highland-lowland evening - stitch.pdf]

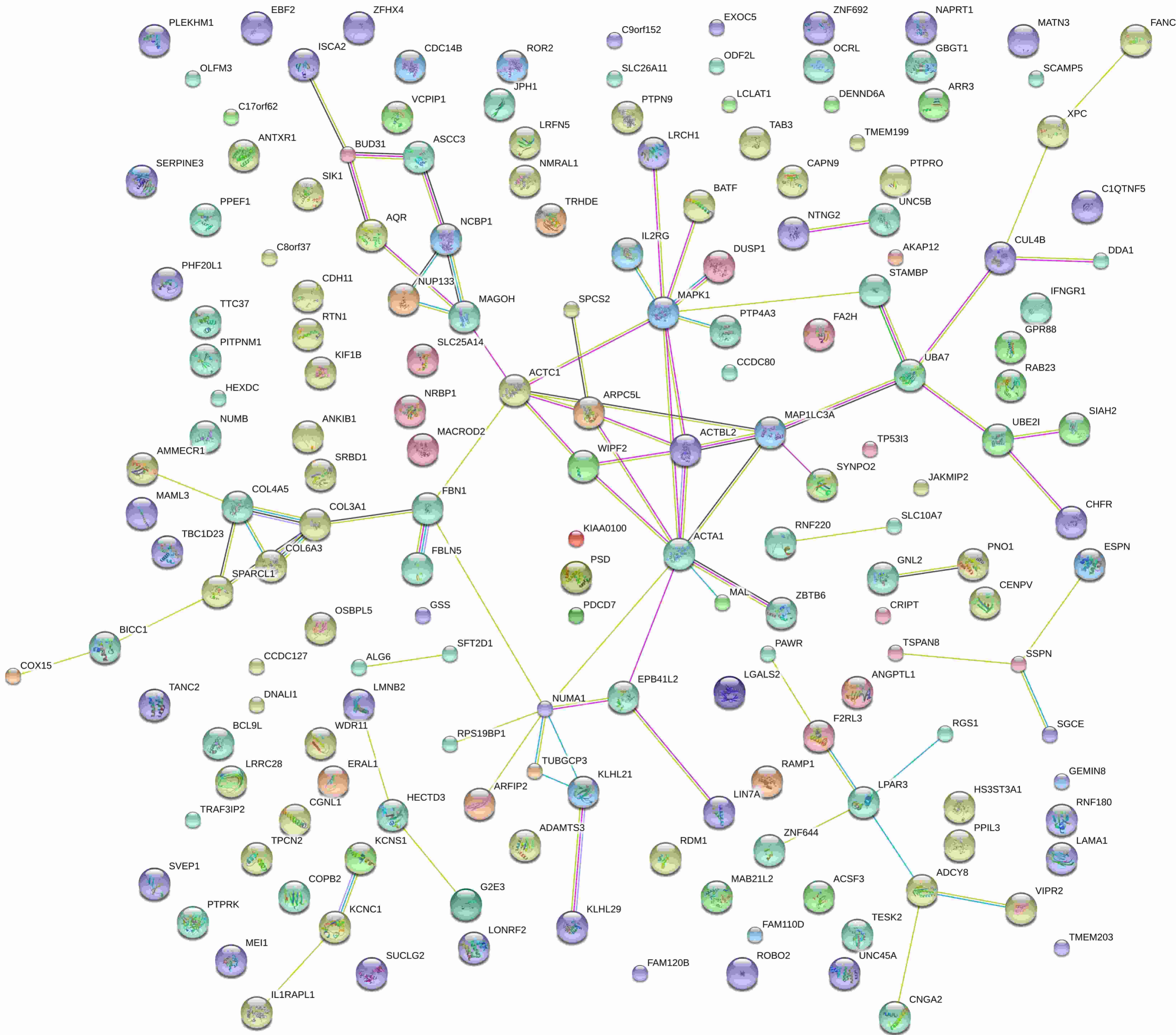

Supplement: Supplementary file 6 — Network analysis figures. All figures were converted to pdf files. (ZIP 47344 kb) [file 12192_2018_954_MOESM6_ESM.zip › Spleen Highland-lowland evening - string.pdf]

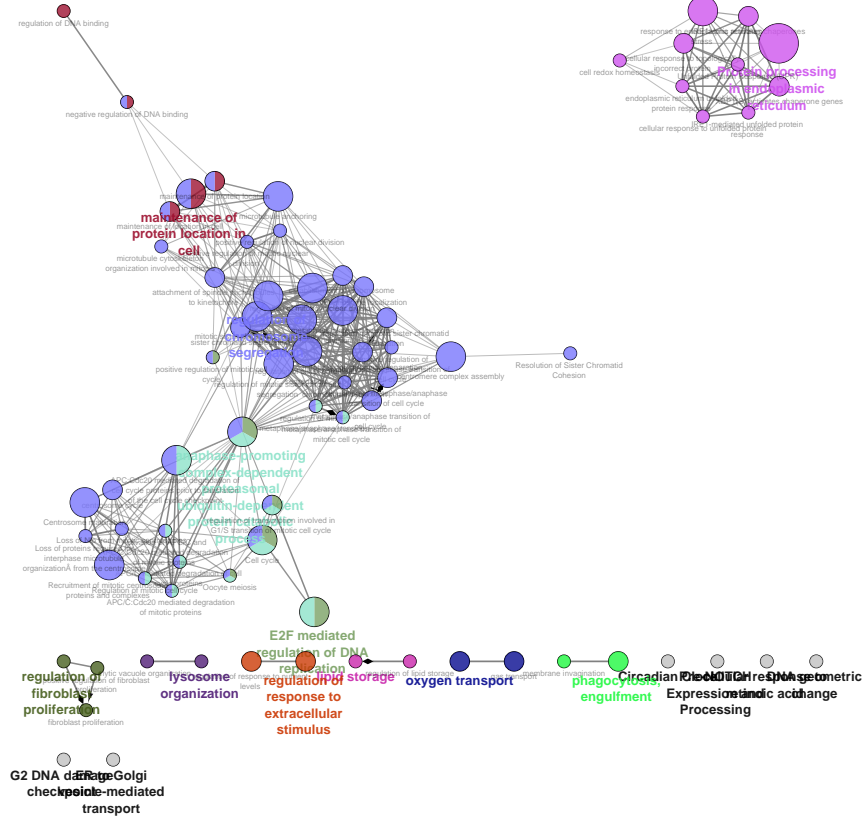

Supplement: Supplementary file 6 — Network analysis figures. All figures were converted to pdf files. (ZIP 47344 kb) [file 12192_2018_954_MOESM6_ESM.zip › Spleen Highland-lowland morning - Cytoscape-ClueGo.pdf]

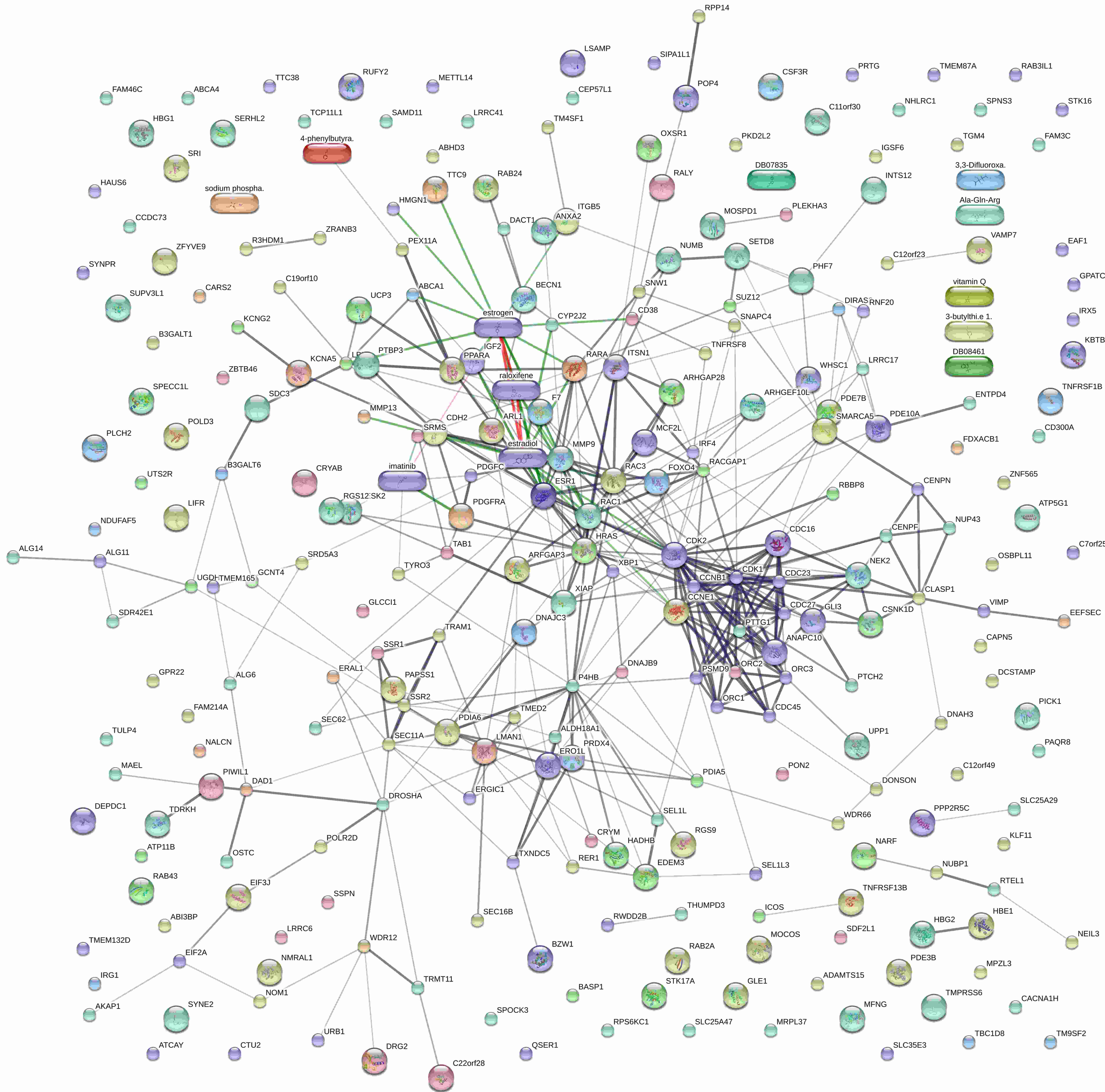

Supplement: Supplementary file 6 — Network analysis figures. All figures were converted to pdf files. (ZIP 47344 kb) [file 12192_2018_954_MOESM6_ESM.zip › Spleen Highland-lowland morning - stitch.pdf]

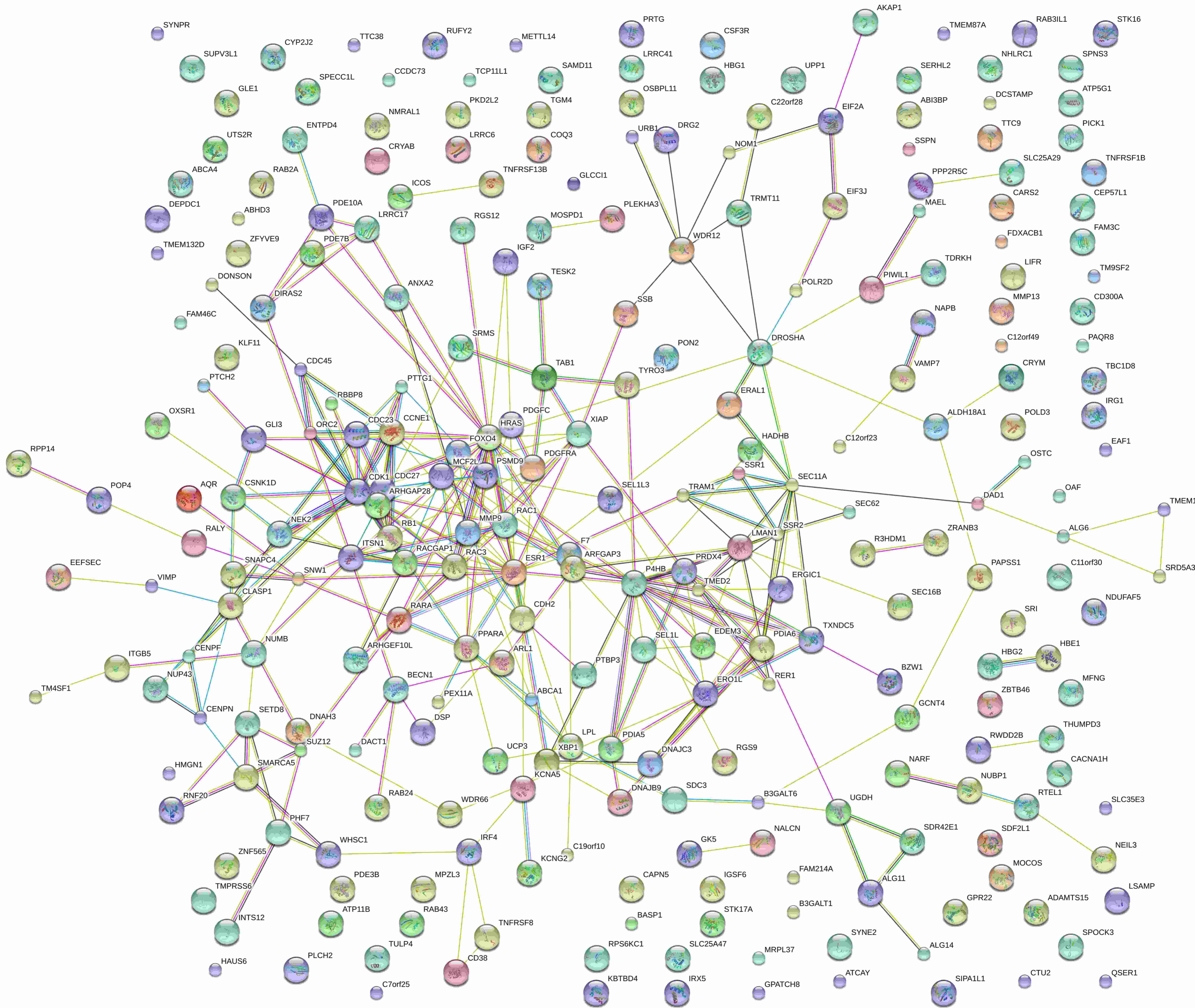

Supplement: Supplementary file 6 — Network analysis figures. All figures were converted to pdf files. (ZIP 47344 kb) [file 12192_2018_954_MOESM6_ESM.zip › Spleen Highland-lowland morning - string.pdf]

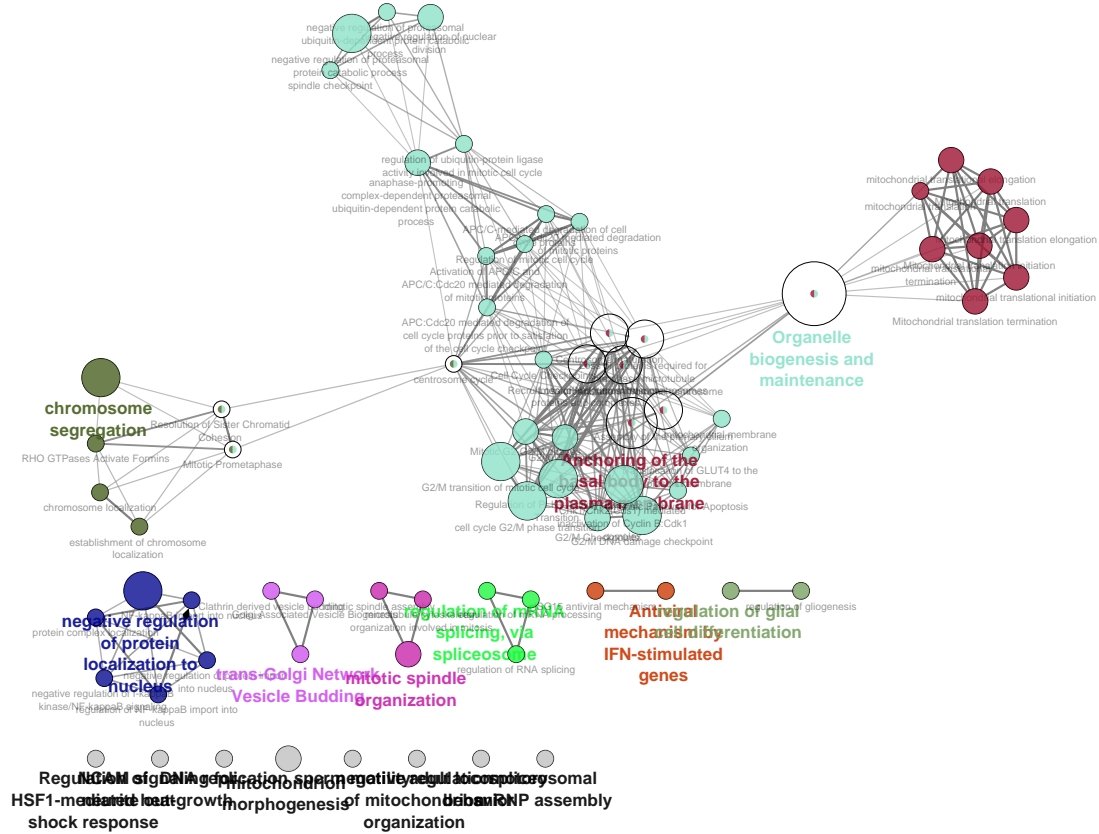

Supplement: Supplementary file 6 — Network analysis figures. All figures were converted to pdf files. (ZIP 47344 kb) [file 12192_2018_954_MOESM6_ESM.zip › Spleen Highland-lowland noon - Cytoscape-ClueGo.pdf]

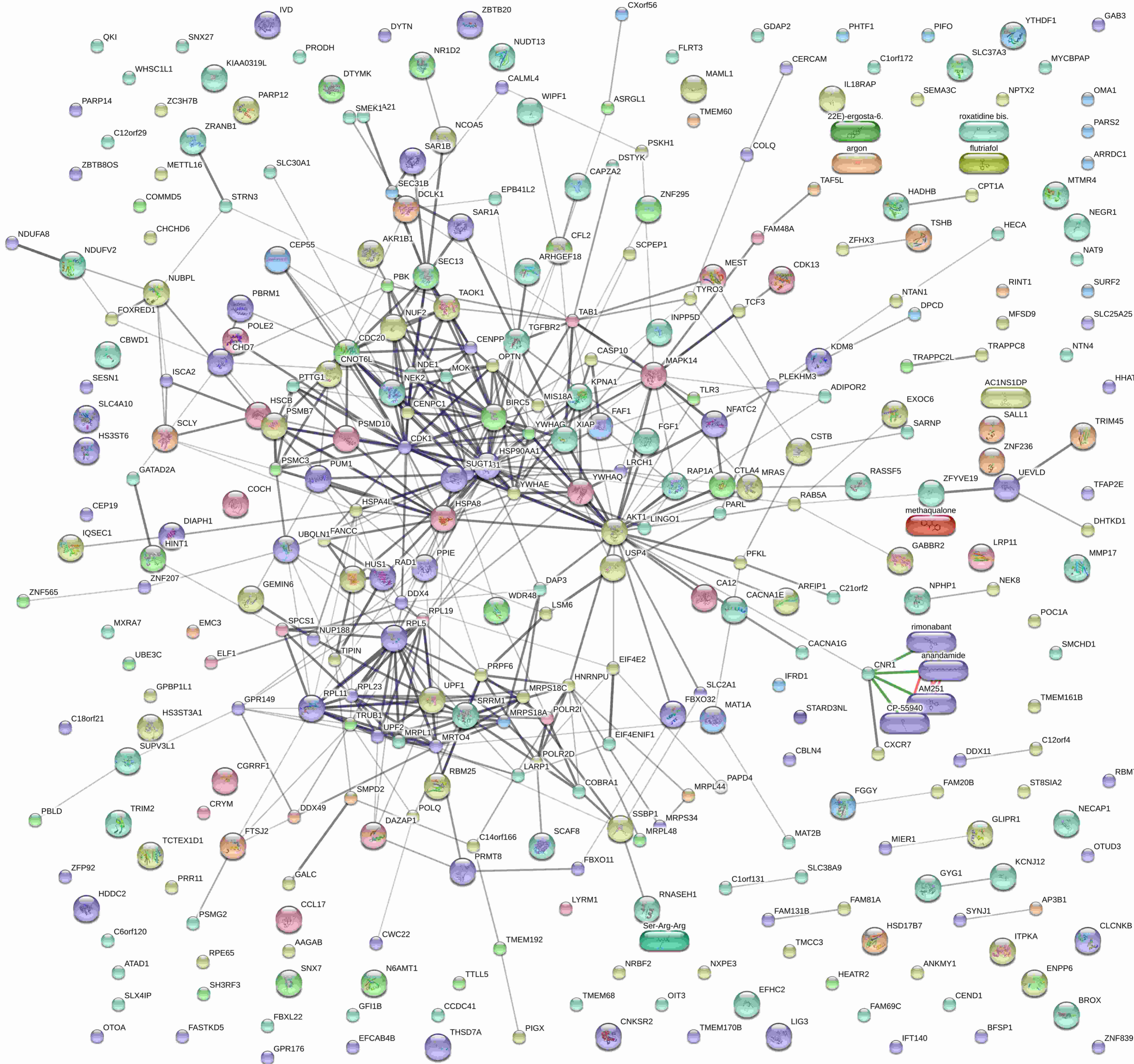

Supplement: Supplementary file 6 — Network analysis figures. All figures were converted to pdf files. (ZIP 47344 kb) [file 12192_2018_954_MOESM6_ESM.zip › Spleen Highland-lowland noon - stitch.pdf]

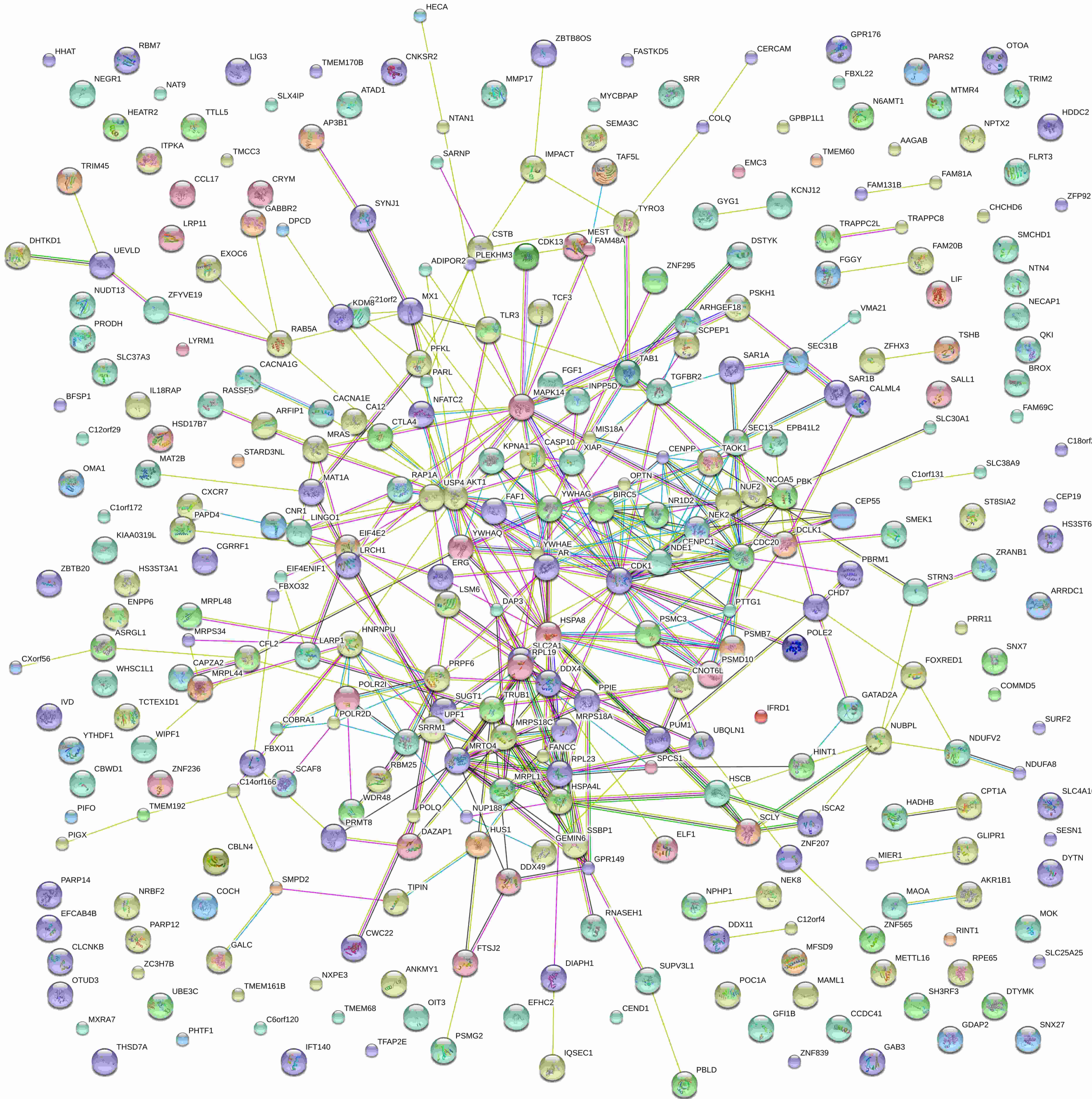

Supplement: Supplementary file 6 — Network analysis figures. All figures were converted to pdf files. (ZIP 47344 kb) [file 12192_2018_954_MOESM6_ESM.zip › Spleen Highland-lowland noon - string.pdf]

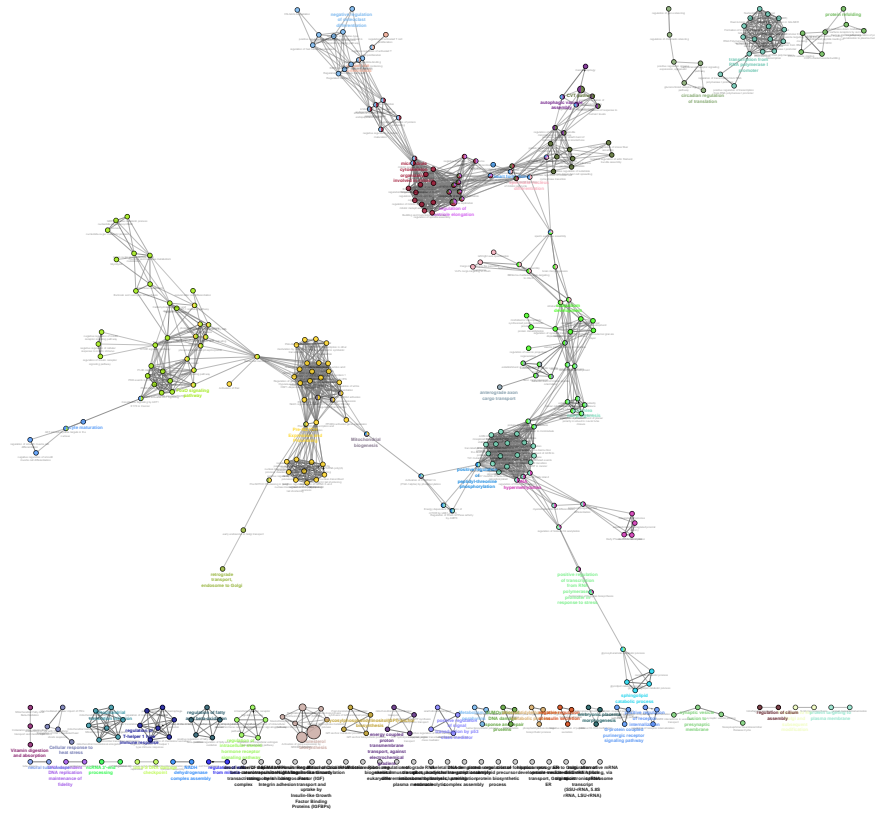

Supplement: Supplementary file 6 — Network analysis figures. All figures were converted to pdf files. (ZIP 47344 kb) [file 12192_2018_954_MOESM6_ESM.zip › Spleen Lowland all - Cytoscape-ClueGo HS.pdf]

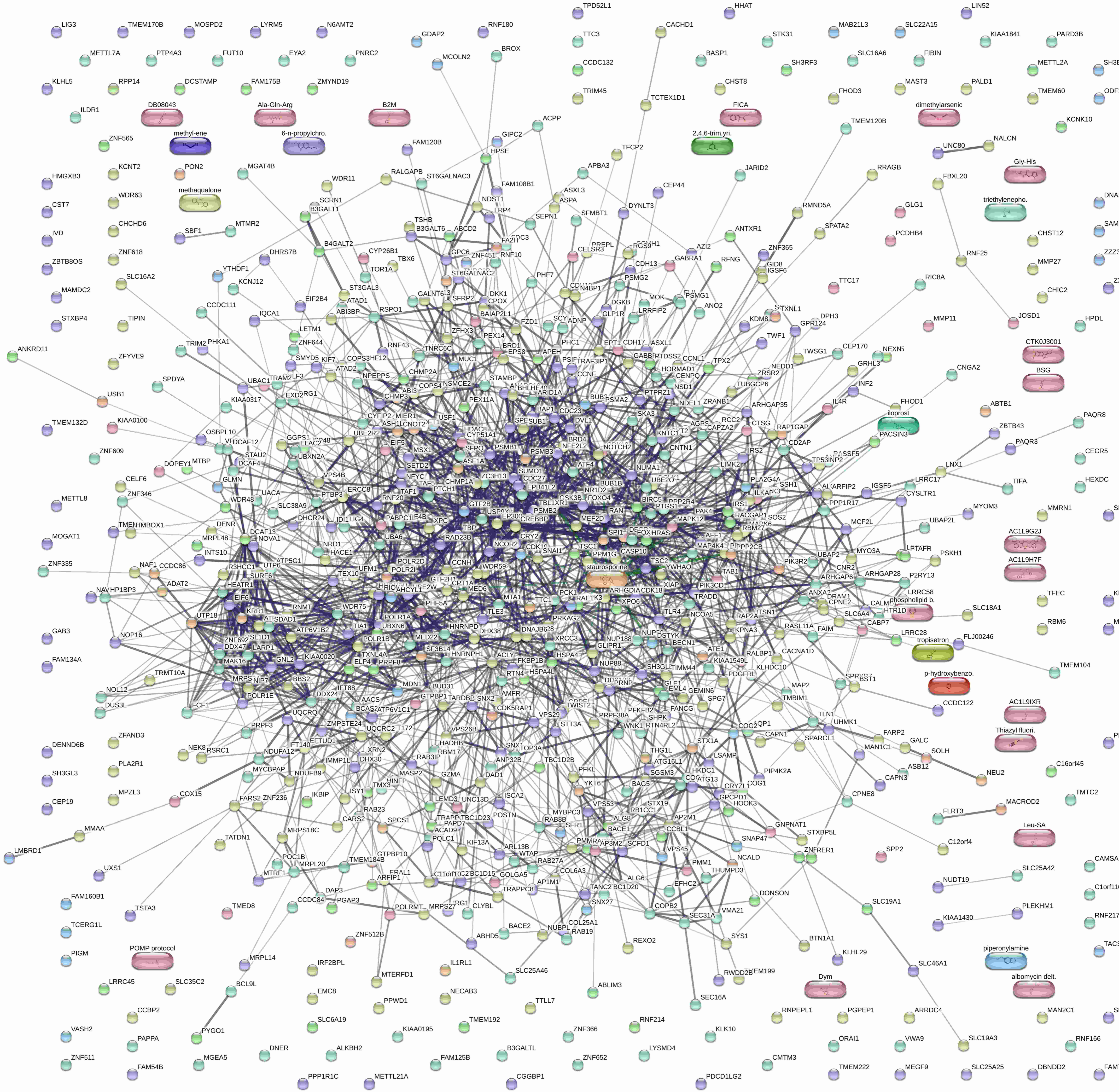

Supplement: Supplementary file 6 — Network analysis figures. All figures were converted to pdf files. (ZIP 47344 kb) [file 12192_2018_954_MOESM6_ESM.zip › Spleen Lowland all - stitch.pdf]

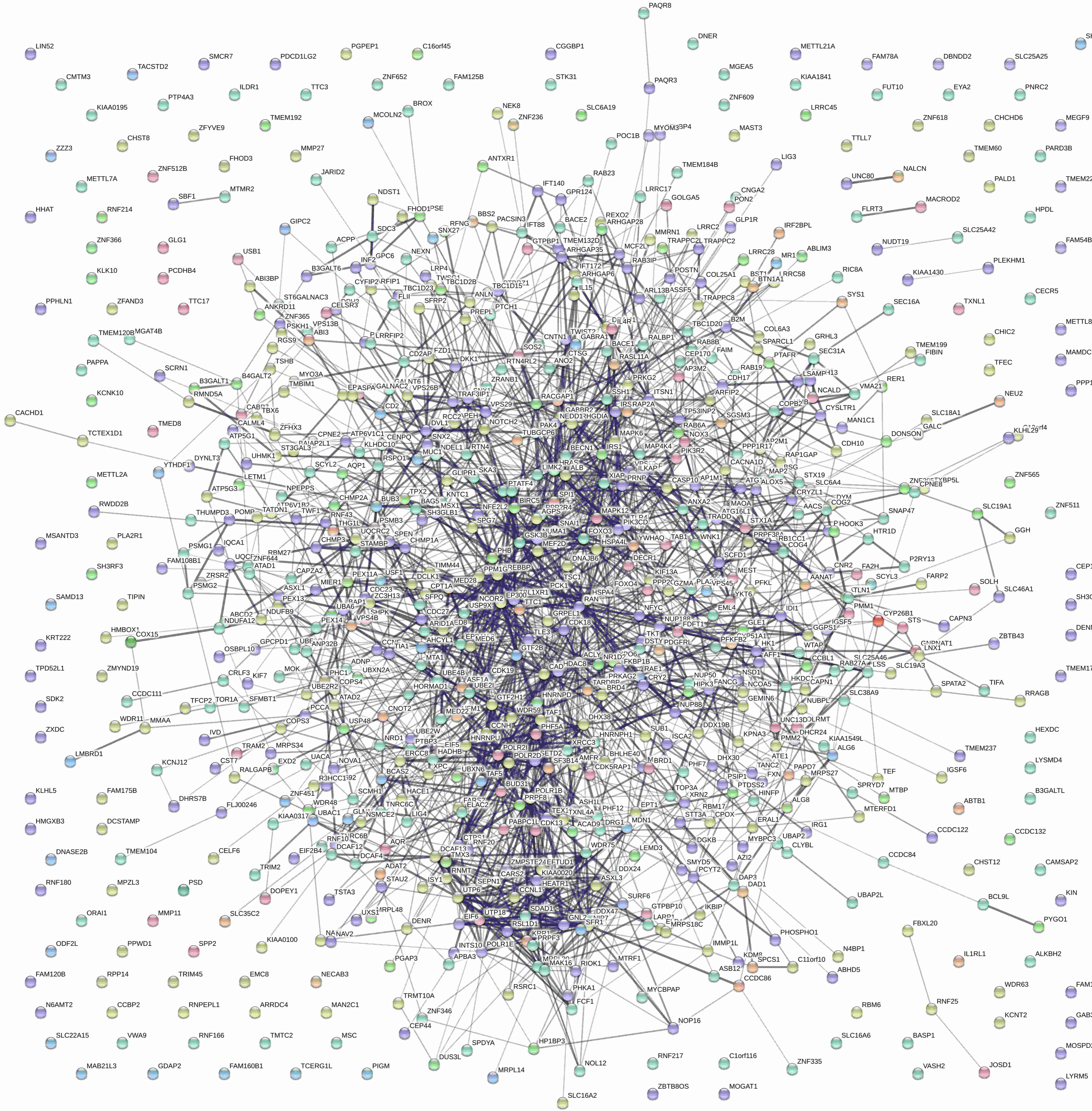

Supplement: Supplementary file 6 — Network analysis figures. All figures were converted to pdf files. (ZIP 47344 kb) [file 12192_2018_954_MOESM6_ESM.zip › Spleen Lowland all - string.pdf]

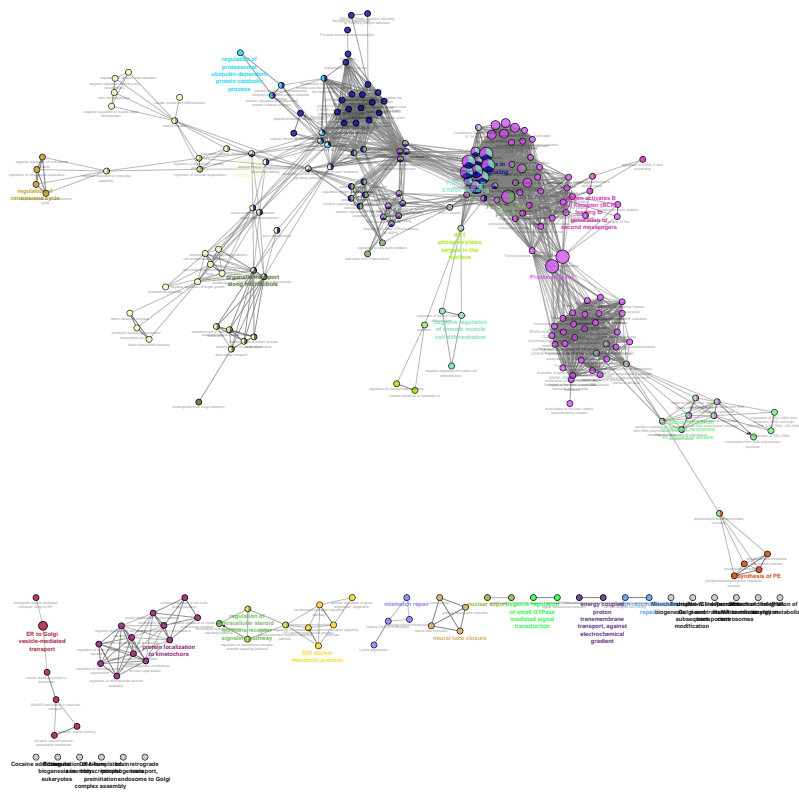

Supplement: Supplementary file 6 — Network analysis figures. All figures were converted to pdf files. (ZIP 47344 kb) [file 12192_2018_954_MOESM6_ESM.zip › Spleen Lowland morning-evening - Cytoscape-ClueGo.pdf]

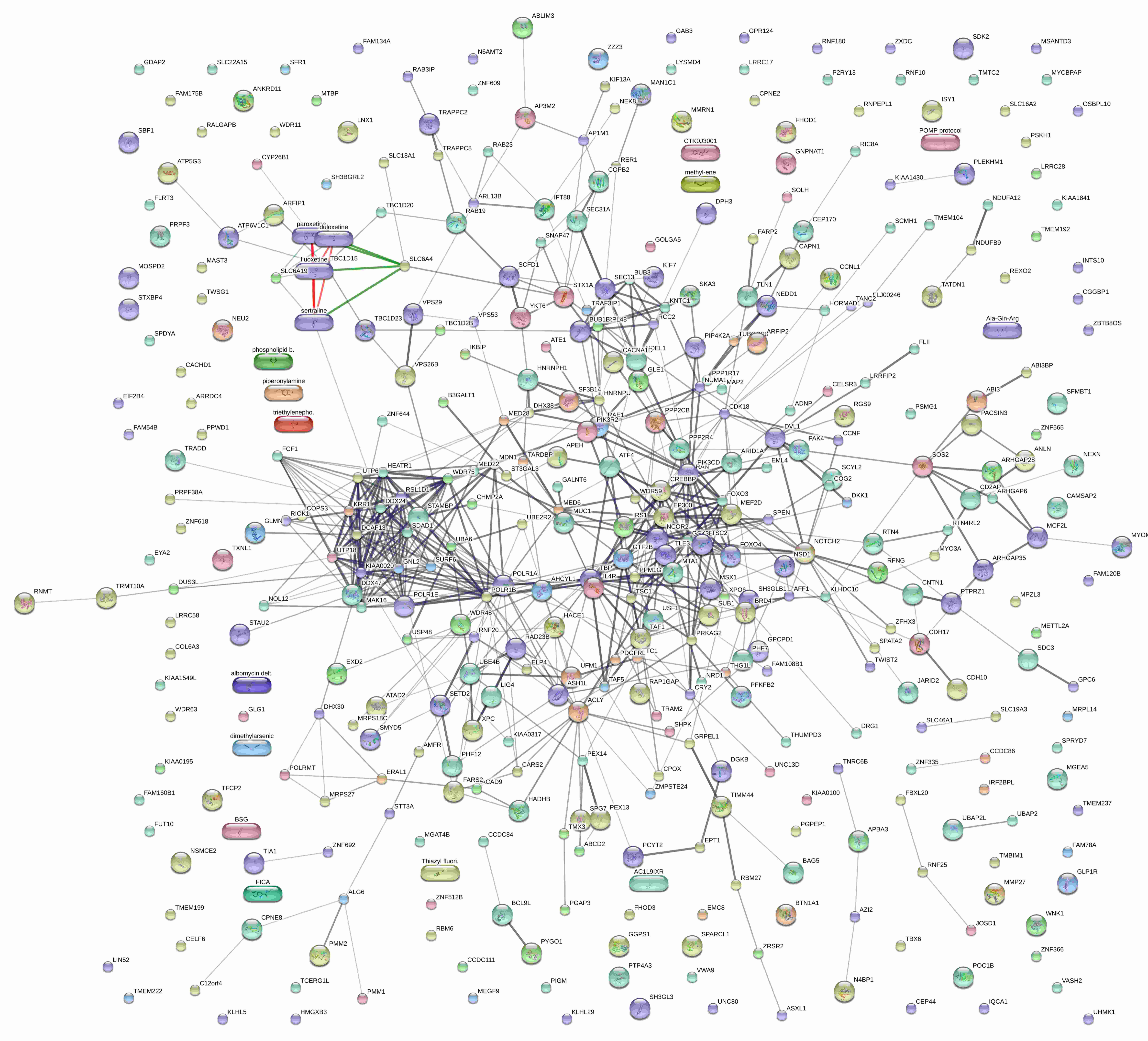

Supplement: Supplementary file 6 — Network analysis figures. All figures were converted to pdf files. (ZIP 47344 kb) [file 12192_2018_954_MOESM6_ESM.zip › Spleen Lowland morning-evening - stitch.pdf]

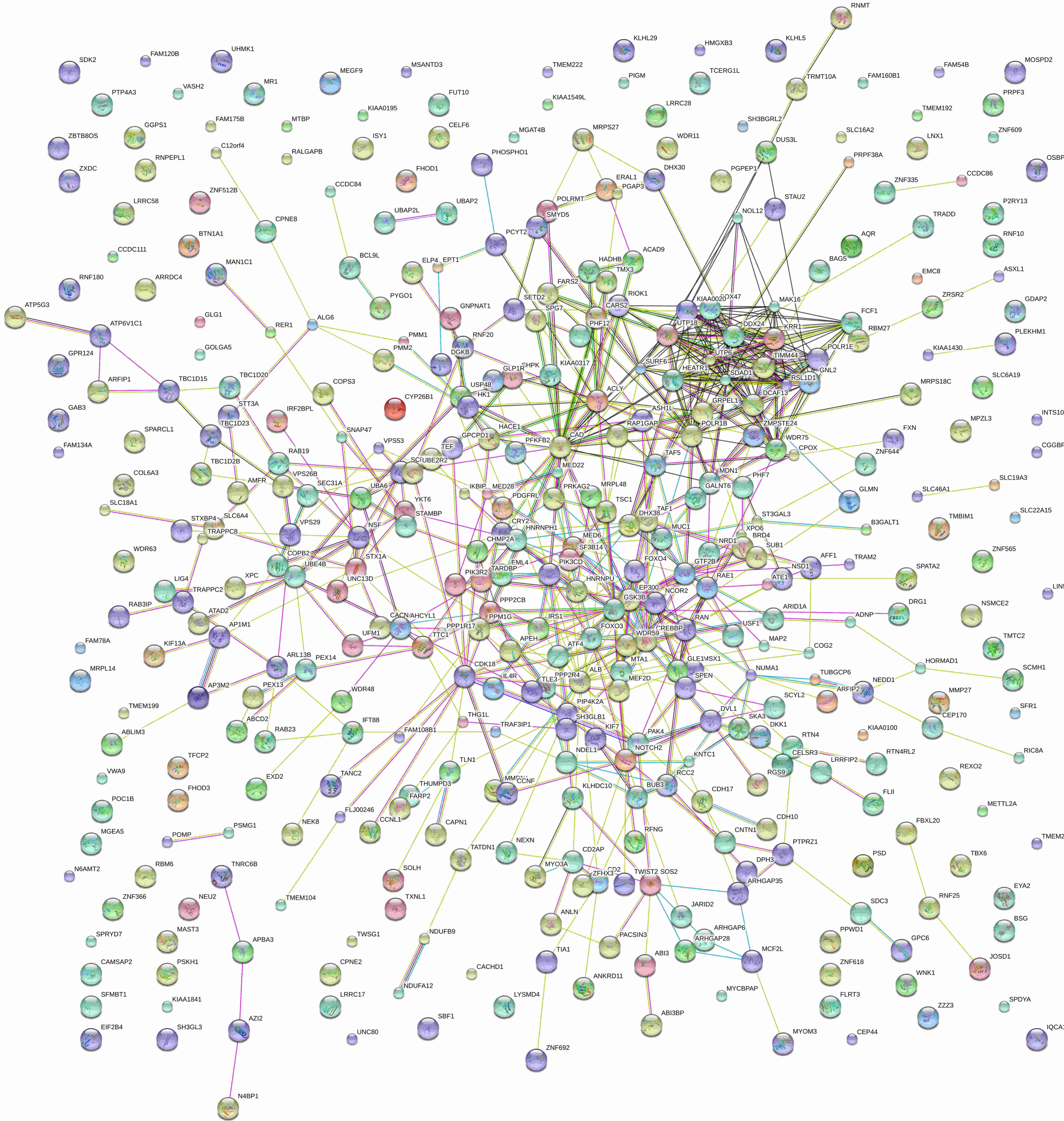

Supplement: Supplementary file 6 — Network analysis figures. All figures were converted to pdf files. (ZIP 47344 kb) [file 12192_2018_954_MOESM6_ESM.zip › Spleen Lowland morning-evening - string.pdf]

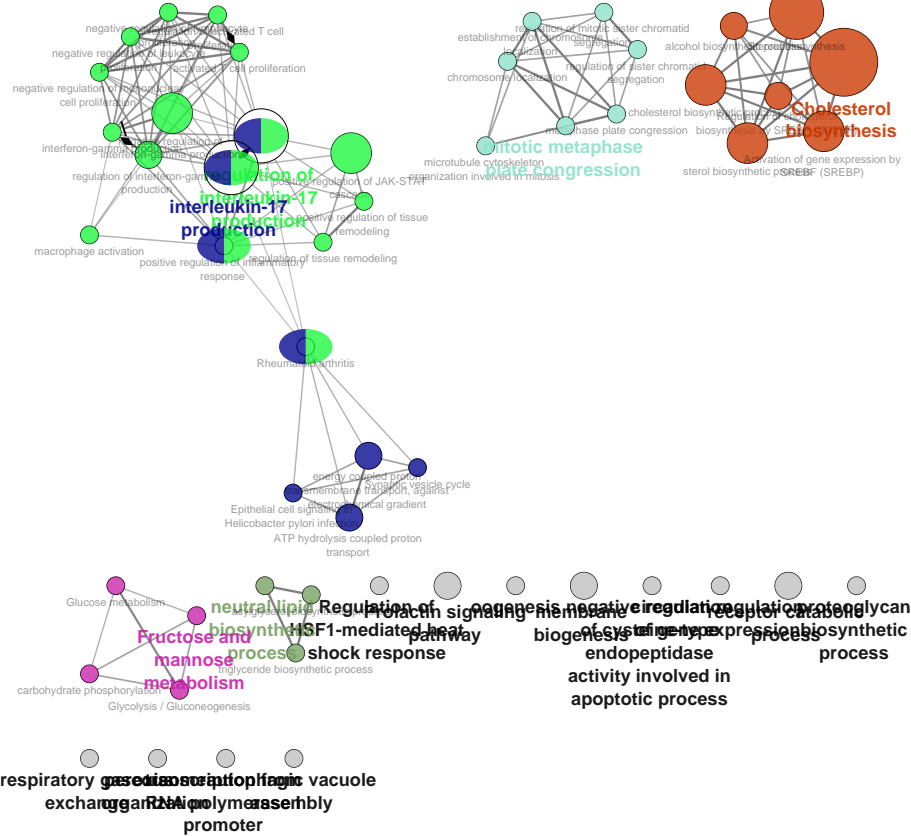

Supplement: Supplementary file 6 — Network analysis figures. All figures were converted to pdf files. (ZIP 47344 kb) [file 12192_2018_954_MOESM6_ESM.zip › Spleen Lowland morning-noon - Cytoscape-ClueGo.pdf]
